# A genome‐wide association meta‐analysis of all‐cause and vascular dementia

**DOI:** 10.1002/alz.14115

**Published:** 2024-07-24

**Authors:** The Mega Vascular Cognitive Impairment and Dementia (MEGAVCID) consortium, Bernard Fongang, Muralidharan Sargurupremraj, Xueqiu Jian, Aniket Mishra, Vincent Damotte, Itziar de Rojas, Olivia Skrobot, Joshua C. Bis, Kang‐Hsien Fan, Erin Jacobsen, Gloria Hoi‐Yee Li, Jingyun Yang, Bizzarro Alessandra, Lauria Alessandra, Saima Hilal, Joyce Ruifen Chong, Yuek Ling Chai, M. J. Knol, Maria Pina Concas, Girotto Giorgia, Moeen Riaz, Chenglong Yu, Alexander Guojonsson, Paul Lacaze, Adam C Naj, Monica Gireud‐Goss, Yannick N. Wadop, Aicha Soumare, Vincent Bouteloup, Vilmundur Gudnason, Petronilla Battista, Aurora Santin, Beatrice Spedicati, Rodolfo Sardone, Lenore Launer, Jan Bressler, Rebecca F Gottesman, Quentin Le Grand, Ilana Caro, Gennady V. Roshchupkin, Hampton L. Leonard, Chaojie Yang, Traci M. Bartz, Constance Bordes, Paul M. Ridker, Mirjam I. Geerlings, Natalie C. Gasca, Ani Manichaikul, Mike A. Nalls, Stephen S. Rich, Carsten O. Schmidt, Stella Trompet, Jessica van Setten, Marion van Vugt, Hans J. Grabe, J Wouter Jukema, Ina L. Rissanen, Sylvia Wassertheil‐Smoller, M. Arfan Ikram, Eleanor M. Simonsick, W T. Longstreth, Daniel I. Chasman, Jerome I. Rotter, Naveed Sattar, David J Stott, Eric J Shiroma, Sigurdur Sigurdsson, Mohsen Ghanbari, Ulf Schminke, Eric Boerwinkle, Hugo J Aparicio, Alexa S Beiser, Jose R Romero, Vasileios Lioutas, Ruiqi Wang, Chloe Sarnowski, Alexander Teumer, Uwe Völker, Thomas H. Mosley, Marta Marquié, Pablo García‐González, Clàudia Olivé, Raquel Puerta, Amanda Cano, Oscar Sotolongo‐Grau, Sergi Valero, Vanesa Veronica Pytel, Maitée Rosende‐Roca, Montserrat Alegret, Lluís Tàrraga, Mercè Boada, Ángel Carracedo, Emilio Franco‐Macías, Gerard Piñol‐Ripoll, Guillermo Garcia‐Ribas, Jordi Pérez‐Tur, Jose Luís Royo, Jose María García‐Alberca, Luis Miguel Real, María Eugenia Sáez, María J. Bullido, Miguel Calero, Miguel Medina, Pablo Mir, Pascual Sánchez‐Juan, Pau Pastor, Victoria Álvarez, Benjamin Grenier‐Boley, Fahri Küçükali, Sven Van der Lee, Oliver Peters, Anja Schneider, Martin Dichgans, Dan Rujescu, Jürgen Deckert, Emrah Düzel, Jens Wiltfang, Michael Wagner, Timo Grimmer, Nikolaos Scarmeas, Fermin Moreno, Raquel Sánchez‐Valle, Luis M Real, Eloy Rodriguez‐Rodriguez, Adolfo Lopez de Munain, Alexandre de Mendonça, Jakub Hort, Caroline Graff, Goran Papenberg, Vilmantas Giedraitis, Børge G. Nordestgaard, Hilkka Soininen, Miia Kivipelto, Annakaisa Haapasalo, Gael Nicolas, Florence Pasquier, Olivier Hanon, Edna Grünblatt, Daniela Galimberti, Beatrice Arosio, Patrizia Mecocci, Alessio Squassina, Lucio Tremolizzo, Innocenzo Rainero, Davide Seripa, Julie Williams, Philippe Amouyel, Frank Jessen, Tsolaki Magda, Ruth Frikke‐Schmidt, Kristel Sleegers, Sebastiaan Engelborghs, Rik Vandenberghe, Martin Ingelsson, Giacomina Rossi, Mikko Hiltunen, Rebecca Sims, Magdalena Gugała‐Iwaniuk, Mitchell K. P. Lai, N Venketasubramanian, Boon‐Yeow Tan, Angelo Baldassare Cefalù, Nicola J Armstrong, Roberta Baschi, Regis bordet, Anne‐Marie Bordet, Henry Brodaty, Srdjan Djurovic, Grazia D'Onofrio, Margaret Esiri, Patrick Gelé, Teresa Juarez‐Cedillo, Raj Kalaria, Pekka Karhunen, Jan LACZO, Ondrej LERCH, Carlo Masullo, Karen A Mather, Vaclav MATOSKA, Susanna Melkas, Roberto Monastero, Katya Numbers, Francesco Panza, Tuomo M Polvikoski, Joe Quinn, Arvid Rongve, Perminder S Sachdev, Michela Scamosci, Anbupalam Thalamuthu, Anne Tybjærg‐Hansen, Martin VYHNALEK, Shawn K. Westaway, Amy E Martinsen, Anne Heidi Skogholt, Cristen J Willer, Eystein Stordal, Geir Bråthen, Jonas Bille Nielsen, Lars G Fritsche, Laurent F Thomas, Linda M Pedersen, Maiken E Gabrielsen, Ole Kristian Drange, Sigrid Botne Sando, Tore Wergeland Meisingset, Genevieve Chene, Wei Zhou, Christophe Tzourio, Adrienne Tin, Oscar L Lopez, Haan Mary, Allison E Aiello, Sigrid Børte, Ingunn Bosnes, Cornelia van Duijn, Ching‐Lung Cheung, David A Bennett, Christopher Chen, M. Ilyas Kamboh, Claudia Satizabal, M. Kamran Ikram, Hieab Adams, Yang Qiong, Gerard D. Schellenberg, Geir Selbæk, Kristian Hveem, Ole A Andreassen, Alfredo Ramirez, Carole Dufouil, Wiesje van der Flier, John‐Anker Zwart, Stéphanie Debette, Myriam Fornage, Bendik Winsvold, Jean‐Charles Lambert, Agustin Ruiz, Patrick G. Kehoe, Galit Weinstein, Sudha Seshadri

**Affiliations:** ^1^ Glenn Biggs Institute for Alzheimer's & Neurodegenerative Diseases University of Texas Health Science Center San Antonio Texas USA; ^2^ Department of Biochemistry and Structural Biology University of Texas Health Science Center San Antonio Texas USA; ^3^ Department of Population Health Sciences University of Texas Health Science Center San Antonio Texas USA; ^4^ University of Bordeaux Inserm Bordeaux Population Health Research Center Bordeaux France; ^5^ Univ. Lille, Inserm, CHU Lille, Institut Pasteur de Lille, U1167‐RID‐AGE facteurs de risque et déterminants moléculaires des maladies liés au vieillissement Lille France; ^6^ Research Center and Memory Clinic ACE Alzheimer Center Barcelona. Universitat Internacional de Catalunya Catalonia Spain; ^7^ Network Center for Biomedical Research in Neurodegenerative Diseases (CIBERNED) Instituto de Salud Carlos III Madrid Spain; ^8^ Population Health Sciences Bristol Medical School University of Bristol Bristol UK; ^9^ Cardiovascular Health Research Unit Department of Medicine University of Washington Seattle Washington USA; ^10^ Department of Human Genetics School of Public Health University of Pittsburgh Pittsburgh Pennsylvania USA; ^11^ Department of Psychiatry and Neurology School of Medicine University of Pittsburgh Pittsburgh Pennsylvania USA; ^12^ Department of Health Technology and Informatics The Hong Kong Polytechnic University Hung Hom Hong Kong; ^13^ Rush Alzheimer's Disease Center and Department of Neurological Sciences Rush University Medical Center Chicago Illinois USA; ^14^ Geriatrics Unit, Policlinico Universitario Fondazione Agostino Gemelli IRCCS Rome Italy; ^15^ Department of Pharmacology National University of Singapore Singapore Singapore; ^16^ Saw Swee Hock School of Public Health National University of Singapore and National University Health System Singapore Singapore; ^17^ Department of Epidemiology Erasmus MC University Medical Center Rotterdam the Netherlands; ^18^ Institute for Maternal and Child Health IRCCS Burlo Garofolo Trieste Italy; ^19^ Department of Medicine Surgery and Health Sciences University of Trieste Trieste Italy; ^20^ Department of Epidemiology and Preventive Medicine Monash University Melbourne VIC Australia; ^21^ Faculty of Medicine University of Iceland Reykjavik Iceland; ^22^ Department of Biostatistics and Epidemiology/Center for Clinical Epidemiology and Biostatistics University of Pennsylvania Perelman School of Medicine Philadelphia Pennsylvania USA; ^23^ Pôle de Santé Publique Centre Hospitalier Universitaire (CHU) de Bordeaux Bordeaux France; ^24^ Icelandic Heart Association Kopavogur Iceland; ^25^ Istituti Clinici Scientifici Maugeri IRCCS Laboratory of Neuropsychology Bari Institute Bari Italy; ^26^ Department of Translational Biomedicine and Neuroscience University of Bari “Aldo Moro ” Bari Italy; ^27^ Unit of Statistics and Epidemiology Local Healthcare Authority of Taranto Taranto Italy; ^28^ Laboratory of Epidemiology and Population Sciences Intramural Research Program National Institute of Aging National Institutes of Health Bethesda Maryland USA; ^29^ Human Genetics Center School of Public Health The University of Texas Health Science Center at Houston Houston Texas USA; ^30^ Stroke Branch National Institute of Neurological Disorders and Stroke Intramural Program National Institutes of Health Bethesda Maryland USA; ^31^ University of Bordeaux Inserm Bordeaux Population Health Research Center Bordeaux France; ^32^ Department of Epidemiology Erasmus MC University Medical Center Rotterdam Rotterdam the Netherlands; ^33^ Department of Radiology and Nuclear Medicine Erasmus MC University Medical Center Rotterdam the Netherlands; ^34^ Center for Alzheimer's and Related Dementias National Institutes of Health Bethesda Maryland USA; ^35^ Laboratory of Neurogenetics National Institute on Aging National Institutes of Health Bethesda Maryland USA; ^36^ Data Tecnica International LLC Glen Echo Maryland USA; ^37^ Center for Public Health Genomics University of Virginia Charlottesville Virginia USA; ^38^ Department of Biochemistry and Molecular Genetics University of Virginia Charlottesville Virginia USA; ^39^ Cardiovascular Health Research Unit Department of Medicine University of Washington Seattle Washington USA; ^40^ Department of Biostatistics University of Washington Seattle Washington USA; ^41^ Division of Preventive Medicine Brigham and Women's Hospital Boston Massachusetts USA; ^42^ Harvard Medical School Boston Massachusetts USA; ^43^ Department of General Practice Amsterdam UMC location University of Amsterdam Amsterdam the Netherlands; ^44^ Amsterdam Public Health Aging & Later Life and Personalized Medicine Amsterdam the Netherlands; ^45^ Amsterdam Neuroscience Neurodegeneration and Mood, Anxiety, Psychosis, Stress, and Sleep Amsterdam the Netherlands; ^46^ Julius Center for Health Sciences and Primary Care University Medical Center Utrecht and Utrecht University Utrecht the Netherlands; ^47^ University Medicine Greifswald Institute for Community Medicine SHIP/KEF Greifswald Germany; ^48^ Department of Internal Medicine Section of Gerontology and Geriatrics Leiden University Medical Center Leiden the Netherlands; ^49^ Department of Cardiology Leiden University Medical Center Leiden the Netherlands; ^50^ Division Heart & Lungs Department of Cardiology University Medical Center Utrecht Utrecht University Utrecht the Netherlands; ^51^ Department of Psychiatry and Psychotherapy University Medicine Greifswald Greifswald Germany; ^52^ German Center for Neurodegenerative Diseases (DZNE) Site Rostock/Greifswald Rostock Germany; ^53^ Netherlands Heart Institute Utrecht the Netherlands; ^54^ Einthoven Laboratory for Experimental Vascular Medicine LUMC Leiden the Netherlands; ^55^ Julius Center for Health Sciences and Primary Care University Medical Center Utrecht Utrecht University Utrecht the Netherlands; ^56^ Department of Epidemiology and Population Health Albert Einstein College of Medicine New York New York USA; ^57^ Longitudinal Studies Section Translational Gerontology Branch National Institute on Aging Baltimore Maryland USA; ^58^ Department of Epidemiology University of Washington Seattle Washington USA; ^59^ Department of Neurology University of Washington Seattle Washington USA; ^60^ Institute for Translational Genomics and Population Sciences Department of Pediatrics Lundquist Institute for Biomedical Innovation at Harbor‐UCLA Medical Center Los Angeles California USA; ^61^ BHF Glasgow Cardiovascular Research Centre Faculty of Medicine Glasgow UK; ^62^ Institute of Cardiovascular and Medical Sciences College of Medical Veterinary and Life Sciences University of Glasgow Glasgow UK; ^63^ Laboratory of Epidemiology and Population Sciences—National Institutes of Health Bethesda Maryland USA; ^64^ Department of Neurology University Medicine Greifswald Greifswald Germany; ^65^ Human Genome Sequencing Center Baylor College of Medicine Houston Texas USA; ^66^ Framingham Heart Study Framingham Massachusetts USA; ^67^ Department of Neurology Boston University School of Medicine Boston Massachusetts USA; ^68^ Department of Biostatistics Boston University School of Public Health Boston Massachusetts USA; ^69^ Department of Neurology Beth Israel Deaconess Medical Center Boston Massachusetts USA; ^70^ Department of Epidemiology Human Genetics and Environmental Sciences University of Texas Health Science Center at Houston School of Public Health Houston Texas USA; ^71^ Department of Epidemiology Human Genetics, and Environmental Sciences (EHGES) UTHealth Science Center School of Public Health San Antonio USA; ^72^ DZHK (German Centre for Cardiovascular Research) Partner Site Greifswald Greifswald Germany; ^73^ Interfaculty Institute for Genetics and Functional Genomics University Medicine Greifswald Greifswald Germany; ^74^ Memory Impairment and Neurodegenerative Dementia (MIND) Center and Department of Medicine University of Mississippi Medical Center Jackson Mississippi USA; ^75^ Grupo de Medicina Xenómica CIBERER, CIMUS. Universidade de Santiago de Compostela Santiago de Compostela Spain; ^76^ Fundación Pública Galega de Medicina Xenómica‐IDIS Santiago de Compostela Spain; ^77^ Unidad de Demencias Servicio de Neurología y Neurofisiología. Instituto de Biomedicina de Sevilla (IBiS) Hospital Universitario Virgen del Rocío/CSIC/Universidad de Sevilla Seville Spain; ^78^ Unitat Trastorns Cognitius Hospital Universitari Santa Maria de Lleida Lleida Spain; ^79^ Institut de Recerca Biomedica de Lleida (IRBLLeida) Lleida Spain; ^80^ Hospital Universitario Ramon y Cajal IRYCIS Madrid Spain; ^81^ Unitat de Genètica Molecular Institut de Biomedicina de València‐CSIC Valencia Spain; ^82^ Unidad Mixta de Neurologia Genètica Instituto de Investigación Sanitaria La Fe Valencia Spain; ^83^ Departamento de Especialidades Quirúrgicas Bioquímica e Inmunología. School of Medicine. University of Málaga Málaga Spain; ^84^ Alzheimer Research Center & Memory Clinic Instituto Andaluz de Neurociencia Málaga Spain; ^85^ Unidad Clínica de Enfermedades Infecciosas y Microbiología Hospital Universitario de Valme Seville Spain; ^86^ Departamento de Especialidades Quirúrgicas Bioquímica e Inmunología Facultad de Medicina Universidad de Málaga Málaga Spain; ^87^ CAEBI Centro Andaluz de Estudios Bioinformáticos Sevilla Spain; ^88^ Centro de Biología Molecular Severo Ochoa (UAM‐CSIC) Madrid Spain; ^89^ Instituto de Investigacion Sanitaria “Hospital la Paz” (IdIPaz) Madrid Spain; ^90^ Universidad Autónoma de Madrid Madrid Spain; ^91^ CIEN Foundation/Queen Sofia Foundation Alzheimer Center/Instituto de Salud Carlos III Madrid Spain; ^92^ UFIEC Instituto de Salud Carlos III Madrid Spain; ^93^ CIEN Foundation/Queen Sofia Foundation Alzheimer Center Madrid Spain; ^94^ Unidad de Trastornos del Movimiento Servicio de Neurología y Neurofisiología. Instituto de Biomedicina de Sevilla (IBiS) Hospital Universitario Virgen del Rocío/CSIC/Universidad de Sevilla Seville Spain; ^95^ Departamento de Medicina Facultad de Medicina Universidad de Sevilla Seville Spain; ^96^ Alzheimer's Centre Reina Sofia‐CIEN Foundation Centro de Investigación Biomédica en Red sobre Enfermedades Neurodegenerativas (CIBERNED) Madrid Spain; ^97^ Unit of Neurodegenerative Diseases Department of Neurology Hospital Germans Trias i Pujol Badalona Barcelona Spain; ^98^ Neurodegenerative Diseases Research Laboratory Germans Trias i Pujol Research Laboratory Badalona Barcelona Spain; ^99^ Laboratorio de Genética Hospital Universitario Central de Asturias Oviedo Spain; ^100^ Instituto de Investigación Sanitaria del Principado de Asturias (ISPA) Asturias Spain; ^101^ Complex Genetics of Alzheimer's Disease Group VIB Center for Molecular Neurology, VIB Antwerp Belgium; ^102^ Laboratory of Neurogenetics Institute Born—Bunge Antwerp Belgium; ^103^ Department of Biomedical Sciences University of Antwerp Neurodegenerative Brain Diseases Group Center for Molecular Neurology, VIB Antwerp Belgium; ^104^ Alzheimer Center Amsterdam Neurology Vrije Universiteit Amsterdam Amsterdam UMC Location VUmc Amsterdam the Netherlands; ^105^ Amsterdam Neuroscience Neurodegeneration Amsterdam the Netherlands; ^106^ Section Genomics of Neurodegenerative Diseases and Aging, Human Genetics, Vrije Universiteit Amsterdam, Amsterdam UMC location VUmc Amsterdam the Netherlands; ^107^ German Center for Neurodegenerative Diseases (DZNE) Berlin Germany; ^108^ Charité – Universitätsmedizin Berlin corporate member of Freie Universität Berlin Humboldt‐Universität zu Berlin and Berlin Institute of Health Institute of Psychiatry and Psychotherapy Berlin Germany; ^109^ German Center for Neurodegenerative Diseases (DZNE) Bonn Germany; ^110^ Department for Neurodegenerative Diseases and Geriatric Psychiatry University Hospital Bonn Bonn Germany; ^111^ Institute for Stroke and Dementia Research (ISD) University Hospital LMU Munich Munich Germany; ^112^ German Center for Neurodegenerative Diseases (DZNE) Munich Germany; ^113^ Munich Cluster for Systems Neurology (SyNergy) Munich Germany; ^114^ Martin‐Luther‐University Halle‐Wittenberg University Clinic and Outpatient Clinic for Psychiatry Psychotherapy and Psychosomatics Halle (Saale) Germany; ^115^ Department of Psychiatry Psychosomatics and Psychotherapy Center of Mental Health University Hospital of Würzburg Würzburg Germany; ^116^ German Center for Neurodegenerative Diseases (DZNE) Magdeburg Germany; ^117^ Institute of Cognitive Neurology and Dementia Research (IKND) Otto‐von‐Guericke University Magdeburg Germany; ^118^ Department of Psychiatry and Psychotherapy University Medical Center Goettingen Goettingen Germany; ^119^ German Center for Neurodegenerative Diseases (DZNE) Goettingen Germany; ^120^ Medical Science Department iBiMED Aveiro Portugal; ^121^ Department of Neurodegenerative Diseases and Geriatric Psychiatry University of Bonn Bonn Germany; ^122^ German Center for Neurodegenerative Diseases (DZNE) Bonn Germany; ^123^ Department of Psychiatry and Psychotherapy Technical University of Munich School of Medicine Klinikum rechts der Isar Munich Germany; ^124^ Taub Institute for Research in Alzheimer's Disease and the Aging Brain The Gertrude H. Sergievsky Center Department of Neurology Columbia University New York New York USA; ^125^ 1st Department of Neurology Aiginition Hospital National and Kapodistrian University of Athens Medical School Athens Greece; ^126^ Department of Neurology Hospital Universitario Donostia San Sebastian Spain; ^127^ Neurosciences Area. Instituto Biodonostia San Sebastian Spain; ^128^ Alzheimer's Disease and Other Cognitive Disorders Unit Service of Neurology Hospital Clinic of Barcelona Institut d'Investigacions Biomèdiques August Pi i Sunyer University of Barcelona Barcelona Spain; ^129^ Depatamento de Especialidades Quirúrgicas Bioquímica e Inmunología Facultad de Medicina Universidad de Málaga Málaga Spain; ^130^ Neurology Service Marqués de Valdecilla University Hospital (University of Cantabria and IDIVAL) Santander Spain; ^131^ Department of Neurosciences Faculty of Medicine and Nursery University of the Basque Country San Sebastián Spain; ^132^ Faculty of Medicine University of Lisbon Lisbon Portugal; ^133^ Memory Clinic Department of Neurology Charles University Second Faculty of Medicine and Motol University Hospital Czech Czech Republic; ^134^ International Clinical Research Center St. Anne's University Hospital Brno Brno Czech Republic; ^135^ Unit for Hereditary Dementias Theme Aging Karolinska University Hospital‐Solna Stockholm Sweden; ^136^ Aging Research Center Department of Neurobiology Care Sciences and Society Karolinska Institutet and Stockholm University Stockholm Sweden; ^137^ Department of Public Health and Carins Sciences/Geriatrics Uppsala University Uppsala Sweden; ^138^ Department of Clinical Biochemistry Copenhagen University Hospital – Herlev Gentofte Copenhagen Denmark; ^139^ Department of Clinical Medicine University of Copenhagen Copenhagen Denmark; ^140^ Institute of Clinical Medicine—Neurology University of Eastern Finland Kuopio Finland; ^141^ Division of Clinical Geriatrics Center for Alzheimer Research Care Sciences and Society (NVS); ^142^ Karolinska Institutet Stockholm Sweden; ^143^ Institute of Public Health and Clinical Nutrition University of Eastern Finland Kuopio Finland; ^144^ Neuroepidemiology and Ageing Research Unit School of Public Health Imperial College London London UK; ^145^ Stockholms Sjukhem Research & Development Unit Stockholm Sweden; ^146^ A.I. Virtanen Institute for Molecular Sciences University of Eastern Finland Kuopio Finland; ^147^ Department of Genetics and CNRMAJ University of Rouen Normandy Normandy University Inserm U1245 and CHU Rouen Rouen France; ^148^ University of Lille Inserm 1171 CHU Clinical and Research Memory Research Centre (CMRR) of Distalz Lille France; ^149^ Université de Paris EA 4468, APHP, Hôpital Broca Paris France; ^150^ Department of Child and Adolescent Psychiatry and Psychotherapy University Hospital of Psychiatry Zurich University of Zurich Zurich Switzerland; ^151^ Neuroscience Center Zurich University of Zurich and ETH Zurich Zurich Switzerland; ^152^ Zurich Center for Integrative Human Physiology University of Zurich Zurich Switzerland; ^153^ Neurodegenerative Diseases Unit Fondazione IRCCS Ca’ Granda Ospedale Policlinico Milan Italy; ^154^ Department of Biomedical Surgical and Dental Sciences University of Milan Milan Italy; ^155^ Department of Clinical Sciences and Community Health University of Milan Milan Italy; ^156^ Geriatric Unit Fondazione IRCCS Ca’ Granda Ospedale Maggiore Policlinico Milan Italy; ^157^ Department of Medicine and Surgery Institute of Gerontology and Geriatrics University of Perugia Perugia Italy; ^158^ Department of Biomedical Sciences University of Cagliari Cagliari Italy; ^159^ Neurology, “San Gerardo” Hospital Monza and University of Milano‐Bicocca Milan Bicocca Italy; ^160^ Department of Neuroscience “Rita Levi Montalcini ” University of Turin Turin Italy; ^161^ Department of Hematology and Stem Cell Transplant Vito Fazzi Hospital Lecce Italy; ^162^ Division of Psychological Medicine and Clinical Neuroscience School of Medicine Cardiff University Wales UK; ^163^ Univ. Lille, Inserm, CHU Lille Institut Pasteur de Lille U1167‐RID‐AGE—LabEx DISTALZ facteurs de risque et déterminants moléculaires des maladies liés au vieillissement Lille France; ^164^ Department of Psychiatry and Psychotherapy Faculty of Medicine and University Hospital Cologne University of Cologne Cologne Germany; ^165^ Cluster of Excellence Cellular Stress Responses in Aging‐Associated Diseases (CECAD) University of Cologne Cologne Germany; ^166^ 1st Department of Neurology Medical school Aristotle University of Thessaloniki Makedonia Thessaloniki Greece; ^167^ Department of Clinical Biochemistry Copenhagen University Hospital—Rigshospitalet Copenhagen Denmark; ^168^ Department of Clinical Medicine University of Copenhagen Copenhagen Denmark; ^169^ Department of Biomedical Sciences University of Antwerp Antwerp Belgium; ^170^ Department of Neurology Universitair Ziekenhuis Brussel and NEUR (Neuroprotection & Neuromodulation) Research Group Center for Neurosciences Vrije Universiteit Brussel (VUB) Brussels Belgium; ^171^ Reference Center for Biological Markers of Dementia (BIODEM) Institute Born‐Bunge University of Antwerp Antwerp Belgium; ^172^ Laboratory for Cognitive Neurology Department of Neurosciences University of Leuven Leuven Belgium; ^173^ Neurology Department University Hospitals Leuven Leuven Belgium; ^174^ Department of Public Health and Carins Sciences/Geriatrics Uppsala University Uppsala Sweden; ^175^ Krembil Brain Institute University Health Network Toronto Canada; ^176^ Tanz Centre for Research in Neurodegenerative Diseases Departments of Medicine and Laboratory Medicine & Pathobiology University of Toronto Toronto Canada; ^177^ Fondazione IRCCS Istituto Neurologico Carlo Besta Milan Italy; ^178^ Institute of Biomedicine University of Eastern Finland Kuopio Finland; ^179^ Institute of Psychiatry and Neurology First Department of Neurology Warsaw Poland; ^180^ Raffles Neuroscience Center Raffles Hospital Raffles Singapore; ^181^ St Luke's Hospital Singapore Singapore; ^182^ Department of Health Promotion Sciences Maternal and Infant Care (PROMISE) University of Palermo Palermo Italy; ^183^ Department of Mathematics and Statistics Curtin University Perth Australia; ^184^ Department of Biomedicine Neuroscience and Advanced Diagnostics (BIND) University of Palermo Palermo Italy; ^185^ Dementia and Parkinson's disease Center University Hospital “Paolo Giaccone Palermo Italy; ^186^ Univ Lille Inserm CHU Lille Lille France; ^187^ Lille Neuroscience & Cognition; ^188^ Centre for Healthy Brain Ageing Discipline of Psychiatry & Mental Health School of Clinical Medicine Faculty of Medicine and Health University of New South Wales Sydney Australia; ^189^ NORMENT Centre University of Bergen Bergen Norway; ^190^ Dept of Medical Genetics Oslo University Hospital Oslo Norway; ^191^ Clinical Psychology Service Health Department Fondazione IRCCS Casa Sollievo della Sofferenza San Giovanni Rotondo (FG) Italy; ^192^ Nuffield Department of Clinical Neurosciences Oxford University Oxford UK; ^193^ Unidad de Investigación en Epidemiología y Servicos de Salud Área Envejecimiento Centro Medico Nacional Siglo XXI Instituto Mexicano del Seguro Social. Ciudad de Mexico Coahuila Mexico; ^194^ Translational and Clinical Research Institute Newcastle University; ^195^ Campus for Ageing and Vitality Newcastle upon Tyne NE4 5PL Newcastle UK; ^196^ Faculty of Medicine and Health Technology Tampere University and Department of Clinical Chemistry Fimlab Laboratories Tampere Finland; ^197^ Institute of Neurology Catholic University of the Sacred Heart School of Medicine Largo Agostino Gemelli Rome Italy; ^198^ Neuroscience Research Australia Sydney Australia; ^199^ Department of Clinical Biochemistry Hematology and Immunology Na Homolce Hospital Prague Czech Republic; ^200^ Helsinki University Hospital University of Helsinki Helsinki Finland; ^201^ Neurodegenerative Disease Unit Department of Basic Medicine Neuroscience, and Sense Organs, University of Bari Aldo Moro, Policlinico, Piazza Giulio Cesare 11 Bari Italy; ^202^ Geriatric Unit & Laboratory of Gerontology and Geriatrics Department of Medical Sciences IRCCS “Casa Sollievo della Sofferenza” San Giovanni Rotondo Foggia Italy; ^203^ Unit of Research Methodology and Data Sciences for Population Health National Institute of Gastroenterology Saverio de Bellis Research Hospital Castellana Grotte Bari Italy; ^204^ Translational and Clinical Research Institute Newcastle University Newcastle UK; ^205^ Department of Neurology OHSU Portland USA; ^206^ Department of Research and Innovation Helse Fonna Haugesund Norway; ^207^ Department of Clinical Medicine (K1) University of Bergen Bergen Norway; ^208^ Neuropsychiatric Institute Euroa Centre Prince of Wales Hospital Sydney Australia; ^209^ Institute of Gerontology and Geriatrics Department of Medicine University of Perugia Perugia Italy; ^210^ Department of Clinical Biochemistry Copenhagen University Hospital – Rigshospitalet Copenhagen Denmark & Department of Clinical Medicine Copenhagen Denmark; ^211^ Department of Neurology Oregon Health & Science University Portland USA; ^212^ Department of Research and Innovation Division of Clinical Neuroscience Oslo University Hospital Oslo Norway; ^213^ Institute of Clinical Medicine Faculty of Medicine University of Oslo Oslo Norway; ^214^ K. G. Jebsen Center for Genetic Epidemiology Department of Public Health and Nursing Faculty of Medicine and Health Sciences Norwegian University of Science and Technology (NTNU) Trondheim Norway; ^215^ Department of Internal Medicine Division of Cardiovascular Medicine University of Michigan Ann Arbor Michigan USA; ^216^ Department of Mental Health Faculty of Medicine and Health Sciences Norwegian University of Science and Technology (NTNU) Trondheim Norway; ^217^ Department of Psychiatry Hospital Namsos Nord‐Trøndelag Health Trust Namsos Norway; ^218^ Department of Neuromedicine and Movement Science Faculty of Medicine and Health Sciences Norwegian University of Science and Technology (NTNU) Trondheim Norway; ^219^ Department of Neurology and Clinical Neurophysiology St. Olav's Hospital Trondheim University Hospital Trondheim Norway; ^220^ K G Jebsen Centre for Alzheimer's Disease. Kavli Institutes of Systems Neuroscience Norwegian University of Science and Technology (NTNU) Trondheim Norway; ^221^ Center for Statistical Genetics Department of Biostatistics University of Michigan Ann Arbor Michigan USA; ^222^ Department of Clinical and Molecular Medicine Norwegian University of Science and Technology (NTNU) Trondheim Norway; ^223^ BioCore—Bioinformatics Core Facility Norwegian University of Science and Technology (NTNU) Trondheim Norway; ^224^ Clinic of Laboratory Medicine St. Olavs Hospital Trondheim University Hospital Trondheim Norway; ^225^ Division of Mental Health Care St. Olavs Hospital Trondheim University Hospital Trondheim Norway; ^226^ Department of Neurology and Clinical Neurophysiology St. Olavs Hospital Trondheim University Hospital Trondheim Norway; ^227^ Department of Computational Medicine and Bioinformatics University of Michigan Ann Arbor Michigan USA; ^228^ Analytic and Translational Genetics Unit Massachusetts General Hospital Boston Massachusetts USA; ^229^ Department of Medical Informatics Bordeaux University Hospital Bordeaux France; ^230^ Memory Impairment and Neurodegenerative Dementia (MIND) Center and Department of Medicine University of Mississippi Medical Center Jackson Mississippi USA; ^231^ Department of Neurology School of Medicine University of Pittsburgh Pennsylvania USA; ^232^ Department of Epidemiology & Biostatistics University of California San Francisco California USA; ^233^ Department of Epidemiology Carolina Population Center University of North Carolina at Chapel Hill Chapel Hill North Carolina USA; ^234^ Research and Communication Unit for Musculoskeletal Health (FORMI) Department of Research and Innovation Division of Clinical Neuroscience Oslo University Hospital Oslo Norway; ^235^ Department of Epidemiology Erasmus Medical Center Rotterdam the Netherlands; ^236^ Nuffield Department of Population Health University of Oxford Old Road Campus, Headington Oxford UK; ^237^ Big Data Institute Li Ka Shing Centre for Health Information and Discovery, Old Road Campus, Headington Oxford UK; ^238^ Department of Pharmacology and Pharmacy Centre for Genomic Sciences University of Hong Kong Pok Fu Lam Hong Kong; ^239^ Department of Neurology Erasmus University Medical Centre Rotterdam the Netherlands; ^240^ Department of Clinical Genetics Erasmus MC Rotterdam the Netherlands; ^241^ Department of Radiology and Nuclear Medicine Erasmus MC Rotterdam the Netherlands; ^242^ Department of Psychology Latin American Brain Health (BrainLat) Universidad Adolfo Ibáñez Santiago Chile; ^243^ Norwegian National Advisory Unit on Ageing and Health Vestfold Hospital Trust Tønsberg Norway; ^244^ Department of Geriatric Medicine Oslo University Hospital Oslo Norway; ^245^ HUNT Research Center Department of Public Health and Nursing Faculty of Medicine and Health Sciences Norwegian University of Science and Technology (NTNU) Trondheim Norway; ^246^ Department of Research Innovation and Education St. Olavs Hospital Trondheim University Hospital Trondheim Norway; ^247^ Division of Mental Health and Addiction Oslo University Hospital Oslo Norway; ^248^ NORMENT University of Oslo Oslo Norway; ^249^ Division of Neurogenetics and Molecular Psychiatry Department of Psychiatry and Psychotherapy Faculty of Medicine and University Hospital Cologne University of Cologne Cologne Germany; ^250^ Department of Neurodegenerative diseases and Geriatric Psychiatry University Hospital Bonn Medical Faculty Bonn Germany; ^251^ Department of Psychiatry & Glenn Biggs Institute for Alzheimer's and Neurodegenerative Diseases San Antonio Texas USA; ^252^ Alzheimer Center Amsterdam Department of Neurology Amsterdam Neuroscience Vrije Universiteit Amsterdam Amsterdam UMC Amsterdam the Netherlands; ^253^ CHU de Bordeaux Department of Neurology Institute for Neurodegenerative Diseasese Bordeaux France; ^254^ Institute of Molecular Medicine McGovern Medical School The University of Texas Health Science Center at Houston Houston Texas USA; ^255^ Department of Research Innovation and Education Division of Clinical Neuroscience Oslo University Hospital Oslo Norway; ^256^ Department of Neurology Oslo University Hospital Oslo Norway; ^257^ Translational Health Sciences Bristol Medical School University of Bristol Bristol UK; ^258^ School of Public Health Faculty of Social Welfare and Health Sciences University of Haifa Haifa Israel; ^259^ Framingham Heart Study Framingham Massachusetts USA; ^260^ Department of Neurology University of Texas Health San Antonio San Antonio Texas USA; ^261^ Department of Neurology Boston University School of Medicine Boston Massachusetts USA

**Keywords:** all‐cause dementia, Alzheimer's disease, cross‐ancestry, genome‐wide association study (GWAS), GWAS meta‐analysis, vascular dementia

## Abstract

**INTRODUCTION:**

Dementia is a multifactorial disease with Alzheimer's disease (AD) and vascular dementia (VaD) pathologies making the largest contributions. Yet, most genome‐wide association studies (GWAS) focus on AD.

**METHODS:**

We conducted a GWAS of all‐cause dementia (ACD) and examined the genetic overlap with VaD. Our dataset includes 800,597 individuals, with 46,902 and 8702 cases of ACD and VaD, respectively. Known AD loci for ACD and VaD were replicated. Bioinformatic analyses prioritized genes that are likely functionally relevant and shared with closely related traits and risk factors.

**RESULTS:**

For ACD, novel loci identified were associated with energy transport (*SEMA4D*), neuronal excitability (*ANO3*), amyloid deposition in the brain (*RBFOX1*), and magnetic resonance imaging markers of small vessel disease (SVD; *HBEGF*). Novel VaD loci were associated with hypertension, diabetes, and neuron maintenance (*SPRY2*, *FOXA2*, *AJAP1*, and *PSMA3*).

**DISCUSSION:**

Our study identified genetic risks underlying ACD, demonstrating overlap with neurodegenerative processes, vascular risk factors, and cerebral SVD.

**Highlights:**

- We conducted the largest genome‐wide association study of all‐cause dementia (ACD) and vascular dementia (VaD).
- Known genetic variants associated with AD were replicated for ACD and VaD.
- Functional analyses identified novel loci for ACD and VaD.
- Genetic risks of ACD overlapped with neurodegeneration, vascular risk factors, and cerebral small vessel disease.

## 1 BACKGROUND

Traditionally, Alzheimer's disease (AD) is considered the most common dementia subtype, followed by vascular dementia (VaD). The two conditions are considered clinically distinct. VaD is diagnosed based on the presence of stroke or extensive cerebral vascular disease, with atherosclerosis and arteriolosclerosis considered the underlying pathologies.^1^ However, a wealth of evidence from recent years has emphasized a broad role for brain vascular damage, beyond that of lacunar and larger cerebral infarcts, as a major mechanism for cognitive impairment.^2^ It is now increasingly recognized that a component of vascular pathology is prominent in all major dementias and acts synergistically with amyloid beta (Aβ), tau, and other neurodegenerative pathologies to affect dementia risk.^3^ Moreover, a new hypothetical model of dementia dynamics suggests that damage to brain vasculature is an early process in the dementia continuum that precedes brain atrophy, neurodegeneration, and the emergence of amyloid and tau biomarker abnormalities.^4^ Recent genetic studies using methods that are relatively immune to reverse causation also suggest a putative causal relationship between brain imaging markers of cerebral small vessel disease (SVD) and AD.^5^

Hence there is a strong rationale to examine the “vascular contributions to cognitive impairment and dementia” (VCID), a term that includes a broad range of vascular mechanisms and phenotypes and represents the multifactorial nature of dementia and related disorders as a pathway for reducing dementia burden.^6^ In particular, genetic exploration of VCID may highlight important mechanisms across the wide spectrum of pathologies, including vascular pathways, which, in turn, are considered to be a major and modifiable target for the prevention of dementia, including the Alzheimer's type.^7^

Emerging evidence suggests that VCID is highly heritable.^8^ Mutations in the *NOTCH3* gene known to cause monogenic cerebral SVD and early cognitive impairment also influence later onset polygenic manifestations of VCID by acting through common, less pathogenic variations in the same genes. Other examples are several point mutations in the amyloid precursor protein (APP) gene that lead to cerebral amyloid angiopathy (CAA)^9^ as well as mutations in HtrA Serine Peptidase 1 (*HTRA1*) and Collagen Type IV Alpha 1 Chain (COL4A1) or COL4A2 genes.^10^ Further support for the strong genetic basis of VCID stems from heritability and genome‐wide association studies (GWASs) of cerebral SVD endophenotypes that are closely related to VCID, including ischemic stroke (IS),^11^ and white matter hyperintensities (WMHs).^12^ ^,^ In contrast to the over 70 loci identified as being associated with AD genetic variance, the genetic architecture of “sporadic” VCID is largely unknown. Most genetic studies of VCID have utilized a candidate gene approach, which did not yield consistent and replicable findings.^13^

**RESEARCH IN CONTEXT**

1. **Systematic review**: While findings from genome‐wide association studies (GWASs) of Alzheimer's disease (AD) highlighted multiple genetic risk variants, the genetics of all‐cause dementia (ACD) and vascular dementia (VaD) has been rarely studied. In this meta‐analysis of unpublished GWASs, we utilized data from 21 cohorts and consortia for a total of 46,902 and 8702 cases of ACD and VaD, respectively.
2. **Interpretation**: Known genetic variants for AD were identified as risk factors for ACD and VaD. Downstream bioinformatics revealed novel genetic loci functionally associated with ACD and VaD, including *SEMA4D*, *RBFOX1*, and *SPRY2*.
3. **Future directions**: These results should be validated in additional datasets. Particularly, studies are warranted to explore the genetic variation of ACD and VaD in non‐European individuals.

GWASs of VCID are sparse. In 2012, a GWAS of VaD conducted among the participants of the Rotterdam Study (*N* = 67 cases and 5700 controls) identified a novel locus associated with VaD, located near the androgen receptor on the X chromosome^14^; however, this finding could not be replicated.^15^ More recently, a GWAS of dementia and its clinical endophenotypes was conducted as part of the GR@ACE study.^16^ This study demonstrated the differential biological pathways associated with clinical AD subgroups based on the degree of vascular burden. It identified a variant near *CNTNAP2* associated with probable or possible VCID (*N* = 373). However, this finding did not reach genome‐wide (GW) significance.

The multifactorial nature of VaD and the heterogeneity of the clinicopathological criteria used to define this entity have hampered the identification of genetic polymorphisms underlying VCID. To overcome these limitations, large‐scale studies with sufficient power to detect genetic signals specific to VCID are needed.^17^ In this study, we investigate the genetic predisposition to VCID specifically. Hence, we explored the genetic variability associated with ACD as a broad phenotype, as well as VaD as an extreme phenotype of the dementia continuum characterized by increased vascular burden. Our findings were then analyzed in light of the knowledge already gained from previous large‐scale GW and sequencing studies on the genetic determinants of AD, stroke, and additional phenotypes along the VCID spectrum.^10^

## 2 METHODS

### 2.1 Study population

A total of 800,597 participants from 21 cohorts and consortia contributed to 46,902 and 8702 cases of ACD and VaD, respectively. The overall sample included individuals from four different ethnicities (European, African, Asian, and Hispanic) from North America, Europe, and Asia. The mean age ranged between 54 and 80 years, with 54% to 68% females. The summary demographics are described in Table 1 (also detailed in Table S1 of Supplementary File 1). Each study obtained written informed consent from participants or, for those with substantial cognitive impairment, from a caregiver, legal guardian, or other proxy. Study protocols for all cohorts were reviewed and approved by the appropriate institutional review boards.

**TABLE 1:** Demographics: Data from 17 CHARGE cohorts were included in our meta‐analysis, as were the UKBB, ADGC, and EADB for the replication of our VaD results in European ancestry.

Study	N/Control	ACD	VaD	Percentage VaD	Age (mean)	Sex, % (percentage female)
**European ancestry**						
3C	6475	808	162	20.1	74.2	61.0
AGES	5656	1501	118	7.9	76.1	61.0
ARIC	3145	165	36	21.8	75.5	60.0
ASPREE	12,480	319	NA	NA	75.0	55.0
CHS	2169	508	156	30.7	74.9	61.5
FVG	804	73	NA	NA	58.2	58.3
FHS	4175	679	167	24.5	54.6	54.3
GRACE	12,599	7516	1953	26.0	78.8	68.2
GREAT‐AGE	1504	138	7	5.1	73.7	50.3
HUNT	69,633	3982	681	17.1	67.7	57.4
MEMENTO	2050	263	36	13.7		
MYHAT	865	50	NA	NA	83.7	59.5
ROSMAP	1335	626	NA	NA	79.8	69.7
RS (1,2,3)	11,390	1715	178	10.4	63.6	56.8
ADGC‐NAJ‐2011	15,675	8309	NA	NA	75.4	59.5
UKBB	314,278	17,008	332	NA	66.1	63.1
**Total European**	**466,606**	**44,009**	**3892**			
**African ancestry**						
ARIC	905	101	31	30.7	75.5	60.0
CHS	514	194	65	33.5	74.9	61.5
ADGC‐Reitz (2013)	5896	1968	NA	NA	80.5	63.9
**Total African**	**7315**	**2263**	**96**			
**Asian ancestry**						
HKOS	2373	349	66	18.9	60.1	67.9
Harmonization	385	153	49	32.0	73.6	55.0
**Total Asian**	**2758**	**502**	**115**			
**Hispanic ancestry**						
SALSA	1271	128	35	27.3	68.9	58.6
**Total Stage 1** for ACD and VaD	477,950	46,902	4138			
**Replication of VaD results in EADB**						
EADB	275,745	NA	4,564			
**Total**	**753,695**	**46,902**	**8702**			

*Note*: Overall, 800,597 individuals were included in this study, accounting for 46,902 and 8702 cases of ACD and VaD, respectively. For UKBB, we used the proxy‐AD (familial AD) for ACD analysis and assessed VaD cases using ICD10 codes (see Methods). We also used ADGC‐NAJ‐201118 and ADGC‐Reitz‐201319 for ACD in European and African ancestry, respectively, to avoid overlap with CHARGE samples. We subsequently replicated our VaD results in EADB.

Abbreviations: ACD, all‐cause dementia; ADGC, Alzheimer's Disease Genetics Consortium; EADB, the European Alzheimer Disease Biobank; UKBB, the UK Biobank.

### 2.2 Phenotype definition

The primary study outcomes are ACD and VaD, measured by each participating cohort as described in Supplementary File 2. Briefly, to diagnose ACD and VaD, a neurological evaluation and diagnosis based on validated criteria were required. These criteria, as shown in Table S1 of Supplementary File 1, include the use of International Statistical Classification of Diseases and Related Health Problems (ICD) codes in most cohorts, as well as additional criteria such as the Diagnostic and Statistical Manual of Mental Disorders, Third to Fifth editions (DSM‐III to V), National Institute of Neurological Disorders and Stroke‐Alzheimer's Disease and Related Disorders Association, National Institute of Neurological Disorders and Stroke‐the Association Internationale pour la Recherche et l'Enseignement en Neurosciences, and dementia by proxy for United Kingdom Biobank (UKBB). Additionally, VaD cases were included in ACD, and the proportion of ACD classified as VaD is reported in Table S1 of Supplementary File 1. Moreover, to increase the sensitivity, cohorts were asked to run separate association analyses for (a) incident ACD, (b) prevalent ACD, (c) incident VaD, (d) prevalent VaD, (e) incident probable and definite VaD, and (f) prevalent probable whenever possible. To address the overlap with AD, we included all VCID (including persons with possible VCID) and separately analyzed only cases of “pure” (probable and autopsy‐proven definite) VCID and requested that all cohorts provide the most accurate, detailed description of their diagnostic algorithm. Although VaD in UKBB was defined based on ICD‐10 codes, we used the family history of dementia GWAS (“imputed dementia”) recently published by Marioni et al.^18^ for ACD. Imputed dementia was defined as individuals at least 65 years old reporting a history of dementia in one or both parents. As explained in Ghosh et al.,^19^ the effect sizes and standard errors of the imputed dementia GWASs were doubled to analytically correct for the use of proxy phenotypes.

### 2.3 Genotyping and imputation

Genotyping was performed using cohort‐specific genotyping arrays as described in Supplementary File 2. Genetic variants were imputed using 1000 Genomes Project (1KG), the Haplotype Reference Consortium (HRC),^20^ and the National Heart, Lung, and Blood Institute Trans‐Omics for Precision Medicine (TOPMed). UKBB imputed the genotypes to HRC, 1KG, and UK10K. Details on study‐specific quality control (QC) filters and software used for phasing and imputation are provided as supplementary materials (Supplementary File 2). Briefly, rare variants (minor allele frequency [MAF] < 1%) and poorly imputed variants (imputation quality, Rsq < 0.3) were excluded, as were variants mapping to sex chromosomes or mitochondria. Samples with poor genotyping call rate (<95%) and Hardy–Weinberg *p* values < 1 × 10^−6^ were removed. All genetic positions are reported in genome build 37 (GRCh37, hg19). Moreover, we used HRC version 1.2 as the main reference panel, and only variants in this panel were subsequently used in the association analyses. Additional details on the genotyping and imputation methods and QC are provided in Supplementary File 2.

### 2.4 Genome‐wide association analyses, QC, and meta‐analysis

#### 2.4.1 Study‐level association analyses

We conducted study and ethnicity‐specific association analyses adjusting for age, sex, sites, and population structure to test the association of each variant with VaD and ACD. Cohorts were asked to run logistic regression and Cox proportional hazard models for prevalent and incident VaD/ACD, respectively, assuming additive allelic effects and imputed dosages. The UKBB association analyses were performed with linear mixed models (LMMs) using the BOLT‐LMM software.^21^ BOLT‐LMM has the advantage over other methods in that it accounts for cryptic relatedness and population structure and, thus, allows the inclusion of related individuals in models, which increases the overall sample size. Details on the methods and software used for study‐level association analyses are provided in Supplementary File 2.

#### 2.4.2 QC of study‐level summary statistics

We performed a stringent QC check of the summary statistics from each cohort using EasyQC.^22^ We mapped each variant from the non‐European ancestry (EA) cohort to the appropriate 1KG project phase 3 reference panel and all EA to HRC (details in Table S1). Then the following steps were performed to ensure proper QC of each file before the meta‐analysis: (a) remove all structural variants and INDELs; (b) filter out variants with missing or unusual values (*p* value < 0 or > 1, effect size > 10, effect allele frequency < 0 or > 1, imputation quality < 0 or > 1); (c) filter out variants with effective allele count (EAC, 2 × minor allele frequency × *N* × imputation quality) < 10; (d) filter out variants with low imputation quality (eg, INFO scores reported by the imputation software); (e) filter out variants with MAF < 1%; (f) align variants to the main reference panel (HRC for EA, and ethnicity‐specific 1KG for others); remove variants with absolute difference between its allele frequencies in the cohort and reference panel greater than 0.2. All variants were assigned a unique identifier as a combination of the chromosome, position, reference, and alternative alleles separated by semi‐colons (CHR:POS:REF:ALT) to avoid issues with chromosomal positions mapping to multiple marker IDs. The foregoing steps were repeated until satisfactory results were obtained after visual inspection of the different diagnostic QC plots (AF, P‐Z, Q‐Q, and SE plots) generated by EasyQC as explained in Winkler et al.^22^

#### 2.4.3 Meta‐analysis of GWAS results

##### Ancestry‐specific meta‐analysis

The meta‐analyses were conducted using the fixed‐effect inverse variance‐weighted method implemented in METAL.^23^ Post‐analysis results were filtered to retain only variants present in more than 40% of the overall cohorts and the effective sample size greater than 40% of the study sample size. We evaluated the heterogeneity across cohorts using the *I* ^2^ statistic provided by METAL, which represents the percentage of variation across studies that is due to heterogeneity rather than chance. We used the standard *p* value thresholds for GW significance, *p* < 5e‐8, and suggestive *p* < 1e‐6. Since there was no evidence of genomic inflation in the cohort summary statistics (lambda 0.98 to 1.06), no genomic control was applied during the meta‐analysis. Genomic loci were defined as the region ±500 kb around the single nucleotide polymorphism (SNP) with the lowest *p* value, considered as the index SNP. We assessed the heterogeneity across studies using the *I* ^2^ statistics of METAL (HetPVal output), which represents the percentage of variation across cohorts that is due to genetic heterogeneity rather than chance. Except for the *APOE* region (defined as SNPs located on chromosome 19 between positions 45,000,000 and 45,800,000 base pairs according to GRCh37 [hg19]), for which the HetPVal was >1e‐8, significant SNPs were selected with HetPVal > 0.01. We conducted ancestry‐specific meta‐analyses of VaD and ACD for EA, African ancestry (AA), Asian ancestry (SA), and Hispanic ancestry (HA). In addition, we used linkage disequilibrium (LD) score regression to quantify the contribution of true polygenicity and biases such as cryptic relatedness and population stratification of the meta‐analysis results.

##### Cross‐ancestry meta‐analysis

We performed cross‐ancestry meta‐analyses to assess whether the increase in sample size could lead to adequate power to identify additional GW significant loci associated with ACD and VaD. To this end, we used Meta‐Regression of Multi‐Ancestry Genetic Association (MR‐MEGA) software, which has proven more efficient than others when dealing with genetic heterogeneity.^24^ MR‐MEGA uses a matrix of mean pairwise allele frequency differences to quantify the genetic similarity between studies and estimate the effect of each SNP after adjusting for ancestry principal components. We applied study‐specific filters, as previously described in the QC section, with EAC > 20, for studies with small sample sizes to reduce the amount of noise in the results‐driven rare SNPs in small cohorts. We fitted three principal components, as suggested by MR‐MEGA authors, which proved sufficient to separate the cohorts into self‐reported ancestry groups (Figure S1). As in the ancestry‐specific meta‐analysis, we retained only SNPs that were present in over 40% of cohorts, with >40% total sample size. GW significant SNPs had *p* < 5e‐8 and showed evidence of allelic heterogeneity across populations (MR‐MEGA P‐Het > 1e‐5).

### 2.5 Shared genetic susceptibility with complex disease traits

A gene‐based association test was conducted using MAGMA,^25^ with *p* < 2.8e−6 as a genome‐wide significance threshold. Gene regions with SNPs not reaching GW significance for ACD or VaD in the primary GWAS analysis and additionally not in LD (*r* ^2^ < 0.10) with the lead SNP were considered novel.

We first explored the association of lead risk variants with related vascular, neurological traits and metabolic traits, excluding the APOE region. For each related trait, association statistics of SNPs falling in a window of ±250 kb around each lead SNP were queried,^26^ and SNPs satisfying the GW significance threshold in the original study were retained. Leveraging the polygenicity of ACD (mean chi‐squared = 1.1) and VaD (mean chi^‐^squared = 1.06), we systematically explored the genetic overlap of ACD and VaD (in European‐only analysis) with (i) neurological and neurodegenerative traits (any stroke [AS], IS, small vessel stroke [SVS], large artery stroke [LAS], cardioembolic stroke [CES], general cognitive function [GCF], and Alzheimer‐type dementia [AD]); (ii) common magnetic resonance imaging (MRI) marker of cerebral SVD (WMHs)^5^; and (iii) vascular risk factors (systolic blood pressure [SBP], diastolic blood pressure [DBP], pulse pressure [PP], high‐density lipoprotein [HDL], low‐density lipoprotein [LDL]).^27^ We acquired summary statistics of the largest European‐only GWAS for these traits.

Using LD score regression (LDSR) analysis,^28^ genetic correlation estimates between ACD/VaD and the aforementioned complex traits were obtained. A similar and potentially powerful approach called genetic covariance analyzer (GNOVA)^29^ was additionally used to study the shared genetic covariance across the genome between a given pair of complex traits. LDSR and GNOVA compute genetic correlation and covariance, respectively, while adjusting for potential sample overlap and accounting for the LD of genetic variants. Though LDSR and GNOVA are substantially similar, differences in the minor allele frequency thresholds may influence genetic correlation estimates and significance to some extent. A *p* value < 8.3e‐3 correcting for six independent phenotypes was considered significant. Additionally, for the traits with significant genetic overlap, we performed causal inference analysis in the Mendelian randomization (MR) framework with ACD/VaD as the outcome. Using the MR‐LAP method,^30^ we addressed potential bias in the causal effects due to sample overlap between the exposure and the outcome variables. Briefly, MR‐LAP utilizes the LDSR intercept estimates – a measure of the degree of sample overlap, polygenic architecture, and the heritability of the genetic instruments of the exposures – to account for the sample overlap bias and other biases (weak instrument and winner's curse bias) that push the causal estimates toward the null.

Since GW correlation estimates may miss significant correlations at the regional level (balancing effect),^31^ a Bayesian pairwise GWAS approach (GWAS‐PW) was applied.^32^ GWAS‐PW identifies trait pairs with high posterior probability of association (PPA) with a shared genetic variant (Model 3, PPA3 ≥ 0.90). To ensure that PPA3 is unbiased by sample overlap, fgwas version 0.3.6 was run on each pair of traits, and the correlation estimated from regions with null association evidence (PPA < 0.20) was used as a correction factor.^32^ We then calculated Spearman's rank correlation for regions showing PPA3 > 0.90, approximating the direction of effect.

Finally, using a Bayesian method – *ashR* ^33^ – we studied the effect‐size distribution for ACD and VaD and related risk factors. Briefly, *ashr* tests the probability of non‐zero effect conferred by SNPs as a function of LD score, measuring the true effect size that is not zero and the underlying polygenic background. Using MTAG,^34^ traits falling in similar polygenic profile to ACD or VaD are jointly analyzed in a bivariate scheme leveraging the pairwise trait genetic correlation to boost power to discover new loci. The significance threshold in the MTAG analysis is determined based on the number of traits sharing a similar polygenic profile and was additionally restricted to SNPs that also had nominal significance (*p* < 0.05) for each phenotype separately in the pre‐existing univariate GWAS.

### 2.6 Transcriptome‐wide association study and colocalization

We performed transcriptome‐wide association studies (TWASs) using the association statistics from the ACD and VaD (European‐only) and weights from 22 publicly available gene expression reference panels from blood (Netherlands Twin Registry [NTR], Young Finns Study [YFS]), arterial (genotype‐tissue expression [GTEx]), brain (GTEx, CommonMind Consortium [CMC]), and peripheral nerve tissues (GTEx). For each gene in the reference panel, precomputed SNP‐expression weights in the 1‐Mb window were obtained, including the highly tissue‐specific splicing quantitative trait loci (sQTLs) information on gene isoforms in the dorsolateral prefrontal cortex (DLPFC) derived from the CMC. TWAS‐Fusion^35^ was used to estimate the TWAS *z*‐score (association statistic between predicted expression and ACD or VaD), derived from the SNP‐expression weights, SNP‐trait effect estimates, and the SNP correlation matrix. Transcriptome‐wide (TW) significant genes (eGenes) and the corresponding QTLs (expression QTLs [eQTLs]) were determined using Bonferroni correction in each reference panel, based on the average number of features (4235 genes) tested across all the reference panels.^35^ eGene regions with eQTLs not reaching GW significance in association with ACD or VaD and not in LD (*r* ^2 ^< 0.01) with the lead SNP for GW significant risk loci were considered novel. Finally, a colocalization analysis (COLOC)^36^ was carried out at each locus to estimate the posterior probability of a shared causal variant (PP4 ≥ 0.75) between the gene expression and trait association, using a prior probability of 1.1 × 10^−5^. Furthermore, functional validation of the eGenes was performed by testing for positional overlap of the best eQTLs from TWAS with enhancer (H3K4me1, H3K27ac) and/or promoter (H3K4me3/H3K9ac) elements across a broad category of relevant tissue types (blood [BLD], brain/neurological [BRN]) using Haploreg version 4.1.^37^

### 2.7 Identification of independent case–case loci with case–case GWAS

Leveraging summary statistics from our GWAS of ACD and VaD, as well as from publicly available existing GWASs of AD^38^ and stroke,^39^ we examined genetic uniqueness between these highly correlated though distinct disorders using case–case GWAS (CC‐GWAS), a method that tests for differences in allele frequency between cases of two disorders without individual‐level data.^40^ By allowing for sample overlap between the two case‐control GWASs, CC‐GWAS can increase the power to detect signals otherwise missed in case‐control GWASs. We used a LD threshold of 0.2 (*r* ^2^ < 0.2) to distinguish CC‐GWAS‐specific loci from genome‐wide significant variants identified in the input case‐control GWAS.

## 3 RESULTS

Our analysis included 800,597 individuals comprising 46,902 and 8702 cases of ACD and VaD, respectively. They were recruited from the 19 Cohorts for Heart and Aging Research in Genomic Epidemiology (CHARGE) cohorts, the Alzheimer's Disease Genetics Consortium (ADGC), the European Alzheimer Disease Biobank (EADB), and the UK Biobank (UKBB), encompassing four different reported ancestries: European (98.5%), African (1.0%), Asian (0.4%), and Hispanics/Latino (0.1%). Association analyses were performed in each cohort following a predefined analysis plan, using logistic regression and Cox proportional hazards models for prevalent and incident cases, respectively. We performed study‐specific QC of the summary statistics data, followed by ancestry‐specific meta‐analyses and cross‐ancestry meta‐analyses of ACD and VaD, as described in the Methods section. For each cohort, a description, association analysis method, QC parameters, and cutoffs are provided in Supplementary File 2.

### 3.1 Meta‐analyses of ACD and VaD GWAS in European ancestry populations replicated known AD loci

We conducted fixed‐effects inverse variance‐weighted meta‐analyses of the 14 European ancestry cohorts (*N* = 466,606, *N*_ACD_ = 44,009, *N*_VaD_ = 3892) from the CHARGE consortium, ADGC, and the UKBB. Furthermore, we replicated significant and suggestive signals from our VaD GWAS in the EADB consortium VCID data (*N* = 275,745, *N*_VaD_ = 4564). The complete list of cohorts included in this study is provided in Table S1.

**FIGURE 1:**
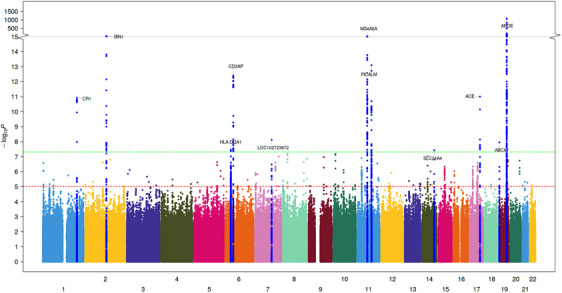
Manhattan plot of ACD GWAS. In addition to variants in APOE region, we identified five new genetic loci associated with VaD. Blue and red lines correspond to *p* value of 5e^−7^ and 5e^−8^ for genome‐wide suggestive and significant SNPs, respectively. Manhattan plots for the cross‐ancestry meta‐analysis. Each dot represents a SNP, the *x*‐axis shows the chromosomes where each SNP is located, and the *y*‐axis shows −log10 *p* value of the association of each SNP with ACD in the cross‐ancestry meta‐analysis. The red horizontal line shows the genome‐wide significant threshold (*p* value = 5e‐8; −log10 *p* value = 7.30). The nearest gene to the most significant SNP in each locus has been labeled.

**FIGURE 2:**
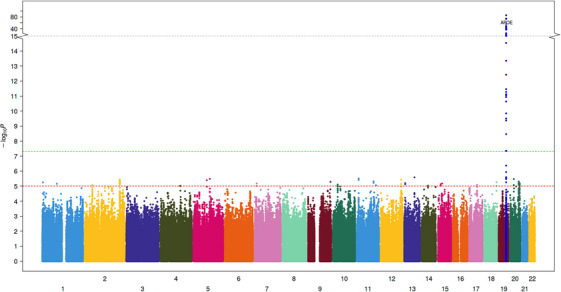
Manhattan plot of VaD GWAS. In addition to variants in the APOE region, we identified five new genetic loci associated with VaD. Blue and red lines correspond to a *p* value of 5e^−7^ and 5e^−8^ for genome‐wide suggestive and significant SNPs, respectively. Manhattan plots for cross‐ancestry meta‐analysis. Each dot represents a SNP, the *x*‐axis shows the chromosomes where each SNP is located, and the *y*‐axis shows the −log10 *p* value of the association of each SNP with VaD in the cross‐ancestry meta‐analysis. The red horizontal line shows the genome‐wide significant threshold (*p* value = 5e‐8; −log10 *p* value = 7.30). The gene closest to the most significant SNP in each locus has been labeled.

**FIGURE 3:**
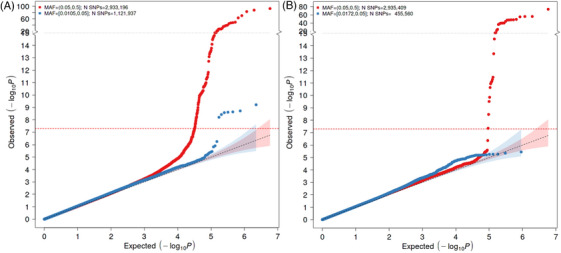
Q‐Q plots of ACD (left) and VaD (right) GWASs. The expected *p* values (*x*‐axis) are plotted against the observed *p* values (*y*‐axis). The units of the axes are the −log10 of the *p* value. The red and blue curves represent the plots with MAF ≥ 0.05 and 0.01, respectively. The diagonal line of the null hypothesis and its 95% confidence interval are plotted in gray based on the *p* values without the previously reported SNPs. The red dotted line represents the cutoff for genome‐wide significance. MAF, minor allele frequency.

A total of 11,596,629 and 9,878,961 SNPs passed the study‐level QC criteria and were tested for association with ACD and VaD, respectively. After post‐meta‐analysis QC, we identified 10 GW significant loci associated with ACD (GWS, *p* < 5 × 10^−8^), all of which had been previously associated with AD (Table 2, extended results in Table S2). Significant loci associated with ACD included signals in or around known AD genes such as *APOE*, *BIN1*, *MS4A6A*, *PICALM*, *CR1*, *CD2AP*, *ABCA7*, *PILRB*, *SLC24A4*, and *ACE*. For VaD, only one variant located near the *APOE* gene reached GW significance (Table 3, extended results in Table S3). The genomic inflation coefficients (lambda) were 1.05 and 1.07 for ACD and VaD, respectively. The lambda intercept computed with the LDSC software was 1.01 for both analyses, suggesting no systematic inflations of association statistics. The Manhattan and quantile‐quantile (QQ) plots for both analyses are provided in Figures 1, 2, 3 (Forest and locusZoom plots for prominent signals are provided in Figures S2 to S65).

**TABLE 2:** Genome‐wide significant (*p* < 5 × 10^−8^) and suggestive (*p* < 1 × 10^−6^) variants associated with all‐cause dementia in European populations.

rsID	Nearest gene	CHR	POS	EA/NEA	EAF	BETA	*p* value	HetISq	HetChiSq	HetPVal
rs429358	APOE	19q13.32	45411941	C/T	0.1515	1.1728	6.87E‐305	98.3	1104.478	2.534E‐222
rs4663105	BIN1	2q14.3	127891427	C/A	0.4151	0.156	5.856E‐34	63.4	51.892	0.00006868
rs12453	MS4A6A	11q12.2	59945745	C/T	0.3998	−0.0933	1.116E‐16	13.2	23.037	0.287
rs10792832	PICALM	11q14.2	85867875	A/G	0.3585	−0.0857	8.234E‐14	61.8	52.41	0.0000992
rs10948367	CD2AP	6p12.3	47585615	G/A	0.2712	0.0886	4.041E‐13	4.2	20.88	0.4042
rs4295	ACE	17q23.3	61556298	C/G	0.3897	−0.0784	1.014E‐11	10.8	22.43	0.3176
rs4844610	CR1	1q32.2	207802552	A/C	0.1846	0.0961	1.245E‐11	71.4	66.468	3.519E‐07
rs2906644	PILRB	7q22.1	99956290	G/C	0.1294	−0.1016	7.627E‐09	0	8.219	0.9616
rs3764650	ABCA7	19p13.3	1046520	G/T	0.0957	0.1141	1.125E‐08	35	24.604	0.07712
rs9323877	SLC24A4	14q32.12	92934269	G/A	0.2521	0.0699	3.804E‐08	0	19.881	0.4654
rs1532278	CLU	8p21.1	27466315	T/C	0.3819	−0.0629	6.262E‐08	66.2	59.185	9.516E‐06
rs7912495	USP6NL	10p14	11718713	G/A	0.4612	0.0684	6.774E‐08	0	15.343	0.7006
rs17125924	FERMT2	14q22.1	53391680	G/A	0.0928	0.1009	9.38E‐08	2.6	19.508	0.4247
rs11767557	EPHA1	7q34	143109139	C/T	0.1998	−0.0742	9.721E‐08	31.2	29.052	0.08675
rs1854554	SEMA4D	9q22.2	92155871	A/G	0.409	0.0598	1.096E‐07	4	20.83	0.4072
rs7118826	ANO3	11p14.2	26195535	G/C	0.4585	0.0686	1.238E‐07	18.7	23.368	0.2215
rs79832570	SPATC1	8q24.3	145097720	C/T	0.086	0.1433	1.385E‐07	37.3	19.147	0.08504
rs13010870	RBM43	2q23.3	151765163	C/T	0.2162	−0.0732	1.649E‐07	0	18.774	0.5366
rs1354106	CD33	19q13.41	51737991	G/T	0.3441	−0.0614	1.673E‐07	6.4	21.373	0.3755
rs6014724	CASS4	20q13.2	54998544	G/A	0.0874	−0.1071	1.863E‐07	0	18.603	0.4825
rs897150	TRIB1	8q24.13	126576702	A/G	0.3016	−0.0634	2.33E‐07	4	20.842	0.4065
rs11168036	HBEGF	5q31.3	139707439	T/G	0.4933	0.0572	2.336E‐07	0	18.21	0.5735
rs17269688	NCK2	2q12.2	106469267	G/A	0.025	−0.1937	2.389E‐07	41.8	27.478	0.03647
rs677649	RNU6‐11P	7	123439244	T/G	0.1756	0.0851	2.505E‐07	9.9	19.987	0.3336
rs8081878	ZNF652	17q21.32	47436812	T/A	0.4634	0.057	2.609E‐07	9.9	22.19	0.3303
rs4654450	RP1‐37J18.2	1	4667378	G/A	0.3314	−0.0697	2.684E‐07	16.2	22.679	0.2518
rs11218343	SORL1	11q24.1	121435587	C/T	0.0364	−0.1658	2.909E‐07	20.7	21.432	0.2076
rs2297508	SREBF1	17p11.2	17715317	C/G	0.3659	−0.0585	4.604E‐07	31.9	29.367	0.08078
rs442495	ADAM10	15q21.3	59022615	C/T	0.3245	−0.0599	4.722E‐07	41	33.895	0.02684
rs13316744	AC091493.2	3	16742711	C/G	0.4544	−0.0549	7.609E‐07	0	15.72	0.7338
rs7068231	ANK3	10q21.2	61784928	T/G	0.4005	−0.0579	7.833E‐07	11.1	22.503	0.3139
rs834398	GABRB2	5q34	160528276	G/A	0.1971	0.0793	8.951E‐07	0	11.885	0.8905
rs62013908	RBFOX1	16p13.3	5991314	G/C	0.2547	0.0724	9.422E‐07	3.9	19.767	0.4087

*Note*: Genome‐wide significant variants are highlighted in orange.

**TABLE 3:** Genome‐wide significant (*p* < 5 × 10^−8^) and suggestive (*p* < 1 × 10^−6^) variants associated with vascular dementia in European populations.

rsID	Nearest gene	CHR	POS	EA/NEA	EAF	BETA CHARGE	*p* value CHARGE	*p* value EADB	*p* value COMBINED	Direction
rs429358	APOE	19q13.32	45411941	C/T	0.1549	0.8794	2.67E‐86	5.66E‐113	2.9E‐196	++
rs11911	SPRY2	13q31.1	80910851	C/A	0.3756	−0.1466	2.60E‐06	0.0653	0.0000335	−
rs7101996	GALNT18	11	11259298	T/C	0.4198	−0.1362	3.06E‐06	0.48	0.00452	−+
rs2845990	LINC02113	5	98907502	C/T	0.352	−0.1383	3.24E‐06	0.872	0.00131	−+
rs117904289	FOXA2	20p11.21	22782154	G/A	0.0858	0.2516	3.35E‐06	0.987	0.00112	+−
rs838941	SCARB1	12q24.31	125183316	A/G	0.4261	0.1344	3.56E‐06	0.33	0.0000654	++
rs17418160	ERBB4	2q34	213119022	C/T	0.0396	0.3341	3.58E‐06	0.882	0.00171	+−
rs77542509	TRPC6	11q22.1	101415824	C/T	0.058	−0.3038	4.77E‐06	0.137	0.0000213	−
rs6127311	DOK5	20q13.2	53501017	C/T	0.0532	−0.3141	4.83E‐06	0.294	0.0144	+−
rs35945091	LCN1P2	9	136185411	C/T	0.2227	0.164	5.15E‐06	0.855	0.00176	+−
rs17059857	ZNF236	18q23	74469493	C/T	0.0403	0.358	5.41E‐06	0.417	0.00934	+−
rs143750890	AJAP1	1p36.32	4602505	C/T	0.0273	0.4283	5.56E‐06	0.163	0.0000334	++
rs9510987	SPATA13	13	24575243	G/T	0.2525	0.1466	6.28E‐06	0.291	0.0000744	−
rs55709546	PHACTR3	20q13.32	58261107	C/A	0.0484	0.3044	6.53E‐06	0.645	0.000496	++
rs12667855	TMEM106B	7p21.3	12124166	T/G	0.0981	0.225	6.59E‐06	0.542	0.000299	++
rs281219	SEMA6D	15q21.1	47711652	A/G	0.1954	0.1641	6.65E‐06	0.316	0.0129	+−
rs138352554	GBP1	1p22.2	89517105	G/A	0.0359	0.3761	6.95E‐06	0.573	0.000406	++
rs16967121	RASGRP1	15q14	38923007	G/A	0.0658	0.2605	7.31E‐06	0.916	0.00126	++
rs11007123	WAC	10p12.1	28763005	C/T	0.2804	0.1395	7.70E‐06	0.292	0.0139	+−
rs4794009	GIP	17q21.32	47051955	A/G	0.4412	0.1274	8.17E‐06	0.797	0.000746	++
rs35448830	PRKCE	2p21	46080762	C/T	0.0368	0.3346	8.48E‐06	0.631	0.000579	++
rs2233754	PSMA3	14q23.1	58755574	C/A	0.07	0.2575	9.39E‐06	0.601	0.00604	+−

*Note*: The meta‐analysis includes 11 cohorts from the CHARGE consortium and the UK Biobank (UKBB) GWAS. Direction denotes the direction of association in CHARGE and EADB. The genome‐wide significant variant is highlighted in orange.

Abbreviations: ADGC, Alzheimer's Disease Genetics Consortium; CHARGE, Cohorts for Heart and Aging Research in Genomic Epidemiology; EADB, European Alzheimer Disease Biobank; UKBB, UK Biobank.

For the VaD trait, we selected all variants with a *p* value less than 1 × 10^−5^ and meta‐analyzed with EADB summary results using a weighted sum of *z*‐scores approach. Only one variant near the *APOE* gene was statistically significant and had the same direction of effect in both studies (Table 3).

The meta‐analyses of ACD and VaD GWAS in African, Asian, and Hispanic/Latino ancestries did not provide new GW significant variants.

We replicated our VaD signals using EADB data (Table 3). Overall, we replicated an association within the *APOE* region. The suggestive variant near *SPRY2* also has the lowest *p* value in the EADB GWAS with the same direction of effect in both studies.

### 3.2 Cross‐ancestry meta‐analysis of ACD and VaD GWAS

Next, we performed a cross‐ancestry meta‐analysis using MR‐MEGA, first to assess whether the increased sample size could lead to the identification of additional loci associated with ACD and VaD and to identify loci that are relevant in other ancestries. Most of the cross‐ancestry meta‐analyses included individuals of European ancestry and smaller samples from African, Asian, and Hispanic/Latino ancestries. The total number of variants included was 17,054,226 and 11,595,061 for ACD and VaD, respectively. The Manhattan plots of the SNP‐wide meta‐analyses for both traits are provided in Figures S66 and S67. Significant and suggestive signals for ACD and VaD are presented in Tables 4 and 5, and the extended results are in Tables S4 and S5. We identified novel signals reaching GW significance at 20q11.21 (*CHD6*, an oxidative DNA damage response factor previously associated with neurological phenotype),^41^ 2q14.1 (*DAW1*, involved in cerebrospinal fluid circulation and cilia motility during development),^42^ and 15q15.1 (*PWRN2*, previously associated with tauopathy and Prader–Willi syndrome)^43^ for ACD and 17q21.1 (*MARCHF10*) for VaD.

**TABLE 4:** Genome‐wide significant (*p* < 5 × 10^−8^) variants associated with all‐cause dementia in cross‐ancestry meta‐analysis.

rsID	Nearest gene	Chr	Pos	EA/NEA	*P* value	MAF	Beta	SE
rs10402524	BCAM	19p11	45329344	T/C	1.21E‐17	0.2336	−0.168	0.045
rs744373	BIN1	2q14.3	127894615	A/G	1.90E‐17	0.358	−0.139	0.031
rs2278867	MS4A6A	11q13.1	59943109	A/T	1.72E‐15	0.2897	0.113	0.020
rs10792832	PICALM	11q13.1	85867875	A/G	3.77E‐12	0.3135	−0.074	0.036
rs10948367	CD2AP	6q14.3	47585615	A/G	1.67E‐11	0.2328	−0.042	0.017
rs1408077	CR1	1q11.1	207804141	A/C	4.75E‐10	0.1412	0.088	0.055
rs4295	ACE	17q21.1	61556298	C/G	1.60E‐09	0.3666	−0.066	0.018
rs2208524	CHD6	20q11.21	40423299	T/C	1.66E‐09	0.1268	−0.103	0.027
rs11691153	DAW1	2q14.1	228780072	T/C	1.83E‐09	0.1536	0.099	0.025
rs6853262	LPHN3	4q22.1	61221892	C/T	6.22E‐09	0.06989	0.208	0.112
rs2677386	PWRN2	15q15.1	24432053	T/C	7.18E‐09	0.3612	−0.083	0.017
rs7006786	ARHGEF10	8q13.2	1792639	G/A	8.81E‐09	0.08986	0.097	0.045
rs35483531	DEGS2	14q21.3	100653772	C/T	1.35E‐08	0.2993	−0.004	0.024
rs170084	PMFBP1	16q11.2	72178483	T/A	2.79E‐08	0.107	−0.068	0.029
rs10940421	SNX18	5q14.3	54036059	A/G	3.34E‐08	0.372	0.040	0.017
rs138908633	EPB41L4A	5q14.3	111649017	G/A	3.76E‐08	0.03095	−0.029	0.050
rs74435987	DUSP6	12q14.1	89152253	G/T	4.20E‐08	0.08766	0.078	0.130
rs11225924	DDI1	11q13.1	103493165	C/T	4.27E‐08	0.1034	0.152	0.105
rs113747850	MAPK9	5q14.3	179710663	T/C	4.93E‐08	0.123	0.071	0.024

*Note*: The meta‐analysis includes European, African, Asian, and Hispanic/Latino ancestries. Three new variants at 20q11.21, 2q14.1, and 15q15.1 reached genome‐wide significance (highlighted in orange).

Abbreviation: MAF, minor allele frequency.

**TABLE 5:** Genome‐wide significant (*p* < 5 × 10^−8^) and suggestive (*p* < 1 × 10^−6^) variants associated with vascular dementia in cross‐ancestry meta‐analysis.

rsID	Nearest gene	Chr	Pos	EA/NEA	*p* value	MAF	Beta	SE
rs10119	TOMM40	19	45406673	G/A	1.21E‐17	0.2476	−0.327	0.054
rs4380108	MARCHF10	17	60893485	C/T	9.59E‐09	0.3127	−0.172	0.031
rs55747619	ITSN2	2	24530447	C/G	8.05E‐08	0.08706	−0.384	1.969
rs9379092	CAGE1	6	7344531	G/A	9.80E‐08	0.1172	−0.336	0.077
rs3757193	RPS6KA2	6	166923463	C/T	1.09E‐07	0.08347	2.151	0.636
rs3871399	CMTM7	3	32496413	C/G	2.61E‐07	0.124	0.550	0.640
rs17315346	BRINP2	1	177282235	C/T	2.67E‐07	0.01538	−2.412	5.17
rs1738249	DNAH8	6	38753960	C/T	2.86E‐07	0.3013	−0.050	0.040
rs12095469	OSBPL9	1	52206082	G/A	3.60E‐07	0.05292	3.654	3.513
rs4820650	ADRBK2	22	25925358	T/C	3.82E‐07	0.2468	0.050	0.054
rs61859886	MGMT	10	131353192	T/G	4.45E‐07	0.1528	−0.274	0.057
rs9857196	RYK	3	133830660	T/A	5.09E‐07	0.01997	3.438	2.670
rs637924	PCDH7	4	31465610	T/C	6.77E‐07	0.2564	−0.056	0.051
rs35810115	ZNF675	19	23780763	C/T	6.81E‐07	0.04992	−1.169	2.339
rs115331896	CRBN	3	3204942	T/G	6.95E‐07	0.01218	3.139	2.706
rs4401880	SLC18A1	8	19946066	C/T	7.20E‐07	0.3249	0.019	0.051
rs4823298	FBLN1	22	45915987	T/C	7.96E‐07	0.4581	0.029	0.0439
rs17335455	NXPH1	7	8853946	T/G	8.20E‐07	0.1633	−0.096	0.042
rs517484	RP11‐6N13.1	5	104490130	T/C	8.51E‐07	0.1965	−0.120	0.046
rs12814413	RBMS2	12	56916614	T/C	8.77E‐07	0.3514	0.050	0.051
rs4665372	CGREF1	2	27325837	T/A	9.19E‐07	0.3948	−0.104	0.043

*Note*: The meta‐analysis includes European, African, Asian, and Hispanic/Latino ancestries. Genome‐wide significant variants are highlighted in orange.

Abbreviation: MAF, minor allele frequency.

### 3.3 Functional characterization of GW suggestive signals for ACD and VaD meta‐analyses

#### 3.3.1 Shared genetic susceptibility with complex disease traits

**FIGURE 4:**
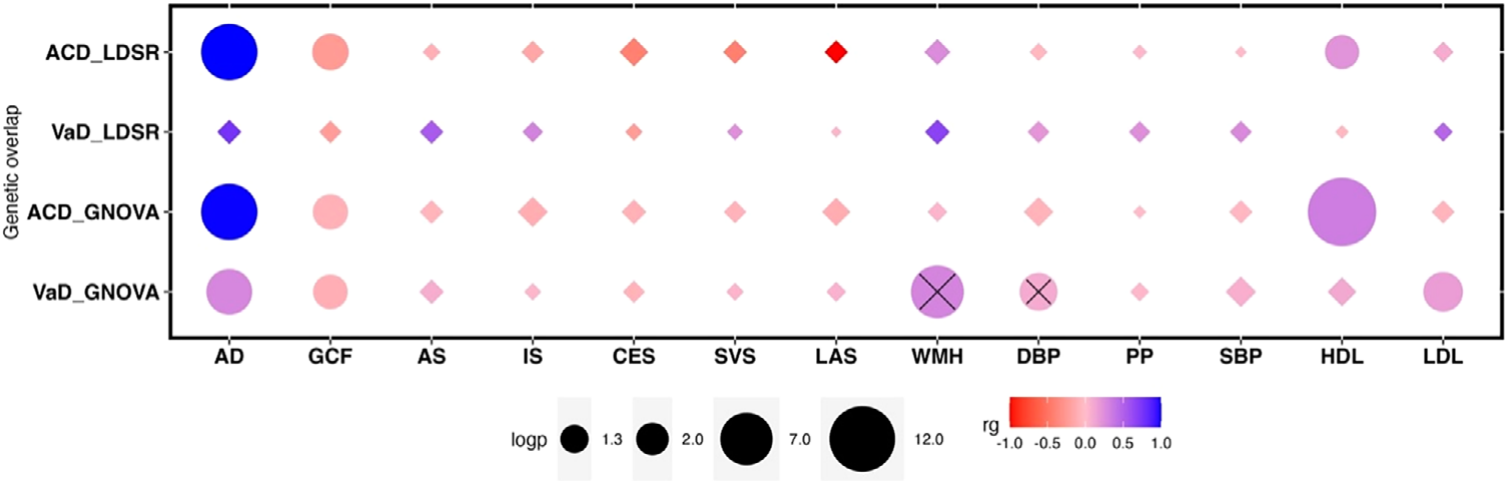
Shared genetic contribution between ACD/VaD and related risk factors. Contributions determined by LD score regression analysis (LDSR) (top), and Genetic Covariation Analyzer (GNOVA) (bottom). Effect sizes (rg) and significance levels (logp) are represented by color and symbol size. AD, Alzheimer's disease; GCF, general cognitive function; all stroke (AS) and its subtypes (ischemic, IS; cardioembolic, CES; small vessel, SVS; large artery, LAS); WMH, white matter hyperintensity burden; DBP, diastolic blood pressure; SBP, systolic blood pressure; PP, pulse pressure; HDL, high‐density lipoprotein; LDL, low‐density lipoprotein. Diamond shape: non‐significant. Cross‐significant causal effect estimates from MR‐LAP analysis.

The substantial shared genetic susceptibility of ACD and VaD with risk factors and complex disease traits is evident across different genomic scales (single variant, regional, and the global level). ACD exhibits genetic pleiotropy with vascular risk factors (hypertension, WMH burden), hematological traits (neutrophil, lymphocyte count), and blood‐based biomarkers indicative of inflammation (C‐reactive protein levels), hemostasis (fibrinogen, factor‐VII levels), and neurodegeneration (soluble *TREM2* levels) (Table S6). This shared genetic susceptibility is primarily driven by the *MS4A* gene famil*y* (membrane‐spanning 4A; *MS4A6A*, *MS4A4A*). The sharing of common genetic variation between ACD and vascular risk factors (blood pressure traits [DBP, SBP, PP], and T2D) at the *ACE* and *PILRB* locus (Figure S68, Table S6) is further supported by our regional Bayesian pairwise (GWAS‐PW) analysis highlighting the high probability of harboring a shared causal variant (Table S7). Interestingly, the GWAS‐PW approach additionally reveals the shared genetic susceptibility of VaD with IS and WMH at the *PRPF8* and *PRDM6* locus. In support, global‐level genetic overlap analysis (excluding the *APOE* region) using GNOVA showed statistically robust evidence for the association of increased levels of WMH with increased risk of VaD (Table S26, Figure 4). Additionally, we observed an inverse association of high levels of HDL (protective) with ACD risk and high levels of DBP and LDL with VaD risk. As expected, a strong genetic correlation between poorer cognitive performance (GCF) and ACD was also observed. Our causal inference analysis, using MR‐LAP, confirmed the putative causal association of increased DBP and WMH levels with VaD risk. However, the genetic correlation between AD and related risk factors using Kunkle 2019 GWAS did not show this causal association (Tables S26–S27, Figure 4).

### 3.4 Polygenicity and multi‐trait analysis

To identify additional SNPs conferring susceptibility to ACD and acting through related risk factors, we jointly studied the GW distribution of genetic effects for ACD and its closely related traits. We first prioritized those traits that have a polygenic background similar to ACD using a Bayesian approach (*ashR*). The *ashR* analysis showed that certain traits (ACD, stroke and its subtypes, WMH, coronary artery disease [CAD]) had specific, possibly overlapping, pathophysiological processes compared to other ACD risk factors (SBP, smoking [SMK], body mass index) that involved multiple biological pathways (Figure S69, Table S9). Next, using multitrait GWAS analysis (MTAG, see Methods) on ACD and the prioritized traits (CAD, stroke, WMH), we identified intronic SNPs in *SMG6* and *ABCG8* to be GW significant (pMTAG < 1.67E‐08, for three phenotypes) for ACD (Table S10). Interestingly, *SMG6* may also have a role in tau biology.^44^ Finally, we explored the genetic difference between ACD/VaD and related disorders using case–case GWAS (CC‐GWAS, see Methods). Specifically, we compared (1) ACD/VaD with AD and (2) ACD with stroke. VaD was not compared with stroke because the two disorders are highly correlated. Here we report signals that were not GW significant in both respective case‐control GWASs. For AD, we identified two loci associated with ACD‐AD status, including the known *APOE* region and the *IQUB* gene (*p* < 2e‐08) on chr7 (Table S11). No GW significant loci were associated with VaD‐AD status, although we observed some suggestive association (Table S12). For ACD‐stroke, we identified 56 variants mapping to 10 genes on chromosomes 17, 8, 11, 15, 4, and 12, most of which are located at the *SREBF1*/*TOM1L2* locus (*p* < 1e‐10) on chr17 (Table S13).

### 3.5 Functional prioritization using molecular profile (gene expression)

To functionally characterize and prioritize individual ACD and VaD genomic risk loci, we performed TWASs using TWAS‐Fusion, ACD, and VaD association statistics and weights from 23 gene‐expression reference panels from blood, arterial, and brain tissues (see Methods). We identified 29 trait‐associated (ACD/VaD) SNPs functioning as eQTLs, regulating the expression of 22 genes (eGenes) in disease‐relevant tissue types (Table S14). To explore whether the observed associations are real or merely reflect the random overlap between eQTLs and non‐causal risk variants for the dementia traits, a colocalization analysis was performed at each significant locus estimating the posterior probability of a shared causal variant (PP4 ≥ 75%) between the gene expression and trait association. Overall, 30% of the eQTL‐eGene satisfied the colocalization threshold for a shared causal variant between the ACD or VaD and gene expression. In addition to fine mapping functional genes (*RP11‐385F7.1*, *CR1*, *MS4A6A*, *ACE*, *APOC4*) in the loci exhibiting GW association with ACD/VaD, the TWASs identified putative novel (*CLU‐*ACD, *PIKFYVE‐*VaD, *SH3D21‐*ACD) genes satisfying transcriptome‐wide significance threshold (pTWAS < 1.18E‐05) and the colocalization probability threshold. Most (91%) of the eGenes are supported by the positional overlap of corresponding eQTLs with regulatory marks (enhancer and promoter binding sites) for active transcription in relevant tissue types.

### 3.6 Protein–protein interaction (PPI) evidenced SEMA4D, RBFOX1, and SPRY2 as hub genes for ACD and VaD

To determine the functional interactome of genes near genome‐wide significant (excluding *APOE* region) and suggestive loci (*p* < 1e‐6) associated with ACD and VaD, we performed a PPI analysis using the STRING database. The analysis comprised 82 ACD and 21 VaD GW significant and suggestive genes that were successfully mapped to the human genome. Evidence of interaction between proteins was based on “experiments,” “co‐occurrence,” “database,” and “co‐expression,” with a minimum score of 0.15. Non‐connected proteins were removed from the network. To further determine how suggestive genes will fit in the network of known AD genes, we used *kmeans* to cluster the proteins based on validated interaction. ACD genes formed two main clusters (Figure S70). The first cluster was enriched in known AD genes, including *BIN1*, *CLU*, *ABCA7*, and *CR1*, but also suggestive genes, including *SEMA4D*, *CHD18*, and *APH1B*, with more than two types of connection evidence. *RBFOX1* appears to be a major hub gene for the second cluster, which includes other suggestive genes like *AJAP1*, *ANO3*, and *TRIB1*. *RBFOX1* and *SEMA4D* strongly (>2 evidence of connection) interact with known AD genes, suggesting their potential role in ACD. The PPI network of VaD (Figure S71) genes highlights the potential role of *SPRY2* as it functionally connects other genes, including *ERBB4*, *RASGRP1*, and *FOXA2*.

### 3.7 Pathway and functional enrichment analysis

We conducted several analyses (pathways, gene ontology, disease enrichment) to obtain functional and biological contexts of genes (near variants with *p* < 1e‐6, excluding the APOE region) associated with ACD and VaD.

#### 3.7.1 Pathway analysis

Pathway analyses (Tables S15 and S16) revealed enrichment in several pathways, including “SREBF and miR33 in cholesterol and lipid homeostasis,” “Hypertrophy model,” and “Cholesterol metabolism with Bloch and Kandutsch‐Russell pathways” for ACD.

#### 3.7.2 Gene Ontology (GO) analysis

GO analysis for ACD (Figure S72 and Table S17) focusing on the biological processes (GO‐BP) were enriched in terms related to amyloid‐beta, “amyloid‐beta metabolic process,” “amyloid precursor protein catabolic process,” and “negative regulation of amyloid precursor protein catabolic process” for ACD. For VaD (Figure S73 and Table S18), GO‐BP analysis was enriched in several terms, including “response to glucose,” “response to hexose,” “response to monosaccharide,” “mesenchymal cell differentiation,” and “response to carbohydrate.”

#### 3.7.3 Disease enrichment and association analysis

(Figures S74 and S75, Tables S19–S25) revealed that ACD genes were previously connected to AD, tauopathy, nephritis, and central nervous system disease. It also highlighted previous associations of *SEMA4D* and *RBFOX1* with diseases of the central nervous system. Besides the AD connection, VaD genes were previously related to cancer, diabetes, and colorectal carcinoma. Finally, we used Framingham Heart Study data to estimate the heritability of VaD and the genetic correlation with ACD. We found the heritability of VaD to be 6.1%, with a 95% confidence interval of [3.2%, 21%]. The genetic correlation of VaD and ACD was 0.48 (SE = 0.84).

## 4 DISCUSSION

Our findings expand the current knowledge base of dementia genetics by focusing on both ACD and VaD. Our GWAS of ACD replicated several genes previously associated with AD, and GWAS of VaD identified SNPs in the *APOE* region. Using functional PPI and TW analyses, we identified novel genes underlying ACD that have been implicated in recovery from vascular injury and in neurotrophin signaling. On the basis of LD score regression analysis, we suggest that certain vascular risk factors may not have a causal role not in both ACD and VaD pathogenesis.

In our ACD analysis of European ancestry, we identified 10 GW significant loci, including *APOE*, *BIN1*, *MS4A6A*, *PICALM*, *CR1*, *CD2AP*, *ABCA7*, *PILRB*, *SLC24A4*, and *ACE*, all of which have been linked with AD risk in prior studies.^45^ In addition, our analyses highlighted 24 suggestive risk loci, of which 13 are novel. Among them are variants located near *ANO3*, a gene that encodes anoctamin‐3, a transmembrane protein that belongs to a family of calcium‐activated chloride channels and is implicated in focal dystonia, particularly craniocervical.^46^ Another suggestive locus was located near *SEMA4D*, a gene that encodes Semaphorin 4D and is known to modulate various processes related to neuroinflammation and neurodegeneration, including the initiation of inflammatory microglial activation.^47^ Indeed, SEMA4D is critical in regulating the transition between homeostatic and reactive states of various types of glial cells. Antibody blockade of *SEMA4D* is being explored as a potential disease‐modifying strategy to slow cognitive decline in patients with early Huntington's disease^48^ and may be beneficial in other ACD. We have also identified a prominent signal near *RBFOX1*, a gene that encodes the RNA binding fox‐1, which has been shown to have a role in alternative splicing of the amyloid precursor protein. Genetic variation in this gene has been associated with brain amyloid burden in preclinical and early AD and with the risk of clinical AD in African Americans.^49^ This gene may also impact dementia risk through non‐amyloidogenic pathways as it additionally regulates neuron development and neuronal excitability, including brain‐derived neurotrophic factor (BDNF)‐dependent long‐term potentiation in the hippocampus and has been implicated in brain development, essential tremor, and schizophrenia.^50^

Other suggestive loci are located in the *ZNF652* gene, a transcriptional repressor involved in nucleic acid binding that has diverse effects, including determining the risk of hypertension. Hypertension is the most important risk factor for stroke and WMH and may be the most important modifiable risk factor for population prevention of dementia.^51^ We additionally identified a variant near Heparin Binding EGF like growth factor (*HBEGF*), a growth factor implicated in the pathobiology of cerebral autosomal dominant arteriopathy with sub‐cortical infarcts and leukoencephalopathy (CADASIL),^52^ the major Mendelian prototype of VaD. HBEGF also has an effect on angiogenesis, expression of vascular endothelial growth factor A (VEGF‐A), inflammation, and oxidative stress and has been implicated in hydrocephalus.^53^

*APOE* was strongly associated with both ACD and VaD in our meta‐analysis. While AD could drive the association of APOE with ACD, the relationship with VaD is less established but has been demonstrated in some population studies and candidate‐gene analyses^54^ and in a recent GWAS among the GR@ACE project participants.^16^ The link of *APOE* with VaD is in line with recent literature suggesting that the pathogenesis of *APOE* extends beyond Aβ peptide aggregation and clearance.^55^ Indeed, *APOE* also influences microglia and the blood‐brain barrier (BBB)^56^ and is associated with intracranial atherosclerosis,^57^ WMH burden, and the presence of cerebral microbleeds,^58^ as well as with cerebral hypertensive angiopathy, which is common in individuals with VaD.^59^

In addition to a significant association of *APOE* with VaD in our sample, we identified several suggestive variants also associated with VaD. These include variants near the *SPPRY2* protein‐coding gene as well as *GALNT8*, *FOXA1*, *ERBB4*, *PSMA3*, and *SEMA6D* with consistency across samples in the direction of effect and many SNPs in LD with the lead SNP. Our downstream analyses supported a highly plausible causal link between variants, including *SEMA4D*, *HBEGF*, *PIKFYVE*, and *RBFOX1* with ACD and *SPRY2* with VaD. These genes collectively emphasize a possible role for novel pathological mechanisms in ACD and VaD. Our findings highlight a crucial mechanism underlying ACD: recovery after vascular injury. For example, *SEMA4D*, a member of the semaphorin family, is upregulated in the neurovascular unit after IS, where it exerts multiple neuroprotective effects.^60^ Moreover, this gene has been additionally highlighted in our PPI analysis as strongly associated with known AD genes. Another example is *SPRY2*, highlighted in our study as a suggestive gene for VaD, with strong functional associations with known AD and related dementias genes.

In the replication analysis of VaD signals in the EADB dataset, *SPRY2* has the lowest *p* value and a consistent direction of association. This gene has also been suggested as a possible pharmacological target for stroke patients, as it promotes angiogenesis and glial scarring around the ischemic injury, preventing an increase in lesion size and secondary damage to brain tissue.^61^ Also, *SPRY2* may exert neuroprotective effects as its expression regulates BDNF‐induced signaling pathways.^62^ Similarly, *PIKFYVE* is an essential regulator of platelet lysosome homeostasis, which in turn may promote recovery after IS.^63^ Another hub gene in our analyses is *RBFOX1*, which, in addition to having a role in amyloid accumulation as discussed earlier, mediates ischemic damage by enhancing neuronal survival and BBB integrity after stroke.^64^ This gene is a neuron‐specific splicing factor implicated in intellectual disability, epilepsy, autism, and Parkinson's disease. Its downregulation has been associated with destabilizing mRNAs encoding for synaptic transmission proteins, which may contribute to the loss of synaptic function in AD.^65^ Furthermore, *RBFOX1* upregulation was shown to influence neuronal expression levels of the BDNF receptor, TrkB, which in turn may affect the risk for ACD.^66^

We found that the MS4A gene cluster drove genetic pleiotropy that involves vascular risk factors, inflammation, hemostasis, and soluble TREM2 levels. These findings align with preclinical studies^67^ and emphasize the critical role and multifactorial contribution of this gene cluster to ACD pathogenesis. Although previous literature pointed to an association of *ACE* with AD but not VaD,^68^ we herein show that this gene underlies both ACD and vascular risk factors. A recent study supports this finding by showing that overexpression of *ACE* on macrophages reduces vascular amyloid and GFAP+ astroglial reactivation, indicating its role in the protection of the neurovascular unit.^69^ Moreover, our pairwise analysis highlighted a locus at the *PRDM6* that explained a shared genetic susceptibility of VaD with IS and WMH. Low levels of leukocyte DNA methylation of the *PRDM6* gene have been associated with an increased risk of IS and worse outcomes 3 months after an IS.^70^ Moreover, *PRDM6* acts as an epigenetic regulator of vascular smooth muscle cell plasticity.^71^

Despite evidence showing an inverse relationship between plasma HDL levels and risk of incident AD, results are conflicting, with some studies pointing to higher dementia risk in individuals with high HDL levels, as was also the case in our study.^72^ It should be acknowledged that HDL represents a class of lipoproteins that are heterogeneous in structure and function, which is not reflected by a simple measurement of HDL plasma levels. High HDL levels can be deleterious under certain conditions.^73^ Vascular risk factors and the presence of cardiovascular disease can alter HDL functionality by changing the structure of HDLs and converting them into pro‐inflammatory, pro‐oxidant, prothrombotic, and proapoptotic compounds. Our observation aligns with recent Mendelian randomization data implicating an elevated HDL in risk of AD.^74^

The following limitations should be considered when interpreting the results of this study. First, the multifactorial nature and heterogeneous clinical manifestations of ACD and VaD have led to various attempts to develop diagnostic criteria, which were differentially applied across the participating cohorts. ACD has been ascertained using DSM‐IV in some studies. In contrast, others have used ICD‐9/10 codes alone or in combination with autopsy or death certificate information, which can result in a varying proportion of persons identified as having dementia. The various cohorts also used different diagnostic criteria to define VaD. In all cohorts, a key requirement for VaD diagnosis remains the demonstration of a cognitive deficit and the presence of cerebrovascular disease, consistent with the most recent consensus criteria for VCID.^75^ Whereas these criteria differ in sensitivity and specificity, thereby introducing statistical noise, this heterogeneity does not diminish the importance of the loci identified despite the constraints. A second limitation is the limited power to identify associations with VaD in ancestries other than European.

Our study identified several putative genetic variants and biological pathways associated with ACD and VaD and added additional support for the involvement of vascular mechanisms in dementia pathogenesis.

## CONFLICT OF INTEREST STATEMENT

Agustin Ruiz and Itziar de Rojas acknowledge research support from Grifols SA (Spain), Fundacion Bacaria LaCaixa (Spain), Instituto de Salud Carlos III Ministry of Health (Spain), Roche, and Janssen. Agustin Ruiz received consulting fees and honoraria from Landsteiner Genmed SL, Grifols SA, and Janssen; support for attending meetings from Grifols SA; and stock options from Landsteiner Genmed SL. All authors report no conflicts of interest. Additional author disclosures are available in the supporting information (Supplementary File 2).

## CONSENT STATEMENT

All participants provided written informed permission, or, for those with substantial cognitive impairment, consent was provided by a caregiver, legal guardian, or other proxy. Author disclosures are available in the supporting information.

## SOFTWARE AVAILABILITY

CC‐GWAS: https://github.com/wouterpeyrot/CCGWAS

Gene expression weights for TWAS: http://gusevlab.org/projects/fusion/

HESS: https://huwenboshi.github.io/hess/local_hsqg/

LDSR: https://github.com/bulik/ldsc

GWAS‐PW: https://github.com/joepickrell/gwas‐pw

Radial‐MR: https://github.com/WSpiller/RadialMR

GREP: https://github.com/saorisakaue/GREP

EPIGWAS: https://immunogenomics.hms.harvard.edu/code

Magma.Celltyping: https://github.com/NathanSkene/MAGMA_Celltyping

MR‐MEGA: https://www.geenivaramu.ee/en/tools/mr‐mega

## DEFINITIONS

GWAS: Genome‐Wide Association Analysis Study

ACD: All‐cause dementia

VaD: Vascular dementia

VCID: Vascular Cognitive Impairment and Dementia

CHARGE: Cohorts for Heart and Aging Research in Genomic Epidemiology

ADGC: Alzheimer's Disease Genetics *Consortium*

UKBB: UK Biobank

EADB: European Alzheimer Disease DNA BioBank

## Supporting information

Supporting Information

Supporting Information

Supporting Information

Supporting Information

## Data Availability

The data that support the findings of this study are available on request from the corresponding author. The data are not publicly available due to privacy or ethical restrictions. https://ctg.cncr.nl/software/summary_statistics, https://pubmed.ncbi.nlm.nih.gov/36180795/

## 1 APPENDIX COLLABORATORS

Bernard Fongang^1,2,3,#,*^, Muralidharan Sargurupremraj^1,3,*^, Xueqiu Jian^1,3^, Aniket Mishra^4^, Vincent Damotte^5^, Itziar de Rojas^6,7^, Olivia Skrobot^8^, Joshua C. Bis^9^, Kang‐Hsien Fan^10^, Erin Jacobsen^11^, Gloria Hoi‐Yee Li^12^, Jingyun Yang^13^, Bizzarro Alessandra^14^, Lauria Alessandra^14^, Saima Hilal^15,16^, Joyce Ruifen Chong^15^, Yuek Ling Chai^15^, M. J. Knol^17^, Maria Pina Concas^18^, Girotto Giorgia^18,19^, Moeen Riaz^20^, Chenglong Yu^20^, Alexander Guojonsson^21^, Paul Lacaze^20^, Adam C Naj^22^, Monica Gireud‐Goss^1^, Yannick N. Wadop^1^, Aicha Soumare^4^, Vincent Bouteloup^4,23^, Vilmundur Gudnason^21,24^, Petronilla Battista^25^, Aurora Santin^19^, Beatrice Spedicati^19^, Rodolfo Sardone^26,27^, Lenore Launer^28^, Jan Bressler^29^, Rebecca F Gottesman^30^, Quentin Le Grand^31^, Ilana Caro^31^, Gennady V. Roshchupkin^32,33^, Hampton L. Leonard^34,35,36^, Chaojie Yang^37,38^, Traci M. Bartz^39,40^, Constance Bordes^31^, Paul M. Ridker^41,42^, Mirjam I. Geerlings^43,44,45,46^, Natalie C. Gasca^40^, Ani Manichaikul^37^, Mike A. Nalls^34,35,36^, Stephen S. Rich^37^, Carsten O. Schmidt^47^, Stella Trompet^48,49^, Jessica van Setten^50^, Marion van Vugt^50^, Hans J. Grabe^51,52^, J Wouter Jukema^49,53,54^, Ina L. Rissanen^55^, Sylvia Wassertheil‐Smoller^56^, M. Arfan Ikram^32^, Eleanor M. Simonsick^57^, W T. Longstreth^58,59^, Daniel I. Chasman^41,42^, Jerome I. Rotter^60^, Naveed Sattar^61^, David J Stott^62^, Eric J Shiroma^63^, Sigurdur Sigurdsson^24^, Mohsen Ghanbari^32^, Ulf Schminke^64^, Eric Boerwinkle^29,65^, Hugo J Aparicio^66,67^, Alexa S Beiser^66,68^, Jose R Romero^66,67^, Vasileios Lioutas^66,69^, Ruiqi Wang^66,68^, Chloe Sarnowski^70,71^, Alexander Teumer^51,72^, Uwe Völker^72,73^, Thomas H. Mosley^74^, Marta Marquié^6,7^, Pablo García‐González^6,7^, Clàudia Olivé^6^, Raquel Puerta^6^, Amanda Cano^6,7^, Oscar Sotolongo‐Grau^6,7^, Sergi Valero^6,7^, Vanesa Veronica Pytel^6^, Maitée Rosende‐Roca^6,7^, Montserrat Alegret^6,7^, Lluís Tàrraga^6,7^, Mercè Boada^6,7^, Ángel Carracedo^75,76^, Emilio Franco‐Macías^7,77^, Gerard Piñol‐Ripoll^78,79^, Guillermo Garcia‐Ribas^7,80,81,82^, Jordi Pérez‐Tur^7,82^, Jose Luís Royo^83^, Jose María García‐Alberca^84^, Luis Miguel Real^85,86^, María Eugenia Sáez^87^, María J. Bullido^7,88,89,90^, Miguel Calero^7,91,92^, Miguel Medina^7,93^, Pablo Mir^7,94,95^, Pascual Sánchez‐Juan^7,96^, Pau Pastor^97,98^, Victoria Álvarez^99,100^, Benjamin Grenier‐Boley^5^, Fahri Küçükali^101,102,103^, Sven Van der Lee^104,105,106^, Oliver Peters^107,108^, Anja Schneider^109,110^, Martin Dichgans^111,112,113^, Dan Rujescu^114^, Jürgen Deckert^115^, Emrah Düzel^116,117^, Jens Wiltfang^118,119,120^, Michael Wagner^121,122^, Timo Grimmer^123^, Nikolaos Scarmeas^124,125^, Fermin Moreno^7,126,127^, Raquel Sánchez‐Valle^128^, Luis M Real^85,129^, Eloy Rodriguez‐Rodriguez^7,130^, Adolfo Lopez de Munain^7,126,131^, Alexandre de Mendonça^132^, Jakub Hort^133,134^, Caroline Graff^135^, Goran Papenberg^136^, Vilmantas Giedraitis^137^, Børge G. Nordestgaard^138,139^, Hilkka Soininen^140^, Miia Kivipelto^141,142,143,144,145^, Annakaisa Haapasalo^146^, Gael Nicolas^147^, Florence Pasquier^148^, Olivier Hanon^149^, Edna Grünblatt^150,151,152^, Daniela Galimberti^153,154^, Beatrice Arosio^155,156^, Patrizia Mecocci^157^, Alessio Squassina^158^, Lucio Tremolizzo^159^, Innocenzo Rainero^160^, Davide Seripa^161^, Julie Williams^162^, Philippe Amouyel^163^, Frank Jessen^109,164,165^, Tsolaki Magda^166^, Ruth Frikke‐Schmidt^167,168^, Kristel Sleegers^101,102,169^, Sebastiaan Engelborghs^170,171^, Rik Vandenberghe^172,173^, Martin Ingelsson^174,175,176^, Giacomina Rossi^177^, Mikko Hiltunen^178^, Rebecca Sims^162^, Magdalena Gugała‐Iwaniuk^179^, Mitchell K. P. Lai^15^, Venketasubramanian N^180^, Boon‐Yeow Tan^181^, Angelo Baldassare Cefalù^182^, Nicola J Armstrong^183^, Roberta Baschi^184,185^, Regis bordet ^186,187^, Anne‐Marie Bordet^186,187^, Henry Brodaty^188^, Srdjan Djurovic^189,190^, Grazia D'Onofrio^191^, Margaret Esiri^192^, Patrick Gelé^186,187^, Teresa Juarez‐Cedillo^193^, Raj Kalaria^194,195^, Pekka Karhunen^196^, Jan LACZO^133^, Ondrej LERCH^133,134^, Carlo Masullo^197^, Karen A Mather^188,198^, Vaclav MATOSKA^199^, Susanna Melkas^200^, Roberto Monastero^184,185^, Katya Numbers^188^, Francesco Panza^201,202,203^, Tuomo M Polvikoski^195,204^, Joe Quinn^205^, Arvid Rongve^206,207^, Perminder S Sachdev^188,208^, Michela Scamosci^209^, Anbupalam Thalamuthu^188^, Anne Tybjærg‐Hansen^210^, Martin VYHNALEK^133,134^, Shawn K. Westaway^211^, Amy E Martinsen^212,213,214^, Anne Heidi Skogholt^214^, Cristen J Willer^215^, Eystein Stordal^216,217^, Geir Bråthen^218,219,220^, Jonas Bille Nielsen^214,215^, Lars G Fritsche^221^, Laurent F Thomas^214,222,223,224^, Linda M Pedersen^212^, Maiken E Gabrielsen^214^, Ole Kristian Drange^216,225^, Sigrid Botne Sando^214,218,226^, Tore Wergeland Meisingset^218,226^, Genevieve Chene^4,23^, Wei Zhou^227,228^, Christophe Tzourio^4,229^, Adrienne Tin^230^, Oscar L Lopez^231^, Haan Mary^232^, Allison E Aiello^233^, Sigrid Børte^213,214,234^, Ingunn Bosnes^216,217^, Cornelia van Duijn^235,236,237^, Ching‐Lung Cheung^238^, David A Bennett^13^, Christopher Chen^15^, M. Ilyas Kamboh^10^, Claudia Satizabal^1,3^, M. Kamran Ikram^17,239^, Hieab Adams^240,241,242^, Yang Qiong^68^, Gerard D. Schellenberg^22^, Geir Selbæk^213,243,244^, Kristian Hveem^214,245,246^, Ole A Andreassen^247,248^, Alfredo Ramirez^109,249,250,251^, Carole Dufouil^4,23^, Wiesje van der Flier^252^, John‐Anker Zwart^212,213,214^, Stéphanie Debette^4,253^, Myriam Fornage^29,254^, Bendik Winsvold^214,255,256^, Jean‐Charles Lambert^5^, Agustin Ruiz^6,7^, Patrick G. Kehoe^257^, Galit Weinstein^258,#^, and Sudha Seshadri^1,259,260,261,#^

^1^ Glenn Biggs Institute for Alzheimer's & Neurodegenerative Diseases, University of Texas Health Science Center, San Antonio, TX, USA

^2^ Department of Biochemistry and Structural Biology, University of Texas Health Science Center, San Antonio, TX, USA

^3^ Department of Population Health Sciences, University of Texas Health Science Center, San Antonio, TX, USA

^4^ University of Bordeaux, Inserm, Bordeaux Population Health Research Center, UMR 1219, F‐33000 Bordeaux, France

^5^ Univ. Lille, Inserm, CHU Lille, Institut Pasteur de Lille, U1167‐RID‐AGE facteurs de risque et déterminants moléculaires des maladies liés au vieillissement, Lille, France

^6^ Research Center and Memory Clinic, ACE Alzheimer Center Barcelona. Universitat Internacional de Catalunya, Spain

^7^ Network Center for Biomedical Research in Neurodegenerative Diseases (CIBERNED), Instituto de Salud Carlos III, Madrid, Spain

^8^ Population Health Sciences, Bristol Medical School, University of Bristol, Bristol, UK

^9^ Cardiovascular Health Research Unit, Department of Medicine, University of Washington, Seattle, WA

^10^ Department of Human Genetics, School of Public Health, University of Pittsburgh, Pittsburgh, PA, USA

^11^ Department of Psychiatry and Neurology, School of Medicine, University of Pittsburgh, Pittsburgh, PA, USA

^12^ Department of Health Technology and Informatics, The Hong Kong Polytechnic University, Hung Hom, Hong Kong

^13^ Rush Alzheimer's Disease Center and Department of Neurological Sciences, Rush University Medical Center, Chicago, IL, USA

^14^ Geriatrics Unit, Policlinico Universitario Fondazione Agostino Gemelli IRCCS, Largo a Gemelli, 8−00168 Rome, Italy

^15^ Department of Pharmacology, National University of Singapore, Singapore

^16^ Saw Swee Hock School of Public Health, National University of Singapore and National University Health System, Singapore

^17^ Department of Epidemiology, Erasmus MC, University Medical Center, Rotterdam, the Netherlands

^18^ Institute for Maternal and Child Health, IRCCS Burlo Garofolo, 34127 Trieste, Italy

^19^ Department of Medicine, Surgery and Health Sciences, University of Trieste, 34139 Trieste, Italy

^20^ Department of Epidemiology and Preventive Medicine, Monash University, Melbourne, VIC, Australia

^21^ Faculty of Medicine, University of Iceland, Reykjavik, Iceland

^22^ Department of Biostatistics and Epidemiology/Center for Clinical Epidemiology and Biostatistics, University of Pennsylvania Perelman School of Medicine, Philadelphia, PA, USA

^23^ Pôle de Santé Publique Centre Hospitalier Universitaire (CHU) de Bordeaux, Bordeaux, France

^24^ Icelandic Heart Association, Kopavogur, Iceland

^25^ Istituti Clinici Scientifici Maugeri IRCCS, Laboratory of Neuropsychology, Bari Institute, Italy

^26^ Department of Translational Biomedicine and Neuroscience, University of Bari “Aldo Moro,” Bari, Italy

^27^ Unit of Statistics and Epidemiology, Local Healthcare Authority of Taranto, Taranto, Italy

^28^ Laboratory of Epidemiology and Population Sciences, Intramural Research Program, National Institute of Aging, National Institutes of Health, Bethesda, MD, USA

^29^ Human Genetics Center, School of Public Health, The University of Texas Health Science Center at Houston, Houston TX, USA

^30^ Stroke Branch, National Institute of Neurological Disorders and Stroke Intramural Program, National Institutes of Health, Bethesda, MD, USA

^31^ University of Bordeaux, Inserm, Bordeaux Population Health Research Center, team ELEANOR, UMR 1219, F‐33000 Bordeaux, France

^32^ Department of Epidemiology, Erasmus MC University Medical Center Rotterdam, Rotterdam, the Netherlands

^33^ Department of Radiology and Nuclear Medicine, Erasmus MC University Medical Center, the Netherlands

^34^ Center for Alzheimer's and Related Dementias, National Institutes of Health, Bethesda, MD, USA

^35^ Laboratory of Neurogenetics, National Institute on Aging, National Institutes of Health, Bethesda, MD, USA

^36^ Data Tecnica International LLC, Glen Echo, MD, USA

^37^ Center for Public Health Genomics, University of Virginia, Charlottesville, VA, USA

^38^ Department of Biochemistry and Molecular Genetics, University of Virginia, Charlottesville, VA, USA; Department of Biochemistry and Molecular Genetics, University of Virginia, Charlottesville, VA, USA

^39^ Cardiovascular Health Research Unit, Department of Medicine, University of Washington, Seattle, WA, USA

^40^ Department of Biostatistics, University of Washington, Seattle, WA, USA

^41^ Division of Preventive Medicine, Brigham and Women's Hospital, Boston, MA, USA

^42^ Harvard Medical School, Boston, MA, USA

^43^ Department of General Practice, Amsterdam UMC, location University of Amsterdam, Meibergdreef 9, Amsterdam, the Netherlands

^44^ Amsterdam Public Health, Aging & Later Life and Personalized Medicine, Amsterdam, the Netherlands

^45^ Amsterdam Neuroscience, Neurodegeneration and Mood, Anxiety, Psychosis, Stress, and Sleep, Amsterdam, the Netherlands

^46^ Julius Center for Health Sciences and Primary Care, University Medical Center Utrecht and Utrecht University, Utrecht, the Netherlands

^47^ University Medicine Greifswald, Institute for Community Medicine, SHIP/KEF, Greifswald, Germany

^48^ Department of Internal Medicine, Section of Gerontology and Geriatrics, Leiden University Medical Center, Leiden, the Netherlands

^49^ Department of Cardiology, Leiden University Medical Center, Leiden, the Netherlands

^50^ Division Heart & Lungs, Department of Cardiology, University Medical Center Utrecht, Utrecht University, Utrecht, the Netherlands

^51^ Department of Psychiatry and Psychotherapy, University Medicine Greifswald, Germany

^52^ German Center for Neurodegenerative Diseases (DZNE), Site Rostock/Greifswald, Rostock, Germany

^53^ Netherlands Heart Institute, Utrecht, the Netherlands

^54^ Einthoven Laboratory for Experimental Vascular Medicine, LUMC, Leiden, the Netherlands

^55^ Julius Center for Health Sciences and Primary Care, University Medical Center Utrecht, Utrecht University, Utrecht, the Netherlands

^56^ Department of Epidemiology and Population Health, Albert Einstein College of Medicine, New York, NY, USA

^57^ Longitudinal Studies Section, Translational Gerontology Branch, National Institute on Aging, Baltimore, MD, USA

^58^ Department of Epidemiology, University of Washington, Seattle, WA, USA

^59^ Department of Neurology, University of Washington, Seattle, WA, USA

^60^ Institute for Translational Genomics and Population Sciences, Department of Pediatrics, Lundquist Institute for Biomedical Innovation at Harbor‐UCLA Medical Center, Los Angeles, CA, USA

^61^ BHF Glasgow Cardiovascular Research Centre, Faculty of Medicine, Glasgow, UK

^62^ Institute of Cardiovascular and Medical Sciences, College of Medical, Veterinary and Life Sciences, University of Glasgow, UK

^63^ Laboratory of Epidemiology and Population Sciences—National Institutes of Health, Bethesda, MD, USA

^64^ University Medicine Greifswald, Department of Neurology, Greifswald, Germany

^65^ Human Genome Sequencing Center, Baylor College of Medicine, Houston, TX, USA

^66^ Framingham Heart Study, Framingham, MA, USA

^67^ Department of Neurology, Boston University School of Medicine, Boston, MA, USA

^68^ Department of Biostatistics, Boston University School of Public Health, Boston, MA, USA

^69^ Department of Neurology, Beth Israel Deaconess Medical Center, Boston, MA, USA

^70^ Department of Epidemiology, Human Genetics and Environmental Sciences, University of Texas Health Science Center at Houston, School of Public Health, Houston, TX, USA

^71^ Department of Epidemiology, Human Genetics and Environmental Sciences, The University of Texas School of Public Health, Houston, Texas, USA

^72^ DZHK (German Centre for Cardiovascular Research), Partner Site Greifswald, Greifswald, Germany

^73^ Interfaculty Institute for Genetics and Functional Genomics, University Medicine Greifswald, Greifswald, Germany

^74^ Memory Impairment and Neurodegenerative Dementia (MIND) Center and Department of Medicine, University of Mississippi Medical Center, Jackson, MS, USA

^75^ Grupo de Medicina Xenómica, CIBERER, CIMUS. Universidade de Santiago de Compostela, Santiago de Compostela, Spain

^76^ Fundación Pública Galega de Medicina Xenómica‐IDIS, Santiago de Compostela, Spain

^77^ Unidad de Demencias, Servicio de Neurología y Neurofisiología. Instituto de Biomedicina de Sevilla (IBiS), Hospital Universitario Virgen del Rocío/CSIC/Universidad de Sevilla, Seville, Spain

^78^ Unitat Trastorns Cognitius, Hospital Universitari Santa Maria de Lleida, Lleida, Spain

^79^ Institut de Recerca Biomedica de Lleida (IRBLLeida), Lleida, Spain

^80^ Hospital Universitario Ramon y Cajal, IRYCIS, Madrid, Spain

^81^ Unitat de Genètica Molecular, Institut de Biomedicina de València‐CSIC, Valencia, Spain

^82^ Unidad Mixta de Neurologia Genètica, Instituto de Investigación Sanitaria La Fe, Valencia, Spain

^83^ Departamento de Especialidades Quirúrgicas, Bioquímica e Inmunología. School of Medicine. University of Málaga, Málaga, Spain

^84^ Alzheimer Research Center & Memory Clinic, Instituto Andaluz de Neurociencia, Málaga, Spain

^85^ Unidad Clínica de Enfermedades Infecciosas y Microbiología, Hospital Universitario de Valme, Seville, Spain

^86^ Departamento de Especialidades Quirúrgicas, Bioquímica e Inmunología, Facultad de Medicina, Universidad de Málaga, Málaga, Spain

^87^ CAEBI, Centro Andaluz de Estudios Bioinformáticos, Sevilla, Spain

^88^ Centro de Biología Molecular Severo Ochoa (UAM‐CSIC)

^89^ Instituto de Investigacion Sanitaria “Hospital la Paz” (IdIPaz), Madrid, Spain

^90^ Universidad Autónoma de Madrid

^91^ CIEN Foundation/Queen Sofia Foundation Alzheimer Center/Instituto de Salud Carlos III

^92^ UFIEC, Instituto de Salud Carlos III, Madrid, Spain

^93^ CIEN Foundation/Queen Sofia Foundation Alzheimer Center, Madrid, Spain

^94^ Unidad de Trastornos del Movimiento, Servicio de Neurología y Neurofisiología. Instituto de Biomedicina de Sevilla (IBiS), Hospital Universitario Virgen del Rocío/CSIC/Universidad de Sevilla, Seville, Spain

^95^ Departamento de Medicina, Facultad de Medicina, Universidad de Sevilla, Seville, Spain

^96^ Alzheimer's Centre Reina Sofia‐CIEN Foundation, Centro de Investigación Biomédica en Red sobre Enfermedades Neurodegenerativas (CIBERNED), Madrid, Spain

^97^ Unit of Neurodegenerative Diseases, Department of Neurology, Hospital Germans Trias i Pujol, Badalona, Barcelona, Spain

^98^ Neurodegenerative Diseases Research Laboratory, Germans Trias i Pujol Research Laboratory, Badalona, Barcelona, Spain

^99^ Laboratorio de Genética, Hospital Universitario Central de Asturias, Oviedo, Spain

^100^ Instituto de Investigación Sanitaria del Principado de Asturias (ISPA), Oviedo, Spain

^101^ Complex Genetics of Alzheimer's Disease Group, VIB Center for Molecular Neurology, VIB, Antwerp, Belgium

^102^ Laboratory of Neurogenetics, Institute Born—Bunge, Antwerp, Belgium

^103^ Department of Biomedical Sciences, University of Antwerp, Neurodegenerative Brain Diseases Group, Center for Molecular Neurology, VIB, Antwerp, Belgium

^104^ Alzheimer Center Amsterdam, Neurology, Vrije Universiteit Amsterdam, Amsterdam UMC Location VUmc, Amsterdam, the Netherlands

^105^ Amsterdam Neuroscience, Neurodegeneration, Amsterdam, the Netherlands

^106^ Section Genomics of Neurodegenerative Diseases and Aging, Human Genetics, Vrije Universiteit Amsterdam, Amsterdam UMC location VUmc, Amsterdam, the Netherlands

^107^ German Center for Neurodegenerative Diseases (DZNE), Berlin, Germany

^108^ Charité – Universitätsmedizin Berlin, corporate member of Freie Universität Berlin, Humboldt‐Universität zu Berlin, and Berlin Institute of Health, Institute of Psychiatry and Psychotherapy, Hindenburgdamm 30, 12203 Berlin, Germany

^109^ German Center for Neurodegenerative Diseases (DZNE), Bonn, Germany

^110^ Department for Neurodegenerative Diseases and Geriatric Psychiatry, University Hospital Bonn, Venusberg‐Campus 1, 53127 Bonn, Germany

^111^ Institute for Stroke and Dementia Research (ISD), University Hospital, LMU Munich, Munich, Germany

^112^ German Center for Neurodegenerative Diseases (DZNE), Munich, Germany

^113^ Munich Cluster for Systems Neurology (SyNergy), Munich, Germany

^114^ Martin‐Luther‐University Halle‐Wittenberg, University Clinic and Outpatient Clinic for Psychiatry, Psychotherapy and Psychosomatics, Halle (Saale), Germany

^115^ Department of Psychiatry, Psychosomatics and Psychotherapy, Center of Mental Health, University Hospital of Würzburg, Germany

^116^ German Center for Neurodegenerative Diseases (DZNE), Magdeburg, Germany

^117^ Institute of Cognitive Neurology and Dementia Research (IKND), Otto‐von‐Guericke University, Magdeburg, Germany

^118^ Department of Psychiatry and Psychotherapy, University Medical Center Goettingen, Goettingen, Germany

^119^ German Center for Neurodegenerative Diseases (DZNE), Goettingen, Germany

^120^ Medical Science Department, iBiMED, Aveiro, Portugal

^121^ Department of Neurodegenerative Diseases and Geriatric Psychiatry, University of Bonn, Bonn, Germany

^122^ German Center for Neurodegenerative Diseases (DZNE), Bonn, Germany

^123^ Technical University of Munich, School of Medicine, Klinikum rechts der Isar, Department of Psychiatry and Psychotherapy

^124^ Taub Institute for Research in Alzheimer's Disease and the Aging Brain, The Gertrude H. Sergievsky Center, Department of Neurology, Columbia University, New York, NY, USA

^125^ 1st Department of Neurology, Aiginition Hospital, National and Kapodistrian University of Athens, Medical School, Greece

^126^ Department of Neurology, Hospital Universitario Donostia, San Sebastian, Spain

^127^ Neurosciences Area. Instituto Biodonostia. San Sebastian, Spain

^128^ Alzheimer's Disease and Other Cognitive Disorders Unit, Service of Neurology, Hospital Clinic of Barcelona, Institut d'Investigacions Biomèdiques August Pi i Sunyer, University of Barcelona, Barcelona, Spain

^129^ Depatamento de Especialidades Quirúrgicas, Bioquímica e Inmunología, Facultad de Medicina, Universidad de Málaga, Málaga, Spain

^130^ Neurology Service, Marqués de Valdecilla University Hospital (University of Cantabria and IDIVAL), Santander, Spain

^131^ Department of Neurosciences, Faculty of Medicine and Nursery, University of the Basque Country, San Sebastián, Spain

^132^ Faculty of Medicine, University of Lisbon, Portugal

^133^ Memory Clinic, Department of Neurology, Charles University, Second Faculty of Medicine and Motol University Hospital, Czech Republic

^134^ International Clinical Research Center, St. Anne's University Hospital Brno, Brno, Czech Republic

^135^ Unit for Hereditary Dementias, Theme Aging, Karolinska University Hospital‐Solna, 171 64 Stockholm, Sweden

^136^ Aging Research Center, Department of Neurobiology, Care Sciences and Society, Karolinska Institutet and Stockholm University, Stockholm, Sweden

^137^ Department of Public Health and Carins Sciences/Geriatrics, Uppsala University, Sweden

^138^ Department of Clinical Biochemistry, Copenhagen University Hospital – Herlev Gentofte, Denmark

^139^ Department of Clinical Medicine, University of Copenhagen, Denmark

^140^ Institute of Clinical Medicine—Neurology, University of Eastern Finland, Finland

^141^ Division of Clinical Geriatrics, Center for Alzheimer Research, Care Sciences and Society (NVS), Stockholm, Sweden

^142^ Karolinska Institutet, Stockholm, Sweden

^143^ Institute of Public Health and Clinical Nutrition, University of Eastern Finland, Kuopio, Finland

^144^ Neuroepidemiology and Ageing Research Unit, School of Public Health, Imperial College London, London, UK

^145^ Stockholms Sjukhem, Research & Development Unit, Stockholm, Sweden

^146^ A.I. Virtanen Institute for Molecular Sciences, University of Eastern Finland, Kuopio, Finland

^147^ University of Rouen Normandy, Normandy University, Inserm U1245 and CHU Rouen, Department of Genetics and CNRMAJ, Rouen, France

^148^ University of Lille Inserm 1171, CHU Clinical and Research Memory Research Centre (CMRR) of Distalz, Lille, France

^149^ Université de Paris, EA 4468, APHP, Hôpital Broca, Paris, France

^150^ Department of Child and Adolescent Psychiatry and Psychotherapy, University Hospital of Psychiatry Zurich, University of Zurich, Zurich, Switzerland

^151^ Neuroscience Center Zurich, University of Zurich and ETH Zurich, Switzerland

^152^ Zurich Center for Integrative Human Physiology, University of Zurich, Switzerland

^153^ Neurodegenerative Diseases Unit, Fondazione IRCCS Ca’ Granda, Ospedale Policlinico, Milan, IT

^154^ Department of Biomedical, Surgical and Dental Sciences, University of Milan, Milan, Italy

^155^ Department of Clinical Sciences and Community Health, University of Milan, Milan, Italy

^156^ Geriatric Unit, Fondazione IRCCS Ca’ Granda Ospedale Maggiore Policlinico, Milan, Italy

^157^ Institute of Gerontology and Geriatrics, Department of Medicine and Surgery, University of Perugia, Italy

^158^ Department of Biomedical Sciences, University of Cagliari, Cagliari, Italy

^159^ Neurology, “San Gerardo” Hospital, Monza and University of Milano‐Bicocca, Italy

^160^ Department of Neuroscience “Rita Levi Montalcini,” University of Turin, Turin, Italy

^161^ Department of Hematology and Stem Cell Transplant, Vito Fazzi Hospital, Lecce, Italy

^162^ Division of Psychological Medicine and Clinical Neuroscience, School of Medicine, Cardiff University, Wales, UK

^163^ Univ. Lille, Inserm, CHU Lille, Institut Pasteur de Lille, U1167‐RID‐AGE—LabEx DISTALZ, facteurs de risque et déterminants moléculaires des maladies liés au vieillissement, Lille, France

^164^ Department of Psychiatry and Psychotherapy, Faculty of Medicine and University Hospital Cologne, University of Cologne, Cologne, Germany

^165^ Cluster of Excellence Cellular Stress Responses in Aging‐Associated Diseases (CECAD), University of Cologne, Cologne, Germany

^166^ 1st Department of Neurology, Medical school, Aristotle University of Thessaloniki, Thessaloniki, Makedonia, Greece

^167^ Department of Clinical Biochemistry, Copenhagen University Hospital—Rigshospitalet, Copenhagen, Denmark

^168^ Department of Clinical Medicine, University of Copenhagen, Copenhagen, Denmark

^169^ Department of Biomedical Sciences, University of Antwerp, Antwerp, Belgium

^170^ Department of Neurology, Universitair Ziekenhuis Brussel and NEUR (Neuroprotection & Neuromodulation) Research Group, Center for Neurosciences, Vrije Universiteit Brussel (VUB), Brussels, Belgium

^171^ Reference Center for Biological Markers of Dementia (BIODEM), Institute Born‐Bunge, University of Antwerp, Antwerp, Belgium

^172^ Laboratory for Cognitive Neurology, Department of Neurosciences, University of Leuven, Leuven, Belgium

^173^ Neurology Department, University Hospitals Leuven, Leuven, Belgium

^174^ Department of Public Health and Carins Sciences/Geriatrics, Uppsala University, Sweden

^175^ Krembil Brain Institute, University Health Network, Toronto, Canada

^176^ Tanz Centre for Research in Neurodegenerative Diseases, Departments of Medicine and Laboratory Medicine & Pathobiology, University of Toronto, Toronto, Canada

^177^ Fondazione IRCCS Istituto Neurologico Carlo Besta, Milan, Italy

^178^ Institute of Biomedicine, University of Eastern Finland, Kuopio, Finland

^179^ Institute of Psychiatry and Neurology, First Department of Neurology, Warsaw, Poland

^180^ Raffles Neuroscience Center, Raffles Hospital, Singapore

^181^ St Luke's Hospital, Singapore, Singapore

^182^ Department of Health Promotion Sciences, Maternal and Infant Care (PROMISE), University of Palermo, Palermo, Italy

^183^ Department of Mathematics and Statistics, Curtin University, Perth, Australia

^184^ Department of Biomedicine, Neuroscience and Advanced Diagnostics (BIND), University of Palermo, Palermo, Italy

^185^ Dementia and Parkinson's disease Center, University Hospital, “Paolo Giaccone,” Palermo, Italy

^186^ Univ Lille, Inserm, CHU Lille, France

^187^ Lille Neuroscience & Cognition, Lille, France

^188^ Centre for Healthy Brain Ageing, Discipline of Psychiatry & Mental Health, School of Clinical Medicine, Faculty of Medicine and Health, University of New South Wales, Sydney, Australia

^189^ NORMENT Centre, University of Bergen, Bergen, Norway

^190^ Dept of Medical Genetics, Oslo University Hospital, Oslo, Norway

^191^ Clinical Psychology Service, Health Department, Fondazione IRCCS Casa Sollievo della Sofferenza, San Giovanni Rotondo (FG), Italy

^192^ Nuffield Department of Clinical Neurosciences, Oxford University, Oxford, UK

^193^ Unidad de Investigación en Epidemiología y Servicos de Salud Área Envejecimiento, Centro Medico Nacional Siglo XXI, Instituto Mexicano del Seguro Social. Ciudad de Mexico, Mexico

^194^ Translational and Clinical Research Institute, Newcastle University

^195^ Campus for Ageing and Vitality, Newcastle upon Tyne NE4 5PL, UK

^196^ Faculty of Medicine and Health Technology, Tampere University, and Department of Clinical Chemistry, Fimlab Laboratories. Tampere, Finland

^197^ Institute of Neurology, Catholic University of the Sacred Heart, School of Medicine, Largo Agostino Gemelli, Rome, Italy

^198^ Neuroscience Research Australia, Sydney, Australia

^199^ Department of Clinical Biochemistry, Hematology and Immunology, Na Homolce Hospital, Prague, Czech Republic

^200^ Helsinki University Hospital, University of Helsinki, Helsinki, Finland

^201^ Neurodegenerative Disease Unit, Department of Basic Medicine, Neuroscience, and Sense Organs, University of Bari Aldo Moro, Policlinico, Piazza Giulio Cesare 11, 70124 Bari, Italy

^202^ Geriatric Unit & Laboratory of Gerontology and Geriatrics, Department of Medical Sciences, IRCCS “Casa Sollievo della Sofferenza,” San Giovanni Rotondo, Viale Cappuccini 1, 71013 San Giovanni Rotondo, Foggia, Italy

^203^ Unit of Research Methodology and Data Sciences for Population Health, National Institute of Gastroenterology Saverio de Bellis, Research Hospital, Castellana Grotte, Bari, Italy

^204^ Translational and Clinical Research Institute, Newcastle University, Newcastle, UK

^205^ Department of Neurology, Oregon Health & Science University, Portland, Oregon, USA

^206^ Department of Research and Innovation, Helse Fonna, Haugesund, Norway

^207^ Department of Clinical Medicine (K1), University of Bergen, Bergen, Norway

^208^ Neuropsychiatric Institute, Euroa Centre, Prince of Wales Hospital, Sydney, Australia

^209^ Institute of Gerontology and Geriatrics, Department of Medicine, University of Perugia, Perugia, Italy

^210^ Department of Clinical Biochemistry, Copenhagen University Hospital – Rigshospitalet, Copenhagen Denmark & Department of Clinical Medicine, Copenhagen, Denmark

^211^ Department of Neurology, Oregon Health & Science University, Portland, Oregon, USA

^212^ Department of Research and Innovation, Division of Clinical Neuroscience, Oslo University Hospital, Oslo, Norway

^213^ Institute of Clinical Medicine, Faculty of Medicine, University of Oslo, Oslo, Norway

^214^ K. G. Jebsen Center for Genetic Epidemiology, Department of Public Health and Nursing, Faculty of Medicine and Health Sciences, Norwegian University of Science and Technology (NTNU), Trondheim, Norway

^215^ Department of Internal Medicine, Division of Cardiovascular Medicine, University of Michigan, Ann Arbor, MI, USA

^216^ Department of Mental Health, Faculty of Medicine and Health Sciences, Norwegian University of Science and Technology (NTNU), Trondheim, Norway

^217^ Department of Psychiatry, Hospital Namsos, Nord‐Trøndelag Health Trust, Namsos, Norway

^218^ Department of Neuromedicine and Movement Science, Faculty of Medicine and Health Sciences, Norwegian University of Science and Technology (NTNU), Trondheim, Norway

^219^ Department of Neurology and Clinical Neurophysiology, St. Olav's Hospital, Trondheim University Hospital, Trondheim, Norway

^220^ K G Jebsen Centre for Alzheimer's Disease. Kavli Institutes of Systems Neuroscience, Norwegian University of Science and Technology (NTNU), Trondheim, Norway

^221^ Center for Statistical Genetics, Department of Biostatistics, University of Michigan, Ann Arbor, MI, USA

^222^ Department of Clinical and Molecular Medicine, Norwegian University of Science and Technology (NTNU), Trondheim, Norway

^223^ BioCore—Bioinformatics Core Facility, Norwegian University of Science and Technology (NTNU), Trondheim, Norway

^224^ Clinic of Laboratory Medicine, St. Olavs Hospital, Trondheim University Hospital, Trondheim, Norway

^225^ Division of Mental Health Care, St. Olavs Hospital, Trondheim University Hospital, Trondheim, Norway

^226^ Department of Neurology and Clinical Neurophysiology, St. Olavs Hospital, Trondheim University Hospital, Trondheim, Norway

^227^ Department of Computational Medicine and Bioinformatics, University of Michigan, Ann Arbor, MI, USA

^228^ Analytic and Translational Genetics Unit, Massachusetts General Hospital, Boston, MA, USA

^229^ Bordeaux University Hospital, Department of Medical Informatics, Bordeaux, France

^230^ Memory Impairment and Neurodegenerative Dementia (MIND) Center and Department of Medicine, University of Mississippi Medical Center, Jackson, MS, USA

^231^ Department of Neurology, School of Medicine, University of Pittsburgh, PA, USA

^232^ Department of Epidemiology & Biostatistics, University of California, San Francisco, CA, USA

^233^ Department of Epidemiology, Carolina Population Center, University of North Carolina at Chapel Hill, Chapel Hill, NC, USA

^234^ Research and Communication Unit for Musculoskeletal Health (FORMI), Department of Research and Innovation, Division of Clinical Neuroscience, Oslo University Hospital, Oslo, Norway

^235^ Department of Epidemiology, Erasmus Medical Center, Rotterdam, the Netherlands

^236^ Nuffield Department of Population Health, University of Oxford, Old Road Campus, Headington, Oxford, UK

^237^ Big Data Institute, Li Ka Shing Centre for Health Information and Discovery, Old Road Campus, Headington, Oxford, UK

^238^ Department of Pharmacology and Pharmacy, Centre for Genomic Sciences, University of Hong Kong, Hong Kong

^239^ Department of Neurology, Erasmus University Medical Centre, Rotterdam, the Netherlands

^240^ Department of Clinical Genetics, Erasmus MC, Rotterdam, the Netherlands

^241^ Department of Radiology and Nuclear Medicine, Erasmus MC, Rotterdam, the Netherlands

^242^ Department of Psychology, Latin American Brain Health (BrainLat), Universidad Adolfo Ibáñez, Santiago, Chile

^243^ Norwegian National Advisory Unit on Ageing and Health, Vestfold Hospital Trust, Tønsberg, Norway

^244^ Department of Geriatric Medicine, Oslo University Hospital, Oslo, Norway

^245^ HUNT Research Center, Department of Public Health and Nursing, Faculty of Medicine and Health Sciences, Norwegian University of Science and Technology (NTNU), Trondheim, Norway

^246^ Department of Research, Innovation and Education, St. Olavs Hospital, Trondheim University Hospital, Trondheim, Norway

^247^ Division of Mental Health and Addiction, Oslo University Hospital, Oslo, Norway

^248^ NORMENT, University of Oslo, Oslo, Norway

^249^ Division of Neurogenetics and Molecular Psychiatry, Department of Psychiatry and Psychotherapy, Faculty of Medicine and University Hospital Cologne, University of Cologne, Cologne, Germany

^250^ Department of Neurodegenerative diseases and Geriatric Psychiatry, University Hospital Bonn, Medical Faculty, Bonn, Germany

^251^ Department of Psychiatry & Glenn Biggs Institute for Alzheimer's and Neurodegenerative Diseases, San Antonio, TX, USA

^252^ Alzheimer Center Amsterdam, Department of Neurology, Amsterdam Neuroscience, Vrije Universiteit Amsterdam, Amsterdam UMC, Amsterdam, the Netherlands

^253^ CHU de Bordeaux, Department of Neurology, Institute for Neurodegenerative Diseasese, Bordeaux, France

^254^ Institute of Molecular Medicine, McGovern Medical School, The University of Texas Health Science Center at Houston, Houston, TX, USA

^255^ Department of Research, Innovation and Education, Division of Clinical Neuroscience, Oslo University Hospital, Oslo, Norway

^256^ Department of Neurology, Oslo University Hospital, Oslo, Norway

^257^ Translational Health Sciences, Bristol Medical School, University of Bristol, Bristol, UK

^258^ School of Public Health, Faculty of Social Welfare and Health Sciences, University of Haifa, Haifa, Israel

^259^ Framingham Heart Study, Framingham, MA, USA

^260^ Department of Neurology, University of Texas Health San Antonio, San Antonio, TX, USA

^261^ Department of Neurology, Boston University School of Medicine, Boston, MA, USA
