# Supplementary material for: A genome‐wide association meta‐analysis of all‐cause and vascular dementia
Source: Alzheimers Dement. 2024 Jul 24;20(9):5973–95. doi: 10.1002/alz.14115 (PMC11497727; doi:10.1002/alz.14115)
Supplement: Supplementary file 1 — Supporting Information [file ALZ-20-5973-s002.docx]

***A genome-wide association meta-analysis of all-cause and vascular dementia***

Bernard Fongang^1,2,3,*,#^, Muralidharan Sargurupremraj^1,3,*^, Xueqiu Jian^1,3^, Aniket Mishra^4^, Olivia Skrobot^5^, Itziar de Rojas^6,7^, Vincent Damotte^8^, Joshua C Bis^9^, Kang-Hsien Fan^10^, Erin Jacobsen^11^, Gloria Hoi-Yee Li^12^, Jingyun Yang^13^, Bizzarro Alessandra^14^, Lauria Alessandra^14^, Saima Hilal^15,16^, Joyce Chong^15^, Yuek Ling Chai^15^, M.J. Knol^17^, Maria Pina Concas^18^, Girotto Giorgia^18,19^, Moeen Riaz^20^, Chenglong Yu^20^, Alexander Guðjónsson^21^, Paul Lacaze^20^, Adam C Naj^22^, Monica Goss^1^, Yannick W. Ngouongo^1^, Aicha Soumare^4^, Vincent Bouteloup^4,23^, Vilmundur Guðnason^21^, Petronilla Battista^24^, Aurora Santin^19^, Beatrice Spedicati^19^, Rodolfo Sardone^25,26^, Lenore Launer^27^, Jan Bressler^28^, Rebecca F Gottesman^29^, Quentin Le Grand^30^, Ilana Caro^30^, Gennady V. Roshchupkin^17,31^, Hampton L. Leonard^32,33,34^, Chaojie Yang^35,36^, Traci M. Bartz^37,38^, Constance Bordes^30^, Paul M. Ridker^39,40^, Mirjam I. Geerlings^41^, Natalie C. Gasca^38^, Ani Manichaikul^35^, Mike A. Nalls^32,33,34^, Stephen S. Rich^35^, Carsten O. Schmidt^42^, Stella Trompet^43,44^, Marion van Vugt^45^, Hans J. Grabe^46,47^, J Wouter Jukema^44,48,49^, Ina L. Rissanen^41^, Sylvia Wassertheil-Smoller^50^, M. Arfan Ikram^17^, Eleanor M. Simonsick^51^, W T. Longstreth^52,53^, Daniel I. Chasman^39,40^, Jerome I. Rotter^54^, Naveed Sattar^55^, David J Stott^56^, Eric J Shiroma^57^, Sigurdur Sigurdsson^58^, Mohsen Ghanbari^17^, Ulf Schminke^59^, Eric Boerwinkle^60,61^, Hugo J Aparicio^62,63^, Alexa S Beiser^62,64^, Jose R Romero^62,63^, Vasileios Lioutas^62,65^, Ruiqi Wang^62,64^, Chloe Sarnowski^66^, Alexander Teumer^42,67^, Uwe Völker^67,68^, Thomas H. Mosley^69^, Marta Marquié^6,7^, Pablo García-González^6,7^, Clàudia Olivé^6^, Raquel Puerta^6^, Amanda Cano^6,7^, Oscar Sotolongo-Grau^6,7^, Sergi Valero^6,7^, Vanesa Veronica Pytel^6^, Maitée Rosende-Roca^6,7^, Montserrat Alegret^6,7^, Lluís Tàrraga^6,7^, Mercè Boada^6,7^, Ángel Carracedo^70,71^, Emilio Franco-Macías^7,72^, Gerard Piñol-Ripoll^73,74^, Guillermo Garcia-Ribas^75,76,77,78^, Jordi Pérez-Tur^7,78,78^, Jose Luís Royo^79^, Jose María García-Alberca^80^, Luis Miguel Real^81,82^, María Eugenia Sáez^83^, María J. Bullido^7,84,85,86^, Miguel Calero^7,87,88^, Miguel Medina^7,89^, Pablo Mir^7,90,91^, Pascual Sánchez-Juan^7,92^, Pau Pastor^93,94^, Victoria Álvarez^95,96^, Benjamin Grenier-Boley^8^, Fahri Küçükali^97,98,99^, Sven Van der Lee^100,101,102^, Oliver Peters^103,104^, Anja Schneider^105,106^, Martin Dichgans^107,108,109^, Dan Rujescu^110^, Deckert Jürgen^111^, Emrah Düzel^112,113^, Jens Wiltfang^114,115,116^, Susanne Moebus^117^, Michael Wagner^118,119^, Timo Grimmer^120^, Nikolaos Scarmeas^121,122^, Jordi Clarimon^7,123^, Fermin Moreno^7,124,125^, Raquel Sánchez-Valle^126^, Luis M Real^81,127^, Eloy Rodriguez-Rodriguez^7,128^, Adolfo Lopez de Munain^77,124,129^, Alexandre de Mendonça^130^, Jakub Hort^131,132^, Caroline Graff^133^, Goran Papenberg^134^, Vilmantas Giedraitis^135^, Børge G. Nordestgaard^136,137^, Hilkka Soininen^138^, Miia Kivipelto^139,140,141,142,143^, Annakaisa Haapasalo^144^, Gael Nicolas^145^, Florence Pasquier^146^, Olivier Hanon^147^, Edna Grünblatt^148,149,150^, Julius Popp^151,152,153^, Luisa Benussi^154^, Daniela Galimberti^155,156^, Beatrice Arosio^157,158^, Patrizia Mecocci^159^, Alessio Squassina^160^, Lucio Tremolizzo^161^, Innocenzo Rainero^162^, Gianfranco Spalletta^163^, Davide Seripa^164^, Julie Williams^165^, Philippe Amouyel^8^, Frank Jessen^105,166,167^, Tsolaki Magda^168^, Ruth Frikke-Schmidt^169,170^, Kristel Sleegers^97,98,99^, Sebastiaan Engelborghs^171,172^, Rik Vandenberghe^173,174^, Martin Ingelsson^135,175,176^, Giacomina Rossi^177^, Mikko Hiltunen^178^, Rebecca Sims^165^, Alla Graban^179^, Anna Bochynska^179^, Magdalena Gugała-Iwaniuk^179^, Danuta Ryglewicz^179^, Hanna Wehr^179^, Joyce Ruifen Chong^15^, Mitchell KP Lai^15^, Venketasubramanian N^180^, Boon-Yeow Tan^181^, Angelo Baldassare Cefalù^182^, Rossella Spina^182^, Robin Guariglia^183^, Patrizia Bastiani^184^, Nicola J Armstrong^185^, Roberta Baschi^186,187^, Malgorzata Bednarska-Makaruk^188^, Regis bordet ^189,190^, Anne-Marie Bordet^189,190^, Henry Brodaty^191^, Roberta Cecchetti^192^, Srdjan Djurovic^193,194^, Grazia D’Onofrio^195^, Timo Erkinjuntti^196^, Margaret Esiri^197^, Patrick Gelé^189,190^, Catharine Joachim^198^, Teresa Juarez-Cedillo^199^, Raj Kalaria^200,201^, Pekka Karhunen^202^, Jan LACZO^131,132^, Ondrej LERCH^131,132^, Carlo Masullo^203^, Karen A Mather^191,204^, Vaclav MATOSKA^205^, Susanna Melkas^206,207^, Roberto Monastero^186,187^, Katya Numbers^191^, Francesco Panza^208,209,210^, Tuomo M Polvikoski^200,201^, Joe Quinn^183^, Arvid Rongve^211,212^, Perminder S Sachdev^191,213^, Michela Scamosci^192^, Anbupalam Thalamuthu^191^, Anne Tybjærg-Hansen^214^, Martin VYHNALEK^131,132^, Shawn K. Westaway^215^, Amy E Martinsen^216,217,218^, Anne Heidi Skogholt^218^, Cristen J Willer^219^, Eystein Stordal^220,221^, Geir Bråthen^222^, Jonas Bille Nielsen^218,219^, Lars G Fritsche^223^, Laurent F Thomas^218,224,225,226^, Linda M Pedersen^216^, Maiken E Gabrielsen^218^, Ole Kristian Drange^220,227^, Sigrid Botne Sando^218,222,228,229^, Tore Wergeland Meisingset^222,228^, Genevieve Chene^4,23^, Wei Zhou^230,231^, Christophe Tzourio^4,232^, Adrienne Tin^69^, Oscar L Lopez^233^, Haan Mary^234^, Sigrid Børte^217,218,229^, Ingunn Bosnes^220,221^, Mary Ganguli^10,11^, Ching-Lung Cheung^12^, David A Bennett^13^, Christopher Chen^15^, M. Ilyas Kamboh^10^, Claudia Satizabal^1,3^, M. Kamran Ikram^17,235^, Hieab Adams^236,237,238^, Yang Qiong^64^, Gerard D. Schellenberg^22^, Geir Selbæk^217,228,239,240^, Kristian Hveem^218,241,242^, Ole A Andreassen^243,244^, Alfredo Ramirez^105,245,246,247^, Carole Dufouil^4,23^, Wiesje van der Flier^248^, John-Anker Zwart^216,217,218^, Stéphanie Debette^4,249^, Myriam Fornage^28,250^, Bendik Winsvold^218,251,252^, Jean-Charles Lambert^8^, Agustin Ruiz^6,7^, Patrick G. Kehoe^253^, Galit Weinstein^254,#^, and Sudha Seshadri^1,255,256,257,#^, for the Cohorts for Heart and Aging Research in Genomic Epidemiology (CHARGE)

^1^ Glenn Biggs Institute for Alzheimer’s & Neurodegenerative Diseases, University of Texas Health Science Center, San Antonio, TX, USA
^2^ Department of Biochemistry and Structural Biology, University of Texas Health Science Center, San Antonio, TX, USA
^3^ Department of Population Health Sciences, University of Texas Health Science Center, San Antonio, TX, USA
^4^ University of Bordeaux, Inserm, Bordeaux Population Health Research Center, UMR 1219, F-33000 Bordeaux, France
^5^ Population Health Sciences, Bristol Medical School, University of Bristol, Bristol, UK
^6^ Research Center and Memory Clinic, ACE Alzheimer Center Barcelona. Universitat Internacional de Catalunya, Spain
^7^ Networking Research Center on Neurodegenerative Diseases (CIBERNED), Instituto de Salud Carlos III, Madrid, Spain
^8^ Univ. Lille, Inserm, CHU Lille, Institut Pasteur de Lille, U1167-RID-AGE facteurs de risque et déterminants moléculaires des maladies liés au vieillissement, Lille, France
^9^ Cardiovascular Health Research Unit, Department of Medicine, University of Washington, Seattle, Washington
^10^ Department of Human Genetics, School of Public Health, University of Pittsburgh, Pittsburgh, PA, USA
^11^ Department of Psychiatry and Neurology, School of Medicine, University of Pittsburgh, Pittsburgh, PA, USA
^12^ Department of Pharmacology and Pharmacy, Centre for Genomic Sciences, The University of Hong Kong, Hong Kong
^13^ Rush Alzheimer’s Disease Center and Department of Neurological Sciences, Rush University Medical Center, Chicago, IL, USA
^14^ GERIATRICS UNIT, FONDAZIONE POLICLINICO UNIVERSITARIO A. GEMELLI IRCCS, LARGO A GEMELLI, 8 -00168 ROMA- ITALY
^15^ Department of Pharmacology, National University of Singapore, Singapore
^16^ Saw Swee Hock School of Public Health, National University of Singapore and National University Health System, Singapore
^17^ Department of Epidemiology, Erasmus MC, University Medical Center, Rotterdam, the Netherlands
^18^ Institute for Maternal and Child Health, IRCCS Burlo Garofolo, 34127 Trieste, Italy
^19^ Department of Medicine, Surgery and Health Sciences, University of Trieste, 34139 Trieste, Italy
^20^ Department of Epidemiology and Preventive Medicine, Monash University, Melbourne, VIC, Australia
^21^ Faculty of Medicine, University of Iceland, Reykjavik, Iceland
^22^ Department of Biostatistics and Epidemiology/Center for Clinical Epidemiology and Biostatistics, University of Pennsylvania Perelman School of Medicine, Philadelphia, PA, USA
^23^ Pôle de Santé Publique Centre Hospitalier Universitaire (CHU) de Bordeaux, Bordeaux, France
^24^ Istituti Clinici Scientifici Maugeri, Pavia. IRCCS di Bari, Bari, Italy
^25^ Department of Translational Biomedicine and Neuroscience, University of Bari “Aldo Moro”
^26^ Unit of Methodology and Data Sciences for Population Health, National Institute IRCCS Saverio de Bellis Research Hospital, Castellana Grotte, Italy
^27^ Laboratory of Epidemiology and Population Sciences, Intramural Research Program, National Institute of Aging, National Institutes of Health, Bethesda, MD, USA
^28^ Human Genetics Center, School of Public Health, The University of Texas Health Science Center at Houston, Houston, TX, USA
^29^ Stroke Branch, National Institute of Neurological Disorders and Stroke Intramural Program, National Institutes of Health, Bethesda, MD
^30^ University of Bordeaux, Inserm, Bordeaux Population Health Research Center, team ELEANOR, UMR 1219, F-33000 Bordeaux, France
^31^ Department of Radiology and Nuclear Medicine, Erasmus MC University Medical Center
^32^ Center for Alzheimer’s and Related Dementias, National Institutes of Health, Bethesda, USA
^33^ Laboratory of Neurogenetics, National Institute on Aging, National Institutes of Health, Bethesda USA
^34^ Data Tecnica International LLC, Glen Echo, USA
^35^ Center for Public Health Genomics, University of Virginia, Charlottesville, VA, USA
^36^ Department of Biochemistry and Molecular Genetics, University of Virginia, Charlottesville, VA, USADepartment of Biochemistry and Molecular Genetics, University of Virginia, Charlottesville, VA, USA
^37^ Cardiovascular Health Research Unit, Department of Medicine, University of Washington, Seattle, WA, USA
^38^ Department of Biostatistics, University of Washington, Seattle, WA, USA
^39^ Division of Preventive Medicine, Brigham and Women’s Hospital, Boston, MA 02215, USA
^40^ Harvard Medical School, Boston, MA 02115, USA
^41^ Julius Center for Health Sciences and Primary Care, University Medical Center Utrecht, Utrecht University, Utrecht, the Netherlands
^42^ University Medicine Greifswald, Institute for Community Medicine, SHIP/KEF, Germany
^43^ Department of Internal Medicine, Section of Gerontology and Geriatrics, Leiden University Medical Center, Leiden, the Netherlands
^44^ Department of Cardiology, Leiden University Medical Center, Leiden, the Netherlands
^45^ Division Heart & Lungs, Department of Cardiology, University Medical Center Utrecht, Utrecht University, Utrecht, The Netherlands
^46^ Department of Psychiatry and Psychotherapy, University Medicine Greifswald, Germany
^47^ German Center for Neurodegenerative Diseases (DZNE), Site Rostock/ Greifswald, Rostock, Germany
^48^ Netherlands Heart Institute, Utrecht, the Netherlands
^49^ Einthoven Laboratory for Experimental Vascular Medicine, LUMC, Leiden, the Netherlands
^50^ Department of Epidemiology and Population Health, Albert Einstein College of Medicine, New York, NY, USA
^51^ Longitudinal Studies Section, Translational Gerontology Branch, National Institute on Aging, Baltimore, Maryland, USA
^52^ Department of Epidemiology, University of Washington, Seattle, WA, USA
^53^ Department of Neurology, University of Washington, Seattle, Washington, USA
^54^ The Institute for Translational Genomics and Population Sciences, Department of Pediatrics, The Lundquist Institute for Biomedical Innovation at Harbor-UCLA Medical Center, CA, USA
^55^ BHF Glasgow Cardiovascular Research Centre, Faculty of Medicine, Glasgow, UK
^56^ Institute of Cardiovascular and Medical Sciences, College of Medical, Veterinary and Life Sciences, University of Glasgow, UK
^57^ Laboratory of Epidemiology and Population Sciences - National Institute of Health
^58^ Icelandic Heart Association, Kopavogur, Iceland
^59^ University Medicine Greifswald, Department of Neurology, Greifswald, Germany
^60^ Human Genetics Center, School of Public Health, University of Texas Health Science Center at Houston, Houston, TX, USA
^61^ Human Genome Sequencing Center, Baylor College of Medicine, Houston, TX, USA
^62^ Framingham Heart Study, Framingham, MA, USA
^63^ Department of Neurology, Boston University School of Medicine, Boston, MA 2115, USA
^64^ Department of Biostatistics, Boston University School of Public Health, Boston, MA, USA
^65^ Department of Neurology, Beth Israel Deaconess Medical Center, Boston, MA, USA
^66^ Department of Epidemiology, Human Genetics and Environmental Sciences, University of Texas Health Science Center at Houston, School of Public Health, Houston, TX
^67^ DZHK (German Centre for Cardiovascular Research), Partner Site Greifswald, Greifswald, Germany
^68^ Interfaculty Institute for Genetics and Functional Genomics, University Medicine Greifswald, Greifswald, Germany
^69^ Memory Impairment and Neurodegenerative Dementia (MIND) Center and Department of Medicine, University of Mississippi Medical Center, Jackson, MS
^70^ Grupo de Medicina Xenómica, CIBERER, CIMUS. Universidade de Santiago de Compostela, Santiago de Compostela, Spain
^71^ Fundación Pública Galega de Medicina Xenómica- IDIS, Santiago de Compostela, Spain
^72^ Unidad de Demencias, Servicio de Neurología y Neurofisiología. Instituto de Biomedicina de Sevilla (IBiS), Hospital Universitario Virgen del Rocío/CSIC/Universidad de Sevilla, Seville, Spain
^73^ Unitat Trastorns Cognitius, Hospital Universitari Santa Maria de Lleida, Lleida, Spain
^74^ Institut de Recerca Biomedica de Lleida (IRBLLeida), Lleida, Spain
^75^ Hospital Universitario Ramon y Cajal, IRYCIS, Madrid, Spain
^76^ Unitat de Genètica Molecular, Institut de Biomedicina de València-CSIC, Valencia, Spain
^77^ CIBERNED, Network Center for Biomedical Research in Neurodegenerative Diseases, National Institute of Health Carlos III, Madrid, Spain
^78^ Unidad Mixta de Neurologia Genètica, Instituto de Investigación Sanitaria La Fe, Valencia, Spain
^79^ Departamento de Especialidades Quirúrgicas, Bioquímica e Inmunología. School of Medicine. University of Malaga. Málaga, Spain
^80^ Alzheimer Research Center & Memory Clinic, Instituto Andaluz de Neurociencia, Málaga, Spain
^81^ Unidad Clínica de Enfermedades Infecciosas y Microbiología. Hospital Universitario de Valme, Sevilla, Spain
^82^ Departamento de Especialidades Quirúrgicas, Bioquímica e Inmunología. Facultad de Medicina. Universidad de Málaga. Málaga, Spain
^83^ CAEBI, Centro Andaluz de Estudios Bioinformáticos, Sevilla, Spain
^84^ Centro de Biología Molecular Severo Ochoa (UAM-CSIC)
^85^ Instituto de Investigacion Sanitaria ‘Hospital la Paz’ (IdIPaz), Madrid, Spain
^86^ Universidad Autónoma de Madrid
^87^ CIEN Foundation/Queen Sofia Foundation Alzheimer Center/Instituto de Salud Carlos III
^88^ UFIEC, Instituto de Salud Carlos III
^89^ CIEN Foundation/Queen Sofia Foundation Alzheimer Center
^90^ Unidad de Trastornos del Movimiento, Servicio de Neurología y Neurofisiología. Instituto de Biomedicina de Sevilla (IBiS), Hospital Universitario Virgen del Rocío/CSIC/Universidad de Sevilla, Seville, Spain
^91^ Departamento de Medicina, Facultad de Medicina, Universidad de Sevilla, Seville, Spain
^92^ Alzheimer’s Centre Reina Sofia-CIEN Foundation, Centro de Investigación Biomédica en Red sobre Enfermedades Neurodegenerativas (CIBERNED), Madrid, Spain
^93^ Unit of Neurodegenerative Diseases, Department of Neurology, Hospital Germans Trias i Pujol, Badalona, Barcelona, Spain
^94^ Neurodegenerative Diseases Research Laboratory, Germans Trias i Pujol Research Laboratory, Badalona, Barcelona, Spain
^95^ Laboratorio de Genética. Hospital Universitario Central de Asturias, Oviedo, Spain
^96^ Instituto de Investigación Sanitaria del Principado de Asturias (ISPA)
^97^ Complex Genetics of Alzheimer’s Disease Group, VIB Center for Molecular Neurology, VIB, Antwerp, Belgium
^98^ Laboratory of Neurogenetics, Institute Born - Bunge, Antwerp, Belgium
^99^ Department of Biomedical Sciences, University of Antwerp, Neurodegenerative Brain Diseases Group, Center for Molecular Neurology, VIB, Antwerp, Belgium
^100^ Alzheimer Center Amsterdam, Neurology, Vrije Universiteit Amsterdam, Amsterdam UMC Location VUmc, Amsterdam, The Netherlands.
^101^ Amsterdam Neuroscience, Neurodegeneration, Amsterdam, The Netherlands
^102^ Section Genomics of Neurodegenerative Diseases and Aging, Human Genetics, Vrije Universiteit Amsterdam, Amsterdam UMC location VUmc, Amsterdam, The Netherlands
^103^ German Center for Neurodegenerative Diseases (DZNE), Berlin, Germany
^104^ Charité – Universitätsmedizin Berlin, corporate member of Freie Universität Berlin, Humboldt-Universität zu Berlin, and Berlin Institute of Health, Institute of Psychiatry and Psychotherapy, Hindenburgdamm 30, 12203 Berlin, Germany
^105^ German Center for Neurodegenerative Diseases (DZNE), Bonn, Germany
^106^ Department for Neurodegenerative Diseases and Geriatric Psychiatry, University Hospital Bonn, Venusberg-Campus 1, 53127 Bonn, Germany
^107^ Institute for Stroke and Dementia Research (ISD), University Hospital, LMU Munich, Munich, Germany.
^108^ German Center for Neurodegenerative Diseases (DZNE), Munich, Germany
^109^ Munich Cluster for Systems Neurology (SyNergy), Munich, Germany
^110^ Martin-Luther-University Halle-Wittenberg, University Clinic and Outpatient Clinic for Psychiatry, Psychotherapy and Psychosomatics, Halle (Saale), Germany
^111^ Department of Psychiatry, Psychosomatics and Psychotherapy, Center of Mental Health, University Hospital of Würzburg, Germany
^112^ German Center for Neurodegenerative Diseases (DZNE), Magdeburg, Germany
^113^ Institute of Cognitive Neurology and Dementia Research (IKND), Otto-von-Guericke University, Magdeburg, Germany
^114^ Department of Psychiatry and Psychotherapy, University Medical Center Goettingen, Goettingen, Germany
^115^ German Center for Neurodegenerative Diseases (DZNE), Goettingen, Germany
^116^ Medical Science Department, iBiMED, Aveiro, Portugal
^117^ Institute for Urban Public Health, University Hospital of University Duisburg-Essen, Essen, Germany.
^118^ Department of Neurodegenerative Diseases and Geriatric Psychiatry, University of Bonn, 53127 Bonn, Germany.
^119^ German Center for Neurodegenerative Diseases (DZNE), 53127 Bonn, Germany.
^120^ Technical University of Munich, School of Medicine, Klinikum rechts der Isar, Department of Psychiatry and Psychotherapy
^121^ Taub Institute for Research in Alzheimer’s Disease and the Aging Brain, The Gertrude H. Sergievsky Center, Department of Neurology, Columbia University, New York, NY
^122^ 1st Department of Neurology, Aiginition Hospital, National and Kapodistrian University of Athens, Medical School, Greece
^123^ Department of Neurology, II B Sant Pau, Hospital de la Santa Creu i Sant Pau, Universitat Autònoma de Barcelona, Barcelona, Spain.
^124^ Department of Neurology. Hospital Universitario Donostia. San Sebastian, Spain
^125^ Neurosciences Area. Instituto Biodonostia. San Sebastian, Spain
^126^ Alzheimer’s disease and other cognitive disorders unit. Service of Neurology. Hospital Clínic of Barcelona. Institut d’Investigacions Biomèdiques August Pi i Sunyer, University of Barcelona, Barcelona, Spain
^127^ Depatamento de Especialidades Quirúrgicas, Bioquímica e Inmunología. Facultad de Medicina. Universidad de Málaga. Málaga, Spain
^128^ Neurology Service, Marqués de Valdecilla University Hospital (University of Cantabria and IDIVAL), Santander, Spain.
^129^ Department of Neurosciences. Faculty of Medicine and Nursery. University of the Basque Country, San Sebastián, Spain
^130^ Faculty of Medicine, University of Lisbon, Portugal
^131^ Memory Clinic, Department of Neurology, Charles University, 2nd Faculty of Medicine and Motol University Hospital, Czech Republic
^132^ International Clinical Research Center, St. Anne’s University Hospital Brno, Brno, Czech Republic
^133^ Unit for Hereditary Dementias, Theme Aging, Karolinska University Hospital-Solna, 171 64 Stockholm Sweden
^134^ Aging Research Center, Department of Neurobiology, Care Sciences and Society, Karolinska Institutet and Stockholm University, Stockholm, Sweden
^135^ Dept.of Public Health and Caring Sciences / Geriatrics, Uppsala University, Sweden
^136^ Department of Clinical Biochemistry, Copenhagen University Hospital – Herlev Gentofte, Denmark
^137^ Department of Clinical Medicine, University of Copenhagen, Denmark
^138^ Institute of Clinical Medicine - Neurology, University of Eastern Finland, Finland
^139^ Division of Clinical Geriatrics, Center for Alzheimer Research, Care Sciences and Society (NVS)
^140^ Karolinska Institutet, Stockholm, Sweden
^141^ Institute of Public Health and Clinical Nutrition, University of Eastern Finland, Kuopio, Finland
^142^ Neuroepidemiology and Ageing Research Unit, School of Public Health, Imperial College London, London, United Kingdom
^143^ Stockholms Sjukhem, Research & Development Unit, Stockholm, Sweden
^144^ A.I Virtanen Institute for Molecular Sciences, University of Eastern Finland, Kuopio, Finland
^145^ Normandie Univ, UNIROUEN, Inserm U1245 and Rouen University Hospital, Department of Genetics and CNR-MAJ, F 76000, Normandy Center for Genomic and Personalized Medicine, Rouen, France
^146^ Univ Lille Inserm 1171, CHU Clinical and Research Memory Research Centre (CMRR) of Distalz Lille France.
^147^ Université de Paris, EA 4468, APHP, Hôpital Broca, Paris, France
^148^ Department of Child and Adolescent Psychiatry and Psychotherapy, University Hospital of Psychiatry Zurich, University of Zurich, Zurich, Switzerland
^149^ Neuroscience Center Zurich, University of Zurich and ETH Zurich, Switzerland
^150^ Zurich Center for Integrative Human Physiology, University of Zurich, Switzerland
^151^ Old Age Psychiatry, Department of Psychiatry, Lausanne University Hospital, Lausanne, Switzerland
^152^ Department of Geriatric Psychiatry, University Hospital of Psychiatry Zürich, Zürich, Switzerland
^153^ Institute for Regenerative Medicine, University of Zürich, Switzerland
^154^ Molecular Markers Laboratory, IRCCS Istituto Centro San Giovanni di Dio Fatebenefratelli, Brescia
^155^ Neurodegenerative Diseases Unit, Fondazione IRCCS Ca’ Granda, Ospedale Policlinico, Milan, IT
^156^ Dept. of Biomedical, Surgical and Dental Sciences, University of Milan, Milan, IT
^157^ Department of Clinical Sciences and Community Health, University of Milan, 20122 Milan, Italy
^158^ Geriatric Unit, Fondazione IRCCS Ca’ Granda Ospedale Maggiore Policlinico, 20122 Milan, Italy
^159^ Institute of Gerontology and Geriatrics, Department of Medicine and Surgery, University of Perugia, Italy
^160^ Department of Biomedical Sciences, University of Cagliari, Italy.
^161^ Neurology, “San Gerardo” Hospital, Monza and University of Milano-Bicocca, Italy
^162^ Department of Neuroscience “Rita Levi Montalcini”, University of Torino, Torino, Italy
^163^ Laboratory of Neuropsychiatry, IRCCS Santa Lucia Foundation
^164^ Department of Hematology and Stem Cell Transplant, Vito Fazzi Hospital, Lecce, Italy
^165^ Division of Psychological Medicine and Clinical Neuroscience, School of Medicine, Cardiff University, Wales, UK
^166^ Department of Psychiatry and Psychotherapy, Faculty of Medicine and University Hospital Cologne, University of Cologne, Cologne, Germany
^167^ Cluster of Excellence Cellular Stress Responses in Aging-associated Diseases (CECAD), University of Cologne, Cologne, Germany
^168^ 1st Department of Neurology, Medical School, Aristotle University of Thessaloniki, Thessaloniki, Makedonia, Greece
^169^ Department of Clinical Biochemistry, Copenhagen University Hospital - Rigshospitalet, Copenhagen, Denmark
^170^ Department of Clinical Medicine, University of Copenhagen, Copenhagen, Denmark
^171^ Center for Neurosciences, Vrije Universiteit Brussel (VUB), Brussels, Belgium.
^172^ Reference Center for Biological Markers of Dementia (BIODEM), Institute Born-Bunge, University of Antwerp, Antwerp, Belgium.
^173^ Laboratory for Cognitive Neurology, Department of Neurosciences, University of Leuven, Leuven, Belgium.
^174^ Neurology Department, University Hospitals Leuven, Leuven, Belgium
^175^ Krembil Brain Institute, University Health Network, Toronto, Canada
^176^ Dept. of Medicine and Tanz Centre for Research in Neurodegenerative Diseases, University of Toronto, Canada
^177^ Fondazione IRCCS Istituto Neurologico Carlo Besta, Milan, Italy
^178^ Institute of Biomedicine, University of Eastern Finland, Finland
^179^ Institute of Psychiatry and Neurology, First Department of Neurology, Warsaw, Poland
^180^ Raffles Neuroscience Center, Raffles Hospital, Singapore
^181^ St Luke’s Hospital, Singapore, Singapore
^182^ Department of Health Promotion Sciences, Maternal and Infant Care (PROMISE), University of Palermo, Palermo, Italy
^183^ Department of Neurology, OHSU
^184^ Institute of Gerontology and Geriatrics, Department of Medicine, University of Perugia Perugia, Italy
^185^ Department of Mathematics and Statistics, Curtin University, Perth, Australia
^186^ Department of Biomedicine, Neuroscience and Advanced Diagnostics (BIND), University of Palermo, Palermo, Italy
^187^ Dementia and Parkinson’s Disease Center, University Hospital, “Paolo Giaccone”, Palermo, Italy
^188^ Institute of Psychiatry and Neurology, Department of Genetics, Warsaw, Poland
^189^ Univ Lille, Inserm, CHU Lille, France 
^190^ Lille Neuroscience & Cognition
^191^ Centre for Healthy Brain Ageing, Discipline of Psychiatry & Mental Health, School of Clinical Medicine, Faculty of Medicine and Health, University of New South Wales, Sydney, Australia
^192^ Institute of Gerontology and Geriatrics, Department of Medicine, University of Perugia Perugia (Italy)
^193^ NORMENT Centre, University of Bergen, Bergen, Norway.
^194^ Dept of Medical Genetics, Oslo University Hospital, Oslo, Norway
^195^ Clinical Psychology Service, Health Department, Fondazione IRCCS Casa Sollievo della Sofferenza, San Giovanni Rotondo (FG), Italy.
^196^ Clinical Neurosciences, Neurology, University of Helsinki and
^197^ Nuffield Department of Clinical Neurosciences, Oxford University
^198^ University of Oxford, Oxford UK
^199^ Unidad de Investigación en Epidemiología y Servicos de Salud Área Envejecimiento, Centro Medico Nacional Siglo XXI, Instituto Mexicano del Seguro Social. Ciudad de Mexico.
^200^ Translational and Clinical Research Institute, Newcastle University
^201^ Campus for Ageing and Vitality, Newcastle upon Tyne NE4 5PL, United Kingdom
^202^ Faculty of Medicine and Health Technology, Tampere University, and Department of Clinical Chemistry, Fimlab Laboratories. Tampere, Finland
^203^ INSTITUTE OF NEUROLOGY, CATHOLIC UNIVERSITY OF THE SACRED HEART, SCHOOL OF MEDICINE, LARGO A GEMELLI, 8 - 00168 ROMA - ITALY
^204^ Neuroscience Research Australia, Sydney, Australia
^205^ Department of Clinical Biochemistry, Hematology and Immunology, Na Homolce Hospital, Prague, Czech Republic
^206^ Helsinki University Hospital
^207^ University of Helsinki
^208^ Neurodegenerative Disease Unit, Department of Basic Medicine, Neuroscience, and Sense Organs, University of Bari Aldo Moro, Policlinico, Piazza Giulio Cesare 11, 70124 Bari, Italy
^209^ Geriatric Unit & Laboratory of Gerontology and Geriatrics, Department of Medical Sciences, IRCCS “Casa Sollievo della Sofferenza”, San Giovanni Rotondo, Viale Cappuccini 1, 71013 San Giovanni Rotondo, Foggia, Italy
^210^ Unit of Research Methodology and Data Sciences for Population Health, National Institute of Gastroenterology Saverio de Bellis, Research Hospital, Castellana Grotte, Bari, Italy
^211^ Department of Research and Innovation, Helse Fonna, Haugesund, Norway.
^212^ Department of Clinical Medicine (K1), University of Bergen, Bergen, Norway
^213^ Neuropsychiatric Institute, Euroa Centre, Prince of Wales Hospital, Sydney, Australia
^214^ Department of Clinical Biochemistry, Copenhagen University Hospital – Rigshospitalet, Copenhagen Denmark & Department of Clinical Medicine, Copenhagen Denmark.
^215^ Department of Neurology, Oregon Health & Science University
^216^ Department of Research and Innovation, Division of Clinical Neuroscience, Oslo University Hospital, Oslo, Norway
^217^ Institute of Clinical Medicine, Faculty of Medicine, University of Oslo, Oslo, Norway
^218^ K. G. Jebsen Center for Genetic Epidemiology, Department of Public Health and Nursing, Faculty of Medicine and Health Sciences, Norwegian University of Science and Technology (NTNU), Trondheim, Norway
^219^ Department of Internal Medicine, Division of Cardiovascular Medicine, University of Michigan, Ann Arbor, MI, 48109, USA
^220^ Department of Mental Health, Faculty of Medicine and Health Sciences, Norwegian University of Science and Technology (NTNU), Trondheim, Norway
^221^ Department of Psychiatry, Hospital Namsos, Nord-Trøndelag Health Trust, Namsos, Norway
^222^ Department of Neuromedicine and Movement Science, Faculty of Medicine and Health Sciences, Norwegian University of Science and Technology (NTNU), Trondheim, Norway
^223^ Center for Statistical Genetics, Department of Biostatistics, University of Michigan, Ann Arbor, MI, 48109, USA
^224^ Department of Clinical and Molecular Medicine, Norwegian University of Science and Technology (NTNU), Trondheim, Norway
^225^ BioCore - Bioinformatics Core Facility, Norwegian University of Science and Technology (NTNU), Trondheim, Norway
^226^ Clinic of Laboratory Medicine, St. Olavs Hospital, Trondheim University Hospital, Trondheim, Norway
^227^ Division of Mental Health Care, St. Olavs Hospital, Trondheim University Hospital, Trondheim, Norway
^228^ Department of Neurology and Clinical Neurophysiology, St. Olavs Hospital, Trondheim University Hospital, Trondheim, Norway
^229^ Research and Communication Unit for Musculoskeletal Health (FORMI), Department of Research and Innovation, Division of Clinical Neuroscience, Oslo University Hospital, Oslo, Norway
^230^ Department of Computational Medicine and Bioinformatics, University of Michigan, Ann Arbor, MI, 48109, USA
^231^ Analytic and Translational Genetics Unit, Massachusetts General Hospital, Boston, MA, USA
^232^ Bordeaux University Hospital, Department of Medical Informatics, F-33000 Bordeaux, France
^233^ Department of Neurology, School of Medicine, University of Pittsburgh, PA, USA
^234^ Department of Epidemiology & Biostatistics, University of California, San Francisco, California 94158, USA
^235^ Department of Neurology, Erasmus University Medical Centre, Rotterdam, Netherlands
^236^ Department of Clinical Genetics, Erasmus MC, Rotterdam, the Netherlands
^237^ Department of Radiology and Nuclear Medicine, Erasmus MC, Rotterdam, the Netherlands
^238^ Department of Psychology, Latin American Brain Health (BrainLat), Universidad Adolfo Ibáñez, Santiago, Chile
^239^ Norwegian National Advisory Unit on Ageing and Health, Vestfold Hospital Trust, Tønsberg, Norway
^240^ Department of Geriatric Medicine, Oslo University Hospital, Oslo, Norway
^241^ HUNT Research Center, Department of Public Health and Nursing, Faculty of Medicine and Health Sciences, Norwegian University of Science and Technology (NTNU), Trondheim, Norway
^242^ Department of Research, Innovation and Education, St. Olavs Hospital, Trondheim University Hospital, Trondheim, Norway
^243^ Division of Mental Health and Addiction, Oslo University Hospital, Oslo, Norway
^244^ NORMENT, University of Oslo, Oslo, Norway
^245^ Division of Neurogenetics and Molecular Psychiatry, Department of Psychiatry and Psychotherapy, Faculty of Medicine and University Hospital Cologne, University of Cologne, Cologne, Germany
^246^ Department of Neurodegenerative Diseases and Geriatric Psychiatry, University Hospital Bonn, Medical Faculty, Bonn, Germany
^247^ Department of Psychiatry & Glenn Biggs Institute for Alzheimer’s and Neurodegenerative Diseases, San Antonio, TX, USA
^248^ Alzheimer Center Amsterdam, Department of Neurology, Amsterdam Neuroscience, Vrije Universiteit Amsterdam, Amsterdam UMC, Amsterdam, The Netherlands
^249^ CHU de Bordeaux, Department of Neurology, Institute for Neurodegenerative Diseases, F-33000 Bordeaux, France
^250^ Institute of Molecular Medicine, McGovern Medical School,, The University of Texas Health Science Center at Houston, Houston TX, USA
^251^ Department of Research, Innovation and Education, Division of Clinical Neuroscience, Oslo University Hospital, Oslo, Norway
^252^ Department of Neurology, Oslo University Hospital, Oslo, Norway
^253^ Translational Health Sciences, Bristol Medical School, University of Bristol, Bristol, UK
^254^ School of Public Health, Faculty of Social Welfare and Health Sciences, University of Haifa, Haifa, Israel
^255^ Framingham Heart Study, MA, USA
^256^ Department of Neurology, UT Health San Antonio, 7703 Floyd Curl Drive, San Antonio, TX, USA
^257^ Department of Neurology, Boston University School of Medicine, Boston, Massachusetts, USA

* These authors contributed equally.

# corresponding authors:

Bernard Fongang: [fongang@uthscsa.edu](mailto:fongang@uthscsa.edu)

Galit Weinstein: gweinstei@univ.haifa.ac.il

Sudha Seshadri: [Seshadri@uthscsa.edu](mailto:Seshadri@uthscsa.edu)

**Supplementary Figures**

Table of Contents

[Introduction 11](#_Toc142840468)

[1 - Cross-ancestry meta-analysis: ancestry PCs plot 11](#_Toc142840469)

[2 – Variant plots 11](#_Toc142840470)

[2 – 1 All-cause dementia genome-wide significant variants 11](#_Toc142840471)

[2 – 1 – 1 APOE locus 12](#_Toc142840472)

[2 – 1 – 2 BIN1 locus 13](#_Toc142840473)

[2 – 1 – 3 MS4A6A locus 14](#_Toc142840474)

[2 – 1 – 4 PICALM locus 15](#_Toc142840475)

[2 – 1 – 5 CD2AP locus 16](#_Toc142840476)

[2 – 1 – 6 ACE locus 17](#_Toc142840477)

[2 – 1 – 7 CR1 locus 18](#_Toc142840478)

[2 – 1 – 8 PILRB locus 19](#_Toc142840479)

[2 – 1 – 9 ABCA7 locus 20](#_Toc142840480)

[2 – 1 – 10 SLC24A4 locus 21](#_Toc142840481)

[2 – 2 All-cause dementia suggestive variants 22](#_Toc142840482)

[2 – 2 – 1 ANO3 locus 22](#_Toc142840483)

[2 – 2 – 2 SEMA4D locus 23](#_Toc142840484)

[2 – 2 – 3 RBFOX1 locus 24](#_Toc142840485)

[2 – 2 – 4 TRIB1 locus 25](#_Toc142840486)

[2 – 2 – 5 HBEGF locus 26](#_Toc142840487)

[2 – 2 – 5 ZNF652 locus 27](#_Toc142840488)

[2 – 2 – 6 SREBF1 locus 28](#_Toc142840489)

[2 – 2 – 7 INPP5D locus 29](#_Toc142840490)

[2 – 3 Vascular dementia genome-wide significant variants 30](#_Toc142840491)

[2 – 3 – 1 APOE locus 30](#_Toc142840492)

[2 – 4 Vascular dementia suggestive variants 31](#_Toc142840493)

[**2 – 4 – 1 SPRY2 locus** 31](#_Toc142840494)

[2 – 4 – 2 SEMA6D locus 32](#_Toc142840495)

[2 – 4 – 3 SCARB1 locus 33](#_Toc142840496)

[**2 – 4 – 4 PSMA3 locus** 34](#_Toc142840497)

[**2 – 4 – 5 LINC02113 locus** 35](#_Toc142840498)

[**2 – 4 – 6 GIP locus** 36](#_Toc142840499)

[**2 – 4 – 7 DOK5 locus** 37](#_Toc142840500)

[**2 – 4 – 8 GALNT18 locus** 38](#_Toc142840501)

[**2 – 4 – 9 WAC locus** 39](#_Toc142840502)

[**2 – 4 – 10 ERBB4 locus** 40](#_Toc142840503)

[**2 – 4 – 11 PRKCE locus** 41](#_Toc142840504)

[**2 – 4 – 12 PHACTR3 locus** 42](#_Toc142840505)

[**2 – 4 – 13 AJAP1 locus** 43](#_Toc142840506)

[2 – 5 Cross-Ancestry meta-analysis plots 44](#_Toc142840507)

[3 – Other Figures 45](#_Toc142840508)

[**3 – 1 Variant Overlap with other complex traits** 45](#_Toc142840509)

[**3 – 2 Functional analysis Figures** 47](#_Toc142840510)

# Introduction

# 1 - Cross-ancestry meta-analysis: ancestry PCs plot


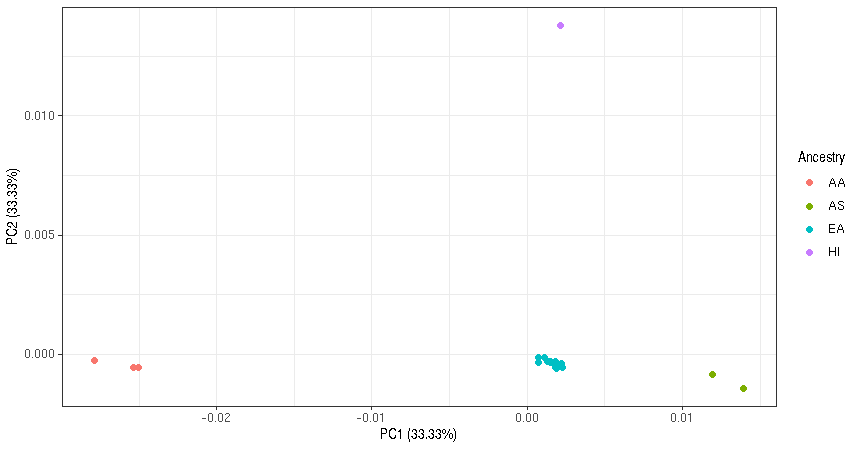


**Figure S1**: **Principal components generated using the mean allele frequency difference between studies.**Principal components (PCs) are generated using MR-MEGA from a matrix of mean pairwise allele frequency differences between cohorts (total n=). Color denotes self-reported ancestry for each cohort. Selected outliers are labeled with cohort names. Three PCs were chosen as per the author's recommendations and, as shown, are sufficient to separate self-reported ancestry groups.

# 2 – Variant plots

## 2 – 1 All-cause dementia genome-wide significant variants

### 2 – 1 – 1 APOE locus

**Figure S2: Regional association plot showing the genomic region containing APOE.** For each SNP, the P-value (log10 scale) of the association with ACD is represented (y-axis, left). The recombination rates (y-axis right), which reflect the local linkage disequilibrium structure, are also plotted.


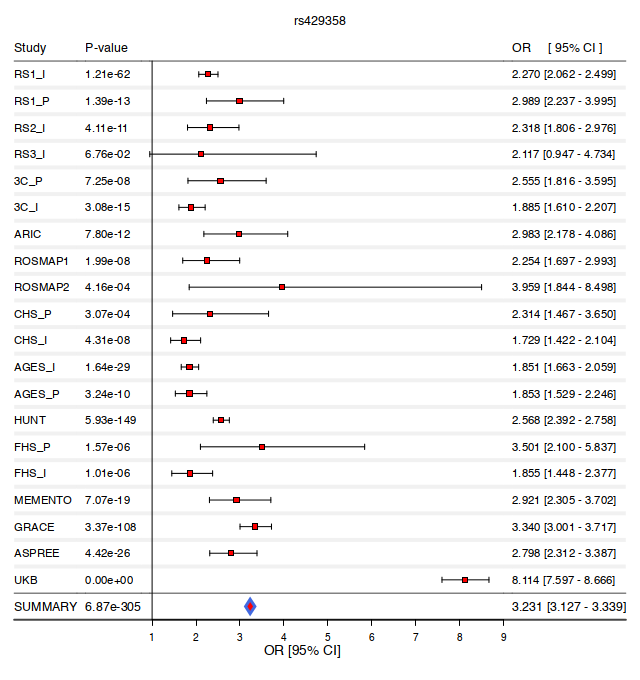


**Figure S3: Forest plot of the locus near APOE in ACD GWAS of European ancestry.**

### 2 – 1 – 2 BIN1 locus

**Figure S4: Regional association plot showing the genomic region containing BIN1.** For each SNP, the P-value (log10 scale) of the association with ACD is represented (y-axis, left). The recombination rates (y-axis right), which reflect the local linkage disequilibrium structure, are also plotted.


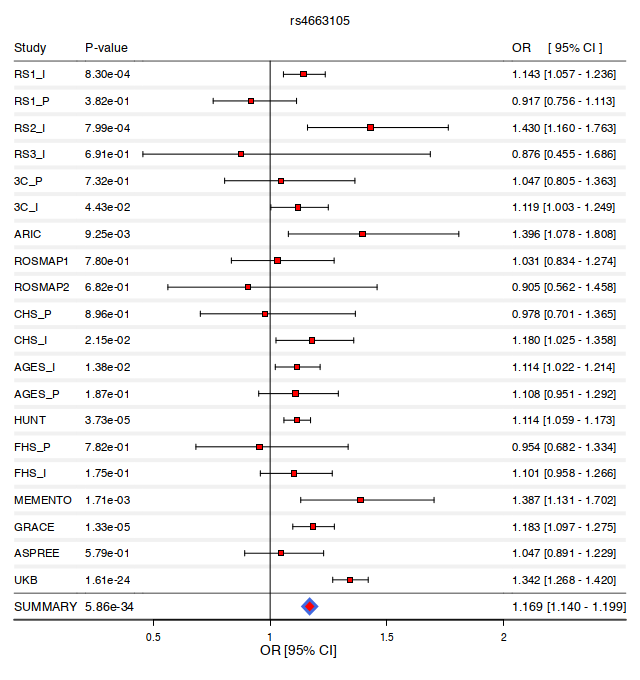


**Figure S5: Forest plot of the locus near BIN1 in ACD GWAS of European ancestry.**

### 2 – 1 – 3 MS4A6A locus

**Figure S6: Regional association plot showing the genomic region containing MS4A6A.** For each SNP, the P-value (log10 scale) of the association with ACD is represented (y-axis, left). The recombination rates (y-axis right), which reflect the local linkage disequilibrium structure, are also plotted.


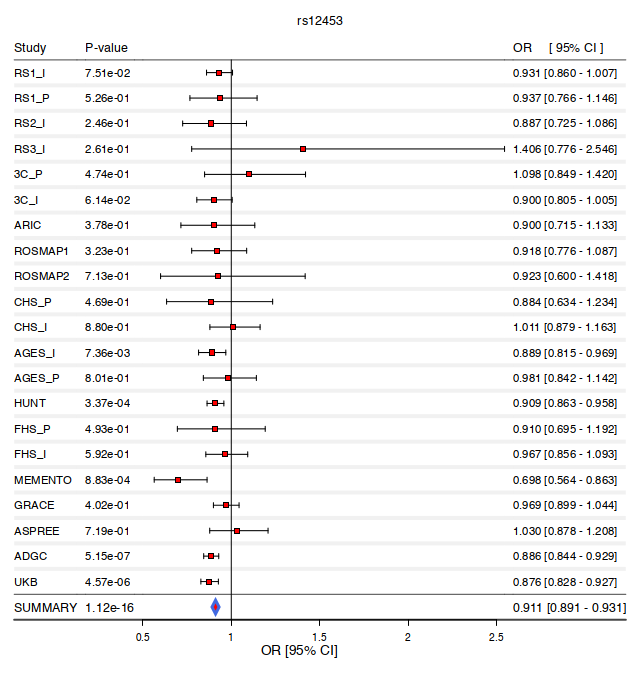


**Figure S7: Forest plot of the locus near MS4A6A in ACD GWAS of European ancestry.**

### 2 – 1 – 4 PICALM locus

**Figure S8: Regional association plot showing the genomic region containing PICALM.** For each SNP, the P-value (log10 scale) of the association with ACD is represented (y-axis, left). The recombination rates (y-axis right), which reflect the local linkage disequilibrium structure, are also plotted.


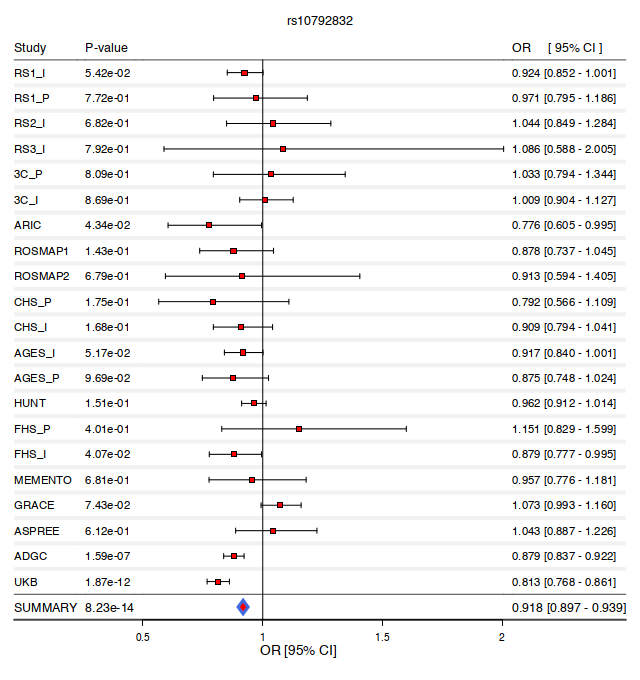


**Figure S9: Forest plot of the locus near PICALM in ACD GWAS of European ancestry.**

### 2 – 1 – 5 CD2AP locus

**Figure S10: Regional association plot showing the genomic region containing CD2AP.** For each SNP, the P-value (log10 scale) of the association with ACD is represented (y-axis, left). The recombination rates (y-axis right), which reflect the local linkage disequilibrium structure, are also plotted.


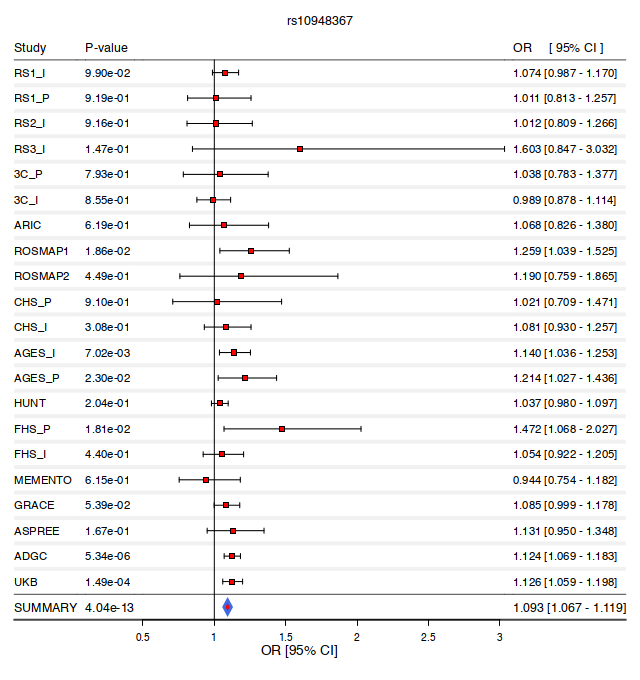


**Figure S11: Forest plot of the locus near CD2AP in ACD GWAS of European ancestry.**

### 2 – 1 – 6 ACE locus

**Figure S12: Regional association plot showing the genomic region containing ACE.** For each SNP, the P-value (log10 scale) of the association with ACD is represented (y-axis, left). The recombination rates (y-axis right), which reflect the local linkage disequilibrium structure, are also plotted.


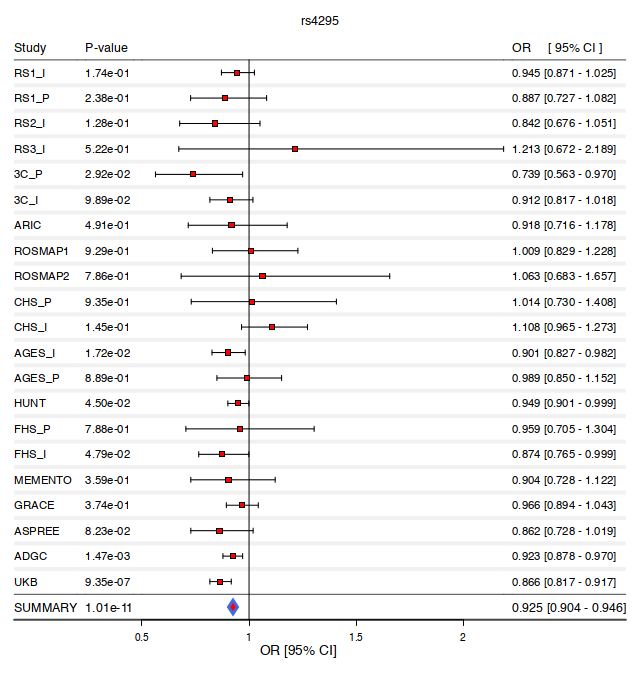


**Figure S13: Forest plot of the locus near ACE in ACD GWAS of European ancestry.**

### 2 – 1 – 7 CR1 locus

**Figure S14: Regional association plot showing the genomic region containing CR1.** For each SNP, the P-value (log10 scale) of the association with ACD is represented (y-axis, left). The recombination rates (y-axis right), which reflect the local linkage disequilibrium structure, are also plotted.


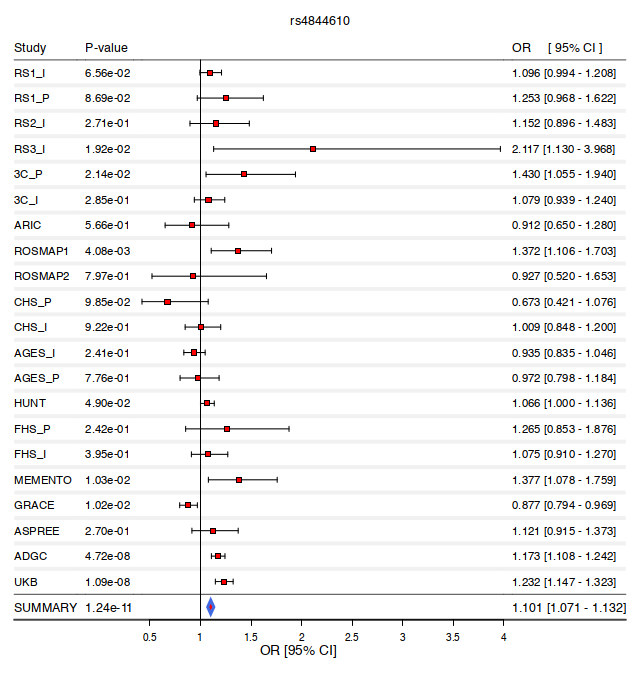


**Figure S15: Forest plot of the locus near CR1 in ACD GWAS of European ancestry.**

### 2 – 1 – 8 PILRB locus

**Figure S16: Regional association plot showing the genomic region containing PILRB.** For each SNP, the P-value (log10 scale) of the association with ACD is represented (y-axis, left). The recombination rates (y-axis right), which reflect the local linkage disequilibrium structure, are also plotted.


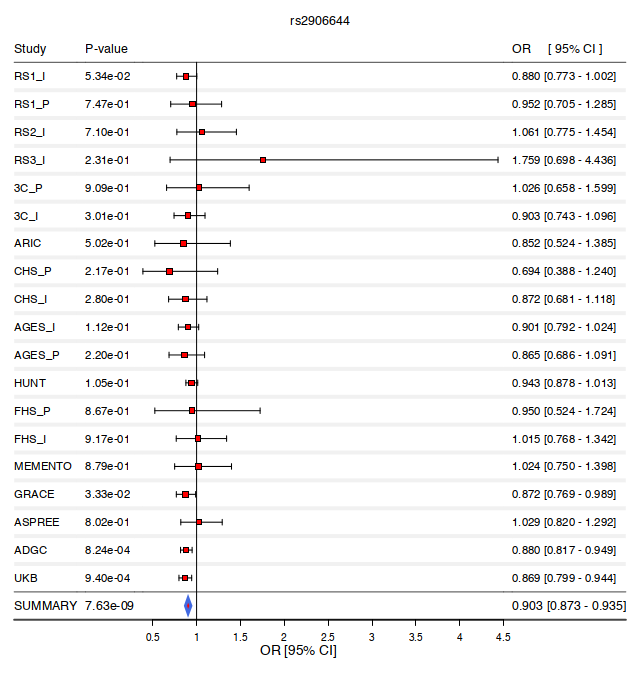


**Figure S17: Forest plot of the locus near PILRB in ACD GWAS of European ancestry.**

### 2 – 1 – 9 ABCA7 locus

**Figure S18: Regional association plot showing the genomic region containing ABCA7.** For each SNP, the P-value (log10 scale) of the association with ACD is represented (y-axis, left). The recombination rates (y-axis right), which reflect the local linkage disequilibrium structure, are also plotted.


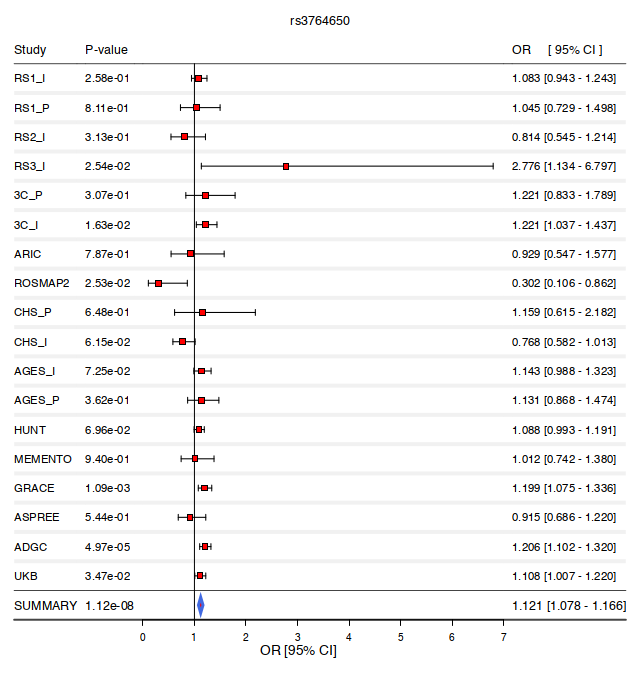


**Figure S19: Forest plot of the locus near ABCA7 in ACD GWAS of European ancestry.**

### 2 – 1 – 10 SLC24A4 locus

**Figure S20: Regional association plot showing the genomic region containing SLC24A4.** For each SNP, the P-value (log10 scale) of the association with ACD is represented (y-axis, left). The recombination rates (y-axis right), which reflect the local linkage disequilibrium structure, are also plotted.


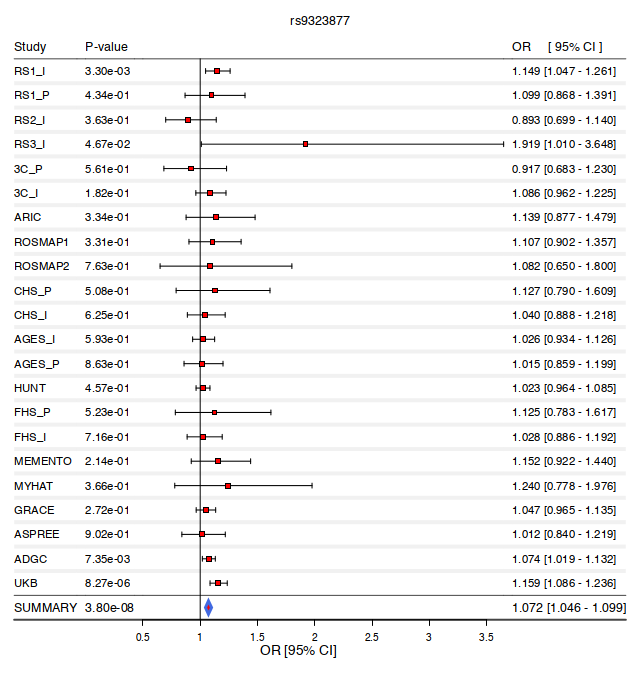


**Figure S21: Forest plot of the locus near SLC24A4 in ACD GWAS of European ancestry.**

## 2 – 2 All-cause dementia suggestive variants

### 2 – 2 – 1 ANO3 locus

**Figure S22: Regional association plot showing the genomic region containing ANO3.** For each SNP, the P-value (log10 scale) of the association with ACD is represented (y-axis, left). The recombination rates (y-axis right), which reflect the local linkage disequilibrium structure, are also plotted.

**
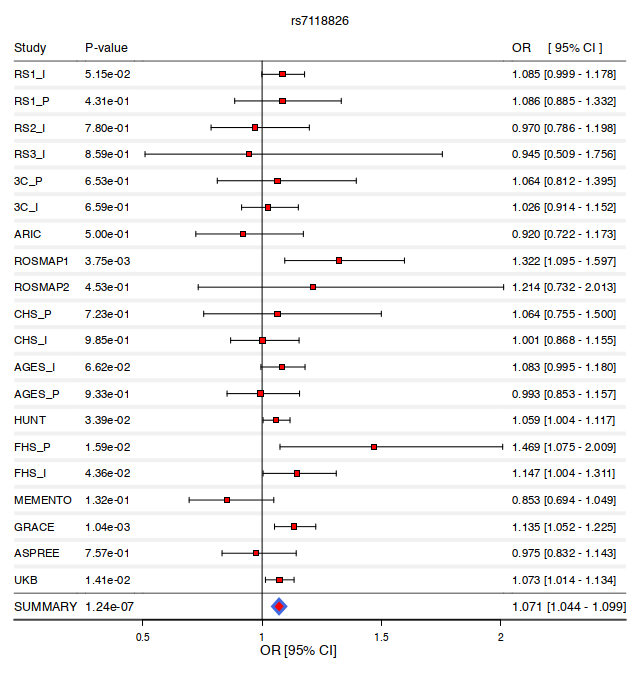
**

**Figure S23: Forest plot of the locus near ANO3 in ACD GWAS of European ancestry.**

### 2 – 2 – 2 SEMA4D locus

**Figure S24: Regional association plot showing the genomic region containing SEMA4D.** For each SNP, the P-value (log10 scale) of the association with ACD is represented (y-axis, left). The recombination rates (y-axis right), which reflect the local linkage disequilibrium structure, are also plotted.


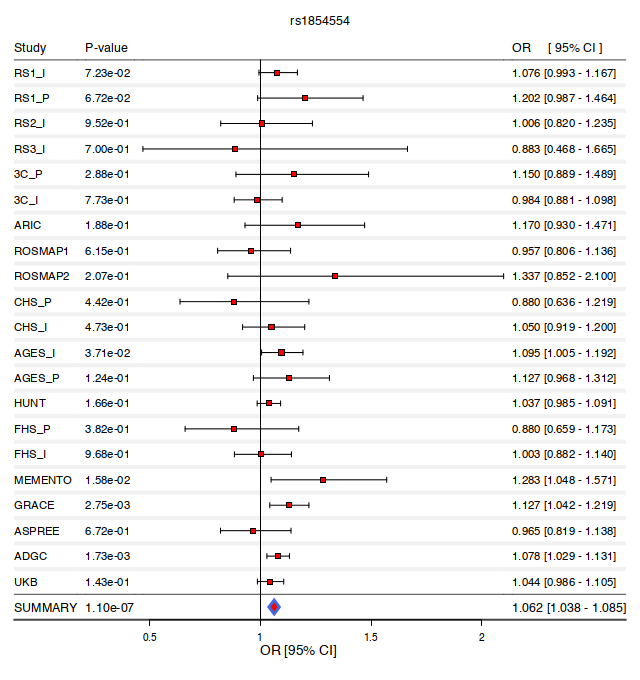


**Figure S25: Forest plot of the locus near SEMA4D in ACD GWAS of European ancestry.**

### 2 – 2 – 3 RBFOX1 locus

**Figure S26: Regional association plot showing the genomic region containing RBFOX1.** For each SNP, the P-value (log10 scale) of the association with ACD is represented (y-axis, left). The recombination rates (y-axis right), which reflect the local linkage disequilibrium structure, are also plotted.


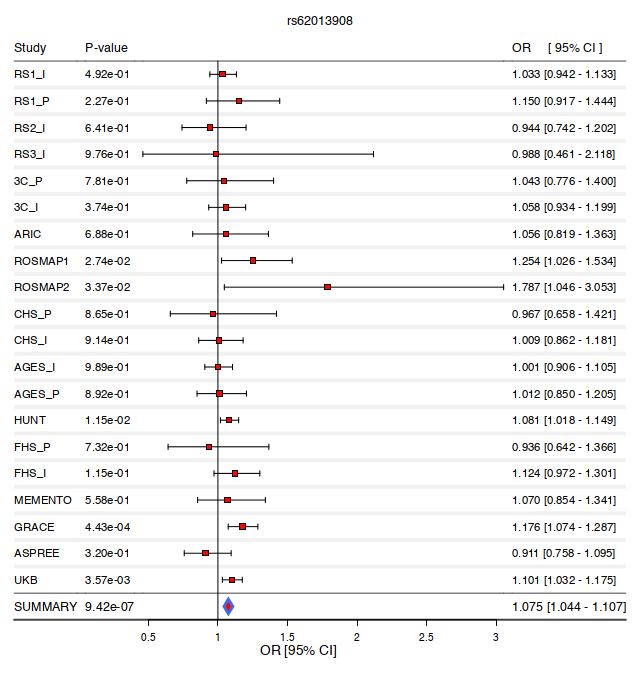


**Figure S27: Forest plot of the locus near RBFOX1 in ACD GWAS of European ancestry.**

### 2 – 2 – 4 TRIB1 locus

**Figure S28: Regional association plot showing the genomic region containing TRIB1.** For each SNP, the P-value (log10 scale) of the association with ACD is represented (y-axis, left). The recombination rates (y-axis right), which reflect the local linkage disequilibrium structure, are also plotted.


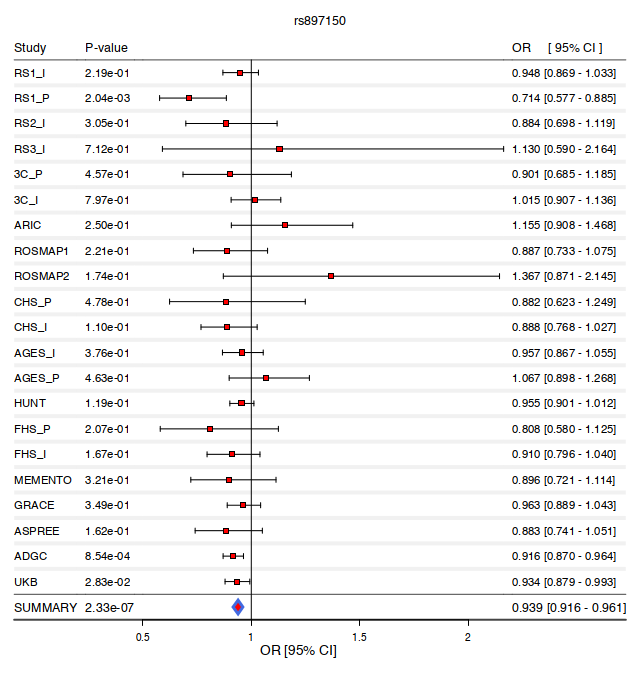


**Figure S29: Forest plot of the locus near TRIB1 in ACD GWAS of European ancestry.**

### 2 – 2 – 5 HBEGF locus

**Figure S30: Regional association plot showing the genomic region containing HBEGF.** For each SNP, the P-value (log10 scale) of the association with ACD is represented (y-axis, left). The recombination rates (y-axis right), which reflect the local linkage disequilibrium structure, are also plotted.


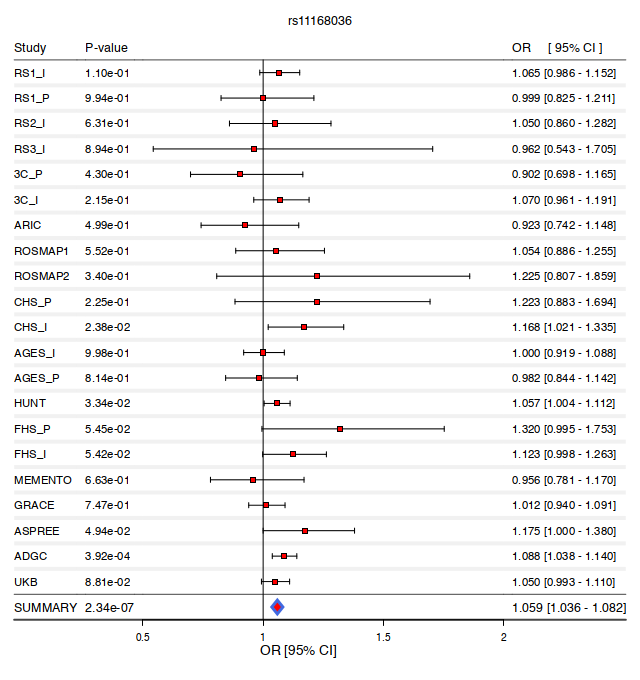


**Figure S31: Forest plot of the locus near HBEGF in ACD GWAS of European ancestry.**

### 2 – 2 – 5 ZNF652 locus

**Figure S32: Regional association plot showing the genomic region containing ZNF652.** For each SNP, the P-value (log10 scale) of the association with ACD is represented (y-axis, left). The recombination rates (y-axis right), which reflect the local linkage disequilibrium structure, are also plotted.


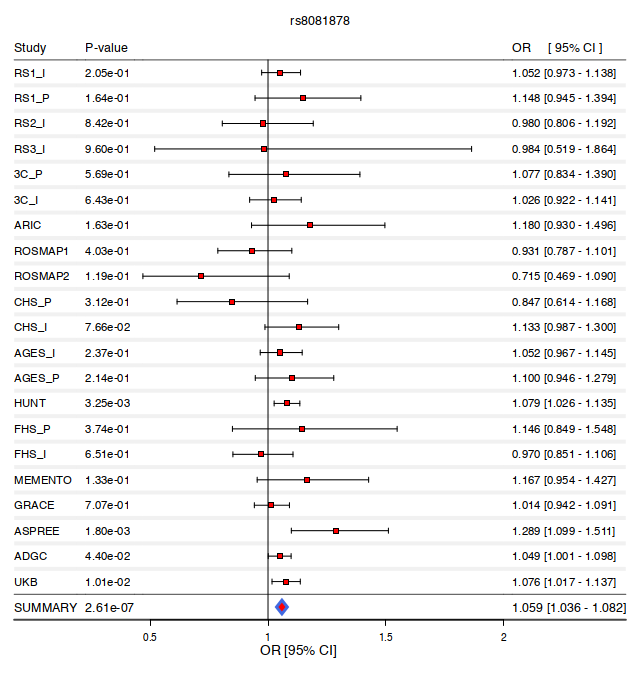


**Figure S33: Forest plot of the locus near ZNF652 in ACD GWAS of European ancestry.**

### 2 – 2 – 6 SREBF1 locus

**Figure S34: Regional association plot showing the genomic region containing SREBF1.** For each SNP, the P-value (log10 scale) of the association with ACD is represented (y-axis, left). The recombination rates (y-axis right), which reflect the local linkage disequilibrium structure, are also plotted.


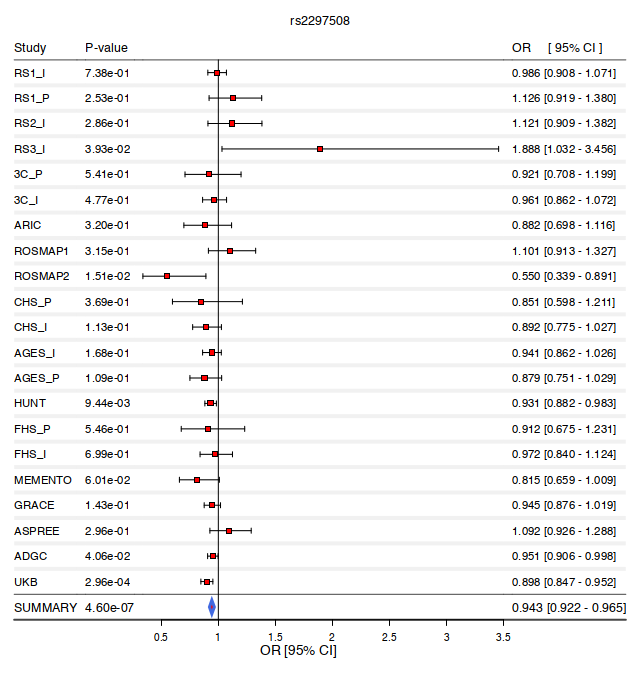


**Figure S35: Forest plot of the locus near SREBF1 in ACD GWAS of European ancestry.**

### 2 – 2 – 7 INPP5D locus

**Figure S36: Regional association plot showing the genomic region containing INPP5D.** For each SNP, the P-value (log10 scale) of the association with ACD is represented (y-axis, left). The recombination rates (y-axis right), which reflect the local linkage disequilibrium structure, are also plotted.


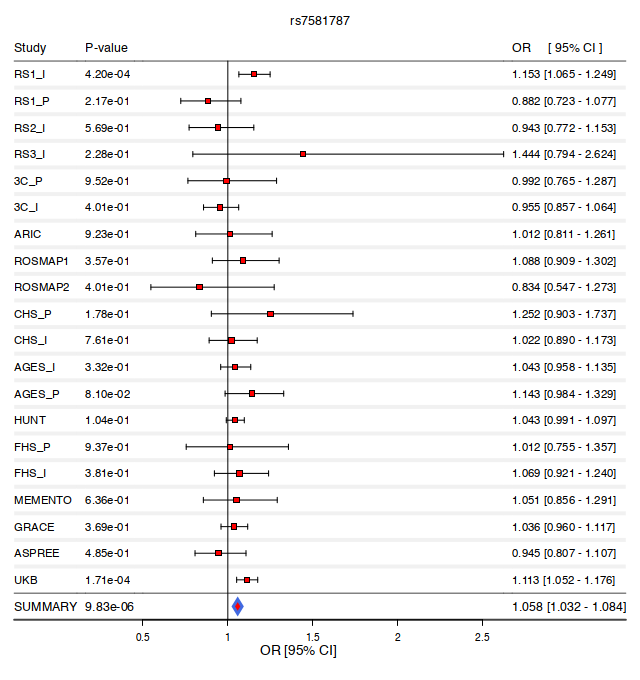


**Figure S37: Forest plot of the locus near INPP5D in ACD GWAS of European ancestry.**

## 2 – 3 Vascular dementia genome-wide significant variants

### 2 – 3 – 1 APOE locus

**Figure S38: Regional association plot showing the genomic region containing INPP5D.** For each SNP, the P-value (log10 scale) of the association with ACD is represented (y-axis, left). The recombination rates (y-axis right), which reflect the local linkage disequilibrium structure, are also plotted.


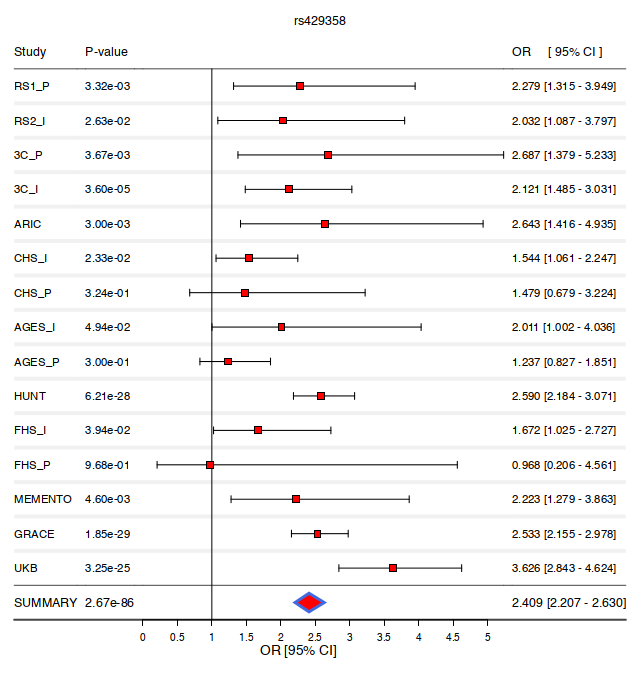


**Figure S39: Forest plot of the locus near INPP5D in ACD GWAS of European ancestry.**

## 2 – 4 Vascular dementia suggestive variants

### **2 – 4 – 1 SPRY2 locus**

**Figure S40: Regional association plot showing the genomic region containing SPRY2.** For each SNP, the P-value (log10 scale) of the association with VaD is represented (y-axis, left). The recombination rates (y-axis right), which reflect the local linkage disequilibrium structure, are also plotted.


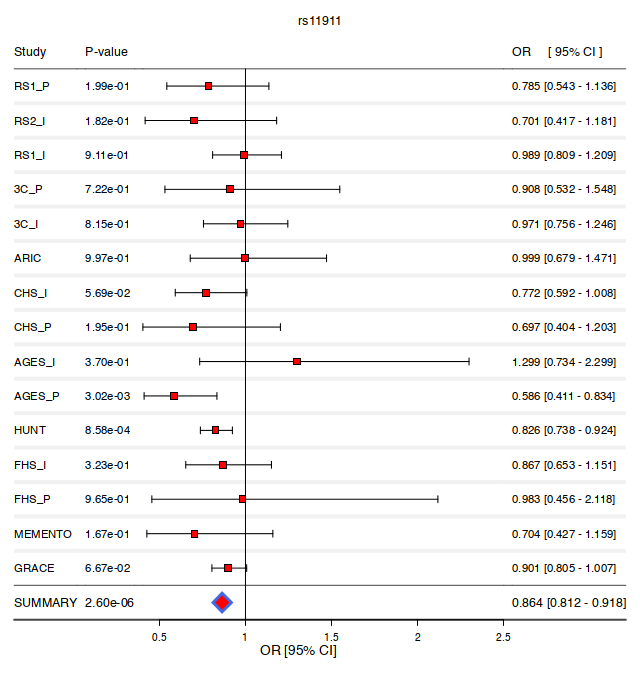


**Figure S41: Forest plot of the locus near SPRY2 in VaD GWAS of European ancestry.**

### 2 – 4 – 2 SEMA6D locus

**Figure S42: Regional association plot showing the genomic region containing SEMA6D.** For each SNP, the P-value (log10 scale) of the association with VaD is represented (y-axis, left). The recombination rates (y-axis right), which reflect the local linkage disequilibrium structure, are also plotted.


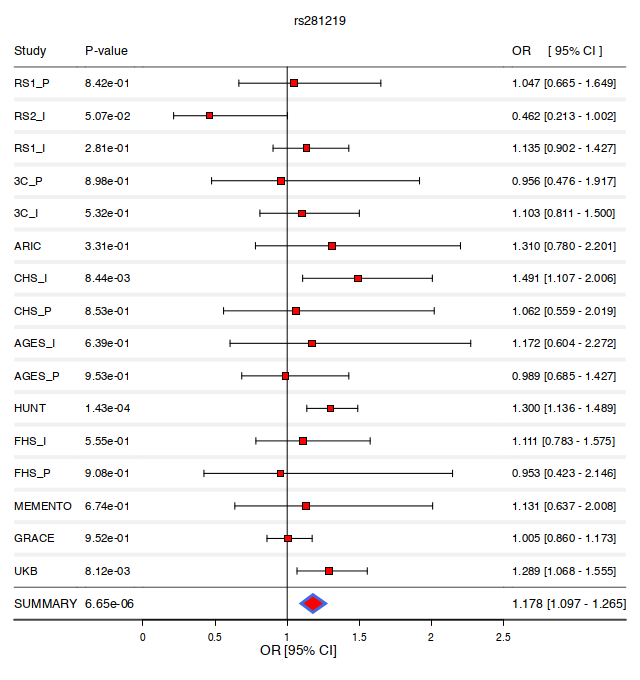


**Figure S43: Forest plot of the locus near SEMA6D in VaD GWAS of European ancestry.**

### 2 – 4 – 3 SCARB1 locus

**Figure S44: Regional association plot showing the genomic region containing SCARB1.** For each SNP, the P-value (log10 scale) of the association with VaD is represented (y-axis, left). The recombination rates (y-axis right), which reflect the local linkage disequilibrium structure, are also plotted.


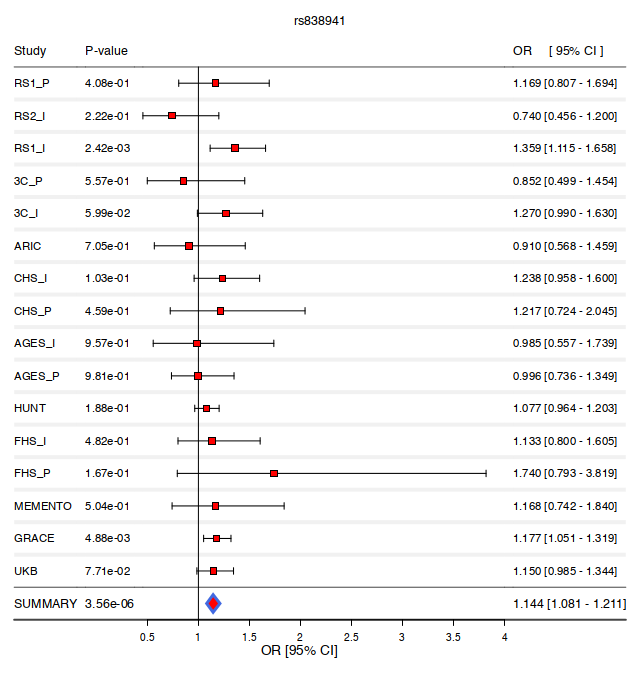


**Figure S45: Forest plot of the locus near SCARB1 in VaD GWAS of European ancestry.**

### **2 – 4 – 4 PSMA3 locus**

**Figure S46: Regional association plot showing the genomic region containing PSMA3.** For each SNP, the P-value (log10 scale) of the association with VaD is represented (y-axis, left). The recombination rates (y-axis right), which reflect the local linkage disequilibrium structure, are also plotted.


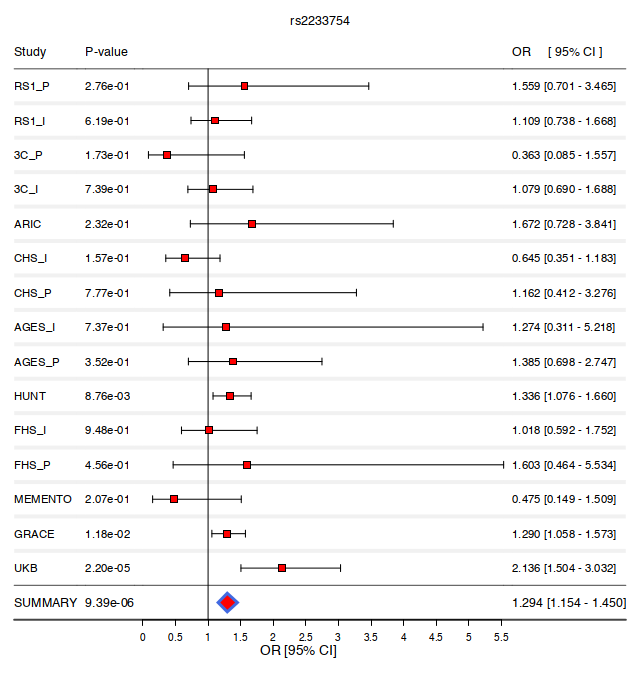


**Figure S47: Forest plot of the locus near PSMA3 in VaD GWAS of European ancestry.**

### **2 – 4 – 5 LINC02113 locus**

**Figure S48: Regional association plot showing the genomic region containing LINC02113.** For each SNP, the P-value (log10 scale) of the association with VaD is represented (y-axis, left). The recombination rates (y-axis right), which reflect the local linkage disequilibrium structure, are also plotted.


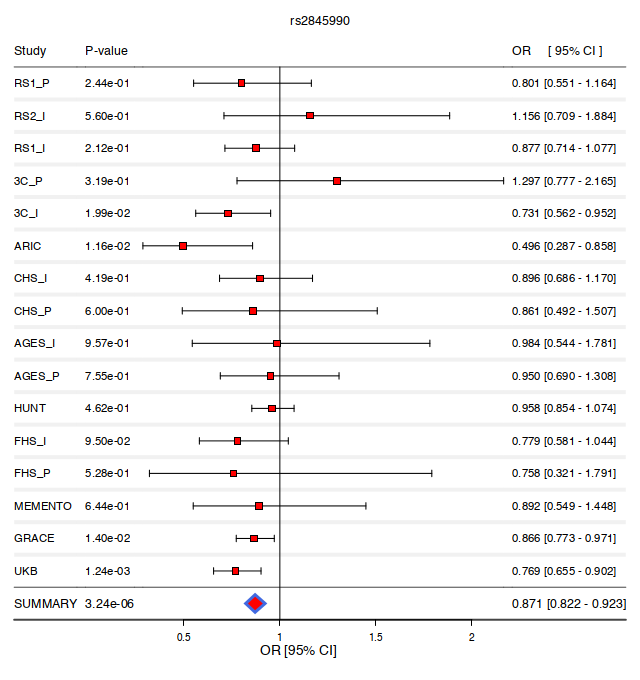


**Figure S49: Forest plot of the locus near LINC02113 in VaD GWAS of European ancestry.**

### **2 – 4 – 6 GIP locus**

**Figure S50: Regional association plot showing the genomic region containing GIP.** For each SNP, the P-value (log10 scale) of the association with VaD is represented (y-axis, left). The recombination rates (y-axis right), which reflect the local linkage disequilibrium structure, are also plotted.


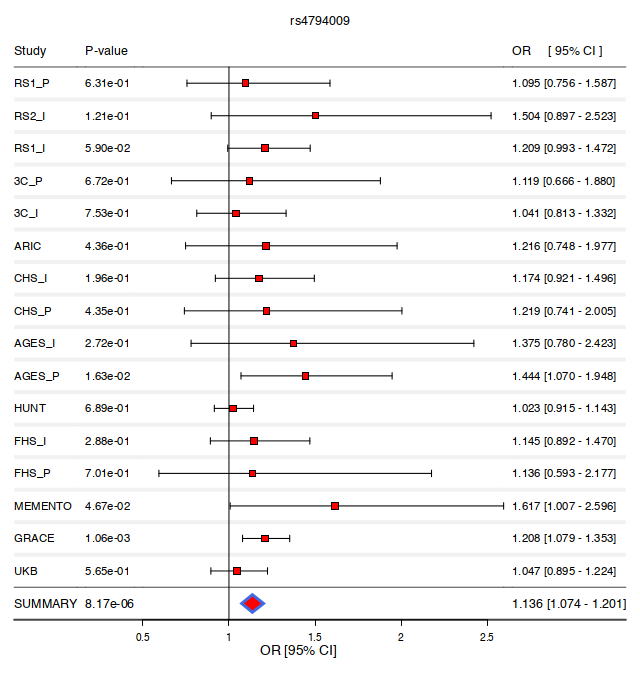


**Figure S51: Forest plot of the locus near GIP in VaD GWAS of European ancestry.**

### **2 – 4 – 7 DOK5 locus**

**Figure S52: Regional association plot showing the genomic region containing DOK5.** For each SNP, the P-value (log10 scale) of the association with VaD is represented (y-axis, left). The recombination rates (y-axis right), which reflect the local linkage disequilibrium structure, are also plotted.


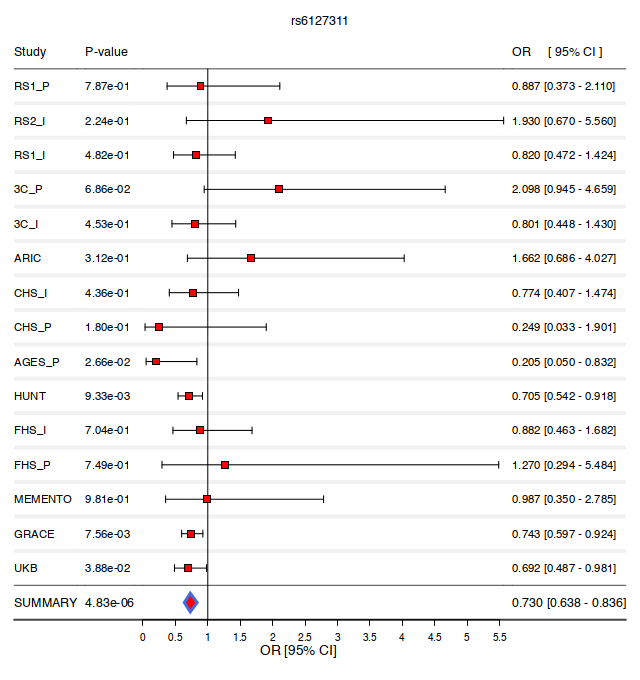


**Figure S53: Forest plot of the locus near DOK5 in VaD GWAS of European ancestry.**

### **2 – 4 – 8 GALNT18 locus**

**Figure S54: Regional association plot showing the genomic region containing GALNT18.** For each SNP, the P-value (log10 scale) of the association with VaD is represented (y-axis, left). The recombination rates (y-axis right), which reflect the local linkage disequilibrium structure, are also plotted.


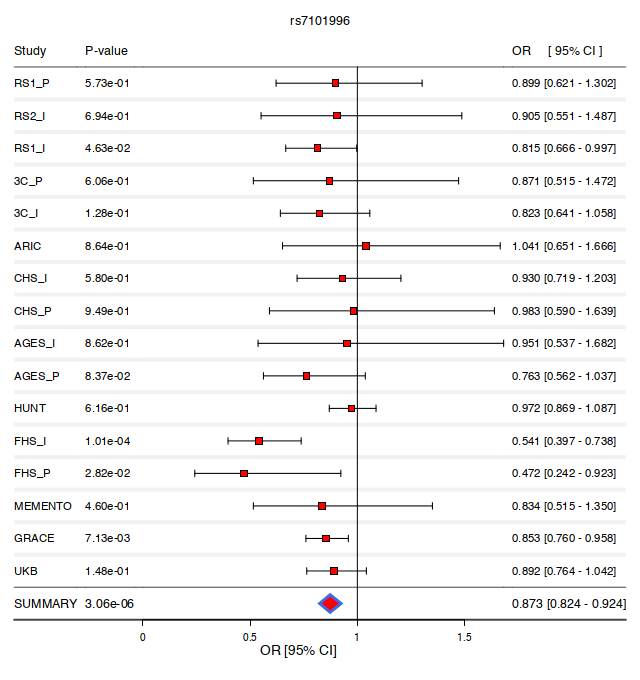


**Figure S55: Forest plot of the locus near GALNT18 in VaD GWAS of European ancestry.**

### **2 – 4 – 9 WAC locus**

**Figure S56: Regional association plot showing the genomic region containing WAC.** For each SNP, the P-value (log10 scale) of the association with VaD is represented (y-axis, left). The recombination rates (y-axis right), which reflect the local linkage disequilibrium structure, are also plotted.


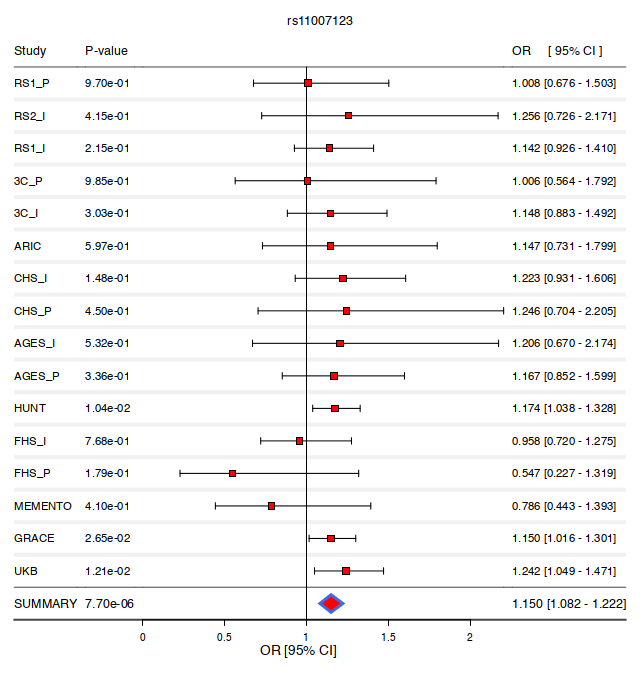


**Figure S57: Forest plot of the locus near WAC in VaD GWAS of European ancestry.**

### **2 – 4 – 10 ERBB4 locus**

**Figure S58: Regional association plot showing the genomic region containing ERBB4.** For each SNP, the P-value (log10 scale) of the association with VaD is represented (y-axis, left). The recombination rates (y-axis right), which reflect the local linkage disequilibrium structure, are also plotted.


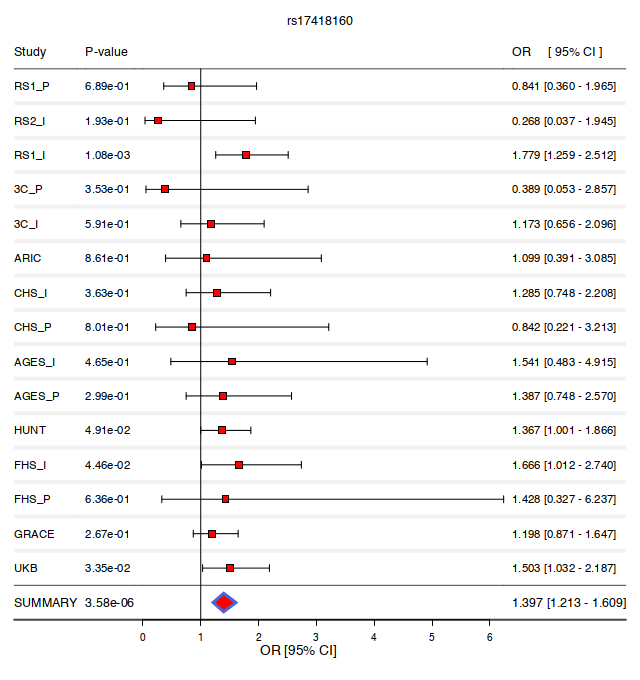


**Figure S59: Forest plot of the locus near ERBB4 in VaD GWAS of European ancestry.**

### **2 – 4 – 11 PRKCE locus**

**Figure S60: Regional association plot showing the genomic region containing PRKCE.** For each SNP, the P-value (log10 scale) of the association with VaD is represented (y-axis, left). The recombination rates (y-axis right), which reflect the local linkage disequilibrium structure, are also plotted.


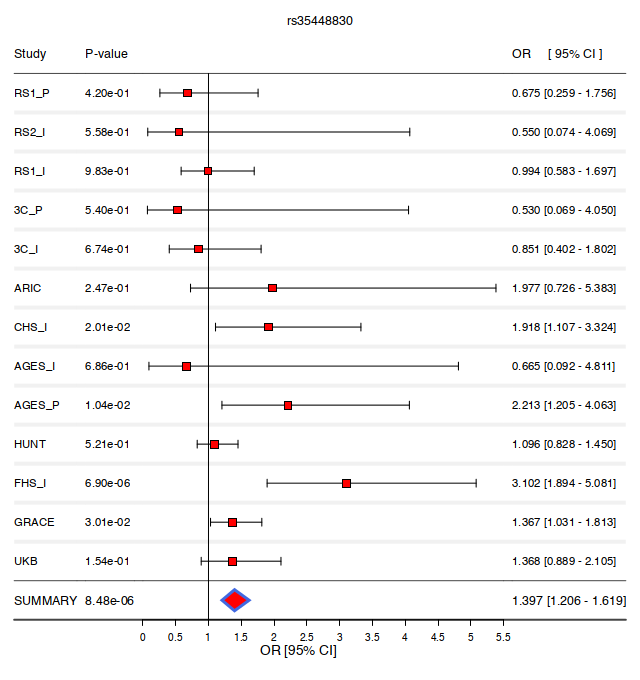


**Figure S61: Forest plot of the locus near PRKCE in VaD GWAS of European ancestry.**

### **2 – 4 – 12 PHACTR3 locus**

**Figure S62: Regional association plot showing the genomic region containing PHACTR3.** For each SNP, the P-value (log10 scale) of the association with VaD is represented (y-axis, left). The recombination rates (y-axis right), which reflect the local linkage disequilibrium structure, are also plotted


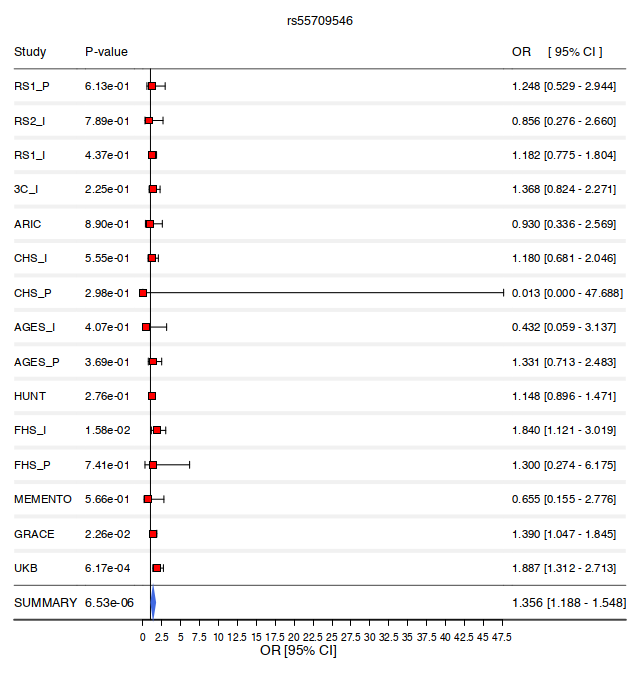


**Figure S63: Forest plot of the locus near PHACTR3 in VaD GWAS of European ancestry.**

### **2 – 4 – 13 AJAP1 locus**

**Figure S64: Regional association plot showing the genomic region containing AJAP1.** For each SNP, the P-value (log10 scale) of the association with VaD is represented (y-axis, left). The recombination rates (y-axis right), which reflect the local linkage disequilibrium structure, are also plotted.


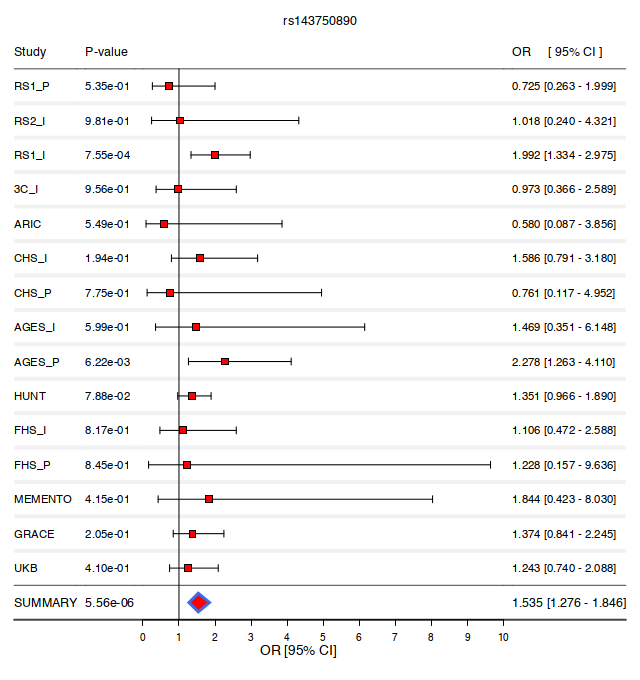


**Figure S65: Forest plot of the locus near AJAP1 in VaD GWAS of European ancestry.**

## 2 – 5 Cross-Ancestry meta-analysis plots


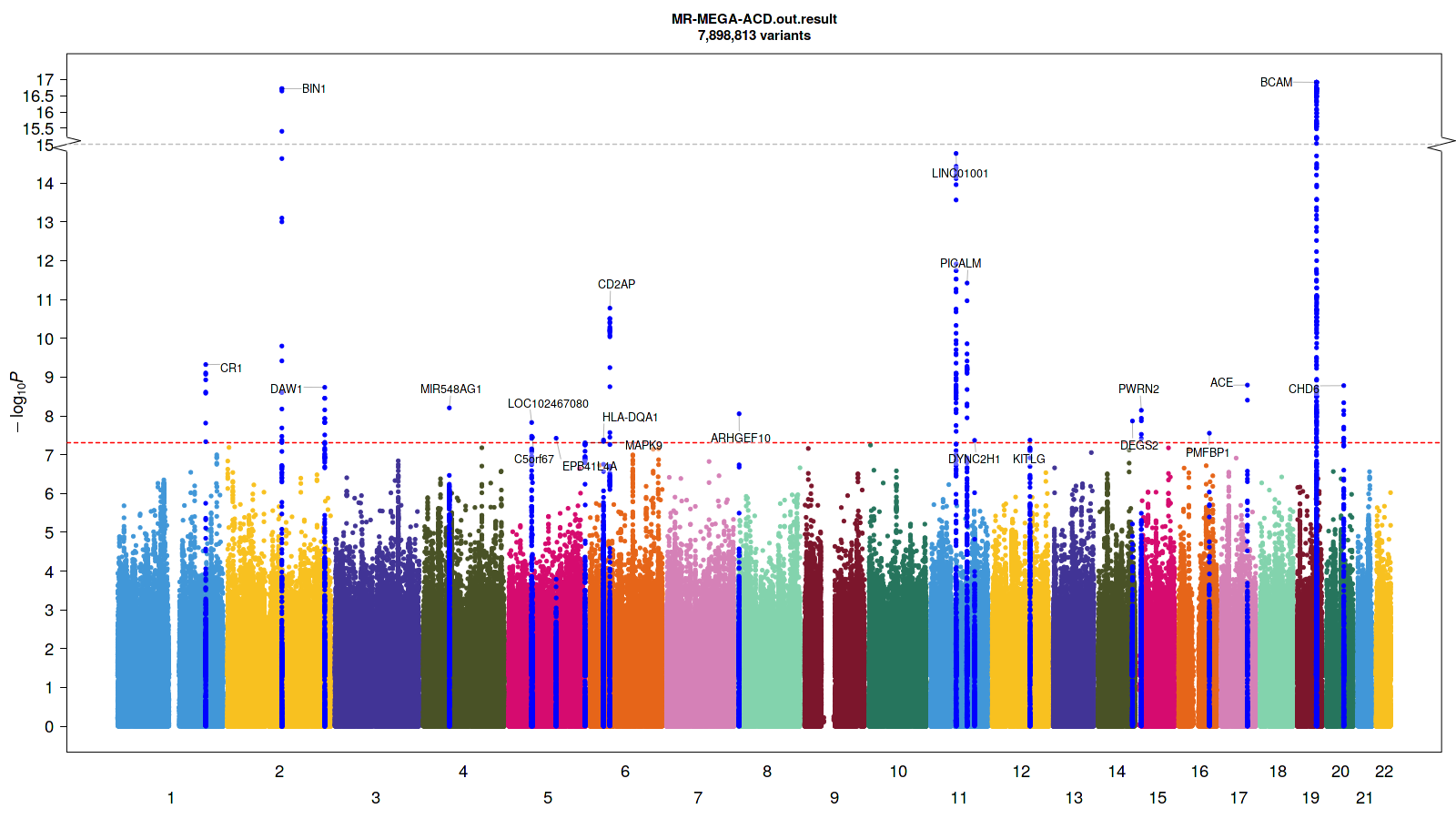


**Figure S66:** **Manhattan plot of the trans-ancestral meta-analysis of ACD GWASs.** In addition to variants in the APOE region, we identified five new genetic loci associated with VaD. Blue and red lines correspond to the p-value of 5e^-7^ and 5e^-8^ for genome-wide suggestive and significant SNPs, respectively. Manhattan plots for the cross-ancestry meta-analysis. Each dot represents a SNP, the X-axis shows the chromosomes where each SNP is located, and the Y-axis shows -log10 P-value of the association of each SNP with POAG in the cross-ancestry meta-analysis. The red horizontal line shows the genome-wide significant threshold (P-value=5e-8; -log10 P-value=7.30). The nearest gene to the most significant SNP in each locus has been labeled.


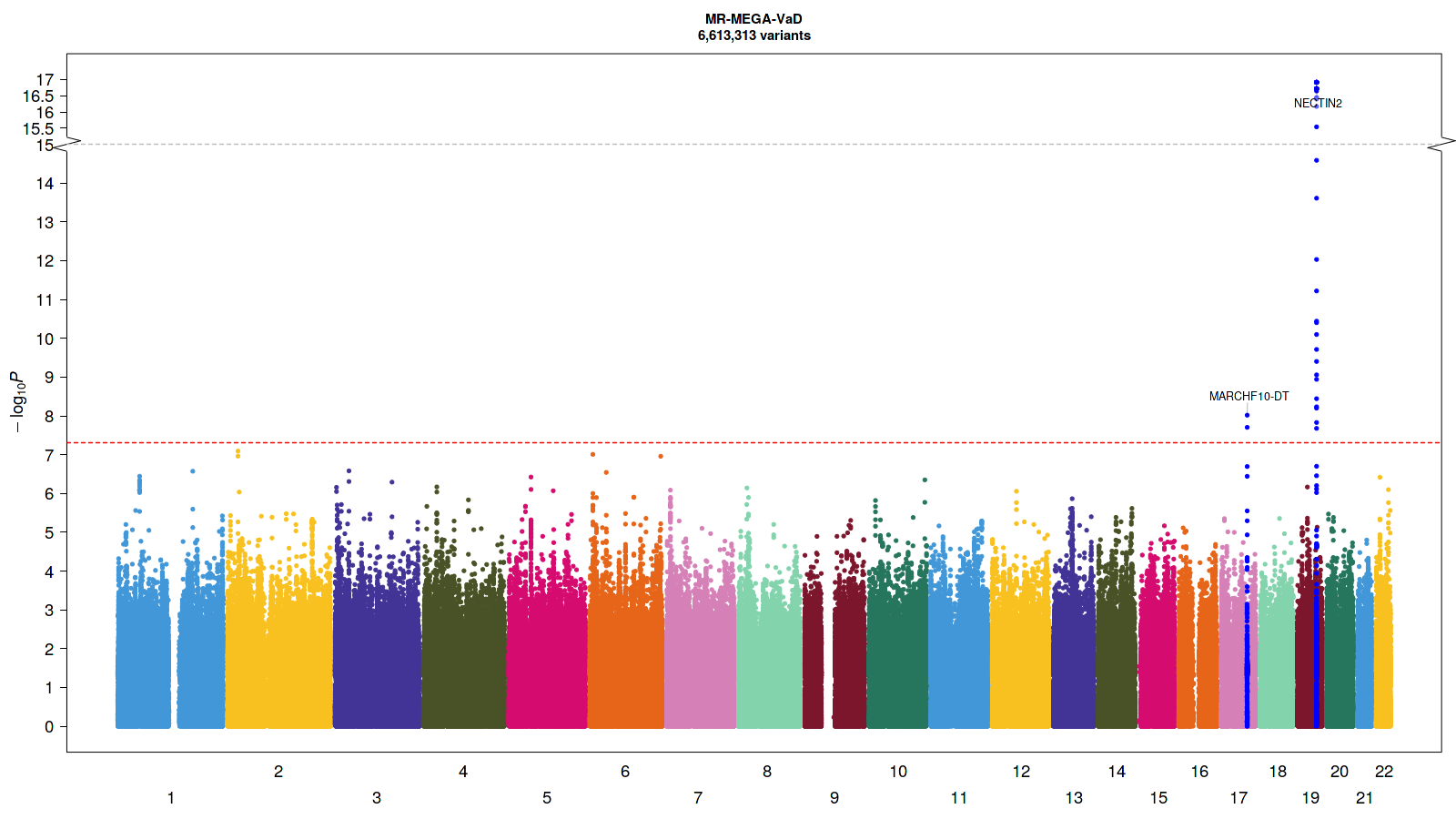


**Figure S67:** **Manhattan plot of the trans-ancestral meta-analysis of VaD GWASs.** In addition to variants in the APOE region, we identified five new genetic loci associated with VaD. Blue and red lines correspond to the p-value of 5e^-7^ and 5e^-8^ for genome-wide suggestive and significant SNPs, respectively. Manhattan plots for the cross-ancestry meta-analysis. Each dot represents a SNP, the X-axis shows the chromosomes where each SNP is located, and the Y-axis shows -log10 P-value of the association of each SNP with POAG in the cross-ancestry meta-analysis. The red horizontal line shows the genome-wide significant threshold (P-value=5e-8; -log10 P-value=7.30). The nearest gene to the most significant SNP in each locus has been labeled.

## **3 – Other Figures**

### **3 – 1 Variant Overlap with other complex traits**


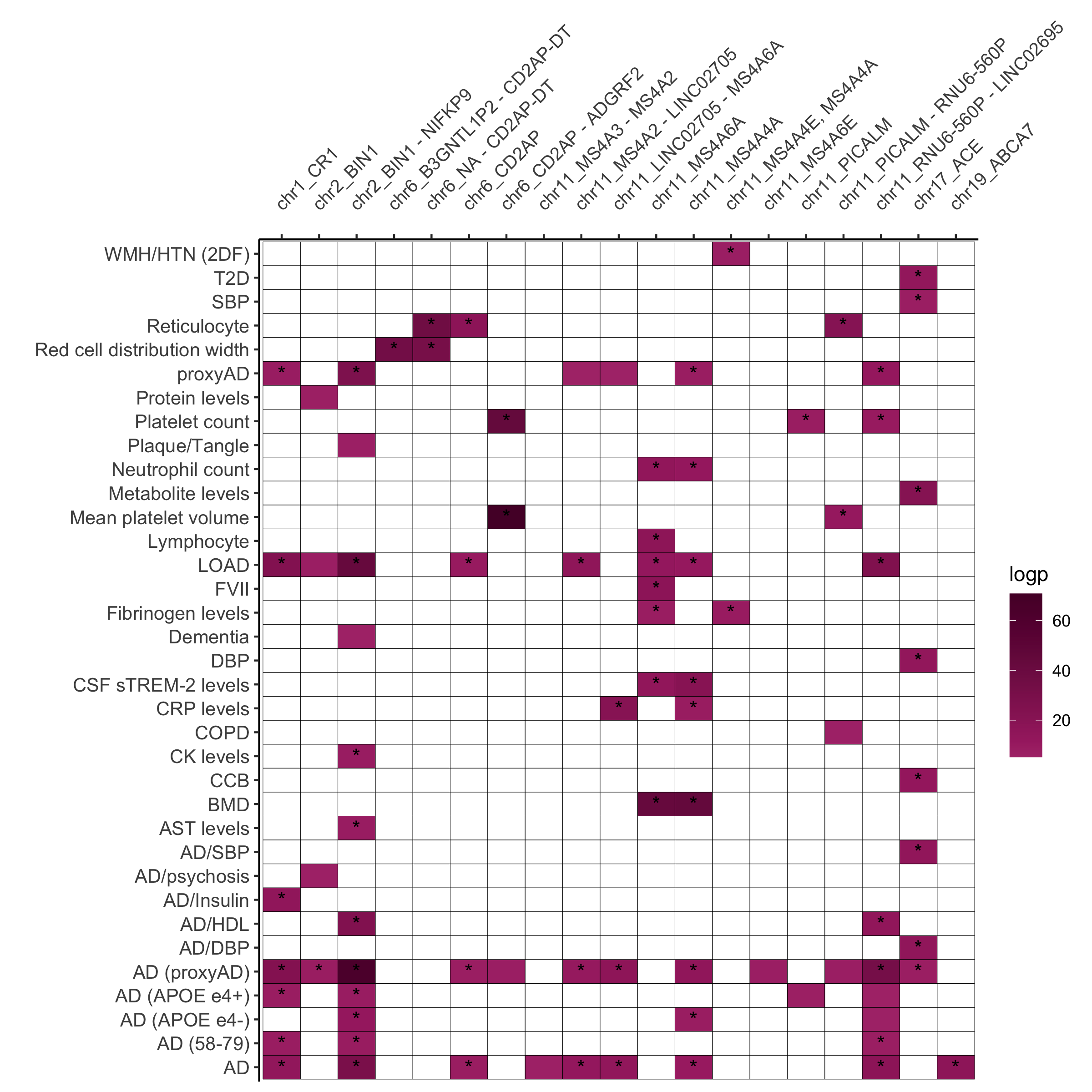


**Figure S68: Variant level overlap with complex disease traits**


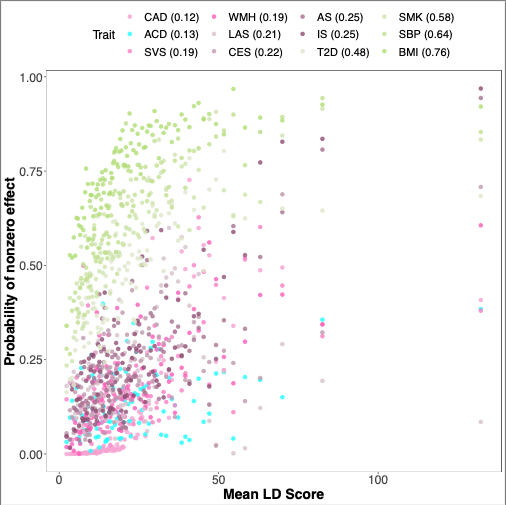


**Figure S69: Non-zero effect distribution of ACD and other complex disease traits**

### **3 – 2 Functional analysis Figures**

| 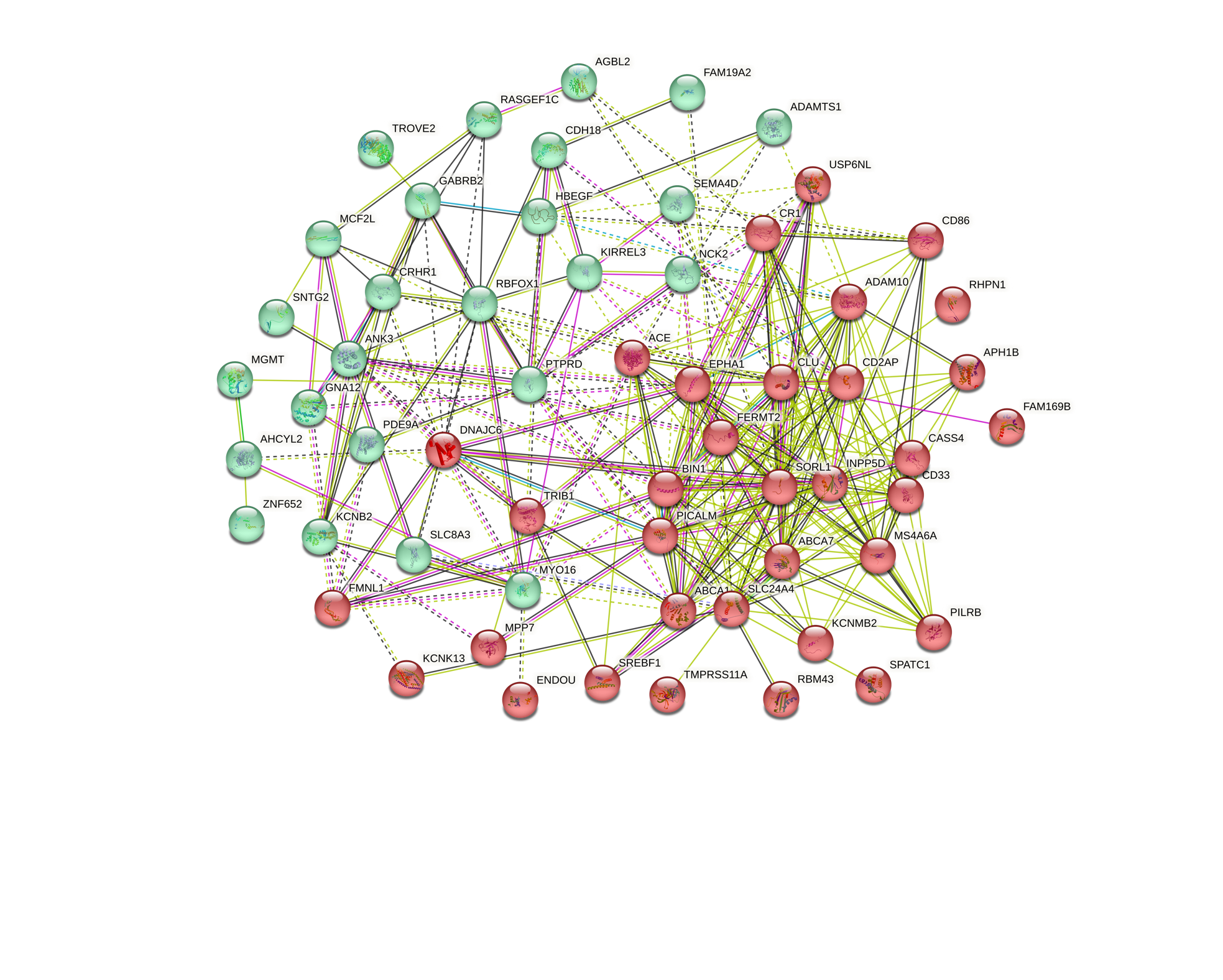 |
| --- |

**Figure S70**: **STRING clustering of ACD genes. The hierarchical** clustering of ACD in two groups provides insights into the potential interactions between prominent genes. The red cluster is highly enriched in known AD-related genes, while the green cluster mostly included our ACD-suggestive genes.

| 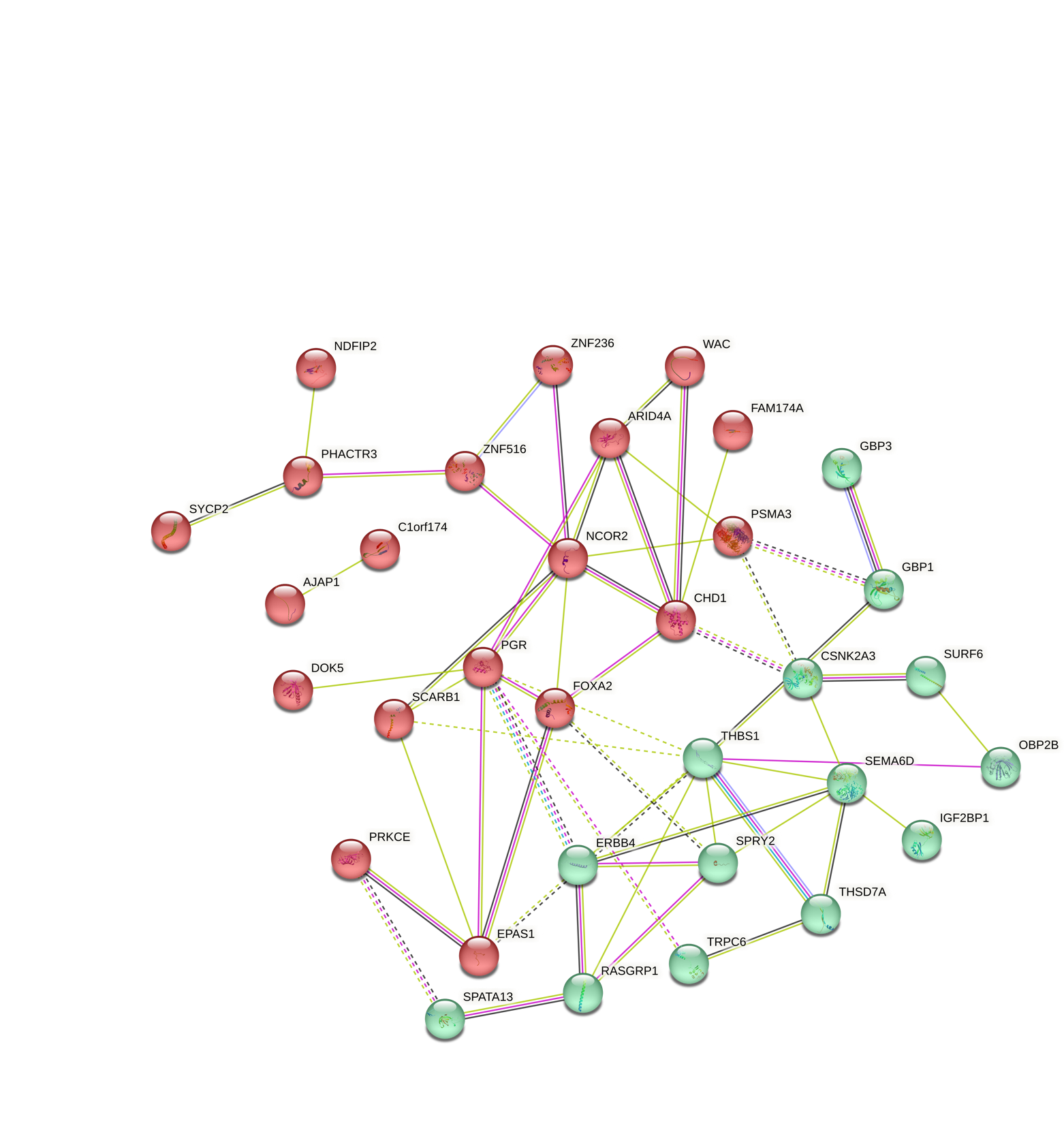 |
| --- |

**Figure S71**: **STRING clustering of VaD genes. The hierarchical** clustering of VaD in two groups provides insights into the potential interactions between prominent genes.

| 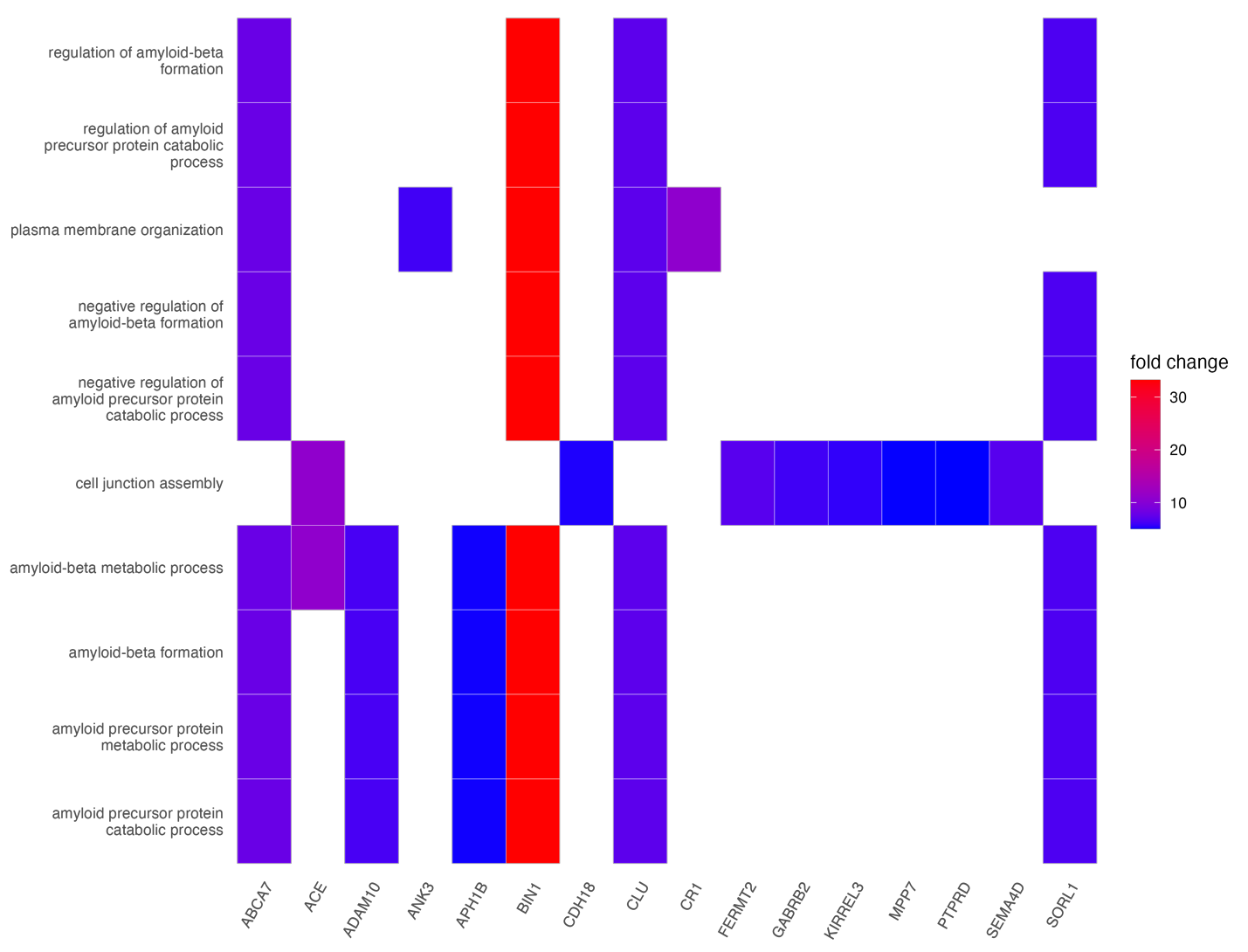 |
| --- |

**Figure S72**: **ACD Gene Ontology (GO) analysis, Biological Processes.**

| 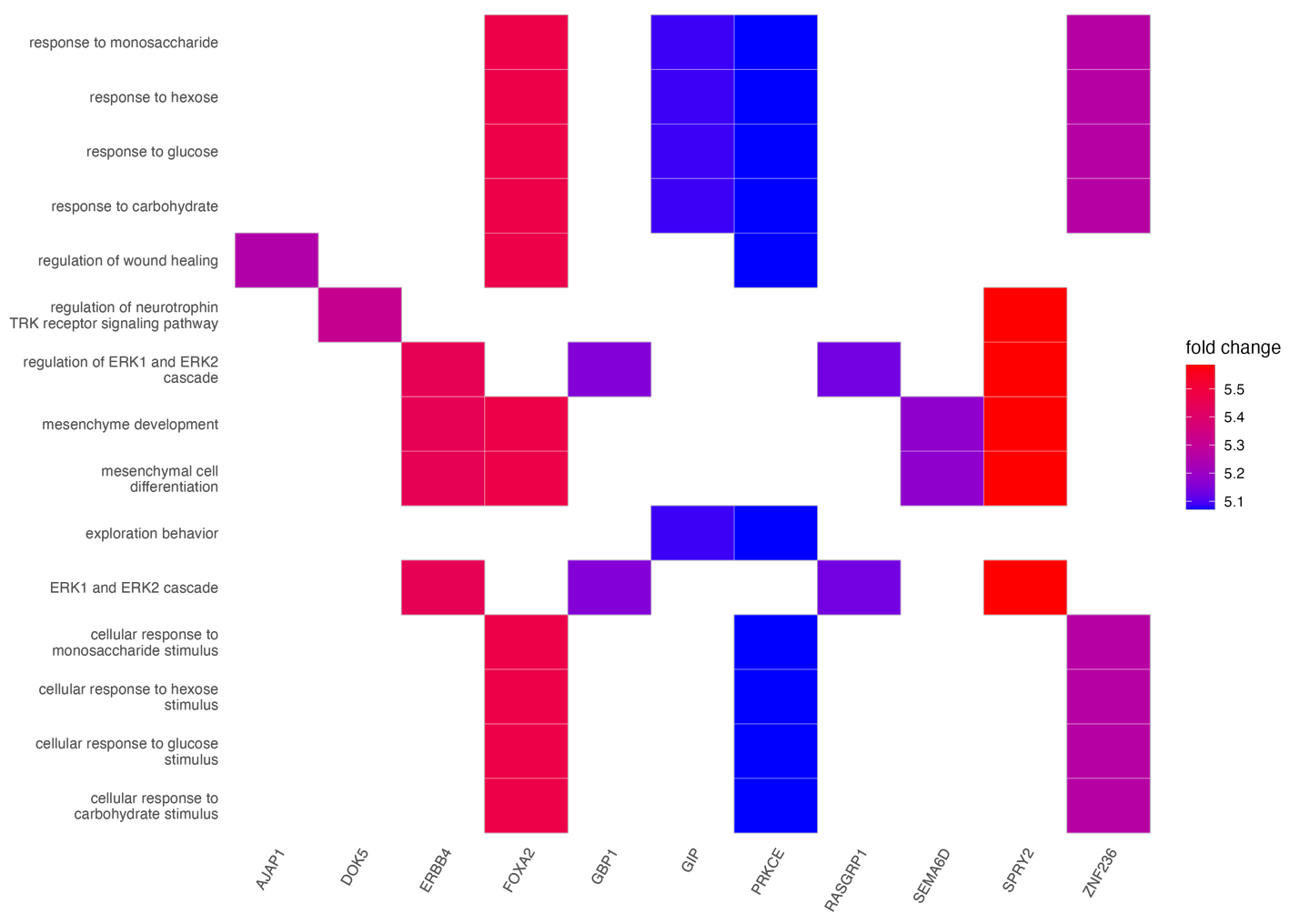 |
| --- |

**Figure S73**: **VaD Gene Ontology (GO) analysis, Biological Processes.**

| 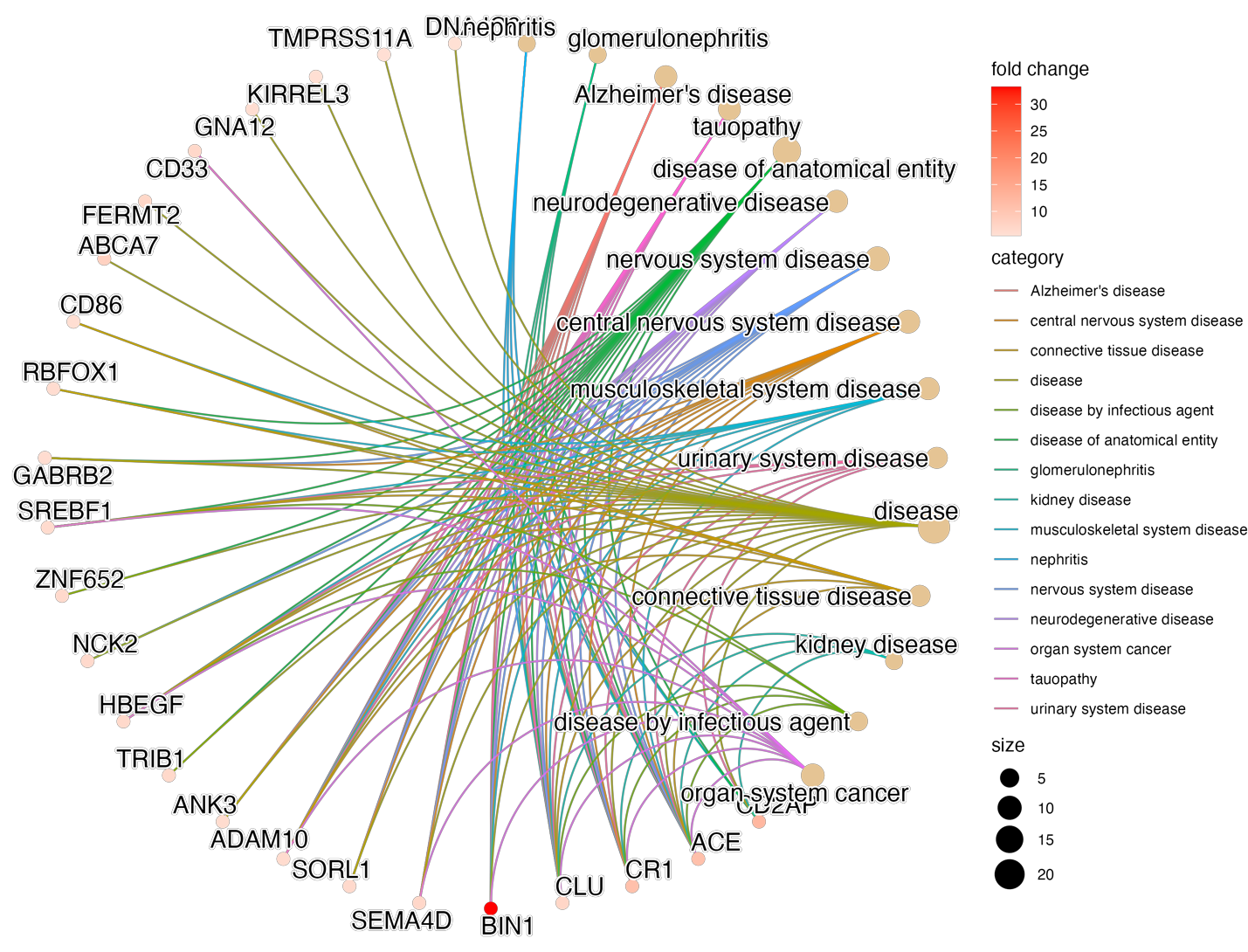 |
| --- |

**Figure S74**: **ACD disease association analysis**

| 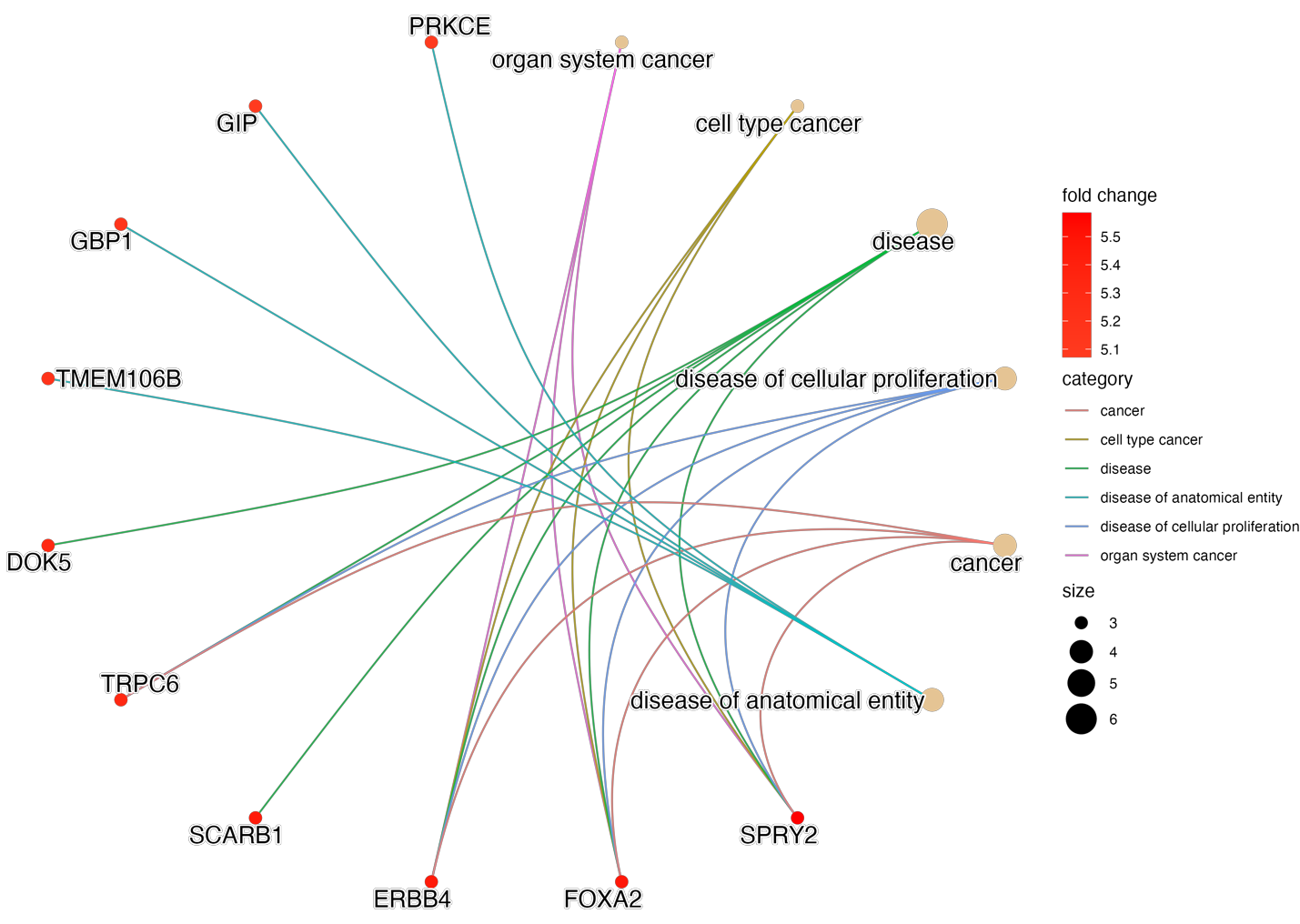 |
| --- |

**Figure S75**: **VaD disease association analysis.**
