# Supplementary material for: A genome‐wide association meta‐analysis of all‐cause and vascular dementia
Source: Alzheimers Dement. 2024 Jul 24;20(9):5973–95. doi: 10.1002/alz.14115 (PMC11497727; doi:10.1002/alz.14115)
Supplement: Supplementary file 3 — Supporting Information [file ALZ-20-5973-s003.docx]

**A genome-wide association meta-analysis of all-cause and vascular dementia**

Bernard Fongang^1,2,3,*,#^, Muralidharan Sargurupremraj^1,3,*^, Xueqiu Jian^1,3^, Aniket Mishra^4^, Olivia Skrobot^5^, Itziar de Rojas^6,7^, Vincent Damotte^8^, Joshua C Bis^9^, Kang-Hsien Fan^10^, Erin Jacobsen^11^, Gloria Hoi-Yee Li^12^, Jingyun Yang^13^, Bizzarro Alessandra^14^, Lauria Alessandra^14^, Saima Hilal^15,16^, Joyce Chong^15^, Yuek Ling Chai^15^, M.J. Knol^17^, Maria Pina Concas^18^, Girotto Giorgia^18,19^, Moeen Riaz^20^, Chenglong Yu^20^, Alexander Guðjónsson^21^, Paul Lacaze^20^, Adam C Naj^22^, Monica Goss^1^, Yannick W. Ngouongo^1^, Aicha Soumare^4^, Vincent Bouteloup^4,23^, Vilmundur Guðnason^21^, Petronilla Battista^24^, Aurora Santin^19^, Beatrice Spedicati^19^, Rodolfo Sardone^25,26^, Lenore Launer^27^, Jan Bressler^28^, Rebecca F Gottesman^29^, Quentin Le Grand^30^, Ilana Caro^30^, Gennady V. Roshchupkin^17,31^, Hampton L. Leonard^32,33,34^, Chaojie Yang^35,36^, Traci M. Bartz^37,38^, Constance Bordes^30^, Paul M. Ridker^39,40^, Mirjam I. Geerlings^41^, Natalie C. Gasca^38^, Ani Manichaikul^35^, Mike A. Nalls^32,33,34^, Stephen S. Rich^35^, Carsten O. Schmidt^42^, Stella Trompet^43,44^, Marion van Vugt^45^, Hans J. Grabe^46,47^, J Wouter Jukema^44,48,49^, Ina L. Rissanen^41^, Sylvia Wassertheil-Smoller^50^, M. Arfan Ikram^17^, Eleanor M. Simonsick^51^, W T. Longstreth^52,53^, Daniel I. Chasman^39,40^, Jerome I. Rotter^54^, Naveed Sattar^55^, David J Stott^56^, Eric J Shiroma^57^, Sigurdur Sigurdsson^58^, Mohsen Ghanbari^17^, Ulf Schminke^59^, Eric Boerwinkle^60,61^, Hugo J Aparicio^62,63^, Alexa S Beiser^62,64^, Jose R Romero^62,63^, Vasileios Lioutas^62,65^, Ruiqi Wang^62,64^, Chloe Sarnowski^66^, Alexander Teumer^42,67^, Uwe Völker^67,68^, Thomas H. Mosley^69^, Marta Marquié^6,7^, Pablo García-González^6,7^, Clàudia Olivé^6^, Raquel Puerta^6^, Amanda Cano^6,7^, Oscar Sotolongo-Grau^6,7^, Sergi Valero^6,7^, Vanesa Veronica Pytel^6^, Maitée Rosende-Roca^6,7^, Montserrat Alegret^6,7^, Lluís Tàrraga^6,7^, Mercè Boada^6,7^, Ángel Carracedo^70,71^, Emilio Franco-Macías^7,72^, Gerard Piñol-Ripoll^73,74^, Guillermo Garcia-Ribas^75,76,77,78^, Jordi Pérez-Tur^7,78,78^, Jose Luís Royo^79^, Jose María García-Alberca^80^, Luis Miguel Real^81,82^, María Eugenia Sáez^83^, María J. Bullido^7,84,85,86^, Miguel Calero^7,87,88^, Miguel Medina^7,89^, Pablo Mir^7,90,91^, Pascual Sánchez-Juan^7,92^, Pau Pastor^93,94^, Victoria Álvarez^95,96^, Benjamin Grenier-Boley^8^, Fahri Küçükali^97,98,99^, Sven Van der Lee^100,101,102^, Oliver Peters^103,104^, Anja Schneider^105,106^, Martin Dichgans^107,108,109^, Dan Rujescu^110^, Deckert Jürgen^111^, Emrah Düzel^112,113^, Jens Wiltfang^114,115,116^, Susanne Moebus^117^, Michael Wagner^118,119^, Timo Grimmer^120^, Nikolaos Scarmeas^121,122^, Jordi Clarimon^7,123^, Fermin Moreno^7,124,125^, Raquel Sánchez-Valle^126^, Luis M Real^81,127^, Eloy Rodriguez-Rodriguez^7,128^, Adolfo Lopez de Munain^77,124,129^, Alexandre de Mendonça^130^, Jakub Hort^131,132^, Caroline Graff^133^, Goran Papenberg^134^, Vilmantas Giedraitis^135^, Børge G. Nordestgaard^136,137^, Hilkka Soininen^138^, Miia Kivipelto^139,140,141,142,143^, Annakaisa Haapasalo^144^, Gael Nicolas^145^, Florence Pasquier^146^, Olivier Hanon^147^, Edna Grünblatt^148,149,150^, Julius Popp^151,152,153^, Luisa Benussi^154^, Daniela Galimberti^155,156^, Beatrice Arosio^157,158^, Patrizia Mecocci^159^, Alessio Squassina^160^, Lucio Tremolizzo^161^, Innocenzo Rainero^162^, Gianfranco Spalletta^163^, Davide Seripa^164^, Julie Williams^165^, Philippe Amouyel^8^, Frank Jessen^105,166,167^, Tsolaki Magda^168^, Ruth Frikke-Schmidt^169,170^, Kristel Sleegers^97,98,99^, Sebastiaan Engelborghs^171,172^, Rik Vandenberghe^173,174^, Martin Ingelsson^135,175,176^, Giacomina Rossi^177^, Mikko Hiltunen^178^, Rebecca Sims^165^, Alla Graban^179^, Anna Bochynska^179^, Magdalena Gugała-Iwaniuk^179^, Danuta Ryglewicz^179^, Hanna Wehr^179^, Joyce Ruifen Chong^15^, Mitchell KP Lai^15^, Venketasubramanian N^180^, Boon-Yeow Tan^181^, Angelo Baldassare Cefalù^182^, Rossella Spina^182^, Robin Guariglia^183^, Patrizia Bastiani^184^, Nicola J Armstrong^185^, Roberta Baschi^186,187^, Malgorzata Bednarska-Makaruk^188^, Regis bordet ^189,190^, Anne-Marie Bordet^189,190^, Henry Brodaty^191^, Roberta Cecchetti^192^, Srdjan Djurovic^193,194^, Grazia D’Onofrio^195^, Timo Erkinjuntti^196^, Margaret Esiri^197^, Patrick Gelé^189,190^, Catharine Joachim^198^, Teresa Juarez-Cedillo^199^, Raj Kalaria^200,201^, Pekka Karhunen^202^, Jan LACZO^131,132^, Ondrej LERCH^131,132^, Carlo Masullo^203^, Karen A Mather^191,204^, Vaclav MATOSKA^205^, Susanna Melkas^206,207^, Roberto Monastero^186,187^, Katya Numbers^191^, Francesco Panza^208,209,210^, Tuomo M Polvikoski^200,201^, Joe Quinn^183^, Arvid Rongve^211,212^, Perminder S Sachdev^191,213^, Michela Scamosci^192^, Anbupalam Thalamuthu^191^, Anne Tybjærg-Hansen^214^, Martin VYHNALEK^131,132^, Shawn K. Westaway^215^, Amy E Martinsen^216,217,218^, Anne Heidi Skogholt^218^, Cristen J Willer^219^, Eystein Stordal^220,221^, Geir Bråthen^222^, Jonas Bille Nielsen^218,219^, Lars G Fritsche^223^, Laurent F Thomas^218,224,225,226^, Linda M Pedersen^216^, Maiken E Gabrielsen^218^, Ole Kristian Drange^220,227^, Sigrid Botne Sando^218,222,228,229^, Tore Wergeland Meisingset^222,228^, Genevieve Chene^4,23^, Wei Zhou^230,231^, Christophe Tzourio^4,232^, Adrienne Tin^69^, Oscar L Lopez^233^, Haan Mary^234^, Sigrid Børte^217,218,229^, Ingunn Bosnes^220,221^, Mary Ganguli^10,11^, Ching-Lung Cheung^12^, David A Bennett^13^, Christopher Chen^15^, M. Ilyas Kamboh^10^, Claudia Satizabal^1,3^, M. Kamran Ikram^17,235^, Hieab Adams^236,237,238^, Yang Qiong^64^, Gerard D. Schellenberg^22^, Geir Selbæk^217,228,239,240^, Kristian Hveem^218,241,242^, Ole A Andreassen^243,244^, Alfredo Ramirez^105,245,246,247^, Carole Dufouil^4,23^, Wiesje van der Flier^248^, John-Anker Zwart^216,217,218^, Stéphanie Debette^4,249^, Myriam Fornage^28,250^, Bendik Winsvold^218,251,252^, Jean-Charles Lambert^8^, Agustin Ruiz^6,7^, Patrick G. Kehoe^253^, Galit Weinstein^254,#^, and Sudha Seshadri^1,255,256,257,#^, for the Cohorts for Heart and Aging Research in Genomic Epidemiology (CHARGE)

^1^ Glenn Biggs Institute for Alzheimer’s & Neurodegenerative Diseases, University of Texas Health Science Center, San Antonio, TX, USA
^2^ Department of Biochemistry and Structural Biology, University of Texas Health Science Center, San Antonio, TX, USA
^3^ Department of Population Health Sciences, University of Texas Health Science Center, San Antonio, TX, USA
^4^ University of Bordeaux, Inserm, Bordeaux Population Health Research Center, UMR 1219, F-33000 Bordeaux, France
^5^ Population Health Sciences, Bristol Medical School, University of Bristol, Bristol, UK
^6^ Research Center and Memory Clinic, ACE Alzheimer Center Barcelona. Universitat Internacional de Catalunya, Spain
^7^ Networking Research Center on Neurodegenerative Diseases (CIBERNED), Instituto de Salud Carlos III, Madrid, Spain
^8^ Univ. Lille, Inserm, CHU Lille, Institut Pasteur de Lille, U1167-RID-AGE facteurs de risque et déterminants moléculaires des maladies liés au vieillissement, Lille, France
^9^ Cardiovascular Health Research Unit, Department of Medicine, University of Washington, Seattle, Washington
^10^ Department of Human Genetics, School of Public Health, University of Pittsburgh, Pittsburgh, PA, USA
^11^ Department of Psychiatry and Neurology, School of Medicine, University of Pittsburgh, Pittsburgh, PA, USA
^12^ Department of Pharmacology and Pharmacy, Centre for Genomic Sciences, The University of Hong Kong, Hong Kong
^13^ Rush Alzheimer’s Disease Center and Department of Neurological Sciences, Rush University Medical Center, Chicago, IL, USA
^14^ GERIATRICS UNIT, FONDAZIONE POLICLINICO UNIVERSITARIO A. GEMELLI IRCCS, LARGO A GEMELLI, 8 -00168 ROMA- ITALY
^15^ Department of Pharmacology, National University of Singapore, Singapore
^16^ Saw Swee Hock School of Public Health, National University of Singapore and National University Health System, Singapore
^17^ Department of Epidemiology, Erasmus MC, University Medical Center, Rotterdam, the Netherlands
^18^ Institute for Maternal and Child Health, IRCCS Burlo Garofolo, 34127 Trieste, Italy
^19^ Department of Medicine, Surgery and Health Sciences, University of Trieste, 34139 Trieste, Italy
^20^ Department of Epidemiology and Preventive Medicine, Monash University, Melbourne, VIC, Australia
^21^ Faculty of Medicine, University of Iceland, Reykjavik, Iceland
^22^ Department of Biostatistics and Epidemiology/Center for Clinical Epidemiology and Biostatistics, University of Pennsylvania Perelman School of Medicine, Philadelphia, PA, USA
^23^ Pôle de Santé Publique Centre Hospitalier Universitaire (CHU) de Bordeaux, Bordeaux, France
^24^ Istituti Clinici Scientifici Maugeri, Pavia. IRCCS di Bari, Bari, Italy
^25^ Department of Translational Biomedicine and Neuroscience, University of Bari “Aldo Moro”
^26^ Unit of Methodology and Data Sciences for Population Health, National Institute IRCCS Saverio de Bellis Research Hospital, Castellana Grotte, Italy
^27^ Laboratory of Epidemiology and Population Sciences, Intramural Research Program, National Institute of Aging, National Institutes of Health, Bethesda, MD, USA
^28^ Human Genetics Center, School of Public Health, The University of Texas Health Science Center at Houston, Houston, TX, USA
^29^ Stroke Branch, National Institute of Neurological Disorders and Stroke Intramural Program, National Institutes of Health, Bethesda, MD
^30^ University of Bordeaux, Inserm, Bordeaux Population Health Research Center, team ELEANOR, UMR 1219, F-33000 Bordeaux, France
^31^ Department of Radiology and Nuclear Medicine, Erasmus MC University Medical Center
^32^ Center for Alzheimer’s and Related Dementias, National Institutes of Health, Bethesda, USA
^33^ Laboratory of Neurogenetics, National Institute on Aging, National Institutes of Health, Bethesda USA
^34^ Data Tecnica International LLC, Glen Echo, USA
^35^ Center for Public Health Genomics, University of Virginia, Charlottesville, VA, USA
^36^ Department of Biochemistry and Molecular Genetics, University of Virginia, Charlottesville, VA, USADepartment of Biochemistry and Molecular Genetics, University of Virginia, Charlottesville, VA, USA
^37^ Cardiovascular Health Research Unit, Department of Medicine, University of Washington, Seattle, WA, USA
^38^ Department of Biostatistics, University of Washington, Seattle, WA, USA
^39^ Division of Preventive Medicine, Brigham and Women’s Hospital, Boston, MA 02215, USA
^40^ Harvard Medical School, Boston, MA 02115, USA
^41^ Julius Center for Health Sciences and Primary Care, University Medical Center Utrecht, Utrecht University, Utrecht, the Netherlands
^42^ University Medicine Greifswald, Institute for Community Medicine, SHIP/KEF, Germany
^43^ Department of Internal Medicine, Section of Gerontology and Geriatrics, Leiden University Medical Center, Leiden, the Netherlands
^44^ Department of Cardiology, Leiden University Medical Center, Leiden, the Netherlands
^45^ Division Heart & Lungs, Department of Cardiology, University Medical Center Utrecht, Utrecht University, Utrecht, The Netherlands
^46^ Department of Psychiatry and Psychotherapy, University Medicine Greifswald, Germany
^47^ German Center for Neurodegenerative Diseases (DZNE), Site Rostock/ Greifswald, Rostock, Germany
^48^ Netherlands Heart Institute, Utrecht, the Netherlands
^49^ Einthoven Laboratory for Experimental Vascular Medicine, LUMC, Leiden, the Netherlands
^50^ Department of Epidemiology and Population Health, Albert Einstein College of Medicine, New York, NY, USA
^51^ Longitudinal Studies Section, Translational Gerontology Branch, National Institute on Aging, Baltimore, Maryland, USA
^52^ Department of Epidemiology, University of Washington, Seattle, WA, USA
^53^ Department of Neurology, University of Washington, Seattle, Washington, USA
^54^ The Institute for Translational Genomics and Population Sciences, Department of Pediatrics, The Lundquist Institute for Biomedical Innovation at Harbor-UCLA Medical Center, CA, USA
^55^ BHF Glasgow Cardiovascular Research Centre, Faculty of Medicine, Glasgow, UK
^56^ Institute of Cardiovascular and Medical Sciences, College of Medical, Veterinary and Life Sciences, University of Glasgow, UK
^57^ Laboratory of Epidemiology and Population Sciences - National Institute of Health
^58^ Icelandic Heart Association, Kopavogur, Iceland
^59^ University Medicine Greifswald, Department of Neurology, Greifswald, Germany
^60^ Human Genetics Center, School of Public Health, University of Texas Health Science Center at Houston, Houston, TX, USA
^61^ Human Genome Sequencing Center, Baylor College of Medicine, Houston, TX, USA
^62^ Framingham Heart Study, Framingham, MA, USA
^63^ Department of Neurology, Boston University School of Medicine, Boston, MA 2115, USA
^64^ Department of Biostatistics, Boston University School of Public Health, Boston, MA, USA
^65^ Department of Neurology, Beth Israel Deaconess Medical Center, Boston, MA, USA
^66^ Department of Epidemiology, Human Genetics and Environmental Sciences, University of Texas Health Science Center at Houston, School of Public Health, Houston, TX
^67^ DZHK (German Centre for Cardiovascular Research), Partner Site Greifswald, Greifswald, Germany
^68^ Interfaculty Institute for Genetics and Functional Genomics, University Medicine Greifswald, Greifswald, Germany
^69^ Memory Impairment and Neurodegenerative Dementia (MIND) Center and Department of Medicine, University of Mississippi Medical Center, Jackson, MS
^70^ Grupo de Medicina Xenómica, CIBERER, CIMUS. Universidade de Santiago de Compostela, Santiago de Compostela, Spain
^71^ Fundación Pública Galega de Medicina Xenómica- IDIS, Santiago de Compostela, Spain
^72^ Unidad de Demencias, Servicio de Neurología y Neurofisiología. Instituto de Biomedicina de Sevilla (IBiS), Hospital Universitario Virgen del Rocío/CSIC/Universidad de Sevilla, Seville, Spain
^73^ Unitat Trastorns Cognitius, Hospital Universitari Santa Maria de Lleida, Lleida, Spain
^74^ Institut de Recerca Biomedica de Lleida (IRBLLeida), Lleida, Spain
^75^ Hospital Universitario Ramon y Cajal, IRYCIS, Madrid, Spain
^76^ Unitat de Genètica Molecular, Institut de Biomedicina de València-CSIC, Valencia, Spain
^77^ CIBERNED, Network Center for Biomedical Research in Neurodegenerative Diseases, National Institute of Health Carlos III, Madrid, Spain
^78^ Unidad Mixta de Neurologia Genètica, Instituto de Investigación Sanitaria La Fe, Valencia, Spain
^79^ Departamento de Especialidades Quirúrgicas, Bioquímica e Inmunología. School of Medicine. University of Malaga. Málaga, Spain
^80^ Alzheimer Research Center & Memory Clinic, Instituto Andaluz de Neurociencia, Málaga, Spain
^81^ Unidad Clínica de Enfermedades Infecciosas y Microbiología. Hospital Universitario de Valme, Sevilla, Spain
^82^ Departamento de Especialidades Quirúrgicas, Bioquímica e Inmunología. Facultad de Medicina. Universidad de Málaga. Málaga, Spain
^83^ CAEBI, Centro Andaluz de Estudios Bioinformáticos, Sevilla, Spain
^84^ Centro de Biología Molecular Severo Ochoa (UAM-CSIC)
^85^ Instituto de Investigacion Sanitaria ‘Hospital la Paz’ (IdIPaz), Madrid, Spain
^86^ Universidad Autónoma de Madrid
^87^ CIEN Foundation/Queen Sofia Foundation Alzheimer Center/Instituto de Salud Carlos III
^88^ UFIEC, Instituto de Salud Carlos III
^89^ CIEN Foundation/Queen Sofia Foundation Alzheimer Center
^90^ Unidad de Trastornos del Movimiento, Servicio de Neurología y Neurofisiología. Instituto de Biomedicina de Sevilla (IBiS), Hospital Universitario Virgen del Rocío/CSIC/Universidad de Sevilla, Seville, Spain
^91^ Departamento de Medicina, Facultad de Medicina, Universidad de Sevilla, Seville, Spain
^92^ Alzheimer’s Centre Reina Sofia-CIEN Foundation, Centro de Investigación Biomédica en Red sobre Enfermedades Neurodegenerativas (CIBERNED), Madrid, Spain
^93^ Unit of Neurodegenerative Diseases, Department of Neurology, Hospital Germans Trias i Pujol, Badalona, Barcelona, Spain
^94^ Neurodegenerative Diseases Research Laboratory, Germans Trias i Pujol Research Laboratory, Badalona, Barcelona, Spain
^95^ Laboratorio de Genética. Hospital Universitario Central de Asturias, Oviedo, Spain
^96^ Instituto de Investigación Sanitaria del Principado de Asturias (ISPA)
^97^ Complex Genetics of Alzheimer’s Disease Group, VIB Center for Molecular Neurology, VIB, Antwerp, Belgium
^98^ Laboratory of Neurogenetics, Institute Born - Bunge, Antwerp, Belgium
^99^ Department of Biomedical Sciences, University of Antwerp, Neurodegenerative Brain Diseases Group, Center for Molecular Neurology, VIB, Antwerp, Belgium
^100^ Alzheimer Center Amsterdam, Neurology, Vrije Universiteit Amsterdam, Amsterdam UMC Location VUmc, Amsterdam, The Netherlands.
^101^ Amsterdam Neuroscience, Neurodegeneration, Amsterdam, The Netherlands
^102^ Section Genomics of Neurodegenerative Diseases and Aging, Human Genetics, Vrije Universiteit Amsterdam, Amsterdam UMC location VUmc, Amsterdam, The Netherlands
^103^ German Center for Neurodegenerative Diseases (DZNE), Berlin, Germany
^104^ Charité – Universitätsmedizin Berlin, corporate member of Freie Universität Berlin, Humboldt-Universität zu Berlin, and Berlin Institute of Health, Institute of Psychiatry and Psychotherapy, Hindenburgdamm 30, 12203 Berlin, Germany
^105^ German Center for Neurodegenerative Diseases (DZNE), Bonn, Germany
^106^ Department for Neurodegenerative Diseases and Geriatric Psychiatry, University Hospital Bonn, Venusberg-Campus 1, 53127 Bonn, Germany
^107^ Institute for Stroke and Dementia Research (ISD), University Hospital, LMU Munich, Munich, Germany.
^108^ German Center for Neurodegenerative Diseases (DZNE), Munich, Germany
^109^ Munich Cluster for Systems Neurology (SyNergy), Munich, Germany
^110^ Martin-Luther-University Halle-Wittenberg, University Clinic and Outpatient Clinic for Psychiatry, Psychotherapy and Psychosomatics, Halle (Saale), Germany
^111^ Department of Psychiatry, Psychosomatics and Psychotherapy, Center of Mental Health, University Hospital of Würzburg, Germany
^112^ German Center for Neurodegenerative Diseases (DZNE), Magdeburg, Germany
^113^ Institute of Cognitive Neurology and Dementia Research (IKND), Otto-von-Guericke University, Magdeburg, Germany
^114^ Department of Psychiatry and Psychotherapy, University Medical Center Goettingen, Goettingen, Germany
^115^ German Center for Neurodegenerative Diseases (DZNE), Goettingen, Germany
^116^ Medical Science Department, iBiMED, Aveiro, Portugal
^117^ Institute for Urban Public Health, University Hospital of University Duisburg-Essen, Essen, Germany.
^118^ Department of Neurodegenerative Diseases and Geriatric Psychiatry, University of Bonn, 53127 Bonn, Germany.
^119^ German Center for Neurodegenerative Diseases (DZNE), 53127 Bonn, Germany.
^120^ Technical University of Munich, School of Medicine, Klinikum rechts der Isar, Department of Psychiatry and Psychotherapy
^121^ Taub Institute for Research in Alzheimer’s Disease and the Aging Brain, The Gertrude H. Sergievsky Center, Department of Neurology, Columbia University, New York, NY
^122^ 1st Department of Neurology, Aiginition Hospital, National and Kapodistrian University of Athens, Medical School, Greece
^123^ Department of Neurology, II B Sant Pau, Hospital de la Santa Creu i Sant Pau, Universitat Autònoma de Barcelona, Barcelona, Spain.
^124^ Department of Neurology. Hospital Universitario Donostia. San Sebastian, Spain
^125^ Neurosciences Area. Instituto Biodonostia. San Sebastian, Spain
^126^ Alzheimer’s disease and other cognitive disorders unit. Service of Neurology. Hospital Clínic of Barcelona. Institut d’Investigacions Biomèdiques August Pi i Sunyer, University of Barcelona, Barcelona, Spain
^127^ Depatamento de Especialidades Quirúrgicas, Bioquímica e Inmunología. Facultad de Medicina. Universidad de Málaga. Málaga, Spain
^128^ Neurology Service, Marqués de Valdecilla University Hospital (University of Cantabria and IDIVAL), Santander, Spain.
^129^ Department of Neurosciences. Faculty of Medicine and Nursery. University of the Basque Country, San Sebastián, Spain
^130^ Faculty of Medicine, University of Lisbon, Portugal
^131^ Memory Clinic, Department of Neurology, Charles University, 2nd Faculty of Medicine and Motol University Hospital, Czech Republic
^132^ International Clinical Research Center, St. Anne’s University Hospital Brno, Brno, Czech Republic
^133^ Unit for Hereditary Dementias, Theme Aging, Karolinska University Hospital-Solna, 171 64 Stockholm Sweden
^134^ Aging Research Center, Department of Neurobiology, Care Sciences and Society, Karolinska Institutet and Stockholm University, Stockholm, Sweden
^135^ Dept.of Public Health and Caring Sciences / Geriatrics, Uppsala University, Sweden
^136^ Department of Clinical Biochemistry, Copenhagen University Hospital – Herlev Gentofte, Denmark
^137^ Department of Clinical Medicine, University of Copenhagen, Denmark
^138^ Institute of Clinical Medicine - Neurology, University of Eastern Finland, Finland
^139^ Division of Clinical Geriatrics, Center for Alzheimer Research, Care Sciences and Society (NVS)
^140^ Karolinska Institutet, Stockholm, Sweden
^141^ Institute of Public Health and Clinical Nutrition, University of Eastern Finland, Kuopio, Finland
^142^ Neuroepidemiology and Ageing Research Unit, School of Public Health, Imperial College London, London, United Kingdom
^143^ Stockholms Sjukhem, Research & Development Unit, Stockholm, Sweden
^144^ A.I Virtanen Institute for Molecular Sciences, University of Eastern Finland, Kuopio, Finland
^145^ Normandie Univ, UNIROUEN, Inserm U1245 and Rouen University Hospital, Department of Genetics and CNR-MAJ, F 76000, Normandy Center for Genomic and Personalized Medicine, Rouen, France
^146^ Univ Lille Inserm 1171, CHU Clinical and Research Memory Research Centre (CMRR) of Distalz Lille France.
^147^ Université de Paris, EA 4468, APHP, Hôpital Broca, Paris, France
^148^ Department of Child and Adolescent Psychiatry and Psychotherapy, University Hospital of Psychiatry Zurich, University of Zurich, Zurich, Switzerland
^149^ Neuroscience Center Zurich, University of Zurich and ETH Zurich, Switzerland
^150^ Zurich Center for Integrative Human Physiology, University of Zurich, Switzerland
^151^ Old Age Psychiatry, Department of Psychiatry, Lausanne University Hospital, Lausanne, Switzerland
^152^ Department of Geriatric Psychiatry, University Hospital of Psychiatry Zürich, Zürich, Switzerland
^153^ Institute for Regenerative Medicine, University of Zürich, Switzerland
^154^ Molecular Markers Laboratory, IRCCS Istituto Centro San Giovanni di Dio Fatebenefratelli, Brescia
^155^ Neurodegenerative Diseases Unit, Fondazione IRCCS Ca’ Granda, Ospedale Policlinico, Milan, IT
^156^ Dept. of Biomedical, Surgical and Dental Sciences, University of Milan, Milan, IT
^157^ Department of Clinical Sciences and Community Health, University of Milan, 20122 Milan, Italy
^158^ Geriatric Unit, Fondazione IRCCS Ca’ Granda Ospedale Maggiore Policlinico, 20122 Milan, Italy
^159^ Institute of Gerontology and Geriatrics, Department of Medicine and Surgery, University of Perugia, Italy
^160^ Department of Biomedical Sciences, University of Cagliari, Italy.
^161^ Neurology, “San Gerardo” Hospital, Monza and University of Milano-Bicocca, Italy
^162^ Department of Neuroscience “Rita Levi Montalcini”, University of Torino, Torino, Italy
^163^ Laboratory of Neuropsychiatry, IRCCS Santa Lucia Foundation
^164^ Department of Hematology and Stem Cell Transplant, Vito Fazzi Hospital, Lecce, Italy
^165^ Division of Psychological Medicine and Clinical Neuroscience, School of Medicine, Cardiff University, Wales, UK
^166^ Department of Psychiatry and Psychotherapy, Faculty of Medicine and University Hospital Cologne, University of Cologne, Cologne, Germany
^167^ Cluster of Excellence Cellular Stress Responses in Aging-associated Diseases (CECAD), University of Cologne, Cologne, Germany
^168^ 1st Department of Neurology, Medical School, Aristotle University of Thessaloniki, Thessaloniki, Makedonia, Greece
^169^ Department of Clinical Biochemistry, Copenhagen University Hospital - Rigshospitalet, Copenhagen, Denmark
^170^ Department of Clinical Medicine, University of Copenhagen, Copenhagen, Denmark
^171^ Center for Neurosciences, Vrije Universiteit Brussel (VUB), Brussels, Belgium.
^172^ Reference Center for Biological Markers of Dementia (BIODEM), Institute Born-Bunge, University of Antwerp, Antwerp, Belgium.
^173^ Laboratory for Cognitive Neurology, Department of Neurosciences, University of Leuven, Leuven, Belgium.
^174^ Neurology Department, University Hospitals Leuven, Leuven, Belgium
^175^ Krembil Brain Institute, University Health Network, Toronto, Canada
^176^ Dept. of Medicine and Tanz Centre for Research in Neurodegenerative Diseases, University of Toronto, Canada
^177^ Fondazione IRCCS Istituto Neurologico Carlo Besta, Milan, Italy
^178^ Institute of Biomedicine, University of Eastern Finland, Finland
^179^ Institute of Psychiatry and Neurology, First Department of Neurology, Warsaw, Poland
^180^ Raffles Neuroscience Center, Raffles Hospital, Singapore
^181^ St Luke’s Hospital, Singapore, Singapore
^182^ Department of Health Promotion Sciences, Maternal and Infant Care (PROMISE), University of Palermo, Palermo, Italy
^183^ Department of Neurology, OHSU
^184^ Institute of Gerontology and Geriatrics, Department of Medicine, University of Perugia Perugia, Italy
^185^ Department of Mathematics and Statistics, Curtin University, Perth, Australia
^186^ Department of Biomedicine, Neuroscience and Advanced Diagnostics (BIND), University of Palermo, Palermo, Italy
^187^ Dementia and Parkinson’s Disease Center, University Hospital, “Paolo Giaccone”, Palermo, Italy
^188^ Institute of Psychiatry and Neurology, Department of Genetics, Warsaw, Poland
^189^ Univ Lille, Inserm, CHU Lille, France 
^190^ Lille Neuroscience & Cognition
^191^ Centre for Healthy Brain Ageing, Discipline of Psychiatry & Mental Health, School of Clinical Medicine, Faculty of Medicine and Health, University of New South Wales, Sydney, Australia
^192^ Institute of Gerontology and Geriatrics, Department of Medicine, University of Perugia Perugia (Italy)
^193^ NORMENT Centre, University of Bergen, Bergen, Norway.
^194^ Dept of Medical Genetics, Oslo University Hospital, Oslo, Norway
^195^ Clinical Psychology Service, Health Department, Fondazione IRCCS Casa Sollievo della Sofferenza, San Giovanni Rotondo (FG), Italy.
^196^ Clinical Neurosciences, Neurology, University of Helsinki and
^197^ Nuffield Department of Clinical Neurosciences, Oxford University
^198^ University of Oxford, Oxford UK
^199^ Unidad de Investigación en Epidemiología y Servicos de Salud Área Envejecimiento, Centro Medico Nacional Siglo XXI, Instituto Mexicano del Seguro Social. Ciudad de Mexico.
^200^ Translational and Clinical Research Institute, Newcastle University
^201^ Campus for Ageing and Vitality, Newcastle upon Tyne NE4 5PL, United Kingdom
^202^ Faculty of Medicine and Health Technology, Tampere University, and Department of Clinical Chemistry, Fimlab Laboratories. Tampere, Finland
^203^ INSTITUTE OF NEUROLOGY, CATHOLIC UNIVERSITY OF THE SACRED HEART, SCHOOL OF MEDICINE, LARGO A GEMELLI, 8 - 00168 ROMA - ITALY
^204^ Neuroscience Research Australia, Sydney, Australia
^205^ Department of Clinical Biochemistry, Hematology and Immunology, Na Homolce Hospital, Prague, Czech Republic
^206^ Helsinki University Hospital
^207^ University of Helsinki
^208^ Neurodegenerative Disease Unit, Department of Basic Medicine, Neuroscience, and Sense Organs, University of Bari Aldo Moro, Policlinico, Piazza Giulio Cesare 11, 70124 Bari, Italy
^209^ Geriatric Unit & Laboratory of Gerontology and Geriatrics, Department of Medical Sciences, IRCCS “Casa Sollievo della Sofferenza”, San Giovanni Rotondo, Viale Cappuccini 1, 71013 San Giovanni Rotondo, Foggia, Italy
^210^ Unit of Research Methodology and Data Sciences for Population Health, National Institute of Gastroenterology Saverio de Bellis, Research Hospital, Castellana Grotte, Bari, Italy
^211^ Department of Research and Innovation, Helse Fonna, Haugesund, Norway.
^212^ Department of Clinical Medicine (K1), University of Bergen, Bergen, Norway
^213^ Neuropsychiatric Institute, Euroa Centre, Prince of Wales Hospital, Sydney, Australia
^214^ Department of Clinical Biochemistry, Copenhagen University Hospital – Rigshospitalet, Copenhagen Denmark & Department of Clinical Medicine, Copenhagen Denmark.
^215^ Department of Neurology, Oregon Health & Science University
^216^ Department of Research and Innovation, Division of Clinical Neuroscience, Oslo University Hospital, Oslo, Norway
^217^ Institute of Clinical Medicine, Faculty of Medicine, University of Oslo, Oslo, Norway
^218^ K. G. Jebsen Center for Genetic Epidemiology, Department of Public Health and Nursing, Faculty of Medicine and Health Sciences, Norwegian University of Science and Technology (NTNU), Trondheim, Norway
^219^ Department of Internal Medicine, Division of Cardiovascular Medicine, University of Michigan, Ann Arbor, MI, 48109, USA
^220^ Department of Mental Health, Faculty of Medicine and Health Sciences, Norwegian University of Science and Technology (NTNU), Trondheim, Norway
^221^ Department of Psychiatry, Hospital Namsos, Nord-Trøndelag Health Trust, Namsos, Norway
^222^ Department of Neuromedicine and Movement Science, Faculty of Medicine and Health Sciences, Norwegian University of Science and Technology (NTNU), Trondheim, Norway
^223^ Center for Statistical Genetics, Department of Biostatistics, University of Michigan, Ann Arbor, MI, 48109, USA
^224^ Department of Clinical and Molecular Medicine, Norwegian University of Science and Technology (NTNU), Trondheim, Norway
^225^ BioCore - Bioinformatics Core Facility, Norwegian University of Science and Technology (NTNU), Trondheim, Norway
^226^ Clinic of Laboratory Medicine, St. Olavs Hospital, Trondheim University Hospital, Trondheim, Norway
^227^ Division of Mental Health Care, St. Olavs Hospital, Trondheim University Hospital, Trondheim, Norway
^228^ Department of Neurology and Clinical Neurophysiology, St. Olavs Hospital, Trondheim University Hospital, Trondheim, Norway
^229^ Research and Communication Unit for Musculoskeletal Health (FORMI), Department of Research and Innovation, Division of Clinical Neuroscience, Oslo University Hospital, Oslo, Norway
^230^ Department of Computational Medicine and Bioinformatics, University of Michigan, Ann Arbor, MI, 48109, USA
^231^ Analytic and Translational Genetics Unit, Massachusetts General Hospital, Boston, MA, USA
^232^ Bordeaux University Hospital, Department of Medical Informatics, F-33000 Bordeaux, France
^233^ Department of Neurology, School of Medicine, University of Pittsburgh, PA, USA
^234^ Department of Epidemiology & Biostatistics, University of California, San Francisco, California 94158, USA
^235^ Department of Neurology, Erasmus University Medical Centre, Rotterdam, Netherlands
^236^ Department of Clinical Genetics, Erasmus MC, Rotterdam, the Netherlands
^237^ Department of Radiology and Nuclear Medicine, Erasmus MC, Rotterdam, the Netherlands
^238^ Department of Psychology, Latin American Brain Health (BrainLat), Universidad Adolfo Ibáñez, Santiago, Chile
^239^ Norwegian National Advisory Unit on Ageing and Health, Vestfold Hospital Trust, Tønsberg, Norway
^240^ Department of Geriatric Medicine, Oslo University Hospital, Oslo, Norway
^241^ HUNT Research Center, Department of Public Health and Nursing, Faculty of Medicine and Health Sciences, Norwegian University of Science and Technology (NTNU), Trondheim, Norway
^242^ Department of Research, Innovation and Education, St. Olavs Hospital, Trondheim University Hospital, Trondheim, Norway
^243^ Division of Mental Health and Addiction, Oslo University Hospital, Oslo, Norway
^244^ NORMENT, University of Oslo, Oslo, Norway
^245^ Division of Neurogenetics and Molecular Psychiatry, Department of Psychiatry and Psychotherapy, Faculty of Medicine and University Hospital Cologne, University of Cologne, Cologne, Germany
^246^ Department of Neurodegenerative Diseases and Geriatric Psychiatry, University Hospital Bonn, Medical Faculty, Bonn, Germany
^247^ Department of Psychiatry & Glenn Biggs Institute for Alzheimer’s and Neurodegenerative Diseases, San Antonio, TX, USA
^248^ Alzheimer Center Amsterdam, Department of Neurology, Amsterdam Neuroscience, Vrije Universiteit Amsterdam, Amsterdam UMC, Amsterdam, The Netherlands
^249^ CHU de Bordeaux, Department of Neurology, Institute for Neurodegenerative Diseases, F-33000 Bordeaux, France
^250^ Institute of Molecular Medicine, McGovern Medical School,, The University of Texas Health Science Center at Houston, Houston TX, USA
^251^ Department of Research, Innovation and Education, Division of Clinical Neuroscience, Oslo University Hospital, Oslo, Norway
^252^ Department of Neurology, Oslo University Hospital, Oslo, Norway
^253^ Translational Health Sciences, Bristol Medical School, University of Bristol, Bristol, UK
^254^ School of Public Health, Faculty of Social Welfare and Health Sciences, University of Haifa, Haifa, Israel
^255^ Framingham Heart Study, MA, USA
^256^ Department of Neurology, UT Health San Antonio, 7703 Floyd Curl Drive, San Antonio, TX, USA
^257^ Department of Neurology, Boston University School of Medicine, Boston, Massachusetts, USA

* These authors contributed equally.

# corresponding authors:

Bernard Fongang: [fongang@uthscsa.edu](mailto:fongang@uthscsa.edu)

Galit Weinstein: gweinstei@univ.haifa.ac.il

Sudha Seshadri: [Seshadri@uthscsa.edu](mailto:Seshadri@uthscsa.edu)

**Supplementary File 2**

**Study Description and Acknowledgments**

Table of Contents

[Introduction 11](#_Toc124913492)

[1 – Study description 11](#_Toc124913493)

[1 – 1. AGES-Reykjavik Study (AGES) 11](#_Toc124913494)

[1 – 2. The Atherosclerosis Risk in Communities Study (ARIC) 13](#_Toc124913495)

[1 – 3. The ASPirin in Reducing Events in the Elderly (ASPREE) 14](#_Toc124913496)

[1 – 4. The Cardiovascular Health Study (CHS) 16](#_Toc124913497)

[1 – 5. European Alzheimer's Disease DNA BioBank (EADB) 19](#_Toc124913498)

[1 – 6. The Framingham Heart Study (FHS) 35](#_Toc124913499)

[1 – 7. INGI-Friuli Venezia Giulia (INGI-FVG) 39](#_Toc124913500)

[1 – 8. The Genome Research at ACE Alzheimer Center Barcelona (GR@ACE) 40](#_Toc124913501)

[1 – 9. The Salus in Apulia Study (SAS) 42](#_Toc124913502)

[1 – 10. The Hong Kong Osteoporosis Study (HKOS) 44](#_Toc124913503)

[1 – 11. The Nord-Trøndelag Health Study (HUNT) 45](#_Toc124913504)

[1 – 12. The MEMENTO cohort 48](#_Toc124913505)

[1 – 13. The Monongahela-Youghiogheny Healthy Aging Team (MYHAT) 49](#_Toc124913506)

[1 – 14. The Religious Orders Study and Memory and Aging Project (ROSMAP) 51](#_Toc124913507)

[1 – 15. The Rotterdam Study (RS1, RS2, RS3) 52](#_Toc124913508)

[1 – 16. The San Antonio Longitudinal Study of Aging (SALSA) 54](#_Toc124913509)

[1 – 17. HARMONIZATION 55](#_Toc124913510)

[1 – 18. Three-City (3C) 56](#_Toc124913511)

[1 – 19. The UK Biobank (UKBB) 58](#_Toc124913512)

[1 – 20. Accessed GWAS summary statistics 59](#_Toc124913513)

[2 - ICD9-10 codes used by cohorts in this project 60](#_Toc124913514)

[3 - Software availability 61](#_Toc124913515)

[4 – Additional Information for EADB 61](#_Toc124913516)

[4 – 1. Supplementary list of authors EADB 62](#_Toc124913517)

[4 – 2. Supplementary list of authors FinnGen 69](#_Toc124913518)

[4 – 3. Additional Support for EADB cohorts 71](#_Toc124913519)

[REFERENCES 76](#_Toc124913520)

# Introduction

This project was conducted within the neurology working group of the Cohorts for Heart and Aging Research in Genomic Epidemiology (*CHARGE*) Consortium. The CHARGE cohorts are supported in part by the National Heart, Lung, and Blood Institute (NHLBI) infrastructure grants R01HL105756 (Psaty), RC2HL102419 (Boerwinkle) and the neurology working group is supported by the National Institute on Aging (NIA) R01 grant AG033193.

# **1 – Study Description**

## **1 – 1. AGES-Reykjavik Study (AGES)**

The AGES-Reykjavik Study is a single center prospective cohort study based on the Reykjavik Study. The Reykjavik Study was initiated in 1967 by the Icelandic Heart Association to study cardiovascular disease and risk factors. The cohort included men and women born between 1907 and 1935 who lived in Reykjavik at the 1967 baseline examination. Reexamination of surviving members of the cohort was initiated in 2002 as part of the AGES-Reykjavik Study. AGES is designed to investigate aging using a multifaceted comprehensive approach that includes detailed measures of brain function and structure. All cohort members were European Caucasians. The study design has been described previously.1 Briefly, as part of a comprehensive examination, all participants answered a questionnaire, underwent a clinical examination, multiple digital measurements were acquired, and blood was drawn.

**All-cause and vascular dementia ascertainment.**

The dementia case finding was based on a 3-step procedure. All participants were screened on the Mini-Mental State Examination and DSST. Screen positives on either of the tests were administered another more complete diagnostic test battery. Those screening positive on the Trails A and B or the Rey Auditory Verbal Learning Test went for a final assessment that included a proxy interview and a neurologic examination. The diagnosis of dementia and subtypes was made during a consensus conference that included a geriatrician, a neurologist, a neuropsychologist, and a neuroradiologist who provided a clinical reading of MRI. Dementia was diagnosed according to the guidelines of the DSM-IV. Alzheimer disease (AD) was diagnosed according to the criteria of the National Institute of Neurological and Communicative Diseases and Stroke–Alzheimer’s Disease and Related Disorders Association. Vascular dementia (VaD) was diagnosed following the criteria of the State of California AD Diagnostic and Treatment Centers; Clinical medical history and MRI were used in the diagnosis. It was possible to diagnose a subject with possible AD and possible VaD if the 2 pathologies were thought to contribute to dementia.

**Genotyping, quality control and imputation.**

Within the AGES cohort, 3219 individuals were genotyped with the Illumina hu370CNV array, and 2,705 individuals genotyped with the Illumina Infinium Global Screening Array. Data from both genotype arrays underwent quality control procedure, separately, removing variants with call rate <95% and HWE P-value < 1 × 10−6. Both arrays were imputed against the Haplotype Reference Consortium imputation panel r1.1 with the Minimac3 software.^1^ Post-imputation quality control consisted of filtering out variants with imputation quality R2 < 0.7, MAF < 0.01, as well as monomorphic and multiallelic variants for each platform separately. Genotypes for remaining variants, with matching location and alleles between platforms, were merged to create a dataset with 7,506,463 variants for 5656 individuals (268 individuals were genotyped on both platforms, with a 99% match of genotypes for the final set of variants between platforms). The quality control procedure was performed using bcftools (v1.9) and PLINK 1.9.^2,3^ All positions are based on genome assembly GRCh37

**Association testing**.

Association testing was performed with PLINK 1.9 adjusting for age, sex and the first five principal components.^3^

**Funding**.

AGES is funded by the National Institute on Aging (NIA) (N01-AG-12100), Hjartavernd (the Icelandic Heart Association), and the Althingi (the Icelandic Parliament), with contributions from the Intramural Research Programs at the NIA and at the National Heart, Lung, and Blood Institute (Z01 HL004607-08 CE). The study was approved by the Icelandic National Bioethics Committee (VSN: 00-063) and the MedStarResearch Institute (project 2003-145).

## **1 – 2. The Atherosclerosis Risk in Communities Study (ARIC)**

The ARIC study is a prospective population-based study of atherosclerosis and clinical atherosclerotic diseases in 15,792 men and women, including 11,478 white participants, drawn from four United States communities (Suburban Minneapolis, Minnesota; Washington County, Maryland; Forsyth County, North Carolina; and Jackson, Mississippi). In the first three communities, the sample reflects the demographic composition of the community. Since the baseline exam, there have been five subsequent follow-up visits with neurocognitive functioning assessed in visit 2 (1990-1992), visit 4 (1996-1998), visit 5 (2011-2013), and visit 6 (2016 – 2017). In Jackson, only black residents were enrolled. Participants were between age 45 and 64 years at their baseline examination in 1987-1989 when blood was drawn for DNA extraction and participants consented to genetic analysis.

**All-cause and vascular dementia ascertainment.**

Cognitive diagnoses were adjudicated at the fifth visit using cognitive, neurologic, and brain imaging assessments (comprehensive diagnostic details are available elsewhere^4^). Cognitive status was not available on the whole sample at the third visit. The ARIC study has been approved by the Institutional Review Board at each field center, including Wake Forest Baptist Medical Center (Forsyth County, NC), University of Mississippi Medical Center (Jackson, MS), University of Minnesota (suburban Minneapolis, MN), and Johns Hopkins University (Washington County, MD). Participants provided written informed consent prior to each examination.

**Genotyping, quality control and imputation and association testing**.

At baseline, blood was drawn for DNA extraction and participants consented to genetic testing. Genome-wide genotyping was conducted at the Broad Institute using the Affymetrix 6.0 SNP Array. Genotyping calling was performed using Birdseed for 9,747 European Americans. Imputation was performed on the QCed data on the Michigan Imputation Server in two steps: pre-phasing with EAGLE and imputation with MiniMac3, using the HRC r1.1 data reference panel.

**Funding**.

ARIC is funded in whole or in part with Federal funds from the National Heart, Lung, and Blood Institute, National Institutes of Health, Department of Health and Human Services, under Contract nos. (HHSN268201700001I, HHSN268201700002I, HHSN268201700003I, HHSN268201700005I, HHSN268201700004I). The authors thank the staff and participants of the ARIC study for their important contributions. Funding support for “Building on GWAS for NHLBI-diseases: the U.S. CHARGE consortium” was provided by the NIH through the American Recovery and Reinvestment Act of 2009 (ARRA) (5RC2HL102419). This project was funded from R01-NS087541 to Myriam Fornage and Eric Boerwinkle.

## **1 – 3. The ASPirin in Reducing Events in the Elderly (ASPREE)**

The ASPirin in Reducing Events in the Elderly (ASPREE) trial was a randomised placebo-controlled clinical trial of daily 100mg low-dose aspirin versus placebo in healthy older people. The design, recruitment, and baseline characteristics of the ASPREE study have been published previously.^5,6^ At enrolment, ASPREE participants had no previous history or current diagnosis of atherothrombotic cardiovascular disease, dementia, loss of independence with basic activities of daily living, or life-threatening illness. Participants passed a global cognition screen at enrollment (>77 on the Modified Mini-Mental State (3MS) Examination). Informed consent for genetic analysis was obtained from all participants who provided a biospecimen to the ASPREE Healthy Ageing Biobank, with ethical approval from the Alfred Hospital Human Research Ethics Committee (390/15) and site-specific Institutional Review Boards (US).

**All-cause and vascular dementia ascertainment.**

After standardized cognition and functional measures, ASPREE participants reporting memory or cognitive problems were assessed by specialists or prescribed dementia medication (in Australia). Following identification of dementia triggers (3MS<78 or a drop of >10.15 points from the participant’s baseline 3MS score, accounting for age and education), additional assessments were conducted, with brain imaging and laboratory analyses collected for adjudication. Each dementia trigger case was reviewed according to the ASPREE protocol for clinical adjudication^5,6^ by an adjudication committee consisting of geriatricians, neurologists and neuropsychologists. Dementia was diagnosed using Diagnostic and Statistical Manual of Mental Disorders, fourth edition criteria. Diagnosis date was recorded as date of trigger. Dementia cases were sub-classified into either ‘probable AD’, ‘possible AD’or ‘non-AD dementia’, using the 2011 NIA-Alzheimer’s Association core clinical criteria ^7^.

**Genotyping, quality control, imputation, and association testing.**

Genotyping of DNA samples provided by ASPREE participants was performed on the Axiom 2.0 Precision Medicine Diversity Research Array (Thermo Fisher Scientific (TFS), Waltham, MA, USA) following standard protocols. Variants were aligned to the human genome reference GRCh38. Participants with European ancestry were included to minimize the effect of population stratification. To estimate population structure, we performed principal component analysis using the 1000 Genomes reference population (Figure S2)^8,9^. Imputation was performed using the haplotype reference consortium European panel^1^. Post-imputation quality control removed variants r2<0.3. *APOE* genotype was measured using two directly genotyped variants (rs7412, rs429358) extracted using plink v1.9^3^. Association analysis was performed following the pre-defined CHARGE dementia GWAS analysis plan.

**Funding**.

This work was supported by an ASPREE Flagship cluster grant (including the Commonwealth Scientific and Industrial Research Organization, Monash University, Menzies Research Institute, Australian National University, University of Melbourne); and grants (U01AG029824 and U19AG062682) from the National Institute on Aging and the National Cancer Institute at the National Institutes of Health, by grants (334047 and 1127060) from the National Health and Medical Research Council of Australia, and by Monash University and the Victorian Cancer Agency. Paul Lacaze is supported by a National Heart Foundation Future Leader Fellowship (102604).

## **1 – 4. The Cardiovascular Health Study (CHS)**

The Cardiovascular Health Study (CHS) is a population-based cohort study of risk factors for coronary heart disease and stroke in adults ≥65 years conducted across four field centers in the United States: Sacramento County, California; Washington County, Maryland; Forsyth County, North Carolina; and Pittsburgh, Allegheny County, Pennsylvania. The original predominantly European ancestry cohort of 5,201 persons was recruited in 1989-1990 from random samples of the Medicare eligibility lists; subsequently, an additional predominantly African American cohort of 687 persons was enrolled for a total sample of 5,888. Blood samples were drawn from all participants at their baseline examination and DNA was subsequently extracted from available samples. CHS was approved by institutional review committees at each field center and individuals in the present analysis had available DNA and gave informed consent including consent to use of genetic information for the study of cardiovascular disease.

**All-cause and vascular dementia ascertainment.**

The Alzheimer’s disease sample for this study included all prevalent cases identified in 1992 and incident events identified between 1992 and December 2006^10^. Briefly, persons were examined annually from enrollment to 1999, and the examination included a 30-minute screening cognitive battery. In 1992-94 and again in 1997-99, participants were invited to undergo brain MRI and detailed cognitive and neurological assessment as part of the CHS Cognition Study. Persons with prevalent dementia were identified, and all others were followed until 1999 for the development of incident dementia and Alzheimer’s disease. Since then, CHS participants at the Maryland and Pennsylvania centers have remained under ongoing dementia surveillance^11^.

Beginning in 1988/89, all participants completed the Modified Mini-Mental State Examination (3MSE) and the DSST at their annual visits, and the Benton Visual Retention Test (BVRT) from 1994 to 1998. The Telephone Interview for Cognitive Status (TICS) was used when participants did not come to the clinic. Further information on cognition was obtained from proxies using the Informant Questionnaire for Cognitive Decline in the Elderly (IQCODE), and the dementia questionnaire (DQ). Symptoms of depression were measured with the modified version of the Center for Epidemiology Studies Depression Scale (CES-D). In 1991-94, 3608 participants had an MRI of the brain and this was repeated in 1997-98. The CHS staff also obtained information from participants and next-of-kin regarding vision and hearing, the circumstances of the illness, history of dementia, functional status, pharmaceutical drug use, and alcohol consumption. Data on instrumental activities of daily living (IADL), and activities of daily living (ADL) were also collected.

Persons suspected to have cognitive impairment based on the screening tests listed above underwent a neuropsychological and a neurological evaluation. The neuropsychological battery included the following tests: the American version of the National Reading test (AMNART), Raven’s Coloured Progressive Matrices, California Verbal Learning Test (CVLT), a modified Rey-Osterreith figure, the Boston Naming test, the Verbal fluency test, the Block design test, the Trails A and B tests, the Baddeley & Papagno Divided Attention Task, the Stroop, Digit Span and Grooved Pegboard Tests. The results of the neuropsychological battery were classified as normal or abnormal (>1.5 standard deviations below individuals of comparable age and education) based on normative data collected from a sample of 250 unimpaired subjects. The neurological exam included a brief mental status examination, as well as a complete examination of other systems. The examiner also completed the Unified Parkinson’s Disease Rating Scale (UPDRS) and the Hachinski Ischemic Scale. After completing the neurological exam, the neurologist classified the participant as normal, having mild cognitive impairment (MCI), or dementia. International diagnostic guidelines, including the NINCDS-ADRDA criteria for probable and possible Alzheimer’s disease and the ADDTC’s State of California criteria for probable and possible vascular dementia (VaD) with or without Alzheimer’s disease, were followed. CHS identified 3 subtypes: possible/probable Alzheimer’s disease without VaD (categorized as pure Alzheimer’s disease, included in all Alzheimer’s disease) and mixed Alzheimer’s disease (for cases that met criteria for both Alzheimer’s disease and VaD, included in all-Alzheimer’s disease), and, possible/probable VaD without Alzheimer’s disease (excluded from current study). For this study, CHS contributed data on 450 Alzheimer’s disease cases and 1,702 healthy controls with Alzheimer’s disease-free status confirmed as of most recent follow- up.

**Genotyping, quality control, imputation, and association testing.**

Genotyping was performed at the General Clinical Research Center’s Phenotyping/Genotyping Laboratory at Cedars-Sinai among CHS participants who consented to genetic testing and had DNA available using Illumina 370CNV BeadChip for European ancestry and Illumina HumanOmni1-Quad_v1 BeadChip for African ancestry. All African ancestry with available DNA and appropriate consent were genotyped. European ancestry participants with presence at study baseline of coronary heart disease, congestive heart failure, peripheral vascular disease, valvular heart disease, stroke or transient ischemic attack or lack of available DNA were excluded from the GWAS study sample. Beyond laboratory genotyping failures, participants were excluded if they had a call rate<=95% or if their genotype was discordant with known sex or prior genotyping (to identify possible sample swaps). After quality control, genotyping was successful for 3,268 European ancestry and 823 African American participants. The following exclusions were applied to identify a final set of autosomal SNPs: call rate < 97%, HWE P < 10-5, > 2 duplicate errors or Mendelian inconsistencies (for reference CEPH trios), heterozygote frequency = 0, SNP not found in HapMap. Imputation to the HRC r1.1 2016 panel was performed on the Michigan imputation server. SNPs were excluded for variance on the allele dosage ≤0.01.

**Funding**.

This CHS research was supported by NHLBI contracts HHSN268201200036C, HHSN268200800007C, HHSN268201800001C, N01HC55222, N01HC85079, N01HC85080, N01HC85081, N01HC85082, N01HC85083, N01HC85086, 75N92021D00006; and NHLBI grants U01HL080295, R01HL087652, R01HL105756, R01HL103612, R01HL120393, and U01HL130114 with additional contribution from the National Institute of Neurological Disorders and Stroke (NINDS). Additional support was provided through R01AG023629, R01AG20098, R01AG15928, and R01AG033193 from the National Institute on Aging (NIA). A full list of principal CHS investigators and institutions can be found at CHS-NHLBI.org.

The provision of genotyping data was supported in part by the National Center for Advancing Translational Sciences, CTSI grant UL1TR001881, and the National Institute of Diabetes and Digestive and Kidney Disease Diabetes Research Center (DRC) grant DK063491 to the Southern California Diabetes Endocrinology Research Center.

.

## **1 – 5. European Alzheimer's Disease DNA BioBank (EADB)**

The European Alzheimer’s Disease Biobank (EADB)^12^ dataset consortium groups together AD cases and controls from 15 European countries (Belgium, Bulgaria, Czech Republic, Denmark, Finland, France, Germany, Greece, Italy, Portugal, Spain, Sweden, Switzerland, The Netherlands and the UK). These samples were genotyped in three independent centers (France, Germany and the Netherlands) as described below.

**All-cause and vascular dementia ascertainment**

This study was conducted within the Vascular Cognitive Impairment Genetics Consortium (VCIGC).

The Vascular Cognitive Impairment Genetics Consortium (VCIGC) was initially set up 2010-2013 by The Dementia Research Group, University of Bristol, to undertake pilot investigations into the genetic basis of forms of Vascular Cognitive Impairment (VCI).^13^ The resultant collaborative collection consists of several thousand cases (vascular dementia, post-stroke dementia or mixed vascular dementia and Alzheimer’s disease) and controls from a number of UK and International Centres with an intended use for further research into the genetic aetiology of VCI.

VCIGC cohort synopses:

**COHORT: BED**

**PI:** Prof. Malgorzata Bednarska-Makaruk; Institute of Psychiatry and Neurology, Department of Genetics, Warsaw, Poland

**Clinical diagnostic criteria:** AD: NINCDS-ADRDA; VaD: NINDS-AIREN; AD+VaD: NINCDS-ADRDA for AD + vascular changes in neuroimaging; vascular MCI: the Petersen criteria for MCI + vascular changes in neuroimaging

**COHORT: BOR**
**PI:** Prof. Regis Bordet; CHRU Lille, France
**Resource:** Clinical cohort study: Study of Factors Influencing Post-stroke Dementia **(STROKDEM)**

**Description:** The STROKDEM study is based on the 5-year prospective follow-up of a population of stroke patients without dementia, who were over 40 years old, displaying an hemorrhagic or an ischemic stroke, with a sus-tentorial localization, and included 72h before the onset of symptoms. At inclusion in the cohort, main antecedents and risk factors, previous treatment and lifestyle, initial severity and etiology are recorded. Clinical severity of stroke is assessed according to the National Institute of Health Stroke Scale (NIHSS). Biological samples (for standard and specialized analyses) and Magnetic Resonance Imaging (MRI) are performed at 72h after stroke occurrence. Thereafter, patients are regularly (6 months, 12 months, 36 months, 60 months) examined for clinical and cognitive assessment with biological samples and MRI. This study procedure was approved by the local ethics committee and registered on clinicaltrials.gov ([NCT01330160](https://clinicaltrials.gov/ct2/show/NCT01330160)). Patients gave written informed content.

**Clinical diagnostic criteria:** The cognitive functions of participants were assessed at M6, M12, M36, M60 post-stroke with a battery of neuropsychological tests, broadly classified into four cognitive domains (executive functions/attention, memory, language, visuospatial abilities). Tests used in the assessment of executive function/attention were Trail Making Test part A and B, a version of Stroop paradigm, and the subtest "code" from WAIS III. For the memory domain, total of the 3 free recalls trials and delayed free recall from the Free and Cued Selective Reminding Test (FCSR) and score of delayed recall from Rey complex figure test were used. The language domain score was built using scores from DO 80, semantic fluency (animal), phonemic fluency (p words) tests. Incomplete letter and number location subtests from VOSP and copy of Rey complex figure test were used in order to assess visuospatial abilities. For every subject, test-specific z-scores based on available norms corrected for age, sex, and education were calculated. We further obtained summary domain-specific z-scores by averaging the test-specific z-scores in each domain. Following this neuropsychological assessment, participants were diagnosed for a cognitive impairment at 6- and 36-months post-stroke, using a summary z-score ≤1.5 in at least one of the four domains as diagnostic threshold.^14-17^

**COHORT: CHE**

**PI:** Prof. Christopher Chen; National University of Singapore

**Resource:** Clinical cohort study - The Memory Aging and Cognition Centre **(MACC,** [*http://www.macc.sg/MACC-Publications-Memory-Problems-Dementia-Prevention*](http://www.macc.sg/MACC-Publications-Memory-Problems-Dementia-Prevention)**)**

**Study description:** The Memory Aging and Cognition Centre (MACC) cohort has a longitudinal case-control design. Cases were recruited from memory clinics in the National University Hospital and Saint Luke’s Hospital, Singapore. Cognitively normal controls were recruited from both memory clinics and the community in the same catchment area. Ethics approval for this study was obtained from the National Healthcare Group Domain-Specific Review Board (DSRB) (DSRB reference: 2010/00017; study protocol number: DEM4233). Subjects are assessed annually for up to 5 years.

**Clinical diagnostic criteria:** The diagnosis of AD was based on the National Institute of Neurological and Communicative Disorders and Stroke and the Alzheimer’s Disease and Related Disorders Association (NINCDS-ADRDA) criteria, whereas VaD was diagnosed using the National Institute of Neurological Disorders and Stroke and Association Internationale pour la Recherché et l’ Enseignement en Neurosciences (NINDS-AIREN) criteria. Subjects who fulfilled the NINCDS-ADRDA criteria for AD but also showed significant cerebrovascular disease on neuroimaging scans (defined as the presence of cortical infarct and/or presence of >=2 lacunes and/or confluent white matter hypertension (ARWMC score >=8) were classified as AD with CVD. No cognitive impairment was diagnosed when subjects showed no objective cognitive impairment in any of the seven cognitive domains tested.^18^

**Acknowledgements:** This work is supported by the National Medical Research Council of Singapore (NMRC/CG/NUHS/2010, NMRC/CG/013/2013, NMRC/CIRG/1446/2016, NMRC/CSA-SI/0007/2016, NMRC/CG/M009/2017_NUH/NUHS, NMRC/CIRG/1485/2018 and MOH-000707-00).

**COHORT: ERK**
**PI:** Prof. Timo Erkinjuntti; Clinical Neurosciences, Neurology, University of Helsinki and Helsinki University Hospital, Finland
**Resource:** Clinical cohort study - Helsinki Stroke Aging Memory **(SAM)**

**Description:** The cohort consists of 486 ischemic stroke patients aged 55 to 85 years admitted consecutively to the Helsinki University Central Hospital (Finland) between December 1, 1993 - March 31, 1995. Cases were examined 3 months after the index stroke. Structured medical, neurological, and radiological (MRI or CT) examinations, mental status, and emotional examination, as well as the Mini-Mental State Examination and detailed clinical mental status examination of defined cognitive domains. Normative values for each cognitive domain were based on a random Finnish-speaking healthy community sample for those under and over 75 years of age. Prestroke and poststroke activities of daily living were assessed with five scales. Interview of a close informant was made also. Types of ischemic stroke were classified according to the TOAST criteria into large-artery atherosclerosis, cardioembolism, small-vessel occlusion (lacunar), and stroke of other determined or undetermined etiology. At 15 months the functional status and depression status were assessed. The patients were followed up to 12 years using hospital registers and mortality statistics.^19^

**Clinical diagnostic criteria:** The criteria for dementia were those of the Diagnostic and Statistical Manual of Mental Disorders (DSM) III

**Acknowledgements:** The study was supported by grants from the Clinical Research Institute, University of Helsinki and the Medical Research Fund of the Helsinki University Central Hospital.

**COHORT: ESI**
**PI:** Prof. Margaret Esiri; Nuffield Department of Clinical Neurosciences, Oxford University
**Resource:** Clinical Cohort study - Oxford Project to Investigate Memory and Ageing **(OPTIMA)**; Oxford Brain Bank (https://www.ndcn.ox.ac.uk/research/centre-prevention-stroke-dementia/resources/optima-oxford-project-to-investigate-memory-and-ageing)

**Description:** The Oxford project to investigate memory and ageing (OPTIMA) was a longitudinal study established by Professors David Smith (Pharmacology) and Margaret Esiri (Neuropathology) in 1988 with the prime purpose of advancing an understanding of the causes, treatment and prevention of dementia, and to develop methods of diagnosing the diseases responsible for it. Professor Gordon Wilcock (Geratology) succeeded as director in 2008, and established the LEAD cohort, which undertook the final follow-up of the last participant in March 2015. Over the course of OPTIMA’s existence, clinical, cognitive and imaging data along with tissue samples were collected annually from more than 1,100 patients with dementia, their spouses or carers, and age-matched controls. Most remarkably, over 500 of these subjects donated their brains for research after their deaths, making this an exceptional resource. Although it is not a population-based cohort, the OPTIMA cohort is ideally suited to case-control studies and a number of novel genetic and non-genetic risk factors have been discovered. The data continue to have great value for mechanistic and biomarker discovery and experimental medicine. More than 250 publications have made use of this resource.

**Clinical diagnostic criteria:** MCI was diagnosed using the Peterson criteria (Peterson RC et al Neurology 2001; 156: 1133-42; AD was diagnosed based on the NINCDS-ARDRA criteria, and the NINDS-AIREN criteria was used for the diagnosis of vascular dementia.

**Acknowledgements:** Oxford samples are part of the NIHR Oxford Biomedical Research Centre supported Oxford Project to Investigate Memory and Ageing (OPTIMA) study. We acknowledge the Oxford Brain Bank, supported by the Medical Research Council (MRC), Brains for Dementia Research (BDR) (Alzheimer Society and Alzheimer Research UK), Autistica UK and the NIHR Oxford Biomedical Research Centre.

**COHORT: HOR**

**PI:** Prof. Jakub Hort; Memory Clinic, Department of Neurology, Motol University Hospital, Prague, Czech Republic

**Resource:** The Czech Brain Aging Study **(CBAS,** www.cbas.cz**)**

**Description:** Longitudinal memory clinic based study recruiting subjects at risk of dementia (subjects referred for cognitive complaints - SCD, MCI), CBAS + study - crossectional study of patients in early stages of dementia.

**Clinical diagnostic criteria:** Cognitively healthy elderly with no significant memory complaint, recruited from patients and staff relatives, advertisement and among 3rd age University participants, age and education matched to CBAS cohort. McKhann 2011 - probable AD dementia with intermediate or high evidence of AD pathophysiological process. bvFTD-Rascovsky 2011. Participants with objective cognitive decline classified as mild cognitive impairment (MCI) based National Institute on Aging and Alzheimer's Association guidelines by Albert et al 2011.^20^

**Acknowledgements:**   Supported by the project no. LQ1605 from the National Program of Sustainability II (MEYS CR), Supported by Ministry of Health of the Czech Republic, grant nr. NV19-04-00270 (All rights reserved), Grant Agency of Charles University Grants No. 693018 and 654217; the Ministry of Health, Czech Republic―conceptual development of research organization, University Hospital Motol, Prague, Czech Republic Grant No. 00064203; the Czech Ministry of Health Project AZV Grant No. 16―27611A; and Institutional Support of Excellence 2. LF UK Grant No. 699012

**COHORT: KAL**
**PI:** Prof. Raj Kalaria; Translational and Clinical Research Institute and The Newcastle Brain Tissue Resource, Newcastle University
**Resource:**  Cognitive Function After Stroke **(CogFAST)** study comprising cohort study derived from North East UK Hospitals Stroke Registers

**Description:** Older stroke patients (n=706) ≥75 years were screened consecutively from hospital-based stroke registers in Tyneside and Wearside in the North East of England. Potential participants were evaluated at least 3 months after first stroke and to enable resolution of acute post stroke delirium with a standardized battery comprised of medical history, MMSE score, assessment of neurological deficits, a blood screen, and review of CT brain scan undertaken at the time of the stroke. Medical histories taken from the participants were supported by review of hospital charts for diagnoses of previous stroke (including whether there was any residual disability from previous stroke), hypertension (a documented history of blood pressure greater than 140/90 mm Hg or treatment of hypertension), atrial fibrillation (AF), ischaemic heart disease (IHD), peripheral vascular disease, hypercholesterolaemia, diabetes (documented or treated) and history of smoking prior to stroke. Follow up was for at least 20 years. As of December 2019, 96 brain donations were received.

**Clinical diagnostic criteria:** Stroke was defined according to the World Health Organization definition and classified according to the Oxford Community Stroke project classification. Participants with a total CAMCOG score < 80 were defined as having cognitive impairment or dementia. The Clinical Dementia Rating (CDR) scale and IQCODE (informant) scores were obtained. Final dementia diagnosis was made when participant met the DSM IV criteria. The apolipoprotein genotype for each participant was determined.

**Case selection criteria:** All autopsies from post-stroke survivors were included. About ~50% developed dementia prior to death. 75% of the cases were diagnosed with VaD in the absence of any significant neurodegenerative pathology or disease.

**Publication reference:** Allan LM et al, Brain 2011; Allan LM et al, British Journal of Psychiatry 2013; Firbank MJ et al, Journal of Neurology Neurosurgery and Psychiatry 2012.

**Acknowledgements:** The Newcastle Brain Tissue Resource is funded in part by: a grant from the UK Medical Research Council (G0400074), by NIHR Newcastle Biomedical Research Centre and Unit funding awarded to the Newcastle upon Tyne NHS Foundation Trust and Newcastle University, by the Brains for Dementia Research Programme, which is itself jointly funded by Alzheimer’s Research UK and Alzheimer’s Society.

**COHORT: KEH**

**PI:** Profs Patrick Gavin Kehoe & Seth Love; Dementia Research Group, Bristol Medical School (THS), University of Bristol, UK
**Resource:** Clinical cohort study - Memory Disorders Clinic **(MDC)**; South West Dementia Brain Bank **(SWDBB)**

**Description:** The former North Bristol NHS Trust Memory Disorders Clinic (MDC) at Blackberry Hill Hospital, Bristol (BRACE Clinic) collected data over a period of approximately twenty years (November 1985 through to July 2006) on those who attended for assessment and diagnosis. Data from patients who consented were collated in a comprehensive database, ©Bristol Dementia Research Group September 2006, for research use. Samples of DNA from patients of the now−closed BRACE Clinic were obtained and stored as part of the South West Dementia Brain Bank (SWDBB) Research Tissue Bank. The SWDBB also provided DNA samples for this study, under local Research Ethics Committee approval. The SWDBB began receipt of brain donations for dementia research in the mid-1980s and in 2010 became one of the recruitment centres and member brain banks in the ABBUK-funded Brains for Dementia Research (BDR) Network project.

**Clinical diagnosis:** MDC patients with a final clinical diagnosis of vascular dementia according to NINDS-AIREN criteria were selected for this study. The clinical diagnosis of dementia was made by experienced clinicians using standard criteria as specified in the Diagnostic and Statistical Manual of Mental Disorders Fourth Edition (DSM-IV).

**Post-mortem diagnosis:** This was based on a combination of clinical history and neuropathological findings. National Institute on Aging-Alzheimer's Association guidelines (Montine et al., 2012) were utilised to ascertain the likelihood that Alzheimer’s disease (AD) neuropathological changes were a sufficient explanation for the dementia. The pure vascular dementia cases had no more than occasional neuritic plaques, a Braak tangle stage of III or less, histopathological evidence of multiple infarcts/ischaemic lesions, moderate to severe atheroma and/or arteriosclerosis, and an absence of histopathological evidence of other disease likely to cause dementia. The AD cases with concomitant vascular dementia had, in addition to histopathological evidence of multiple infarcts/ischaemic lesions and moderate to severe atheroma and/or arteriosclerosis, neurofibrillary tangle and neuritic and Aβ plaque pathology of sufficient severity to indicate a high likelihood that AD neuropathological change would itself have been a sufficient explanation for the dementia. The normal controls had no clinical history of dementia, few or no neuritic plaques, and no other neuropathological abnormalities.

**Acknowledgements:** We would like to thank the South West Dementia Brain Bank (SWDBB) for providing DNA for this study. The SWDBB is part of the Brains for Dementia Research programme, jointly funded by Alzheimer’s Research UK and Alzheimer’s Society and is supported by BRACE (Bristol Research into Alzheimer’s and Care of the Elderly) and the Medical Research Council.

**COHORT: MAS**

**PI:** Prof. Carlo Masullo; Department of Neuroscience, Section of Neurology, Catholic University of the Sacred Heart, Roma, Italy.

**Resource**: **UCSC** Cohort

**Descrition**: UCSC cohort was gathered by enrolling subjects as they entered the outpatient neurogeriatrics unit at the ‘Fondazione Policlinico Universitario A. Gemelli IRCCS’, Catholic University of the Sacred Heart, Roma, Italy. Subjects have been referred for cognitive assessment. Each subject underwent a complete neurological examination and a standardized neuropsychological battery (Caltagirone et Al, 1979) including MMSE, ADL and IADL scores. We selected 110 patients who had been diagnosed with VCI in accordance with standardized VCI diagnostic clinical criteria (NINDS-AIREN). All subjects underwent a brain-MRI imaging study and a complete blood screening test. All subjects were evaluated by two board certified neurologists with expertise in dementia.^21^

**Clinical Diagnostic Criteria**: NINDS-AIREN

**Acknowledgements**: The study has been partially funded by the Italian Ministry of University and Research (MIUR) to Carlo Masullo.

**COHORT: MON**
**PI:** Prof. Roberto Monastero, MD, PhD; Department of Biomedicine, Neuroscience and Advanced Diagnostics (BIND), University of Palermo, Palermo, Italy; Dementia and Parkinson’s disease Center, University Hospital “P. Giaccone”, Palermo, Italy

**Resource:** Population based cohort study **-** Zabùt Aging Project **(ZAP)**

**Description:** ZAP is a prospective population-based cohort study regarding normal and pathological aging, conducted in an Italian cohort of a rural village in southern Italy, Sambuca di Sicilia. The study included a baseline assessment and a 10-year follow-up examination. All subjects underwent a multidimensional protocol including physical, neurological, functional, cognitive-behavioural examination, laboratory testing, DNA sampling with APOE genotyping and - whenever available - neuroimaging with computed tomography or magnetic resonance. The ZAP study was performed in accordance with the principles outlined in the Helsinki declaration and all participants or their caregiver provided written informed consent. The ZAP study design was approved by the local ethical committee ASP–1 (“Azienda Sanitaria Provinciale”, i.e., provincial health authority) of Agrigento, Italy, and general authorization for the genetic data treatment was provided by the Italian data Protection Authority.

**Clinical diagnostic criteria:** The diagnosis of dementia was made according to the DSM-IV-TR criteria and probable AD was diagnosed according to the criteria established by the National Institute on Aging and the Alzheimer’s Association (McKhann GM et al, Alzheimer’s Dement 2011). Vascular dementia was diagnosed according to the NINDS-AIREN International Workshop Diagnostic Criteria (Román GC et al, Neurology 1993). The diagnosis of Mild Vascular Cognitive Disorder was assessed using the Diagnostic Criteria for Vascular Cognitive Disorder: VASCOG statement (Sachdev P, Alzheimer Dis Assoc Disord 2014). Mild Cognitive Impairment was diagnosed according to Petersen’s criteria (J Intern Med 2004), while Subjective Cognitive Decline was diagnosed according to the criteria of Jessen et al. (Alzheimer’s Dement 2014).

**Publication reference:** Marino Gammazza A, Restivo V, Baschi R, Caruso Bavisotto C, Cefalù AB, Accardi G, Conway de Macario E, Macario AJL, Cappello F, Monastero R. Circulating Molecular Chaperones in Subjects with Amnestic Mild Cognitive Impairment and Alzheimer's Disease: Data from the Zabùt Aging Project. J Alzheimers Dis. 2022;87(1):161-172. doi: 10.3233/JAD-180825.

Oral Health Status in Subjects with Amnestic Mild Cognitive Impairment and Alzheimer's Disease: Data from the Zabút Aging Project. J Alzheimers Dis. 2022;87(1):173-183. doi: 10.3233/JAD-200385.

Spina R, Noto D, Barbagallo CM, Monastero R, Ingrassia V, Valenti V, Baschi R, Pipitone A, Giammanco A, La Spada MP, Misiano G, Scrimali C, Cefalù AB, Averna MR. Genetic epidemiology of autosomal recessive hypercholesterolemia in Sicily: Identification by next-generation sequencing of a new kindred. J Clin Lipidol. 2018 Jan-Feb;12(1):145-151. doi: 10.1016/j.jacl.2017.10.014.

**Acknowledgements:** The ZAP data collection was supported by a Grant project for young researcher 2007 (GR–2007–686973) from the Italian Ministry of Health to Roberto Monastero, who is the Principal Investigator of the ZAP. The authors thank all participants and all persons working in the Zabùt Aging Project for data collection and management.

**COHORT: QUI**
**PI:** Asst. Prof. Shawn Westaway; Layton Aging and Alzheimer's Disease Center, Oregon Health & Science University, USA

**Resource:** Clinical cohort study - National Institute on Aging (NIA)− Layton Oregon Aging and Alzheimer’s Disease Center **(OADC)**.

**Description:** A selection of 133 participants of the OADC longitudinal cohort studies was made for this study as follows: the Oregon Brain Aging Study (OBAS) (n = 48), the Intelligent Systems for Assessment of Aging Changes (ISAAC) Study (n = 11), the Klamath Exceptional Aging Project (KEAP) (n = 24), the African American Dementia and Aging Project (AADAPt) (n = 1), the Oregon Community Brain Donor Program (CBDP) (n = 24), the Oregon Living Laboratory (OLL) (n = 3), and the OADC patient registry(n=22)). All studies were approved by the Oregon Health & Science University’s institutional review board, and all participants provided written informed consent. Participants underwent cognitive test batteries annually. Comprehensive longitudinal datasets including demographic background, known APOE status, (determined via restriction digest, sequencing a PCR product, or by SNP genotyping) and cognitive decline were obtained.

**Clinical diagnostic criteria:** Participants were evaluated by board certified neurologists with expertise in dementia. A clinical diagnosis is defined in a consensus conference of neurologists, neuropsychologists, psychiatrist, and nursing staff, according to established clinical criteria (McKhann 1984; Erkinjuntti 1994; Albert 2011).

**Post-mortem diagnostic criteria:** For those participants who received brain autopsy, the brain tissue was examined according to the National Alzheimer’s Coordinating Centre (NACC) protocol and diagnosis rendered by a neuropathologist with expertise in dementia.

**Publication reference:** https://doi.org/10.1186/s13293-019-0228-8; doi: 10.1212/wnl.54.1.105. https://www.alz.washington.edu/WEB/forms_np.html

**Acknowledgements:** Layton Aging and Alzheimer's Disease Center. This study was funded in part by an Agency for Healthcare Research & Quality-funded BIRCWH K12 award (K12 HD 043488) and by grants from the National Institutes of Health (P30AG024978, R01AG024059, P30AG008017), a Merit Review Grant from the Department of Veteran’s Affairs, an Alzheimer’s Association Clinician Scientist Fellowship, and OADRC: P30AG066518 and P30AG008017. We acknowledge supporting grants and funders: Alzheimer Disease Center Clinical Core (PI: Kaye; eIRB 725; supported by NIH P30 AG008017); Oregon Center for Aging & Technology (ORCATECH; PI: Kaye; eIRB 2765; supported by NIH P30 AG024978); ORCATECH Living Laboratory (OLL or “Living Lab”; PI: Kaye; eIRB 2765; supported by NIH R01 AG024059, P30 AG024978, P30 AG008017, Intel Corporation); Bioengineering Research Partnership, Intelligent Systems for Assessing Aging Changes (BRP or ISAAC; PI: Kaye; eIRB 2353; supported by NIH R01 AG024059, P30 AG024978, P30 AG008017, Intel Corporation; The Oregon Brain Aging Study (OBAS; PI: Kaye; eIRB 361; supported by Department of Veterans Affairs, NIH P30 AG008017, M01 RR000334, UL1 RR024140)

**COHORT: SADEM**

**PI:** Dra. Teresa Juárez-Cedillo; Centro Médico Nacional Siglo XXI, Instituto Mexicano del Seguro Social (IMSS), Ciudad de México, Mexico.

**Resource:** The Study on Aging and Dementia in Mexico **(SADEM)**, National Council of Science and Technology (CONACYT) and Found for the Promotion of Health Research, Instituto Mexicano del Seguro Social (FIS/IMSS)

**Study description:**  The Study on Aging and Dementia in Mexico (SADEM), is a cohort with a random sample represented of individuals over 60 years of age, drawn from the senior citizen registry of IMSS in 24 of the 32 delegations across Mexico City. The research protocol was reviewed and approved by The National Commission of Scientific Research and the IMSS Ethics Commission (registration number 2015-785-012). Subjects are assessed for up to 10 years.

**Clinical diagnostic criteria:** The diagnosis of dementia was done in two stages. Phase 1: Screening The Mini-Mental State Exam (MMSE) was used as a screening tool, and a formal diagnosis was only made in phase 2. Diagnostic confirmation with screening test were evaluated in consultation by a specialist used the Clinical Dementia Rating (CDR) and each diagnosis was based on the criteria for dementia in DSM-5, the subjects were grouped according NINCDS-ADRDA and/or the NINDS-AIREN. For clinical assessment we used: MMSE, phonological fluency, Alzheimer disease assessment scale-cognitive (ADAS-cog) and the Frontal Assessment. The level of abnormalities was evaluated by the magnetic resonance images. The diagnoses fell into three categories: a) Probable Alzheimer’s disease (AD), b) vascular dementia (VaD), and c) mixed dementia (MD) without established criteria for MD.

**Publication reference:** doi: 10.3233/JAD-220012, doi: 10.3233/JAD-200574, doi: 10.1159/000345251, doi: 10.1002/gps.4030, doi: 10.1002/gps.4030, doi: 10.1007/s00277-014-2155-4, doi: 10.1002/gps.4216, doi: 10.1016/j.imlet.2016.07.011, doi: 10.1007/s40618-017-0654-6, doi: 10.1002/mgg3.918, doi: 10.1007/s12035-020-02162-3, doi: 10.1007/s11011-021-00740-5, doi: 10.1007/s12035-020-02162-3, doi.org/10.3389/fnagi.2022.952173.

**Acknowledgements:** This project was supported by the Fund for the Promotion of Health Research, Mexican Institute of Social Security, FIS/IMSS/PROT/G09/772 and by grants from SSA/IMSS/ISSSTE-CONACYT (Mexico) Salud-69842.

**COHORT: SER**
**PI:** Davide Seripa; Department of Hematology and Stem Cell Transplant, Vito Fazzi Hospital, Lecce, Italy.

**Resource:** Geriatric Unit

**Description:** Cohort of elderly attending a geriatric ward

**Clinical diagnostic criteria:** NINCDS-ADRDA; Petersen; NINDS-AIREN

We thank our EADB collaborators (see EADB acknowledgements) who contributed genotype data:

- **EADB-GSA** (please refer to the EADB acknowledgements)
- **Copenhagen City Heart Study (CCHS)** - Ruth Frikke-Schmidt; Department of Clinical Biochemistry, Copenhagen University Hospital – Rigshospitalet and Department of Clinical Medicine, University of Copenhagen, Copenhagen, Denmark. The Copenhagen City Heart Study (CCHS) is a prospective study of the Danish general population initiated in 1976-78 with follow-up examinations in 1981-83, 1991-94, 2001-03, and 2011-13. Individuals were selected randomly based on the national Danish Civil Registration System to reflect the adult Danish population aged 20-100. Data were obtained from a self-administered questionnaire reviewed together with an investigator at the day of attendance, a physical examination, and from blood samples including DNA extraction. Genotypes were available on 8,118 individuals from the 1991-94 examination following genotyping on the Illumina Metabochip. ICD10 code F01 was used for the diagnosis. The Copenhagen City Heart Study (CCHS) was funded by The Danish Heart Foundation and the Velux Foundation.
- **DemGene** - Ole A. Andreassen, NORMENT Centre, University of Oslo, Oslo, Norway. DemGene Network is a Norwegian network of clinical sites collecting cases from Memory Clinics based on standardised examination of cognitive, functional and behavioural measures and data on the progression of most patients. The Norwegian DemGene Network includes 2,224 cases and 1,855 healthy controls. The cases were diagnosed according to the recommendations from the National Institute on Aging–Alzheimer’s Association (NIA/AA), the NINCDS-ADRDA criteria or the ICD-10 research criteria. The controls were screened with a standardised interview and cognitive tests. Individuals from the DemGene Study were genotyped using the Human Omni Express-24 v.1.1 (Illumina Inc., San Diego, CA, USA) at deCODE Genetics (Reykjavik, Iceland). The project has received funding from The Research Council of Norway (RCN) Grant Nos. 213837, 223273, 225989, and 324252 and EU JPND Program RCN Grant Nos. 237250, 311993, the South-East Norway Health Authority Grant No. 2013-123, the Norwegian Health Association, and KG Jebsen Stiftelsen. The RCN FRIPRO Mobility grant scheme (FRICON) is co-funded by the European Union’s Seventh Framework Programme for research, technological development and demonstration under Marie Curie grant agreement No 608695. European Community’s grant PIAPP-GA-2011-286213 PsychDPC.
- **FinnGen** - Mikko Hiltunen, Institute of Biomedicine, University of Eastern Finland, Kuopio, Finland (please refer to EADB acknowledgements). FinnGen is a public-private partnership project that aggregates genotype data from Finnish biobanks ([*https://www.finngen.fi/en*](https://www.finngen.fi/en)). The FinnGen study is approved by Finnish Institute for Health and Welfare (permit numbers: THL/2031/6.02.00/2017, THL/1101/5.05.00/2017, THL/341/6.02.00/2018, THL/2222/6.02.00/2018, THL/283/6.02.00/2019, THL/1721/5.05.00/2019, THL/1524/5.05.00/2020, and THL/2364/14.02/2020), Digital and population data service agency (permit numbers: VRK43431/2017-3, VRK/6909/2018-3, VRK/4415/2019-3), the Social Insurance Institution (permit numbers: KELA 58/522/2017, KELA 131/522/2018, KELA 70/522/2019, KELA 98/522/2019, KELA 138/522/2019, KELA 2/522/2020, KELA 16/522/2020 and Statistics Finland (permit numbers: TK-53-1041-17 and TK-53-90-20).
- **Sydney Memory and Ageing Study (SMAS)** - Prof. Perminder Sachdev; Centre for Healthy Brain Ageing, School of Psychiatry, University of New South Wales Sydney, New South Wales, Australia. Participants aged 70-90 years of age were recruited randomly from the community in Sydney, Australia (N=1037) into a longitudinal study to investigate mild cognitive impairment and related syndromes and to determine the rate of cognitive change. A face-to-face interview was undertaken, and questionnaire data collected, including demographics, cognitive performance, a medical exam and medical history. Most participants provided a blood sample for genetic and biochemistry analyses. Neuroimaging was performed on a subset of participants. The majority of participants had an informant, who was able to answer questions about the participant’s cognitive performance and daily functions. The University of New South Wales and the South Eastern Sydney Illawarra Area Health Service Human Research Ethics Committees gave ethics approval for the study. Written informed consent was provided by all participants. All participants whose neuropsychological or functional profiles indicated the possibility of dementia were given consensus diagnoses. Consensus diagnoses were made by an expert panel of clinicians including old age psychiatrists, neuropsychiatrists, clinical neuropsychologists and clinical psychologists using all available clinical data and MRI where available. Participants were classified as VCI if they had MCI (Winblad et al., 2004, J Int Med, 256, 240-246) and any one of the following a) self-reported stroke; b) self-reported TIA; c) presence of 2 or more lacunae; d) any infarcts; e) self-report atrial fibrillation and/or f) were in the upper quartile of white matter hyperintensity (WMH) burden (>17,140 mm3). The diagnosis of dementia was based on DSM-IV criteria (APA 2000); the presence of multiple cognitive deficits that represent a decline from a previous level of functioning and include memory impairment and at least one other cognitive disturbance. The cognitive deficits are sufficiently severe to cause impairment in functioning. doi: 10.1017/S1041610210001067. We gratefully acknowledge and thank the Sydney MAS participants, their supporters and the Sydney MAS Research Team (current and former staff and students). Funding was awarded from the Australian National Health and Medical Research Council (NHMRC) Program Grants (350833, 568969, 109308).

**Genotyping, quality control and imputation.**

**Genotyping**

Individuals from the EADB dataset were genotyped on the Illumina GSA array in three independent centers (Centre National de Recherche en Génomique Humaine, Evry, France; Life&Brain, Bonn, Germany; Erasmus Medical University, Rotterdam, The Netherlands).

Individuals from the SMAS dataset were genotyped on Illumina GSA array at the Centre National de Recherche en Génomique Humaine (CNRGH, Evry, France).

Individuals from the DemGene study and blood donors were genotyped using either the Human Omni Express-24 v1.1 chip (Illumina Inc., San Diego, CA) or the DeCodeGenetics_V1_20012591_A1 chip at deCODE Genetics (Reykjavik, Iceland).

Individuals from the CCHS dataset were genotyped on the Illumina Metabochip and/or the Illumina HumanExome.

Individuals from the FinnGen were genotyped on a FinnGen ThermoFisher Axiom custom array at the Thermo Fisher genotyping service facility in San Diego.

**Quality control and imputation**

Standard quality control was performed on variants and samples on all datasets individually, as described elsewhere.^22^ The samples from EADB, SMAS and CCHS datasets were then imputed with the Trans-Omics for Precision Medicine (TOPMed) reference panel.^1,23^ For DemGene dataset, we used the provided cleaned and imputed data (imputation was performed with the Haplotype Reference Consortium (HRC) panel).^24^

FinnGen quality control and imputation can be found on the FinnGen website (<https://finngen.gitbook.io/documentation/methods/phewas).> Imputation was performed using SiSu reference panel consisting of Finnish individuals.

**Association testing**

Association tests were conducted separately in each dataset using logistic regression assuming an additive genetic model as implemented in SNPTEST^25^, except in FinnGen. Analyses were performed on the genotype probabilities in SNPTEST (newml method) and were adjusted for principal components and genotyping centers when necessary. Detailed description of the FinnGen analysis pipeline can be found on the FinnGen website (https://finngen.gitbook.io/documentation/methods/phewas): Briefly, genome statistics were analyzed using Scalable and Accurate Implementation of Generalized mixed model (SAIGE), which uses saddle point approximation (SPA) to calibrate unbalanced case-control ratios^26^ and the first ten genetic PCs, sex, age, and genotyping batch were used as covariates

We filtered out duplicated variants and variants with (i) missing effect size, standard error or P value, (ii) absolute value of effect size above 5, (iii) imputation quality less than 0.3. For datasets not imputed with the TOPMed reference panel, we also excluded (i) variants for which conversion of position or alleles from the GRCh37 assembly to the GRCh38 assembly was not possible or problematic, or (ii) variants with very large difference of frequency between the TOPMed reference panel and the reference panels used to perform imputation.

Results were then combined across studies with a fixed-effect meta-analysis using the inverse variance weighted approach as implemented in the METAL software.^27^ We excluded (i) variants with heterogeneity P value below 5x10-8, (ii) variants with a minor allele frequency below 0.01, and (iii) variants with frequency amplitude above 0.4 (defined as the difference between the maximum and minimum frequency across studies). We further excluded variants not analyzed in the EADB-TOPMed dataset.

## **1 – 6. The Framingham Heart Study (FHS)**

The FHS is a three-generation, single-site, community-based, ongoing cohort study that was initiated in 1948. It now comprises three generations of participants including the Original cohort followed since 1948 (n=5,209)^28^, their Offspring and spouses of the offspring (n=5,216) followed since 1971^29^; and children from the largest Offspring families enrolled in 2000 (Gen 3)^30^.

Participants in the Original and Offspring cohorts are used in these analyses, but Gen 3 participants were not included since they are young (mean age 40±9 years in 2000) and none had developed Alzheimer’s disease. The Original cohort enrolled 5,209 men and women who comprised two-thirds of the adult population then residing in Framingham, Massachusetts. Survivors continue to receive biennial examinations. The Offspring cohort comprises 5,124 persons (including 3,514 biological offspring) who have been examined approximately once every 4 years. Almost all the FHS Original and Offspring participants are NHW. FHS participants had DNA extracted and provided consent for genotyping in the 1990s. All available eligible participants were genotyped at Affymetrix (Santa Clara, CA) through an NHLBI funded SNP-Health Association Resource (SHARe) project using the Affymetrix GeneChip® Human Mapping 500K Array Set and 50K Human Gene Focused Panel®. In 272 persons, small amounts of DNA were extracted from stored whole blood and required whole genome amplification prior to genotyping. Cell lines were available for most of the remaining participants. Genotyping was attempted in 5,293 Original and Offspring cohort participants, and 4,425 persons met QC criteria. Failures (call rate<97%, extreme heterozygosity or high Mendelian error rate) were largely restricted to persons with whole-genome amplified DNA and DNA extracted from stored serum samples. In addition, since the persons with whole genome amplified DNA represent a group of survivors who may differ from the others we included whole genome amplified status as a covariate in FHS analyses. For the prevalent analyses, we also excluded 2,268 participants who were less than 65 years old at the time of the DNA draw and 14 persons with dementia other than Alzheimer’s disease; the remaining 2,143 subjects constitute the FHS sample for the prevalent study. A total of 806 well-genotyped persons from the Original cohort (which has been under ongoing surveillance for incident dementia since 1975) were included in the incident Alzheimer’s disease analyses. The FHS component of this study was approved by the Institutional Review Board of the Boston Medical Center.

The Original cohort of the FHS has been evaluated biennially since 1948, was screened for prevalent dementia and Alzheimer’s disease in 1974-76 and has been under surveillance for incident dementia and Alzheimer’s disease since then^31-33^. The Offspring have been examined once every 4 years and have been screened for prevalent dementia with a neuropsychological battery and brain MRI^34,35^. In order to be consistent with the sampling frame for the AGES and CHS samples, we excluded FHS subjects with a baseline age <65 yrs at the time of DNA draw which was in the 1990s. To minimize survival biases, Original cohort and Offspring participants who developed dementia prior to the date of DNA draw were treated as prevalent cases, and subsequent events in the Original cohort occurring prior to December 2006 were included in the incident analyses.

**All-cause and vascular dementia ascertainment.**

At each clinic exam, participants receive questionnaires, physical examinations and laboratory testing; between examinations they remain under surveillance (regardless of whether or not they live in the vicinity) via physician referrals, record linkage and annual telephone health history updates. Methods used for dementia screening and follow-up have been previously described^31,36^. Briefly, surviving cohort members who attended biennial examination cycles 14 and 15 (May 1975-November 1979) were administered a standardized neuropsychological test battery to establish a dementia-free cohort.

Beginning at examination cycle 17 (1982), the MMSE was administered biennially to the cohort. A MMSE score below the education-specific cutoff score, a decline of three or more points on subsequent administrations, a decline of more than five points compared with any previous examination, or a physician or family referral prompted further in-depth testing. The Offspring cohort that was enrolled in 1971 has undergone eight re-examinations, one approximately every four years. Starting at the second Offspring examination, participants were questioned regarding any subjective memory complaints and since the fifth Offspring examination participants have been administered the MMSE at each visit. In addition, concurrent with the seventh and eighth Offspring examinations (between 1999 and 2004 and then again between 2005 and 2009) surviving Original cohort and all eligible and consenting Offspring participants have undergone volumetric brain MRI and neuropsychological testing^34,35^. The neuropsychological test battery included the Reading subtest of the Wide Range Achievement Test (WRAT-3), the Logical Memory and the Paired Associates Learning tests from the Wechsler Memory Scale, the Visual Reproduction and Hooper Visual Organization Tests, Trails A and B, the Similarities subtest from the Wechsler Adult Intelligence test, the 30-iterm version of the Boston Naming Test and at the second assessment only, the Digit Span, Controlled Word Association and Clock Drawing Tests. Offspring participants suspected to have cognitive impairment based on their MMSE scores, participant, family or physician referral, hospital records or performance in the neuro-psychological test battery described above were referred for more detailed neuropsychological and neurological evaluation.

Each participant thus identified underwent baseline neurologic and neuropsychological examinations. Neurologists (trained in geriatric behavioral assessment) supplemented their clinical assessment with a few structured cognitive tests and administered the Clinical Dementia Rating (CDR). Persons were reassessed systematically for the onset of at least mild dementia. A panel consisting of at least 1 neurologist (S.A., P.A.W., or S.S.) and 1 neuropsychologist (R.A.) reviewed all available medical records to arrive at a final determination regarding the presence or absence of dementia, the date of onset of dementia, and the type of dementia. For this determination, we used data from the neurologist’s examination, neuropsychological test performance, Framingham Study records, hospital records, information from primary care physicians, structured family interviews, computed tomography and magnetic resonance imaging records, and autopsy confirmation when available. All individuals identified as having dementia satisfied the DSM-IV criteria, had dementia severity equivalent to a CDR of 1 or greater, and had symptoms of dementia for at least 6 months. All individuals identified as having Alzheimer-related dementia met the NINCDS-ADRDA criteria for definite, probable, or possible Alzheimer’s disease. Vascular Dementia was diagnosed using the ADDTC criteria, but the presence of vascular dementia did not disqualify a participant from obtaining a concomitant diagnosis of Alzheimer’s disease if indicated. The recruitment of Original cohort participants at FHS had occurred long before the DNA collection with the result that the majority of dementia events in the FHS (although ascertained prospectively) were prevalent at the time of DNA collection or these persons had died prior to DNA draw and were thus excluded from analyses of incident disease. Due to the limited number of incident dementia and Alzheimer’s disease events in the Framingham Offspring only the Original cohort were included in our analyses of incident events. For this study, FHS contributed data on 330 Alzheimer’s disease cases and 3,910 healthy controls with Alzheimer’s disease-free status confirmed as of most recent follow-up.

**Genotyping, quality control and imputation and association testing**

In the 1990s and early 2000s, DNA samples were collected in the three FHS generations for genetic research. All individuals provided consent for genotyping. In 2007, the FHS began genotyping for the NHLBI funded Single Nucleotide Polymorphism (SNP)-Health Association Resource (SHARe) project using approximately 550 000 SNPs (Affymetrix 250K Nsp and 250K Sty mapping arrays plus Affymetrix 50K gene-centered supplemental array) in 9,274 participants from the three generations (including over 1,500 families). Individuals who did not pass QC criteria (call rate < 97%, extreme heterozygosity or high Mendelian error rate) were excluded. Imputation was performed on the Michigan Imputation Server using miniMACH3 and the Haplotype Reference Consortium (HRC) reference panel release 1.1 April 2016 17 using SNPs passing the following criteria: call-rate ≥ 97%, Hardy-Weinberg P ≥ 10-6, < 1000 Mendelian errors, and minor allele frequency (MAF) ≥ 1%. Prior to imputation, phasing was performed using the duoHMM algorithm incorporated into SHAPEIT2 to account for parental genotypes.

**Funding**.

FHS is supported by the National Heart, Lung and Blood Institute's Framingham Heart Study (Contracts No. N01-HC-25195, No. HHSN268201500001I and No. 75N92019D00031), and its contract with Affymetrix, Inc. for genotyping services (Contract No. N02-HL-6-4278). A portion of this research utilized the Linux Cluster for Genetic Analysis (LinGA-II) funded by the Robert Dawson Evans Endowment of the Department of Medicine at Boston University School of Medicine and Boston Medical Center. This study was also supported by grants from the National Institute of Aging (R01s AG033040, AG033193, AG054076, AG049607, AG059421, U01 AG058589, AG061872 and U01-AG049505) and the National Institute of Neurological Disorders and Stroke (R01-NS017950, UH2 NS100605). Dr. DeCarli is supported by the Alzheimer’s Disease Center (P30 AG 010129). We thank the study participants, as well as the study team (especially the investigators and staff of the neurology team) for their contributions and dedication to the study. The authors are pleased to acknowledge that the computational work reported on in this paper was performed on the Shared Computing Cluster that is administered by Boston University Research Computing Services. URL: www.bu.edu/tech/support/research/.

## **1 – 7. INGI-Friuli Venezia Giulia (INGI-FVG)**

The INGI-Friuli Venezia Giulia (INGI-FVG) cohort is a collection of samples coming from six small villages (Clauzetto, Erto, Illegio, Resia, San Martino del Carso, and Sauris) located in North-Eastern Italy, in the Friuli Venezia Giulia region. The FVG Genetic Park is part of the INGI project, a collaboration between research institutions in Italy aimed at reconstructing the molecular bases of complex traits and pathologies by investigating genetically isolated Italian populations.^37^ Studies were conducted referring to a common operational protocol. Genotyping and phenotypic data for 1590 samples are available. A written informed consent for participation was obtained from all subjects. The project was approved by the Ethical committee of the IRCCS Burlo-Garofolo.

**All-cause and vascular dementia ascertainment.**

Eight hundred seventy-seven genotyped individuals carried out a neurological visit. Based on ICD-codes we defined cases of prevalent all-cause dementia.

**Genotyping, quality control and imputation.**

All samples have been genotyped with Illumina 370K/700K high-density SNP array (Illumina Inc., San Diego, CA, USA). Genotypes were called with Illumina GenomeStudio. Each batch was processed according to standard quality control procedures with the following criteria for inclusion: sample call-rate ≥ 0.95, gender check, SNP call rate ≥ 0.95, Hardy-Weinberg Equilibrium (HWE) p-value > 1 × 10−6, and minor allele frequency (MAF) ≥ 0.01. Genotype imputation was conducted using IMPUTE2 considering as reference a custom panel generated merging the 1000 Genomes phase 3 and whole-genome sequences of INGI samples.^37-39^

**Association testing**.

The analyses were performed with linear mixed models (LMM) using the GEMMA (Genome-wide Efficient Mixed Model Association) software which uses a generalized mixed model to account for sample relatedness and cryptic population structure.^40^ Sex and age were included in the analysis as covariates.

**Funding**.

The INGI-FVG study is funded by D70-RESRICGIROTTO to GG. The authors would like to thank the people of the Friuli Venezia Giulia Region for the everlasting support.

## **1 – 8. The Genome Research at ACE Alzheimer Center Barcelona (GR@ACE)**

The Genome Research at Ace Alzheimer Center Barcelona (GR@ACE) and Dementia Genetics Spanish Consortium (DEGESCO) is a dementia and population-based cohort study of risk factors for Alzheimer’s disease conducted across different centers in Spain (DEGESCO Consortium). The population included in this study was 12,599 participants with European descent (7,516 ACD and 1,953 VaD)) and all participants provided written informed consent. This research protocol was approved by the ethics and scientific committees (Acta 25/2016, Ethics Committee H., Clinic I Provincial, Barcelona, Spain).

**All-cause and vascular dementia ascertainment.**

Study participants completed neurological, neuropsychological and social evaluations at Ace Alzheimer Center Barcelona. For each individual, a consensus-based diagnosis about the cognitive status was reached at the time of the study recruitment by a multidisciplinary team of professionals that included neurologists, neuropsychologists and social workers^41^. Cognitive assessment consisted of the Spanish version of the Mini-Mental State Examination (MMSE)^42,43^ the memory part of the Spanish version of the 7 Minutes test^44^ the Spanish version of the Neuropsychiatric Inventory Questionnaire (NPI-Q)^45^ the GDS^46^ the Clinical Dementia Rating Score (CDR)^47^ the Blessed Dementia Scale^48^, and a comprehensive neuropsychological battery of Fundació ACE (NBACE)^49^ Alzheimer’s disease dementia and Vascular dementia were defined according to the NIA-AA^50^ and NINDS-AIREN International Workshop Criteria^51^ respectively. Mild cognitive impairment (MCI) was defined using Petersen’s^52^ and the Cardiovascular health and cognition study criteria^53^ The control group included healthy controls and individuals with subjective cognitive decline (SCD). SCD refers to the perception of memory or other cognitive problems without impairment on standardized cognitive tests^54^ All individuals in the control group had a CDR of 0, a preserved performance (score ≥ 27) on the MMSE and a strictly normal performance in the NBACE.

**Genotyping, quality control, imputation, and association testing.**

DNA was extracted from peripheral blood according to standard procedures using the Chemagic system (Perkin Elmer). Samples reaching DNA concentrations (>10 ng/µl) and presenting high integrity were included for genotyping. Cases and controls were randomized across sample plates to avoid batch effects. For genotyping, we used the Axiom 815K Spanish biobank array (Thermo Fisher) at the Spanish National Centre for Genotyping (CeGEN, Santiago de Compostela, Spain).

Details on genotyping and quality-control procedures are provided in previous publications.^55^ Briefly, individuals with low-quality samples, excess of heterozygosity, sex discrepancies, and familial relations between samples (PI-HAT > 0.1875) were excluded from the analysis. A principal component analysis (PCA) was performed and population outliers were removed. Variants with call rate below 95% or deviation from the Hardy–Weinberg equilibrium (p ≤ 1×10^-06^) were also removed from the analysis. To maximize genetic coverage, we performed single-nucleotide polymorphism (SNP) imputation on genome build GRCh38 using the Trans-Omics for Precision Medicine (TOPMed) imputation server.^23,56^ Rare variants (MAF <1%) and low imputation quality variants (R^2^ < 0.30) were excluded.

We conducted an association analysis adjusting for age, sex, and population structure (4PCs) to test the association of each variant with VaD and ACD using plink (v2.00a).

**Funding**.

The Genome Research @ Ace Alzheimer Center Barcelona project (GR@ACE) is supported by Grifols SA, Fundación bancaria ‘La Caixa’, Ace Alzheimer Center Barcelona and CIBERNED. We are indebted to Trinitat Port-Carbó legacy and her family for their support of Ace Alzheimer Center Barcelona research programs. Ace Alzheimer Center Barcelona is one of the participating centers of the Dementia Genetics Spanish Consortium (DEGESCO). A.R. and M.B. receive support from the European Union/EFPIA Innovative Medicines Initiative Joint undertaking ADAPTED and MOPEAD projects (grant numbers 115975 and 115985, respectively). M.B. and A.R. are also supported by national grants PI13/02434, PI16/01861, PI17/01474, PI19/01240 and PI19/01301. Acción Estratégica en Salud is integrated into the Spanish National R + D + I Plan and funded by ISCIII (Instituto de Salud Carlos III)–Subdirección General de Evaluación and the Fondo Europeo de Desarrollo Regional (FEDER–‘Una manera de hacer Europa’). I.dR. is supported by national grant from the Instituto de Salud Carlos III FI20/00215. Some control samples and data from patients included in this study were provided in part by the National DNA Bank Carlos III (www.bancoadn.org, University of Salamanca, Spain) and Hospital Universitario Virgen de Valme (Sevilla, Spain); they were processed following standard operating procedures with the appropriate approval of the Ethical and Scientific Committee.

## **1 – 9. The Salus in Apulia Study (SAS)**

The “Salus in Apulia Study” is an ongoing population-based prospective cohort comprising 2,472 individuals aged ≥ 65 years and residents in Castellana Grotte, a town located near Bari, Puglia, in the Southeast of Italy. It focused on the sequence of lifestyle including diet, frailty, and other age-related impairments and age-related disease outcomes. In detail, Salus is a public health initiative funded by the Apulia Regional Government and carried on at IRCCS “S. De Bellis” that combines data from two previous populations: the baseline data (MICOL3, M3) were recorded from 2003 to 2005 and the follow-up data from 2013 to 2015 (GreatAGE Study - MICOL4, M4). The GreatAGE study has been described elsewhere.^57^ The invitation included also subjects of the MICOL studies that were in the respective age range above 64 years. In the GreatAge-M4 examination, in addition to the assessment of clinical and lifestyle aspects, neuropsychological features and genetic components have been also evaluated. The study adhered to the “Standards for Reporting Diagnostic Accuracy Studies” (STARD) guidelines (<http://www.stard-statement.org/>), the “Strengthening the Reporting of Observational Studies in Epidemiology” (STROBE) guidelines (<https://www.strobe-statement.org/>).

The study was approved in the 2014 by the Institutional Review Board of the National Institute of Gastroenterology “S. De Bellis”.

**All-cause and vascular dementia ascertainment.**

Diagnoses of dementia are mainly made obtaining data from hospital registries on ICD-9 and ICD-10 codes from all inpatient and outpatient contacts from 1987 through 2018 for all genotyped participants in the Salus in Apulia study. A subset of these diagnoses has been validated against the ICD-10 criteria for Alzheimer’s disease by two neurologists. Based on ICD-codes we defined cases of prevalent all-cause dementia, vascular dementia and Alzheimer disease. We defined participants as controls if they had never received any of the diagnoses listed under all-cause dementia until 2021.

**Genotyping, quality control and imputation.**

A total of 1801 individuals were successfully genotyped with the Illumina Infinium Global Screening Array (GSA) v1 at IRCCS S. De Bellis, Castellana Grotte, Bari, Italy. Genotype calling was performed using Illumina GenomeStudio. Pre-imputation QC included removing SNP call rate < 0.97; Hardy-Weinberg p-value < 1x10^-6^; MAF < 0.01, sample call rate < 0.97, gender check. Imputation was performed using the Michigan University Imputation server and reference panel HRC (Build 37). EAGLE v2.4 was using for phasing and MINIMAC v4 for imputation process

**Association testing**.

The analyses were performed with linear mixed models (LMM) using the GEMMA (Genome-wide Efficient Mixed Model Association) software which uses a generalized mixed model to account for sample relatedness and cryptic population structure.^40^ Sex and age were included in the analysis as covariates.

**Funding**.

The “Salus in Apulia” study is funded by the Italian Ministry of Health with the “Ricerca Corrente 2019” Grant and under the Aging Network of Italian Research Hospitals (IRCCS).

The authors thank the MICOL Study group and the Salus in Apulia Research Team, and the General Practitioners of Castellana Grotte for the fundamental role in recruiting participants to this study.

## **1 – 10. The Hong Kong Osteoporosis Study (HKOS)**

The Hong Kong Osteoporosis Study (HKOS) has been established since 1995, and the cohort was described elsewhere.^58^ In brief, 9,449 Southern Chinese community-dwelling participants were recruited from public road shows and health fairs in Hong Kong from 1995 to 2010. Self-reported questionnaires, anthropometric data, clinical measurements, biochemical measurements, and bone mineral density measurements were collected from the study participants at baseline and follow-up visits. The HKOS participants were also followed in silico using the representative electronic medical database in Hong Kong, namely Clinical Data Analysis and Reporting System (CDARS), which is managed by the Hong Kong Hospital Authority.

**All-cause and vascular dementia ascertainment.**

The demographic data and diagnosis records (in the form of ICD-9) of all the genotyped HKOS participants were retrieved from CDARS from 1 January 1995 up to 31 December 2019 (study end date). Prevalent all-cause and vascular dementia cases were defined as study participants who had the ICD-9 diagnosis codes in Table SI-2, up to 31 December 2019. Incident cases were study participants who did not have relevant ICD-9 diagnosis codes of all-cause and vascular dementia prior to baseline visit, but were diagnosed during the follow-up period up to the study end date. Controls were defined as study participants who had never been diagnosed with all-cause and vascular dementia up to the study end date.

**Genotyping, quality control and imputation.**

The study participants were either genotyped using Illumina HumanHap 610 Quad chip or Illumina Global Screening Array. Genetic variants which did not pass the quality control criteria (call rate <95%, minor allele frequency [MAF]<1%, or/and violating Hardy-Weinberg equilibrium (p<1x10^-6^ among the controls) were excluded. Upon pre-phasing by duoHMM of SHAPEIT, the data of each genotyping platform was separately imputed with reference to the Haplotype Reference Consortium (HRC) reference panel by Michigan Imputation Server. For imputed genetic variants, only those with info score ≥0.4 were included in further analysis. Quality control was applied again to the imputed data. Genetic variants not passing the quality control criteria (call rate <95%, MAF<1% or/and Hardy-Weinberg equilibrium (p<1x10^-6^ in controls) were excluded. Samples with >5% genotyping missing rate were removed from further analysis.

**Association testing**.

Since the trait is binary, only individuals distantly related were kept, with a cutoff of estimated genetic relationship >0.05 using GCTA. For prevalent all-cause and vascular dementia, GWAS was performed using logistic regression model implemented by PLINK1.9, with the adjustment for gender and age as covariates. For incident all-cause and vascular dementia, Cox proportional hazards regression analysis was performed with age as x-axis and adjustment for gender using the GenABEL package in R.

**Funding**.

Nil

## **1 – 11. The Nord-Trøndelag Health Study (HUNT)**

The HUNT consists of three different population-based health surveys conducted in the county of Nord-Trøndelag, Norway over approximately 20 years (HUNT1 [1984-1986], HUNT2 [1995-1997] and HUNT3 [2006-2008]).^1^ In each survey, the entire adult population (≥ 20 years) was invited to participate by completing questionnaires, attending clinical examinations and interviews. Participation rates in HUNT1, HUNT2 and HUNT3 were 89.4% (n=77,212), 69.5% (n=65 237) and 54.1% (n=50 807), respectively.^1^ Taken together, the study included more than 120,000 different individuals from Nord-Trøndelag County. Biological samples including DNA have been collected for approximately 80,000 participants. The HUNT Study has been described in more detail elsewhere.^1^ For the present study, we included participants from HUNT2 and HUNT3. The current study is approved by the Regional Committee for Medical and Health Research Ethics (ref. 2017/1031).

**All-cause and vascular dementia ascertainment.**

The health care system in Norway is publicly funded. Levanger hospital and Namsos hospital, which are the two only hospitals in Nord-Trøndelag, have catchment area responsibilities for the whole county. Diagnoses of dementia are mainly made at geriatric, neurological, and old age psychiatric wards and outpatient clinics. We obtained data from hospital registries on ICD-9 and ICD-10 codes from all inpatient and outpatient contacts from 1987 through 2018 for all genotyped participants in the HUNT study. A subset of these diagnoses has been validated against the ICD-10 criteria for Alzheimer’s disease by four specialists in geriatrics and old age psychiatry as part of the Health and Memory Study.2

Based on ICD-codes we defined cases of prevalent all-cause dementia, vascular dementia and Alzheimer disease, by at least one local hospital contact due to the diagnoses given in the table below. Incident cases were defined as those who at participation in HUNT had never received any of the diagnoses listed under all-cause dementia in the table below, but fulfilled criteria for either of the dementia types during follow-up (till 2018). We defined participants as controls if they had never received any of the diagnoses listed under all-cause dementia in the table below during follow-up (until 2018).

**Genotyping, quality control and imputation.**

In total, DNA from 71,860 HUNT samples was genotyped using one of three different Illumina HumanCoreExome arrays (HumanCoreExome12 v1.0, HumanCoreExome12 v1.1 and UM HUNT Biobank v1.0). Samples that failed to reach a 99% call rate, had contamination > 2.5% as estimated with BAF Regress,3 large chromosomal copy number variants, lower call rate of a technical duplicate pair and twins, gonosomal constellations other than XX and XY, or whose inferred sex contradicted the reported gender, were excluded. Samples that passed quality control were analysed in a second round of genotype calling following the Genome Studio quality control protocol described elsewhere.4 Genomic position, strand orientation and the reference allele of genotyped variants were determined by aligning their probe sequences against the human genome (Genome Reference Consortium Human genome build 37 and revised Cambridge Reference Sequence of the human mitochondrial DNA; http://genome.ucsc.edu) using BLAT.5 Variants were excluded if their probe sequences could not be perfectly mapped, cluster separation was < 0.3, Gentrain score < 0.15, showed deviations from Hardy Weinberg equilibrium in unrelated samples of European ancestry with p-value < 0.0001), had a call rate < 99%, or another assay with higher call rate genotyped the same variant. Ancestry of all samples was inferred by projecting all genotyped samples into the space of the principal components of the Human Genome Diversity Project (HGDP) reference panel (938 unrelated individuals; downloaded from http://csg.sph.umich.edu/chaolong/LASER/),6,7 using PLINK. Recent European ancestry was defined as samples that fell into an ellipsoid spanning exclusively European population of the HGDP panel. The different arrays were harmonized by reducing to a set of overlapping variants and excluding variants that showed frequency differences > 15% between data sets, or that were monomorphic in one and had MAF > 1% in another data set. The resulting genotype data were phased using Eagle2 v2.3 47.8

**Imputation**

Imputation was performed on the 69,715 samples of recent European ancestry using Minimac3 (v2.0.1, http://genome.sph.umich.edu/wiki/Minimac3)9 with default settings (2.5 Mb reference based chunking with 500kb windows) and a customized Haplotype Reference consortium release 1.1 (HRC v1.1) for autosomal variants and HRC v1.1 for chromosome X variants.10 The customized reference panel represented the merged panel of two reciprocally imputed reference panels: (1) 2,201 low-coverage whole-genome sequences samples from the HUNT study and (2) HRC v1.1 with 1,023 HUNT WGS samples removed before merging. We excluded imputed variants with Rsq < 0.3 or minor allele count <3.

**Association testing**.

We used the Scalable and Accurate Implementation of GEneralized mixed model (SAIGE),11 which uses a generalized mixed model to account for sample relatedness and cryptic population structure. We ran a mixed logistic regression model, including sex, birth year, genotyping batch, and the first 4 principal components as covariates. The principal components were calculated by projecting all samples into the space of the principal components of unrelated HUNT samples, using directly genotyped variants in PLINK v1.9012.

**Acknowledgements and Funding**.

This work was supported by the South-Eastern Norway Regional Health Authority (grant no. 2020034 to B.S.W.). The Trøndelag Health Study (HUNT) is a collaboration between HUNT Research Centre (Faculty of Medicine and Health Sciences, Norwegian University of Science and Technology NTNU), Trøndelag County Council, Central Norway Regional Health Authority, and the Norwegian Institute of Public Health. The genotyping was financed by the National Institute of health (NIH), University of Michigan, The Norwegian Research council, and Central Norway Regional Health Authority and the Faculty of Medicine and Health Sciences, Norwegian University of Science and Technology (NTNU). The genotype quality control and imputation has been conducted by the K.G. Jebsen center for genetic epidemiology, Department of public health and nursing, Faculty of medicine and health sciences, Norwegian University of Science and Technology (NTNU).

## **1 – 12. The MEMENTO cohort**

Memento is a French multicenter cohort that aims to improve current knowledge of the natural history of Alzheimer's disease and related disorders (ADRD) and identify new patient phenotypes associated with the risk of developing dementia. The Memento cohort includes patients from the 26 participating memory clinics across France between 2011 and 2014. Participants were followed at least annually for a median of 5 years.^59,60^ Individuals were eligible for inclusion if they (1) were 18 years or older; presented with at least one cognitive deficit defined as performing worse than 1 SD to the mean in one or more cognitive domains (considered as MCI), or (2) presented with an isolated cognitive complaint and were 60 years of age or older. They also had to score on the clinical dementia rating (CDR) scale ≤0.5 (i.e., not demented); have sufficient visual and auditory abilities to partake in neuropsychological testing; and have health insurance, as required by the French government (France has universal access to health care for all legal residents, independent of age, professional standing, or revenue).^61^ All participants signed an informed consent form.

**All-cause and vascular dementia ascertainment.**

All incident cases of dementia were reviewed by a panel of expert neurologists/geriatricians, blinded to genetic and biological biomarkers using the Diagnostic and Statistical Manual of Mental Disorders (DSM-IV criteria).

The etiologic diagnosis of dementia was made according to NINCDS-ADRDA for Alzheimer Disease, DLB consortium for dementia with Lewy bodies, Rascovsky criteria for frontotemporal lobar degeneration and NINDS-AIREN for Vascular dementia.

**Genotyping, quality control, imputation, and association testing.**

All samples were genotyped using the Illumina Global Screening Array (GSA). Pre-imputation QC included removing SNPs with MAF<0.01, call-rate<0.98 and HWE<0.001; removing samples with call-rate<0.05, heterozygosity beyond 3SD, failed sex-check using genotype data of X-chromosome, related sample based on IBD (pi_hat>0.1875). PCA analysis was performed using Plink v1.90. PCA outliers were defined beyond 6SD of PC1 and PC2. Imputation was performed using the Michigan Imputation Server panel with HRC.r1.1.2016 (predominantly European Ancestry), and phasing was performed using Eagle. GWAS was conducted using Plink v1.90 using logistic regression adjusting for age, sex, center, and PCs 1-4.

**Funding**.

MEMENTO is sponsored by the Fondation Plan Alzheimer (Alzheimer Plan 2008– 2012). This work was also supported by the following: CIC 1401-EC, Bordeaux University Hospital, Inserm, the University of Bordeaux, and a grant (European Alzheimer & Dementia BioBank, EADB) from the EU Joint Program – Neurodegenerative Disease Research (JPND). S Debette is supported by a grant overseen by the French National Research Agency (ANR) as part of the “Investment for the Future” Programme ANR-18-RHUS-002, the EU JPND, the ERC and the EU H2020 under grant agreements No 643417, 640643, 667375, and 754517. Part of the computations were performed at the Bordeaux Bioinformatics Center (CBiB), University of Bordeaux and at the CREDIM (Centre de Ressource et Développement en Informatique Médicale) at University of Bordeaux, on a server infrastructure supported by the Fondation Claude Pompidou. Inserm UMR1167 is also funded by Inserm, Institut Pasteur de Lille, the Lille Métropole Communauté Urbaine, and the French government’s LABEX DISTALZ program (development of innovative strategies for a transdisciplinary approach to Alzheimer’s disease).

## **1 – 13. The Monongahela-Youghiogheny Healthy Aging Team (MYHAT)**

MYHAT is an age-stratified random sample drawn from the publicly available voter registration list for a group of small towns in southwestern, Pennsylvania, USA. This population-based cohort was recruited between 2006 and 2008 and is being followed annually for the development of mild cognitive impairment (MCI) and dementia. Inclusion criteria at study entry included 1) being 65 years and older, 2) living in one of the designated towns, 3) not residing in a long-term-care facility, 4) having vision and hearing sufficient to permit neuropsychological testing, and 5) not being decisionally impaired. Eligible participants who consented were briefly assessed using the Mini-Mental State Exam (MMSE). ([Folstein](https://www.ncbi.nlm.nih.gov/pmc/articles/PMC7572515/#R14) *[et al.](https://www.ncbi.nlm.nih.gov/pmc/articles/PMC7572515/#R14)*[, 1975](https://www.ncbi.nlm.nih.gov/pmc/articles/PMC7572515/#R14)) Only participants without substantial cognitive impairment at recruitment (age-education adjusted MMSE score ([Mungas](https://www.ncbi.nlm.nih.gov/pmc/articles/PMC7572515/#R33) *[et al.](https://www.ncbi.nlm.nih.gov/pmc/articles/PMC7572515/#R33)*[, 1996](https://www.ncbi.nlm.nih.gov/pmc/articles/PMC7572515/#R33)) ≥21) were invited to complete the full assessment and thus eligible for annual follow-up. The University of Pittsburgh Institutional Review Board approved all study procedures, and all participants provided written informed consent. ([Ganguli *et al.*, 2009](https://www.ncbi.nlm.nih.gov/pmc/articles/PMC7572515/#R20))

**All-cause and vascular dementia ascertainment.**

Dementia was ascertained by means of the Clinical Dementia Rating (CDR ®) Staging Instrument (<https://knightadrc.wustl.edu/professionals-clinicians/cdr-dementia-staging-instrument/>) which is based on individuals’ cognitively-driven everyday functioning. Participants were assessed by interviewers trained and certified in the CDR.^62^ We classified participants with CDR=0 as normal, CDR=0.5 as MCI, and CDR ≥1 as dementia.

**Genotyping, quality control and imputation.**

A total of 907 MYHAT samples were genotyped with two different Illumina Omni chips, including HumanOmni1S-8-v1 chip, and HumanOmni2-5-8-v1 chip. No sample had a genotyping rate of less than 95% in either chip. The genotype probs with missing call rate > 5% or only present on one chip were removed. The rest of the common genotype probs were phased with the Eagle and then imputed on the Michigan Imputation Server with 1000 Genome Phase 1 Version 3 reference panel. In post-imputation QC, SNPs with low quality score (R^2^< 0.3), MAF<0.01 and Hardy-Weinberg equilibrium test p-value <1E-5 were removed. A sliding window approach with a window size of 2000 bp shifted every 200 variants was implemented to estimate correlation (r^2^). The principal component analysis (PCA) was implemented with Plink 2.0 after filtering out SNPs with MAF<0.05 and having highly correlation SNPs (r^2^ > 0.5).

**Association testing**.

The association analysis was also performed on Plink with sex, age, years of education, and the first four PCs.

**Funding**.

MYHAT is supported by NIH grants R37 AG023651, R01 AG030653, R01 AG064877

## **1 – 14. The Religious Orders Study and Memory and Aging Project (ROSMAP)**

ROSMAP are two community-based cohort studies. The ROS has been ongoing since 1993, with a rolling admission. Through July of 2010, 1,139 older nuns, priests, and brothers from across the United States initially free of dementia who agreed to annual clinical evaluation and brain donation at the time of death completed their baseline evaluation. The MAP has been on-going since 1997, also with a rolling admission. Through July of 2010, 1,356 older persons from across northeastern Illinois initially free of dementia who agreed to annual clinical evaluation and organ donation at the time of death completed their baseline evaluation. Details of the clinical and neuropathologic evaluations have been previously reported^63-65^.

**All-cause and vascular dementia ascertainment.**

A clinical diagnosis of cognitive status is rendered at every assessment based on a three-stage process including computer scoring of cognitive tests, clinical judgment by a neuropsychologist, and diagnostic classification by a clinician.

All participants undergo a uniform, structured, clinical evaluation including a battery of 19 cognitive tests. These tests were scored by computer using a decision tree designed to mimic clinical judgment and a rating of severity of impairment was given for 5 cognitive domains. A neuropsychologist, blinded to participant demographics, reviews the impairment ratings and other clinical information and renders a clinical judgment regarding the presence of impairment and dementia. A clinician (neurologist, geriatrician, or geriatric nurse practitioner) then reviews all available data and examines the participant and renders a final diagnostic classification.

Clinical diagnosis of dementia and clinical Alzheimer’s dementia are based on criteria of the joint working group of the National Institute of Neurological and Communicative Disorders and Stroke and the Alzheimer’s Disease and Related Disorders Association (NINCDS/ADRDA). The diagnosis of Alzheimer’s dementia requires evidence of a meaningful decline in cognitive function relative to a previous level of performance with impairment in memory and at least one other area of cognition.

Diagnosis of mild cognitive impairment (MCI) is rendered for persons who are judged to have cognitive impairment by the neuropsychologist but are judged to not meet criteria for dementia by the clinician.

Persons diagnosed with MCI or Alzheimer’s dementia may also be diagnosed with another condition that contributes to their cognitive impairment (CI).

Persons without dementia or mild cognitive impairment (MCI) are categorized as having no cognitive impairment (NCI).

**Genotyping, quality control and imputation.**

Genotyping was done on Affy 6.0 and Illimina Omni Express. Imputation was performed using the Michigan Imputation Server panel with HRC.r1 (predominantly European Ancestry). Pre-imputation QC included removing SNPs with MAF<0.01, call-rate<0.95 and HWE<10^-6^. Post-imputation QC included removing monomorphic variants, variants with info<0.3, variants with MAC<5.

**Association testing**.

GWAS for prevalent all-cause dementia was performed using Plink v1.9, with covariates including baseline age, sex, and PCs 1-4. GWAS for incident all-cause dementia was performed using R, with covariates including age at baseline, sex, education, and PCs 1-4.

**Funding**.

Funding from NIA grant P30AG10161, P30AG72975, R01AG17917, RF1AG15819, R01AG30146, U01AG46152, U01AG61256; Translational Genomics Research Institute.

## **1 – 15. The Rotterdam Study (RS1, RS2, RS3)**

The Rotterdam Study is a population-based cohort study among inhabitants of a district of Rotterdam (Ommoord), the Netherlands, that aims to examine the determinants of disease and health in the elderly with a focus on neurogeriatric, cardiovascular, bone, and eye disease.8 In 1990-1993, 7,983 persons aged ≥ 55 years participated and were re-examined every 3 to 4 years (**RS1**). In 2000-2001, the cohort was expanded by 3,011 persons who were of the same age but had not yet been part of the Rotterdam Study (**RS2**) and recently moved into the area. In 2006-2008 a second expansion (**RS3**) of 3,932 persons aged 45 and over was realized. All participants had DNA extracted at their first visit. Genotyping was attempted in participants with high-quality extracted DNA in 2007-2008. In total, 6,291 samples from the Rotterdam Study I, 2,157 samples from Rotterdam Study II and 3,048 samples from Rotterdam Study III were available with good quality genotyping data. Genotyping was done at the Human Genotyping Facility, Genetic Laboratory Department of Internal Medicine, Erasmus MC, Rotterdam, the Netherlands. All participants had blood collected during their first center visit, which was followed by DNA extraction.

**All-cause and vascular dementia ascertainment.**

Participants were screened for dementia at baseline and subsequent centre visits with the Mini-Mental State Examination and the Geriatric Mental Schedule organic level.^66^Those with a Mini-Mental State Examination score <26 or Geriatric Mental Schedule score >0 underwent further investigation and informant interview, including the Cambridge Examination for Mental Disorders of the Elderly. At each centre visit, all participants also underwent routine cognitive assessment, including a verbal fluency test (animal categories), 15-word learning test, letter-digit substitution task, Stroop test, and Purdue pegboard task. In addition, the entire cohort was continuously under surveillance for dementia through electronic linkage of the study database with medical records from general practitioners and the regional institute for outpatient mental health care. Available information on clinical neuroimaging was used when required for diagnosis of dementia subtype. A consensus panel led by a consultant neurologist established the final diagnosis according to standard criteria for dementia (DSM-III-R) and Alzheimer's disease (NINCDS–ADRDA). Follow-up until 1st January 2016 was virtually complete (96.3% of potential person-years). Within this period, participants were censored at date of dementia diagnosis, death, loss to follow-up, or 1st January 2016, whichever came first.

**Genotyping, quality control, imputation, and association testing.**

Genotyping was done in participants with high-quality extracted DNA in 2007-2008 and was performed at the Human Genotyping Facility, Genetic Laboratory Department of Internal Medicine, Erasmus MC, Rotterdam, The Netherlands. Imputation of SNPs was established using the Michigan Imputation server and the HRC reference panel. More specifically, the SHAPEIT2 software was used (v2.r790) to phase the data and Minimac 3 was employed for imputation to the HRC reference panel (v1.0). QC included deletion of participants with a genotype completion rate (<90%), a low genotype call rate (<95%), sex-mismatches, duplicate pairs (just one participant), uncalled variants in over 5% of the individuals and significant violations of the expected Hardy–Weinberg Equilibrium proportions (P<10−6). The GWAS software used was rvtest. Covariates in the association analyses were age, sex, and PCs (1-5).

**Funding**.

The Rotterdam Study is funded by Erasmus Medical Center and Erasmus University, Rotterdam, Netherlands Organization for the Health Research and Development (ZonMw), the Research Institute for Diseases in the Elderly (RIDE), the Ministry of Education, Culture and Science, the Ministry for Health, Welfare and Sports, the European Commission (DG XII), and the Municipality of Rotterdam. This Study is further supported by NWO (Vici 918.76.619). The authors are grateful to the study participants, the staff from the Rotterdam Study and the participating general practitioners and pharmacists. The generation and management of genome-wide association study genotype data for the Rotterdam Study is supported by the Netherlands Organisation of Scientific Research NWO Investments (nr. 175.010.2005.011, 911- 03-012). This study is funded by the Research Institute for Diseases in the Elderly (014-93-015; RIDE2), the Netherlands Genomics Initiative (NGI)/Netherlands Organisation for Scientific Research (NWO) project nr. 050-060-810. The work of CMvD is supported by the NGI Center of Medical Systems Biology. We thank Pascal Arp, Mila Jhamai, Marijn Verkerk, Lizbeth Herrera and Marjolein Peters for their help in creating the GWAS database, and Karol Estrada and Maksim V. Struchalin for their support in creation and analysis of imputed data. The plasma concentrations of total-tau were assessed through the Janssen Prevention Center in Leiden, the Netherlands, on anonymized plasma samples without knowledge of disease status. Janssen had no role in study design and data collection

## **1 – 16. The San Antonio Longitudinal Study of Aging (SALSA)**

The San Antonio Longitudinal Study of Aging (SALSA) is a community-based study of the disablement process in older Mexican Americans (MAs) and European Americans (EAs). Detailed descriptions of the sampling design and response rates have been published previously.^67,68^ Briefly, participants were randomly sampled from three types of neighborhoods purposively selected based on census indicators to represent distinct levels of SES and assimilation to the broader society among Mexican Americans: (1) low-income, almost exclusively MA neighborhoods, where a highly traditional MA cultural orientation predominated (barrio); (2) middle income, ethnically balanced neighborhoods, where upwardly mobile MA families had gradually moved in and EA families had moved out (transitional); and (3) high income, predominantly EA neighborhoods, where MAs had largely adopted the cultural orientation of the broader society (suburbs). The SALSA baseline examination was carried out from April 1992 to June 1996 and consisted of a comprehensive home-based assessment, conducted in the participant’s home, and a performance-based assessment, conducted at a clinical research center. The study was approved by the Institutional Review Board of the University of Texas Health Science Center at San Antonio, and all subjects gave informed consent

## **1 – 17. HARMONIZATION**

Harmonization study is ongoing memory-clinic study, which recruits participants from National University Hospital, Singapore. Four diagnostic categories at baseline were eligible for inclusion in this study:^69^ No cognitive impairment (NCI): individuals who had no objective cognitive impairment on neuropsychological tests, or functional loss, Cognitive impairment no dementia (CIND) was diagnosed in patients who were impaired in at least one cognitive domain on a neuropsychological test battery without loss of daily functions. Vascular CIND was defined as a history of ischemic stroke within the past 6–24 months and neuroimaging evidence of cerebral infarction, with objective evidence of neuropsychological deficits.^70^ Dementia was diagnosed according to Diagnostic and Statistical Manual of Mental Disorders-Fourth Edition (DSM-IV) criteria.

**All-cause and vascular dementia ascertainment.**

The etiological diagnoses of dementia were based on internationally accepted criteria: Alzheimer’s Disease (AD) was diagnosed using the National Institute of Neurological and Communicative Disorders and Stroke and the Alzheimer's Disease and Related Disorders Association (NINCDS-ADRDA)^50^; Vascular dementia (VaD) was defined using the National Institute of Neurological Disorders and Stroke and Association Internationale pour la Recherché et l' Enseignement en Neurosciences (NINDS-AIREN) criteria.^51^

**Funding**.

Harmonization study is funded by the Singapore National Medical Research Council (grants NMRC/CG/NUHS/2010, NMRC/CG/013/2013 and NMRC/CIRG/1485/2018), National Medical Research Council Singapore, Transition Award (A-0006310-00-00).

## **1 – 18. Three-City (3C)**

The 3C is a cohort study conducted in three French cities (Bordeaux, Dijon, and Montpellier), comprising 9,294 participants, designed to estimate the risk of dementia and cognitive impairment attributable to vascular factors. Eligibility criteria included living in the city and being registered on the electoral rolls in 1999, 65 years or older, and not institutionalized. The study protocol was approved by the Ethical Committee of the University Hospital of Kremlin-Bicêtre and each participant signed an informed consent.

**All-cause and vascular dementia ascertainment.**

Diagnosis of dementia was based on a classical three-step procedure. At baseline and each follow-up examination), trained psychologists administered a battery of neuropsychological tests.^1^ Second, a neurologist examined all the participants in Bordeaux and Montpellier. In Dijon, due to the large number of participants, only those who screened positive for dementia using the mini-mental state examination (MMSE) and the Isaacs’ Set Test (IST), with education-level dependent cutoff points,^71^ underwent further clinical examination. The IST is a measure of verbal fluency, and response rapidity, which consists of generating words belonging to given semantic categories (e.g. animal names) in 15 seconds.^72^ This test has been reported to show the earliest decline in the decade preceding dementia diagnosis.^73,74^ Cut-off scores were defined according to education level as previously described; For participants suspected of having dementia, further data on cognitive functioning and daily activities, severity of cognitive disorders (Clinical Dementia Rating Scale), functional assessment which included assessment of disabilities using the Katz (activities of daily living),^75^ Lawton (instrumental activities of daily living)^76^ and Rosow and Breslau scales,^72,77^ and where possible, hospitalization records, CT scans (which was most often used at the beginning of the follow-up period),and magnetic resonance images, were collected using a standardized protocol. Then the study neurologist or geriatrician established a provisional diagnosis. At follow-up, all participants with suspected incident dementia (on the basis on their neuropsychological performances or decline relative to a previous examination) were examined by a neurologist in the three study centers. Third, an independent committee of neurologists and geriatricians reviewed all potential prevalent and incident cases of dementia to reach consensus on the diagnosis and etiology, in accordance with the DSM-IV criteria.^72,78^ The final diagnosis of dementia was made based on all available information.

Dementia subtyping was based on the criteria of the National Institute of Neurological and Communicative Disorders and Stroke–Alzheimer’s Disease and Related Disorders Association (NINCDS-ADRDA) for AD, and on the criteria of the National Institute of Neurological Disorders and Stroke-Association Internationale pour la Recherche et l'Enseignement en Neurosciences (NINDS-AIREN) for vascular dementia.^9,10^In our study, probable and possible VaD cases were combined with mixed dementia (AD with vascular contributions) to define the “all VaD” phenotype, whereas ‘pure’ VaD only considered the probable cases.

**Genotyping, quality control and imputation.**

Genotyping was conducted at the Centre National de Genotypage (www.cng.fr), Evry, France, using the on Illumina Human610-Quad BeadChips. Genotyping was performed on 4,263 participants, of which 186 were excluded for the following reasons: non-Caucasian ethnicity (N=20), first-degree relatives (N=128), call rate < 0.95, gender inconsistencies and population stratification outliers (with principal component values using EIGENSOFT® > 6 standard deviations from the mean of the corresponding component, N=38). After applying quality control measures (call rates of <98%, MAF <1%, Hardy-Weinberg equilibrium p <10^-6^) 537,029 autosomal genotyped SNPs were available for imputation. Imputation to the HRC r1.1 2016 panel was performed on the Michigan imputation server.

**Association testing**.

The GWAS was performed under the additive model of genetic inheritance using the Plink v1.90 software. The association tests were performed using logistic regression adjusting for covariates age, sex, city, and PCs 1-4.

**Funding**.

3C Study is conducted under a partnership agreement among the Institut National de la Santé et de la Recherche Médicale (INSERM), the University of Bordeaux, and Sanofi-Aventis. The Fondation pour la Recherche Médicale funded the preparation and initiation of the study. The 3C Study is also supported by the Caisse Nationale Maladie des Travailleurs Salariés, Direction Générale de la Santé, Mutuelle Générale de l’Education Nationale (MGEN), Institut de la Longévité, Conseils Régionaux of Aquitaine and Bourgogne, Fondation de France, and Ministry of Research–INSERM Programme “Cohortes et collections de données biologiques.” Christophe Tzourio and Stéphanie Debette have received investigator-initiated research funding from the French National Research Agency (ANR) and from the Fondation Leducq. We thank Dr. Anne Boland (CNG) for her technical help in preparing the DNA samples for analyses. This work was supported by the National Foundation for Alzheimer’s disease and related disorders, the Institut Pasteur de Lille, the labex DISTALZ and the Centre National de Génotypage. S.D. is supported by a grant overseen by the French National Research Agency (ANR) as part of the “Investment for the Future Programme” ANR-18-RHUS-0002, by European Union’s Horizon 2020 research and innovation programme under grant agreement No 640643 and 754517.

## **1 – 19. The UK Biobank (UKBB)**

UK Biobank is a large cohort study of more than 500,000 people recruited from across England, Scotland, and Wales. Strengths of UK Biobank include its size, detailed baseline assessment and measurements including biological samples, follow-up assessments for certain issues, and the availability of long-term linkage to outcome data. This has allowed investigators to explore various aspects of multimorbidity including demographic patterns [[4](https://www.ncbi.nlm.nih.gov/pmc/articles/PMC8901063/#pmed.1003931.ref004)], prevalence of disease clusters [[8](https://www.ncbi.nlm.nih.gov/pmc/articles/PMC8901063/#pmed.1003931.ref008)], association with related states such as frailty or sarcopenia [[9](https://www.ncbi.nlm.nih.gov/pmc/articles/PMC8901063/#pmed.1003931.ref009),[10](https://www.ncbi.nlm.nih.gov/pmc/articles/PMC8901063/#pmed.1003931.ref010)], the impact of lifestyle factors in the context of multimorbidity [[11](https://www.ncbi.nlm.nih.gov/pmc/articles/PMC8901063/#pmed.1003931.ref011)], and associations between multimorbidity and adverse health outcomes [[4](https://www.ncbi.nlm.nih.gov/pmc/articles/PMC8901063/#pmed.1003931.ref004),[12](https://www.ncbi.nlm.nih.gov/pmc/articles/PMC8901063/#pmed.1003931.ref012)–[15](https://www.ncbi.nlm.nih.gov/pmc/articles/PMC8901063/#pmed.1003931.ref015)]. This study has been conducted using the UK Biobank Resource under Application Number 58341

**All-cause and vascular dementia ascertainment.**

Although VaD in UKB was defined based on ICD-10 codes (see Table below), we used the family history of dementia GWAS (“imputed dementia”) recently published by Marioni et al for ACD.^79^ Imputed dementia was defined as individuals >= 65 years reporting a history of dementia in one or both parents. As explained in Ghosh et al. ^80^ the effect sizes and standard errors of the imputed dementia GWAS were doubled 2 to analytically correct for the use of proxy phenotypes.

**Genotyping, quality control and imputation.**

Samples were genotyped at the Affymetrix Research Services Laboratory in Santa Clara,
California, USA. Upon receipt of a 96-well plate containing 94 UK Biobank samples,
Affymetrix added two control individuals (from 1000 Genomes) to the same well
positions on each plate: HG00097 to well A12 and HG00264 to well E12. See Affymetrix
laboratory process documentation for further details.

Axiom Array plates were processed on the Affymetrix GeneTitan® Multi-Channel (MC)
Instrument. Genotypes were then called from the resulting intensities in batches of
~4,700 samples (~4,800 including the controls) using the Affymetrix Power Tools
software and the Affymetrix Best Practices Workflow. Supplementary Table S1 shows
the number of samples and plates per batch in the interim release (which includes the
11 UK BiLEVE batches and 22 UK Biobank batches, i.e. 11 batches genotyped on the UK
BiLEVE Axiom array and 22 batches genotyped on the UK Biobank Axiom array).

**Association testing**.

The UKB association analyses were performed with linear mixed models (LMM) using the BOLT-LMM [ref] software. BOLT-LMM has the advantage over other methods in that it accounts for cryptic relatedness and population structure and thus, allows the inclusion of related individuals in the models which increase the overall sample size

## **1 – 20. Accessed GWAS summary statistics**

We used the following data representing GWAS summary statistics of Alzheimer’s disease performed within the ADGC consortium. These data were selected because the participants included did not overlap with other cohorts.

| **Traits** | **Article** | **PMID** | **GWAS access** |
| --- | --- | --- | --- |
| ACD – European – ADGC | Naj et al. 2011 | PMID: [21460841](https://pubmed.ncbi.nlm.nih.gov/21460841) | NIAGADS application |
| ACD – European - UKBB | Marionni et al. 2018 | PMID: [29777097](https://pubmed.ncbi.nlm.nih.gov/29777097) | Nature Supplementary Information |
| ACD – African | Reitz et al 2013 | PMID: [23571587](https://pubmed.ncbi.nlm.nih.gov/23571587) | NIAGADS application |

**Table SI-1: Accessed GWAS summary statistics used in this project**

# **2 - ICD9-10 codes used by cohorts in this project**

| *ICD version* | *ICD code* | *ICD description* |
| --- | --- | --- |
| **All-cause dementia** | | |
| ICD-9 | 290 | Dementia |
| ICD-9 | 294.1 | Dementia with conditions classified elsewhere |
| ICD-9 | 294.9 | Unspecified dementia with conditions classified elsewhere |
| ICD-9 | 331.0 | Alzheimer disease |
| ICD-9 | 331.1 | Pick's disease |
| ICD-9 | 331.2 | Senile degeneration of brain |
| ICD-9 | 331.7 | Cerebral degeneration in diseases classified elsewhere |
| ICD-9 | 331.8 | Other cerebral degeneration |
| ICD-9 | 331.9 | Cerebral degeneration unspecified |
| ICD-10 | F00 | Dementia in Alzheimer disease |
| ICD-10 | F01 | Vascular dementia |
| ICD-10 | F02 | Dementia with diseases classified elsewhere |
| ICD-10 | F03 | Unspecified dementia |
| ICD-10 | F05.1 | Delirium in relation to dementia |
| ICD-10 | F06.7 | Mild cognitive disorder |
| ICD-10 | G30 | Alzheimer disease |
| ICD-10 | G31.0 | Localized brain atrophy |
| ICD-10 | G31.1 | Senile degeneration of brain, not elsewhere classified |
| **Alzheimer disease (AD)** | | |
| ICD-9 | 331.0 | Alzheimer disease |
| ICD-10 | G30 | Alzheimer disease |
| ICD-10 | F00 | Dementia in Alzheimer disease |
| **Vascular dementia** | | |
| ICD-9 | 290.4 | Vascular dementia |
| ICD-10 | F01 | Vascular dementia |

**Table SI-2: ICD9 and ICD10 codes used in this study**

# 3 - Software availability

Gene expression weights for TWAS: <http://gusevlab.org/projects/fusion/>

HESS/ρ-HESS: <https://huwenboshi.github.io/hess/local_hsqg/>

LDSR: <https://github.com/bulik/ldsc>

GWAS-PW: <https://github.com/joepickrell/gwas-pw>

Radial-MR: <https://github.com/WSpiller/RadialMR>

GREP: <https://github.com/saorisakaue/GREP>

EPIGWAS: <https://immunogenomics.hms.harvard.edu/code>

Magma.Celltyping: <https://github.com/NathanSkene/MAGMA_Celltyping>

MR-MEGA: <https://www.geenivaramu.ee/en/tools/mr-mega>;

# 4 – Additional Information for EADB

## 4 – 1. Supplementary list of authors EADB

Céline Bellenguez^1^, Fahri Küçükali^2,3,4^, Iris Jansen^5,6^, Victor Andrade^7,8^, Sonia Moreno-Grau^9,10^, Najaf Amin^11,12^, Benjamin Grenier-Boley^1^, Rafael Campos-Martin^7^, Peter A. Holmans^13^, Anne Boland^14^, Luca Kleineidam^7,8,15^, Vincent Damotte^1^, Sven J. van der Lee^5,16^, Teemu Kuulasmaa^17^, Itziar de Rojas^9,10^, Amber Yaqub^11^, Ivana Prokic^11^, Marcos R, Costa^1,18^, Julien Chapuis^1^, Shahzad Ahmad^11,19^, Vilmantas Giedraitis^20^, Dag Aarsland^21,22^, Pablo Garcia-Gonzalez^9,10^, Carla Abdelnour^9,10^, Emilio Alarcón-Martín^9,23^, Daniel Alcolea^10,24^, Montserrat Alegret^9,10^, Ignacio Alvarez^25,26^, Victoria Álvarez^27,28^, Nicola J. Armstrong^29^, Tsolaki Anthoula^30,31^, Ildebrando Appollonio^32,33^, Marina Arcaro^34^, Silvana Archetti^35^, Alfonso Arias Pastor^36,37^, Beatrice Arosio^38,39^, Lavinia Athanasiu^40^, Henri Bailly^41^, Nerisa Banaj^42^, Miquel Baquero^43^, Ana Belén Pastor^44^, Luisa Benussi^45^, Claudine Berr^46^, Céline Besse^14^, Valentina Bessi^47,48^, Giuliano Binetti^45,49^, Alessandra Bizarro^50^, Rafael Blesa^10,24^, Mercè Boada^9,10^, Barbara Borroni^51^, Silvia Boschi^52^, Paola Bossù^53^, Geir Bråthen^54,55^, Catherine Bresner^13^, Henry Brodaty^29,56^, Keeley J. Brookes^57^, Luis Ignacio Brusco^58,59,60^, Dolores Buiza-Rueda^10,61^, Katharina Bûrger^62,63^, Vanessa Burholt^64,65^, Miguel Calero^10,44,66^, Geneviève Chene^67,68^, Ángel Carracedo^69,70^, Roberta Cecchetti^71^, Laura Cervera-Carles^10,24^, Camille Charbonnier^72^, Caterina Chillotti^73^, Simona Ciccone^39^, Jurgen A.H.R. Claassen^74^, Jordi Clarimon^10,24^, Christopher Clark^75^, Elisa Conti^32^, Anaïs Corma-Gómez^76^, Emanuele Costantini^77^, Carlo Custodero^78^, Delphine Daian^14^, Maria Carolina Dalmasso^7^, Antonio Daniele^77^, Efthimios Dardiotis^79^, Jean-François Dartigues^80^, Peter Paul de Deyn^81^, Stéphanie Debette^80,82^, Jürgen Deckert^83^, Teodoro del Ser^44^, Nicola Denning^84^, Martin Dichgans^62,63,85^, Janine Diehl-Schmid^86^, Mónica Diez-Fairen^25,26^, Paolo Dionigi Rossi^39^, Srdjan Djurovic^40^, Emmanuelle Duron^41^, Emrah Düzel^87,88^, Carole Dufouil^67,68^, Valentina Escott-Price^13,84^, Ana Espinosa^9,10^, Michael Ewers^62,63^, Marta Fernández-Fuertes^76^, Catarina B Ferreira^89^, Evelyn Ferri^39^, Bertrand Fin^14^, Peter Fischer^90^, Tormod Fladby^91^, Klaus Fließbach^8,15^, Juan Fortea^10,24^, Silvia Fostinelli^45^, Nick C. Fox^92^, Emlio Franco-Macías^93^, María J. Bullido^10,94,95^, Ana Frank-García^10,94,96^, Lutz Froelich^97^, Daniela Galimberti^34,88^, Jose Maria García-Alberca^10,98^, Pablo García-González^9^, Sebastian Garcia-Madrona^99^, Guillermo Garcia-Ribas^99^, Roberta Ghidoni^45^, Ina Giegling^100^, Giaccone Giorgio^85^, Oliver Goldhardt^86^, Antonio González-Pérez^101^, Caroline Graff^102,118^, Giulia Grande^103^, Emma Green^104^, Timo Grimmer^86^, Edna Grünblatt^105,106,107^, Tamar Guetta-Baranes^108^, Annakaisa Haapasalo^109^, Georgios Hadjigeorgiou^110^, Harald Hampel^111,112^, Olivier Hanon^41^, John Hardy^113^, Annette M. Hartmann^100^, Lucrezia Hausner^97^, Janet Harwood^13^, Stefanie Heilmann-Heimbach^114^, Seppo Helisalmi^115,116^, Michael T. Heneka^8,16^, Isabel Hernández^9,10^, Martin J. Herrmann^83^, Per Hoffmann^114^, Clive Holmes^117^, Henne Holstege^5,16^, Raquel Huerto Vilas^36,37^, Marc Hulsman^5,16^, Charlotte Johansson^102,118^, Lena Kilander^20^, Anne Kinhult Ståhlbom^102,118^, Miia Kivipelto^119,120,121,122^, Anne Koivisto^115^, Johannes Kornhuber^123^, Mary H. Kosmidis^124^, Carmen Lage^10,125^, Erika J Laukka^103,126^, Alessandra Lauria^50^, Jenni Lehtisalo^115,127^, Ondrej Lerch^128,129^, Alberto Lleó^10,24^, Adolfo Lopez de Munain^10,130^, Malin Löwemark^20^, Lauren Luckcuck^13^, Juan Macías^76^, Catherine A. MacLeod^131^, Wolfgang Maier^8,15^, Francesca Mangialasche^119^, Spallazzi Marco^132^, Marta Marquié^9,10^, Rachel Marshall^13^, Angel Martín Montes^10,94,96^, Carmen Martínez Rodríguez^28^, Carlo Masullo^133^, Simon Mead^134^, Patrizia Mecocci^71^, Miguel Medina^10,44^, Alun Meggy^84^, Shima Mehrabian^135^, Silvia Mendoza^98^, Manuel Menéndez-González^28^, Pablo Mir^10,61^, Susanne Moebus^136^, Merel Mol^137^, Laura Molina-Porcel^138,139^, Laura Montrreal^9^, Laura Morelli^140^, Fermin Moreno^10,130^, Kevin Morgan^141^, Markus M Möthen^114^, Carolina Muchnik^58^, Benedetta Nacmias^47,142^, Tiia Ngandu^127^, Gael Nicolas^72^, Børge G. Nordestgaard^143,144^, Robert Olaso^14^, Adelina Orellana^9,10^, Michela Orsini^77^, Gemma Ortega^9,10^, Alessandro Padovani^51^, Caffarra Paolo^145^, Goran Papenberg^103^, Lucilla Parnetti^87^, Pau Pastor^25,26^, Alba Pérez-Cordón^9^, Jordi Pérez-Tur^10,146,147^, Pierre Pericard^148^, Oliver Peters^149,150^, Yolande A.L. Pijnenburg^5^, Juan A Pineda^76^, Gerard Piñol-Ripoll^36,37^, Claudia Pisanu^151^, Thomas Polak^83^, Julius Popp^152,153,154^, Danielle Posthuma^6^, Josef Priller^150,155^, Raquel Puerta^9^, Olivier Quenez^72^, Inés Quintela^69^, Jesper Qvist Thomassen^156^, Alberto Rábano^10,44^, Innocenzo Rainero^52^, Inez Ramakers^157^, Luis M Real^76,158^, Marcel J.T. Reinders^159^, Steffi Riedel-Heller^160^, Peter Riederer^161^, Natalia Roberto^9^, Eloy Rodriguez-Rodriguez^10,125^, Arvid Rongve^162,163^, Irene Rosas Allende^27,28^, Maitée Rosende-Roca^9,10^, Jose Luis Royo^164^, Elisa Rubino^165^, Dan Rujescu^100^, María Eugenia Sáez^101^, Paraskevi Sakka^166^, Ingvild Saltvedt^55,167^, Ángela Sanabria^9,10^, María Bernal Sánchez-Arjona^93^, Florentino Sanchez-Garcia^168^, Pascual Sánchez Juan^10,125^, Raquel Sánchez-Valle^169^, Sigrid B Sando^54,55^, Michela Scamosci^71^, Nikolaos Scarmeas^170,171^, Elio Scarpini^34,88^, Philip Scheltens^5^, Norbert Scherbaum^172^, Martin Scherer^173^, Matthias Schmid^16,174^, Anja Schneider^8,16^, Jonathan M. Schott^92^, Geir Selbæk^91,175^, Davide Seripa^176^, Alexey A Shadrin^40^, Olivia Skrobot^119^, Hilkka Soininen^115^, Vincenzo Solfrizzi^78^, Alina Solomon^115^, Sandro Sorbi^47,142^, Oscar Sotolongo-Grau^9^, Gianfranco Spalletta^42^, Annika Spottke^16^, Alessio Squassina^177^, Eystein Stordal^178^, Juan Pablo Tartan^9^, Lluís Tárraga^9,10^, Niccolo Tesí^5,16^, Anbupalam Thalamuthu^29^, Tegos Thomas^30,31^, Latchezar Traykov^135^, Lucio Tremolizzo^32,33^, Anne Tybjærg-Hansen^144,156^, Andre Uitterlinden^179^, Abbe Ullgren^102^, Ingun Ulstein^175^, Sergi Valero^9,10^, Aad van der Lugt^180^, Jasper Van Dongen^2,3,4^, Jeroen van Rooij^137^, John van Swieten^137^, Rik Vandenberghe^181,182^, Frans Verhey^157^, Jean-Sébastien Vidal^41^, Jonathan Vogelgsang^183,184^, Martin Vyhnalek^128,129^, Michael Wagner^8,16^, David Wallon^185^, Leonie Weinhold^174^, Jens Wiltfang^183,186,187^, Gill Windle^131^, Bob Woods^131^, Mary Yannakoulia^188^, Miren Zulaica^10,189^, Jan Laczo^128,129^, Vaclav Matoska^190^, Maria Serpente^88^, Francesca Assogna^42^, Fabrizio Piras^42^, Federica Piras^42^, Valentina Ciullo^42^, Jacob Shofany^42^, Carlo Ferrarese^32,33^, Simona Andreoni^32^, Gessica Sala^32^, Chiara Paola Zoia^32^, Maria Del Zompo^177^, Alberto Benussi^51^, Patrizia Bastiani^191^, Mari Takalo*^192^, Teemu Natunen*^192^, Tiina Laatikainen^120,127^, Jaakko Tuomilehto^120,127^, Riitta Antikainen^193,194^, Timo Strandberg^193,195^, Jaana Lindström^127^, Markku Peltonen^127^, Richard Abraham^196^, Ammar Al-Chalabi^197^, Nicholas J. Bass^198^, Carol Brayne^199^, Kristelle S. Brown^200^, John Collinge^201^, David Craig^202^, Pangiotis Deloukas^203^, Nick Fox^204^, Amy Gerrish^204^, Michael Gill^205^, Rhian Gwilliam^203^, John Hardy^206^, Denise Harold^207^, Paul Hollingworth^196^, Jarret A, Johnston^208^, Lesley Jones^196^, Brian Lawlor^205^, Gill Livingston^198^, Simon Lovestone^209^, Michelle Lupton^210,211^, Aoibhinn Lynch^205^, David Mann^212^, Bernadette McGuinness^208^, Andrew McQuillin^198^, Michael C. O’Donovan^196^, Michael J. Owen^196^, Peter Passmore^208^, John F, Powell^210,211^, Petra Proitsi^210,211^, Martin Rossor^204^, Christopher E. Shaw^197^, A. David Smith^213^, Hugh Gurling^214^, Stephen Todd^215^, Catherine Mummery^216^, Nathalie Ryan^216^, Giordano Lacidogna^77^, Ad Adarmes-Gómez^10,61^, Ana Mauleón^9^, Ana Pancho^9^, Anna Gailhajenet^9^, Asunción Lafuente^9^, D Macias-García^10,61^, Elvira Martín^9^, Esther Pelejà^9^, F Carrillo^10,61^, Isabel Sastre Merlín^10,95^, L Garrote-Espina^10,61^, Liliana Vargas^9^, M Carrion-Claro^10,61^, M Marín^93^, Ma Labrador^10,61^, Mar Buendia^9^, María Dolores Alonso^217^, Marina Guitart^9^, Mariona Moreno^9^, Marta Ibarria^9^, Mt Periñán^10,61^, Nuria Aguilera^9^, P Gómez-Garre^10,61^, Pilar Cañabate^9^, R Escuela^10,61^, R Pineda-Sánchez^10,61^, R Vigo-Ortega^10,61^, S Jesús^10,61^, Silvia Preckler^9^, Silvia Rodrigo-Herrero^93^, Susana Diego^9^, Alessandro Vacca^52^, Fausto Roveta^52^, Nicola Salvadori^87^, Elena Chipi^87^, Henning Boecker^15,218^, Christoph Laske^219,220^, Robert Perneczky^65,221^, Costas Anastasiou^188^, Daniel Janowitz^62^, Rainer Malik^62^, Anna Anastasiou^30^, Kayenat Parveen^7^, Carmen Lage^222^, Sara López-García^222^, Anna Antonell^169^, Kalina Yonkova Mihova^223^, Diyana Belezhanska^135^, Heike Weber^224^, Silvia Kochen^225^, Patricia Solis^225^, Nancy Medel^225^, Julieta Lisso^225^, Zulma Sevillano^225^, Daniel G Politis^225,226^, Valeria Cores^225,226^, Carolina Cuesta^225,226^, Cecilia Ortiz^227^, Juan Ignacio Bacha^227^, Mario Rios^228^, Aldo Saenz^228^, Mariana Sanchez Abalos^229^, Eduardo Kohler^230^, Dana Lis Palacio^231^, Ignacio Etchepareborda^231^, Matias Kohler^231^, Gisela Novack^232^, Federico Ariel Prestia^232^, Pablo Galeano^232^, Eduardo M. Castaño^232^, Sandra Germani^233^, Carlos Reyes Toso^233^, Matias Rojo^233^, Carlos Ingino^233^, Carlos Mangone^233^, Sebastiaan Engelborghs^234,235,236,237^, Tagliavini Fabrizio^238^, Sune Fallgaard Nielsen^239^, Lucia Farotti^240^, Chiara Fenoglio^241^, Geert Jan Biessels^242^, Seth Love^243^, Patrick G. Kehoe^243^, Florence Pasquier^244^, Christine Van Broeckhoven^2,3,245^, David C. Rubinsztein^246^, Stefan Teipel^247^, Nathalie Fievet^1^, Vincent Deramecourt^244^, Charlotte Forsell^102,118^, Håkan Thonberg^102,118^, Maria Bjerke^69^, Ellen De Roeck^69^, María Teresa Martínez-Larrad^2248^, Natividad Olivar^233^, Mohsen Ghanbari^11^, Perminder Sachdev^29^, Karen Mather^29^, Frank Jessen^8,16^, M. Arfan Ikram^11^, Alexandre de Mendonça^89^, Jakub Hort^128,129^, Tsolaki Magda^30,31^, Philippe Amouyel^1^, Julie Williams^13^, Ruth Frikke-Schmidt^144,156^, Jordi Clarimon^10,24^, Jean-François Deleuze^14^, Giacomina Rossi^85^, Ole A. Andreassen^40^, Martin Ingelsson^20^, Mikko Hiltunen^17^, Kristel Sleegers^2,3,4^, Cornelia M. van Duijn^11,12^, Rebecca Sims^13^, Wiesje M. van der Flier^5^, Agustín Ruiz^9,10^, Alfredo Ramirez^7,8,16,249^, Jean-Charles Lambert^1^

1. Univ. Lille, Inserm, CHU Lille, Institut Pasteur de Lille, U1167-RID-AGE facteurs de risque et déterminants moléculaires des maladies liées au vieillissement, Lille, France
2. Complex Genetics of Alzheimer's Disease Group, VIB Center for Molecular Neurology, VIB, Antwerp, Belgium
3. Laboratory of Neurogenetics, Institute Born - Bunge, Antwerp, Belgium
4. Department of Biomedical Sciences, University of Antwerp, Neurodegenerative Brain Diseases Group,
5. Alzheimer Center Amsterdam, Department of Neurology, Amsterdam Neuroscience, Vrije Universiteit Amsterdam, Amsterdam UMC, Amsterdam, The Netherlands
6. Department of Complex Trait Genetics, Center for Neurogenomics and Cognitive Research, Amsterdam, The Netherlands
7. Division of Neurogenetics and Molecular Psychiatry, Department of Psychiatry and Psychotherapy, University of Cologne, Medical Faculty, Cologne, Germany.
8. Department of Neurodegenerative Diseases and Geriatric Psychiatry, University Hospital Bonn, Bonn, Germany
9. Research Center and Memory clinic Fundació ACE, Institut Català de Neurociències Aplicades, Universitat Internacional de Catalunya, Barcelona, Spain
10. CIBERNED, Network Center for Biomedical Research in Neurodegenerative Diseases, National Institute of Health Carlos III, Madrid, Spain
11. Department of Epidemiology, ErasmusMC, Totterdam, The Netherlands
12. Nuffield Department of Population Health Oxford University, Oxford, UK
13. MRC Centre for Neuropsychiatric Genetics and Genomics, , Division of Psychological Medicine and Clinical
14. Université Paris-Saclay, CEA, Centre National de Recherche en Génomique Humaine, 91057, Evry, France
15. German Center for Neurodegenerative Diseases (DZNE Bonn), Bonn, Germany
16. Section Genomics of Neurdegenerative Diseases and Aging, Department of Human Genetics Amsterdam, The Netherlands
17. Institute of Biomedicine, University of Eastern Finland, Kuopio, Finland
18. Brain Institute, Federal University of Rio Grande do Norte, Av. Nascimento de Castro 2155 Natal, Brazil
19. LACDR, Leiden, The Netherlands
20. Dept.of Public Health and Carins Sciences / Geriatrics, Uppsala University, Sweden
21. Centre of Age-Related Medicine, Stavanger University Hospital, Norway
22. Institute of Psychiatry, Psychology & Neuroscience, PO 70, 16 De Crespigny Park, London, UK
23. Department of Surgery, Biochemistry and Molecular Biology, School of Medicine, University of Málaga, Málaga, Spain.
24. Department of Neurology, II B Sant Pau, Hospital de la Santa Creu i Sant Pau, Universitat Autònoma de Barcelona, Barcelona, Spain.
25. Fundació Docència i Recerca MútuaTerrassa and Movement Disorders Unit, Department of Neurology, University Hospital MútuaTerrassa, Terrassa 08221, Barcelona, Spain
26. Memory Disorders Unit, Department of Neurology, Hospital Universitari Mutua de Terrassa, Terrassa, Barcelona, Spain.
27. Laboratorio de Genética. Hospital Universitario Central de Asturias, Oviedo, Spain
28. Servicio de Neurología HOspital Universitario Central de Asturias- Oviedo and Instituto de Investigación Biosanitaria del Principado de Asturias, Oviedo, Spain
29. Centre for Healthy Brain Ageing, School of Psychiatry, Faculty of Medicine, University of New South Wales, Sydney, Australia
30. 1st Department of Neurology, Medical school, Aristotle University of Thessaloniki, Thessaloniki, Makedonia, Greece
31. Alzheimer Hellas, Thessaloniki, Makedonia, Greece
32. School of Medicine and Surgery, University of Milano-Bicocca, Italy
33. Neurology Unit, "San Gerardo" hospital, Monza, Italy
34. Fondazione IRCCS Ca' Granda, Ospedale Policlinico, Milan, Italy
35. Department of Laboratory Diagnostics, III Laboratory of Analysis, Brescia Hospital, Brescia, Italy
36. Unitat Trastorns Cognitius, Hospital Universitari Santa Maria de Lleida, Lleida, Spain
37. Institut de Recerca Biomedica de Lleida (IRBLLeida), Lleida, Spain
38. Department of Clinical Sciences and Community Health, University of Milan, Italy
39. Geriatic Unit, Fondazione Cà Granda, IRCCS Ospedale Maggiore Policlinico, Milan, Italy
40. NORMENT Centre, University of Oslo, Oslo, Norway
41. Université de Paris, EA 4468, APHP, Hôpital Broca, Paris, France
42. Laboratory of Neuropsychiatry, Department of Clinical and Behavioral Neurology, IRCCS Santa Lucia Foundation, Rome, Italy
43. Servei de Neurologia, Hospital Universitari i Politècnic La Fe, Valencia, Spain.
44. CIEN Foundation/Queen Sofia Foundation Alzheimer Center, Madrid, Spain
45. Molecular Markers Laboratory, IRCCS Istituto Centro San Giovanni di Dio Fatebenefratelli, Brescia, Italy
46. Univ. Montpellier, Inserm U1061, Neuropsychiatry: epidemiological and clinical research, PSNREC, Montpellier, France
47. Department of Neuroscience, Psychology, Drug Research and Child Health University of Florence, Florence Italy
48. Azienda Ospedaliero-Universitaria Careggi, Florence, Italy
49. MAC - Memory Clinic, IRCCS Istituto Centro San Giovanni di Dio Fatebenefratelli, Brescia
50. Geriatrics Unit Fondazione Policlinico A. Gemelli IRCCS, Rome, Italy
51. Centre for Neurodegenerative Disorders, Department of Clinical and Experimental Sciences, University of Brescia, Brescia, Italy
52. Department of Neuroscience “Rita Levi Montalcini”, University of Torino, Torino, Italy
53. Experimental Neuro-psychobiology Laboratory, Department of Clinical and Behavioral Neurology, IRCCS Santa Lucia Foundation, Rome, Italy
54. Department of Neurology and Clinical Neurophysiology, University Hospital of Trondheim, Trondheim, Norway
55. Department of Neuromedicine and Movement Science, Norwegian University of Science and Technology, Trondheim, Norway
56. Dementia Centre for Research Collaboration, School of Psychiatry, University of New South Wales, Sydney, Australia
57. Biosciences, School of Science and Technology, Nottingham Trent University, Nottingham UK
58. Centro de Neuropsiquiatría y Neurología de la Conducta (CENECON), Facultad de Medicina, Universidad de Buenos Aires (UBA), C.A.B.A, Buenos Aires, Argentina.
59. Departamento Ciencias Fisiológicas UAII, Facultad de Medicina, UBA, C.A.B.A, Buenos Aires, Argentina.
60. Hospital Interzonal General de Agudos Eva Perón, San Martín, Buenos Aires, Argentina.
61. Unidad de Trastornos del Movimiento, Servicio de Neurología y Neurofisiología. Instituto de Biomedicina de Sevilla (IBiS), Hospital Universitario Virgen del Rocío/CSIC/Universidad de Sevilla, Seville, Spain
62. Institute for Stroke and Dementia Research, Klinikum der Universität München, Ludwig-Maximilians-Universität LMU, Munich, Germany.
63. German Center for Neurodegenerative Diseases (DZNE, Munich), Munich, Germany.
64. Faculty of Medical & Health Sciences, University of Auckland, New Zealand
65. Wales Centre for Ageing & Dementia Research, Swansea University, Wales, New Zealand
66. UFIEC, Instituto de Salud Carlos III, Madrid, Spain
67. Inserm, Bordeaux Population Health Research Center, UMR 1219, Univ. Bordeaux, ISPED, CIC 1401-EC, Univ Bordeaux, Bordeaux, France
68. CHU de Bordeaux, Pole santé publique, Bordeaux, France
69. Grupo de Medicina Xenómica, Centro Nacional de Genotipado (CEGEN-PRB3-ISCIII). Universidade de Santiago de Compostela, Santiago de Compostela, Spain.
70. Fundación Pública Galega de Medicina Xenómica- CIBERER-IDIS, University of Santiago de Compostela, Santiago de Compostela, Spain.
71. Institute of Gerontology and Geriatrics, Department of Medicine and Surgery, University of Perugia Perugia, Italy
72. Normandie Univ, UNIROUEN, Inserm U1245 and CHU Rouen, Department of Genetics and CNR-MAJ, Rouen, France
73. Unit of Clinical Pharmacology, University Hospital of Cagliari, Cagliari, Italy
74. Radboudumc Alzheimer Center, Department of Geriatrics, Radboud University Medical Center, Nijmegen, the Netherlands
75. Institute for Regenerative Medicine, University of Zürich, Schlieren, Switzerland
76. Unidad Clínica de Enfermedades Infecciosas y Microbiología. Hospital Universitario de Valme, Sevilla, Spain
77. Department of Neuroscience, Catholic University of Sacred Heart, Fondazione Policlinico Universitario A. Gemelli IRCCS, Rome, Italy
78. University of Bari, “A. Moro”, Bari, Italy
79. School of Medicine, University of Thessaly, Larissa, Greece
80. University Bordeaux, Inserm, Bordeaux Population Health Research Center, France
81. Department of Neurology, University Medical Center Groningen, the Netherlands
82. Department of Neurology, Bordeaux University Hospital, Bordeaux, France
83. Department of Psychiatry, Psychosomatics and Psychotherapy, Center of Mental Health, University Hospital, Wuerzburg
84. UKDRI@ Cardiff, School of Medicine, Cardiff University, Cardiff, UK
85. Munich Cluster for Systems Neurology (SyNergy), Munich, Germany.
86. Technical University of Munich, School of Medicine, Klinikum rechts der Isar, Department of Psychiatry and Psychotherapy, Munich, Germany
87. Institute of Cognitive Neurology and Dementia Research (IKND), Otto-Von-Guericke University, Magdeburg, Germany.
88. German Center for Neurodegenerative Diseases (DZNE), Magdeburg, Germany.
89. Faculty of Medicine, University of Lisbon, Portugal
90. Department of Psychiatry, Social Medicine Center East- Donauspital, Vienna, Austria
91. Institute of Clinical Medicine, University of Oslo, Oslo, Norway.
92. Dementia Research Centre, UCL Queen Square Institute of Neurology, London, United Kingdom
93. Unidad de Demencias, Servicio de Neurología y Neurofisiología. Instituto de Biomedicina de Sevilla (IBiS), Hospital Universitario Virgen del Rocío/CSIC/Universidad de Sevilla, Seville, Spain
94. Instituto de Investigacion Sanitaria ‘Hospital la Paz’ (IdIPaz), Madrid, Spain
95. Centro de Biología Molecular Severo Ochoa (UAM-CSIC), Madrid, Spain
96. Hospital Universitario la Paz, Madrid, Spain
97. Department of geriatric Psychiatry, Central Institute for Mental Health, Mannheim, University of Heidelberg, Germany
98. Alzheimer Research Center & Memory Clinic, Andalusian Institute for Neuroscience, Málaga, Spain.
99. Hospital Universitario Ramon y Cajal, IRYCIS, Madrid
100. Department of Psychiatry and Psychotherapy, Medical University of Vienna, Vienna, Austria
101. CAEBI, Centro Andaluz de Estudios Bioinformáticos, Sevilla, Spain.
102. Karolinska Institutet, Center for Alzheimer Research, Department NVS, Division of Neurogeriatrics, Stockholm, Sweden
103. Aging Research Center, Department of Neurobiology, Care Sciences and Society, Karolinska Institutet and Stockholm University, Stockholm, Sweden
104. Institute of Public Health, University of Cambridge, UK
105. Department of Child and Adolescent Psychiatry and Psychotherapy, University Hospital of Psychiatry Zurich, University of Zurich, Zurich, Switzerland
106. Neuroscience Center Zurich, University of Zurich and ETH Zurich, Switzerland
107. Zurich Center for Integrative Human Physiology, University of Zurich, Switzerland
108. Human Genetics, School of Life Sciences, Life Sciences Building, University Park, University of Nottingham, Nottingham, UK
109. A.I Virtanen Institute for Molecular Sciences, University of Eastern Finland, Kuopio, Finland
110. Department of Neurology, Medical School, University of Cyprus, Cyprus
111. Sorbonne University, GRC n° 21, Alzheimer Precision Medicine Initiative (APMI), AP-HP, Pitié-Salpêtrière Hospital, Boulevard de l'hôpital, Paris, France
112. Eisai Inc., Neurology Business Group, 100 Tice Blvd, Woodcliff Lake, NJ 07677, USA
113. Reta Lila Weston Research Laboratories, Department of Molecular Neuroscience, UCL Institute of Neurology, London, UK.
114. Institute of Human Genetics, University of Bonn, School of Medicine & University Hospital Bonn, Bonn, Germany
115. Insitute of Clinical Medicine - Neurology, University of Eastern, Kuopio, Finland
116. Institute of Clinical Medicine – Internal Medicine, University of Eastern Finland, Kuopio, Finland
117. Clinical and Experimental Science, Faculty of Medicine, University of Southampton, Southampton, UK.
118. Unit for Hereditary dementias, Karolinska University Hospital-Solna, Stockholm, Sweden
119. Division of Clinical Geriatrics, Center for Alzheimer Research, Care Sciences and Society (NVS), Karolinska Institutet, Stockholm, Sweden
120. Institute of Public Health and Clinical Nutrition, University of Eastern Finland, Kuopio, Finland
121. Neuroepidemiology and Ageing Research Unit, School of Public Health, Imperial College London, London, United Kingdom
122. Stockholms Sjukhem, Research & Development Unit, Stockholm, Sweden
123. Department of Psychiatry and Psychotherapy, Universitätsklinikum Erlangen, and Friedrich-Alexander Universität Erlangen-Nürnberg, Erlangen, Germany.
124. Laboratory of Cognitive Neuroscience, School of Psychology, Aristotle University of Thessaloniki, Thessaloniki, Greece
125. Neurology Service, Marqués de Valdecilla University Hospital (University of Cantabria and IDIVAL), Santander, Spain.
126. Stockholm Gerontology Research Center, Stockholm, Sweden
127. Public Health Promotion Unit, Finnish Institute for Health and Welfare, Helsinki, Finland
128. Memory Clinic, Department of Neurology, Charles University, 2nd Faculty of Medicine and Motol University Hospital, Czech Republic
129. International Clinical Research Center, St. Anne’s University Hospital Brno, Brno, Czech Republic
130. Department of Neurology. Hospital Universitario Donostia. OSAKIDETZA-Servicio Vasco de Salud, San Sebastian, Spain
131. School of Health Sciences, Bangor University, UK
132. Unit of Neurology, University of Parma and AOU, Parma, Italy
133. Institute of Neurology, Catholic University of the Sacred Heart, Rome, Itlay
134. MRC Prion Unit at UCL, UCL Institute of Prion Diseases, London, UK
135. Clinic of Neurology, UH "Alexandrovska", Medical University - Sofia, Sofia, Bulgaria
136. Institute for Urban Public Health, University Hospital of University Duisburg-Essen, Essen, Germany
137. Department of Neurology, ErasmusMC, Rotterdam, The Netherlands
138. Neurological Tissue Bank of the Biobanc-Hospital Clinic-IDIBAPS, Institut d'Investigacions Biomèdiques August Pi i Sunyer, Barcelona, Spain.
139. Alzheimer’s disease and other cognitive disorders Unit. Neurology Department, Hospital Clinic, Barcelona, Spain
140. Laboratory of Brain Aging and Neurodegeneration- FIL-CONICET, Buenos Aires, Argentina
141. Human Genetics, School of Life Sciences, University of Nottingham, UK
142. IRCCS Fondazione Don Carlo Gnocchi, Florence, Italy
143. Department of Clinical Biochemistry, Herlev and Gentofte Hospital, Herlev, Denmark
144. Department of Clinical Medicine, University of Copenhagen, Copenhagen, Denmark
145. DIMEC, University of Parma, Parma, Italy
146. Institut de Biomedicina de València-CSIC (valència, Spain) CIBERNED.
147. Unitat Mixta de de Neurología y Genética, Institut d'Investigació Sanitària La Fe (València, Spain)
148. Univ. Lille, CNRS, Inserm, CHU Lille, Institut Pasteur de Lille, US 41-UMS 2014-PLBS, bilille, Lille, France.
149. Institute of Psychiatry and Psychotherapy, Charité-Universitätsmedizin Berlin, Corporate Member of Freie Universität Berlin, Humboldt-Universität Zu Berlin, and Berlin Institute of Health, Berlin, Germany.
150. German Center for Neurodegenerative Diseases (DZNE), Berlin, Germany.
151. Department of Biomedical Sciences, University of Cagliari, Italy
152. CHUV, Old Age Psychiatry, Department of Psychiatry, Lausanne, Switzerland
153. Old Age Psychiatry, Department of Psychiatry, Lausanne University Hospital, Lausanne, Switzerland
154. Department of Geriatric Psychiatry, University Hospital of Psychiatry Zürich, Zürich, Switzerland
155. Department of Neuropsychiatry and Laboratory of Molecular Psychiatry, Charité, Charitéplatz 1, 10117 Berlin, Germany
156. Department of Clinical Biochemistry, Rigshospitalet, Copenhagen, Denmark
157. Maastricht University, Department of Psychiatry & Neuropsychologie, Alzheimer Center Limburg, Maastricht, the Netherlands
158. Depatamento de Especialidades Quirúrgicas, Bioquímica e Inmunología. Facultad de Medicina. Universidad de Málaga. Málaga, Spain
159. Delft Bioinformatics Lab, Delft University of Technology, Delft, The Netherlands
160. Institute of Social Medicine, Occupational Health and Public Health, University of Leipzig, 04103 Leipzig, Germany.
161. Center of Mental Health, Clinic and Policlinic of Psychiatry, Psychosomatics and Psychotherapy, University Hospital of Würzburg, Wuerzburg, Germany
162. Department of Research and Innovation, Helse Fonna, Haugesund Hospital, Haugesund, Norway.
163. The University of Bergen, Institute of Clinical Medicine (K1), Bergen Norway
164. Departamento de Especialidades Quirúrgicas, Bioquímicas e Inmunología, School of Medicine, University of Málaga, Málaga, Spain.
165. Department of Neuroscience and Mental Health, AOU Città della Salute e della Scienza di Torino, Torino, Italy
166. Athens Association of Alzheimer’s disease and Related Disorders, Athens, Greece
167. Department of Geriatrics, St. Olav’s Hospital, Trondheim University Hospital, Norway
168. Department of Immunology, Hospital Universitario Doctor Negrín, Las Palmas de Gran Canaria, Spain.
169. Neurology department-Hospital Clínic, IDIBAPS, Universitat de Barcelona, Barcelona, Spain.
170. Taub Institute for Research in Alzheimer’s Disease and the Aging Brain, The Gertrude H. Sergievsky Center, Depatment of Neurology, Columbia University, New York, NY
171. 1st Department of Neurology, Aiginition Hospital, National and Kapodistrian University of Athens, Medical School, Greece
172. LVR-Hospital Essen, Department of Psychiatry and Psychotherapy, Medical Faculty, University of Duisburg-Essen, Virchowstr. 174, 45147 Essen, Germany
173. Department of Primary Medical Care, University Medical Centre Hamburg-Eppendorf, 20246 Hamburg, Germany.
174. Institute of Medical Biometry, Informatics and Epidemiology, University Hospital of Bonn, Bonn, Germany.
175. Department of Geriatric Medicine, Oslo University Hospital, Oslo, Norway
176. Laboratory for Advanced Hematological Diagnostics, Department of Hematology and Stem Cell Transplant, Lecce, Italy
177. Department of Biomedical Sciences, Section of Neuroscience and Clinical Pharmacology, University of Cagliari, Italy
178. Department of Psychiatry, Namsos Hospital, Namsos, Norway
179. Department of Internal medicine and Biostatistics, ErasmusMC, Rooterdam, The Netherlands
180. Department of Radiology&Nuclear medicine, ErasmusMC, Totterdam, The Netherlands
181. Laboratory for Cognitive Neurology, Department of Neurosciences, University of Leuven, Belgium
182. Neurology Department, University Hospitals Leuven, Leuven, Belgium
183. Department of Psychiatry and Psychotherapy, University Medical Center Goettingen, Goettingen, Germany
184. Department of Psychiatry, Harvard Medical School, McLean Hospital, Belmont, MA, USA
185. Normandie Univ, UNIROUEN, Inserm U1245, CHU Rouen, Department of Neurology and CNR-MAJ, F 76000, Normandy Center for Genomic and Personalized Medicine, Rouen, France
186. German Center for Neurodegenerative Diseases (DZNE), Goettingen, Germany
187. Medical Science Department, iBiMED, Aveiro, Portugal
188. Department of Nutrition and Diatetics, Harokopio University, Athens, Greece
189. Neurosciences Area. Instituto Biodonostia. San Sebastian, Spain
190. Department of Clinical Biochemistry, Hematology and Immunology, Na Homolce Hospital, Prague, Czech republic
191. Institute of Gerontology and Geriatrics, Department of Medicine, University of Perugia Perugia (Italy)
192. Insitute of Biomedicine, University of Eastern Finland, Finland
193. Center for Life Course Health Research, University of Oulu, Oulu, Finland
194. Medical Research Center Oulu, Oulu University Hospital, Oulu, Finland
195. University of Helsinki and Helsinki University Hospital, Helsinki, Finland
196. Division of Psychological Medicine and Clinial Neurosciences, MRC Centre for Neuropsychiatric Genetics and Genomics, Cardiff University, UK
197. Kings College London, Institute of Psychiatry, Psychology and Neuroscience, UK
198. Division of Psychiatry, University College London, UK
199. Institute of Public Health, University of Cambridge, Cambridge, UK
200. Institute of Genetics, Queens Medical Centre, University of Nottingham, Nottingham, UK
201. Department of Neurodegenerative Disease, MRC Prion Unit at UCL, Institute of Prion Diseases, London, UK
202. Ageing Group, Centre for Public Health, School of Medicine, Dentistry and Biomedical Sciences, Queen's University Belfast, UK
203. The Wellcome Trust Sanger Institute, Wellcome Trust Genome Campus, Hinxton, Cambridge, UK.
204. Dementia Research Centre, Department of Neurodegenerative Disease, UCL Institute of Neurology, London, UK
205. Mercer's Institute for Research on Ageing, St James' Hospital, Dublin, Ireland
206. Department of Molecular Neuroscience, UCL, Institute of Neurology, London, UK
207. School of Biotechnology, Dublin City University, Dublin, Ireland
208. Centre for Public Health, School of Medicine, Dentistry and Biomedical Sciences, Queens University, Belfast, UK
209. Department of Psychiatry, University of Oxford, Oxford, UK
210. Department of Basic and Clinical Neuroscience, Institute of Psychiatry, Psychology and Neuroscience, Kings College London, London UK
211. Genetic Epidemiology, QIMR Berghofer Medical Research Institute, Herston, Queensland, Australia
212. Division of Neuroscience and Experimental Psychology, School of Biological Sciences, Faculty of Biology, Medicine and Health, University of Manchester, Manchester Academic Health Science Centre, Manchester M13 9PT, UK
213. Oxford Project to Investigate Memory and Ageing (OPTIMA), University of Oxford, Level 4, John Radcliffe Hospital, Oxford, UK
214. Department of Mental Health Sciences, University College London, London, UK
215. Ageing Group, Centre for Public Health, School of Medicine, Dentistry and Biomedical Sciences, Queen’s University Belfast, Belfast, UK.
216. Dementia Research Centre, UCL, London, UK
217. Servei de Neurologia. Hospital Clínic Universitari de València, Spain
218. Department of Radiology, University Hospital Bonn, Bonn, Germany
219. German Center for Neurodegenerative Diseases (DZNE), Tübingen, Germany
220. Section for Dementia Research, Hertie Institute for Clinical Brain Research and Department of Psychiatry, Tübingen, Germany
221. Department of Psychiatry and Psychotherapy, University Hospital, LMU Munich, Munich, Germany
222. Service of Neurology, University Hospital Marqués de Valdecilla, IDIVAL, University of Cantabria, Santander, Spain
223. Molecular Medicine Center, Department of Medical chemistry and biochemistry, Medical University of Sofia, Bulgaria
224. Department of Psychiatry, Psychosomatics and Psychotherapy, Center of Mental Health, University Hospital of Würzburg, Germany
225. ENYS (Estudio en Neurociencias y Sistemas Complejos) CONICET- Hospital El Cruce "Nestor Kirchner"- UNAJ, Argentina
226. HIGA Eva Perón, Buenes Aires, Agentina
227. Neurología Clinica, Buenes Aires, Agentina
228. Dirección de Atención de Adultos Mayores del Min. Salud Desarrollo Social y Deportes de la Pcia. de Mendoza, Argentina
229. Laboratorio de Genética Forense del Ministerio Público de la Pcia. de La Pampa, Argentina
230. Fundacion Sinapsis, Santa Rosa, Argentina
231. Hospital Dr. Lucio Molas, Santa Rosa; Fundacion Ayuda Enfermo Renal y Alta Complejidad (FERNAC), Santa Rosa, Argentina
232. Laboratory of Brain Aging and Neurodegeneration- FIL, Buneos Aires, Argentina
233. Centro de Neuropsiquiatría y Neurología de la Conducta (CENECON), Facultad de Medicina, Universidad de Buenos Aires (UBA), C.A.B.A, Buenos Aires, Argentina
234. Center for Neurosciences, Vrije Universiteit Brussel (VUB), Brussels, Belgium
235. Reference Center for Biological Markers of Dementia (BIODEM), Institute Born-Bunge, University of Antwerp, Antwerp, Belgium
236. Institute Born-Bunge, University of Antwerp, Antwerp, Belgium
237. Department of Neurology, UZ Brussel, Brussels, Belgium
238. Fondazione IRCCS, Istituto Neurologico Carlo Besta, Milan Italy
239. Department of Clinical Biochemistry, Herlev and Gentofte Hospital, Herlev Denmark
240. Centre for Memory Disturbances, Lab of Clinical Neurochemistry, Section of Neurology, University of Perugia, Italy
241. University of Milan, Milan, Italy
242. Department of Neurology, UMC Utrecht Brain Center, Utrecht, the Netherlands
243. Translational Health Sciences, Bristol Medical School, University of Bristol, Bristol, BS16 1LE, UK
244. Univ Lille Inserm 1172, CHU Clinical and Research Memory Research Centre (CMRR) of Distalz, Licend, Lille France
245. Neurodegenerative Brain Diseases Group, VIB Center for Molecular Neurology, VIB, Antwerp, Belgium
246. Cambridge Institute for Medical Research and UK Dementia Research Institute, University of Cambridge, Cambridge, UK
247. German Center for Neurodegenerative Diseases (DZNE), Rostock, Germany
248. Centro de Investigación Biomédica en Red de Diabetes y Enfermedades Metabólicas Asociadas, CIBERDEM, Spain, Hospital Clínico San Carlos, Madrid, Spain
249. Glenn Biggs Institute for Alzheimer’s and Neurodegenerative Diseases, San Antonio, TX, USA

## 4 – 2. Supplementary list of authors FinnGen

Aarno Palotie^1^, Mark Daly^1^, Howard Jacob^2^, Athena Matakidou^3^, Heiko Runz^4^, Sally John^4^, Robert Plenge^5^, Mark McCarthy^6^, Julie Hunkapiller^6^, Meg Ehm^7^, Dawn Waterworth^7^, Caroline Fox^8^, Anders Malarstig^9^, Kathy Klinger^10^, Kathy Call10, Tim Behrens^11^, Patrick Loerch^12^, Tomi Mäkelä^13^, Jaakko Kaprio^1^, Petri Virolainen^14^, Kari Pulkki^14^, Terhi Kilpi^15^, Markus Perola^15^, Jukka Partanen^16^, Anne Pitkäranta^17^, Riitta Kaarteenaho^18^, Seppo Vainio^18^, Miia Turpeinen^18^, Raisa Serpi^18^, Tarja Laitinen^19^, Johanna Mäkelä^19^, Veli-Matti Kosma^20^, Urho Kujala^21^, Outi Tuovila^22^, Minna Hendolin^22^, Raimo Pakkanen^22^, Jeff Waring^2^, Bridget Riley-Gillis^2^, Jimmy Liu^4^, Shameek Biswas^5^, Julie Hunkapiller^6^, Dorothee Diogo^8^, Catherine Marshall^9^, Xinli Hu^9^, Matthias Gossel^10^, Robert Graham^11^, Tim Behrens^11^, Beryl Cummings^12^, Samuli Ripatti^1^, Johanna Schleutker^14^, Mikko Arvas^16^, Olli Carpén^17^, Reetta Hinttala^18^, Johannes Kettunen^18^, Arto Mannermaa^20^, Jari Laukkanen^21^, Hilkka Soininen^23^, Valtteri Julkunen^23^, Anne Remes^23^, Reetta Kälviäinen^23^, Jukka Peltola^24^, Pentti Tienari^25^, Juha Rinne^26^, Adam Ziemann^2^, Jeffrey Waring^2^, Sahar Esmaeeli^2^, Nizar Smaoui^2^, Anne Lehtonen^2^, Susan Eaton^4^, Sanni Lahdenperä^4^, Janet van Adelsberg^5^, Shameek Biswas^5^, John Michon^6^, Geoff Kerchner^6^, Natalie Bowers^6^, Edmond Teng^6^, John Eicher^8^, Vinay Mehta^8^, Padhraig Gormley^8^, Kari Linden^9^, Christopher Whelan^9^, Fanli Xu^7^, David Pulford^7^, Martti Färkkilä^25^, Sampsa Pikkarainen^25^, Airi Jussila^27^, Timo Blomster^28^, Mikko Kiviniemi^29^, Markku Voutilainen^26^, Bob Georgantas^2^, Graham Heap^2^, Fedik Rahimov^2^, Keith Usiskin^5^, Tim Lu^6^, Danny Oh^6^, Kirsi Kalpala^9^, Melissa Miller^9^, Linda McCarthy^7^, Kari Eklund^25^, Antti Palomäki^26^, Pia Isomäki^27^, Laura Pirilä^26^, Oili Kaipiainen-Seppänen^29^, Johanna Huhtakangas^28^, Bob Georgantas^2^, Fedik Rahimov^2^, Apinya Lertratanakul^2^, Marla Hochfeld^5^, Kirsi Kalpala^9^, Nan Bing^9^, Jorge Esparza Gordillo^7^, Nina Mars^1^, Margit Pelkonen^29^, Paula Kauppi^25^, Hannu Kankaanranta^24^, Terttu Harju^28^, David Close^3^, Steven Greenberg^5^, Hubert Chen^6^, Jo Betts^7^, Soumitra Ghosh^7^, Veikko Salomaa^30^, Teemu Niiranen^30^, Markus Juonala^26^, Kaj Metsärinne^26^, Mika Kähönen^27^, Juhani Junttila^28^, Markku Laakso^23^, Jussi Pihlajamäki^23^, Juha Sinisalo^25^, Marja-Riitta Taskinen^25^, Tiinamaija Tuomi^25^, Ben Challis^3^, Andrew Peterson^6^, Audrey Chu^8^, Jaakko Parkkinen^9^, Melissa Miller^9^, Anthony Muslin^10^, Dawn Waterworth^7^, Heikki Joensuu^25^, Tuomo Meretoja^25^, Lauri Aaltonen^25^, Johanna Mattson^25^, Annika Auranen^24^, Peeter Karihtala^28^, Saila Kauppila^28^, Päivi Auvinen^23^, Klaus Elenius^26^, Relja Popovic^2^, Jennifer Schutzman^6^, Andrey Loboda^8^, Aparna Chhibber^8^, Heli Lehtonen^9^, Stefan McDonough^9^, Marika Crohns^10^, Diptee Kulkarni^7^, Kai Kaarniranta^23^, Joni A Turunen^25^, Terhi Ollila^25^, Sanna Seitsonen^25^, Hannu Uusitalo^24^, Vesa Aaltonen^26^, Hannele Uusitalo-Järvinen^24^, Marja Luodonpää^28^, Nina Hautala^28^, Stephanie Loomis^4^, Erich Strauss^6^, Hao Chen^6^, Anna Podgornaia^8^, Joshua Hoffman^7^, Kaisa Tasanen^28^, Laura Huilaja^28^, Katariina Hannula-Jouppi^25^, Teea Salmi^27^, Sirkku Peltonen^25^, Leena Koulu^25^, Ilkka Harvima^23^, Kirsi Kalpala^9^, Ying Wu^9^, David Choy^6^, Fedik Rahimov^2^, Dawn Waterworth^7^, Pirkko Pussinen^25^, Aino Salminen^25^, Tuula Salo^25^, David Rice^25^, Pekka Nieminen^25^, Ulla Palotie^25^, Maria Siponen^23^, Liisa Suominen^23^, Päivi Mäntylä^23^, Ulvi Gursoy^26^, Vuokko Anttonen^28^, Kirsi Sipilä^28^, Justin Wade Davis^2^, Bridget Riley-Gillis^2^, Danjuma Quarless^2^, Fedik Rahimov^2^, Sahar Esmaeeli^2^, Slavé Petrovski^3^, Eleonor Wigmore^3^, Chia-Yen Chen^4^, Paola Bronson4, Ellen Tsai^4^, Yunfeng Huang^4^, Joseph Maranville^5^, Elmutaz Shaikho Elhaj Mohammed^5^, Samir Wadhawan^31^, Erika Kvikstad^31^, Minal Caliskan^31^, Diana Chang^6^, Tushar Bhangale^6^, Natalie Bowers^6^, Sarah Pendergrass^6^, Emily Holzinger^8^, Xing Chen^9^, Åsa Hedman^9^, Karen S King^7^, Clarence Wang^10^, Ethan Xu^10^, Franck Auge^10^, Clement Chatelain^10^, Deepak Rajpal^10^, Dongyu Liu^10^, Katherine Call^10^, Tai-he Xia^10^, Matt Brauer^11^, Mitja Kurki^1^, Samuli Ripatti^1^, Juha Karjalainen^1^, Aki Havulinna^1^, Anu Jalanko^1^, Priit Palta^1^, Pietro della Briotta Parolo^1^, Wei Zhou^32^, Susanna Lemmelä^1^, Manuel Rivas^33^, Jarmo Harju^1^, Arto Lehisto^1^, Andrea Ganna^1^, Vincent Llorens^1^, Hannele Laivuori^1^, Sina Rüeger^1^, Mari E Niemi^1^, Taru Tukiainen^1^, Mary Pat Reeve^1^, Henrike Heyne^1^, Nina Mars^1^, Kimmo Palin^34^, Javier Garcia-Tabuenca^35^, Harri Siirtola^35^, Tuomo Kiiskinen^1^, Tuomo Kiiskinen^1^, Jiwoo Lee^1^, Kristin Tsuo^1^, Amanda Elliott^1^, Kati Kristiansson^15^, Kati Hyvärinen^36^, Jarmo Ritari^36^, Miika Koskinen^17^, Katri Pylkäs^18^, Marita Kalaoja^18^, Minna Karjalainen^18^, Tuomo Mantere^18^, Eeva Kangasniemi^19^, Sami Heikkinen^20^, Sami Heikkinen^21^, Eija Laakkonen^21^, Csilla Sipeky^37^, Samuel Heron^37^, Antti Karlsson^14^, Dhanaprakash Jambulingam^37^, Venkat Subramaniam Rathinakannan^37^, Anu Jalanko^1^, Risto Kajanne^1^, Mervi Aavikko^1^, Manuel González Jiménez^1^, Mitja Kurki^1^, Juha Karjalainen^1^, Pietro della Briotta Parola^1^, Sina Rüeger^1^, Arto Lehistö^1^, Masahiro Kanai^32^, Hannele Laivuori^1^, Aki Havulinna^1^, Susanna Lemmelä^1^, Tuomo Kiiskinen^1^, Mari Kaunisto^1^, Jarmo Harju^1^, Elina Kilpeläinen^1^, Timo P. Sipilä^1^, Georg Brein^1^, Ghazal Awaisa^1^, Anastasia Shcherban^1^, Kati Donner^1^, Timo P. Sipilä^1^, Anu Loukola^17^, Päivi Laiho^15^, Tuuli Sistonen^15^, Essi Kaiharju^15^, Markku Laukkanen^15^, Elina Järvensivu^15^, Sini Lähteenmäki^15^, Lotta Männikkö^15^, Regis Wong^15^, Hannele Mattsson^15^, Kati Kristiansson^15^, Susanna Lemmelä^1^, Tero Hiekkalinna^15^, Teemu Paajanen^15^, Priit Palta^1^, Kalle Pärn^1^, Harri Siirtola^35^, Javier Gracia-Tabuenca^35^

1. Institute for Molecular Medicine Finland, HiLIFE, University of Helsinki, Finland
2. Abbvie, Chicago, IL, United States
3. Astra Zeneca, Cambridge, United Kingdom
4. Biogen, Cambridge, MA, United States
5. Celgene, Summit, NJ, United States
6. Genentech, San Francisco, CA, United States
7. GlaxoSmithKline, Brentford, United Kingdom
8. Merck, Kenilworth, NJ, United States
9. Pfizer, New York, NY, United States
10. Sanofi, Paris, France
11. Maze Therapeutics, San Francisco, CA, United States
12. Janssen Biotech, Beerse, Belgium
13. HiLIFE, University of Helsinki, Finland, Finland
14. Auria Biobank / University of Turku / Hospital District of Southwest Finland, Turku, Finland
15. THL Biobank / The National Institute of Health and Welfare Helsinki, Finland
16. Finnish Red Cross Blood Service / Finnish Hematology Registry and Clinical Biobank, Helsinki
17. Helsinki Biobank / Helsinki University and Hospital District of Helsinki and Uusimaa, Helsinki
18. Northern Finland Biobank Borealis / University of Oulu / Northern Ostrobothnia Hospital District, Oulu, Finland
19. Finnish Clinical Biobank Tampere **/** University of Tampere / Pirkanmaa Hospital District, Tampere, Finland
20. Biobank of Eastern Finland / University of Eastern Finland / Northern Savo Hospital District, Kuopio, Finland
21. Central Finland Biobank / University of Jyväskylä / Central Finland Health Care District, Jyväskylä, Finland
22. Business Finland, Helsinki, Finland
23. Northern Savo Hospital District, Kuopio, Finland
24. Pirkanmaa Hospital District, Tampere, Finland
25. Hospital District of Helsinki and Uusimaa, Helsinki, Finland
26. Hospital District of Southwest Finland, Turku, Finland
27. Pirkanmaa Hospital District, Tampere, Finland
28. Northern Ostrobothnia Hospital District, Oulu, Finland
29. Northern Savo Hospital District, Kuopio, Finland
30. The National Institute of Health and Welfare Helsinki, Finland
31. Bristol-Meyers-Squibb
32. Broad Institute, Cambridge, MA, United States
33. University of Stanford, Stanford, CA, United States
34. University of Helsinki, Helsinki, Finland
35. University of Tampere, Tampere, Finland
36. Finnish Red Cross Blood Service, Helsinki, Finland
37. University of Turku, Turku, Finland

## 4 – 3. Additional Support for EADB cohorts

We thank the many study participants, researchers, and staff for collecting and contributing to the data, the high-performance computing service at the University of Lille, and the staff at CEA-CNRGH for their help with sample preparation and genotyping, and excellent technical assistance. We thank Antonio Pardinas for his help. We thank the Netherlands Brain Bank^.^

This work was funded by a grant (European Alzheimer DNA BioBank, EADB) from the EU Joint Programme – Neurodegenerative Disease Research (JPND). Inserm UMR1167 is also funded by the Inserm, Institut Pasteur de Lille, Lille Métropole Communauté Urbaine, and the French government’s LABEX DISTALZ program (development of innovative strategies for a transdisciplinary approach to Alzheimer’s disease).

The work for this manuscript was further supported by the CoSTREAM project (www.costream.eu) and funding from the European Union's Horizon 2020 research and innovation programme under grant agreement No 667375. This work is also funded by la fondation pour la recherché médicale (FRM) (EQU202003010147) Italian Ministry of Health (Ricerca Corrente); Ministero dell'Istruzione, del l'Università e della Ricerca–MIUR project “Dipartimenti di Eccellenza 2018–2022” to Department of Neuroscience “Rita Levi Montalcini”, University of Torino (IR), and AIRAlzh Onlus-ANCC-COOP (SB); Partly supported by “Ministero della Salute”, I.R.C.C.S. Research Program, Ricerca Corrente 2018-2020, Linea n. 2 “Meccanismi genetici, predizione e terapie innovative delle malattie complesse” and by the “5 x 1000” voluntary contribution to the Fondazione I.R.C.C.S. Ospedale “Casa Sollievo della Sofferenza”; and RF-2018-12366665, Fondi per la ricerca 2019 (Sandro Sorbi). Copenhagen General Population Study (CGPS): We thank staff and participants of the CGPS for their important contributions. Karolinska Institutet AD cohort: Dr. C.G. and co-authors of the Karolinska Institutet AD cohort report grants from Swedish Research Council (VR) 2015-02926, 2018-02754, 2015-06799, Swedish Alzheimer Foundation, Stockholm County Council ALF and resarch school, Karolinska Institutet StratNeuro, Swedish Demensfonden, and Swedish brain foundation, during the conduct of the study. ADGEN: This work was supported by Academy of Finland (grant numbers 307866); Sigrid Jusélius Foundation; the Strategic Neuroscience Funding of the University of Eastern Finland; EADB project in the JPNDCO-FUND program (grant number 301220). CBAS: Supported by the project no. LQ1605 from the National Program of Sustainability II (MEYS CR), Supported by Ministry of Health of the Czech Republic, grant nr. NV19-04-00270 (All rights reserved), Grant Agency of Charles University Grants No. 693018 and 654217; the Ministry of Health, Czech Republic―conceptual development of research organization, University Hospital Motol, Prague, Czech Republic Grant No. 00064203; the Czech Ministry of Health Project AZV Grant No. 16―27611A; and Institutional Support of Excellence 2. LF UK Grant No. 699012. CNRMAJ-Rouen: This study received fundings from the Centre National de Référence Malades Alzheimer Jeunes (CNRMAJ). The Finnish Geriatric Intervention Study for the Prevention of Cognitive Impairment and Disability (FINGER) data collection was supported by grants from the Academy of Finland, La Carita Foundation, Juho Vainio Foundation, Novo Nordisk Foundation, Finnish Social Insurance Institution, Ministry of Education and Culture Research Grants, Yrjö Jahnsson Foundation, Finnish Cultural Foundation South Osthrobothnia Regional Fund, and EVO/State Research Funding grants of University Hospitals of Kuopio, Oulu and Turku, Seinäjoki Central Hospital and Oulu City Hospital, Alzheimer's Research & Prevention Foundation USA, AXA Research Fund, Knut and Alice Wallenberg Foundation Sweden, Center for Innovative Medicine (CIMED) at Karolinska Institutet Sweden, and Stiftelsen Stockholms sjukhem Sweden. FINGER cohort genotyping was funded by EADB project in the JPND CO-FUND (grant number 301220). Research at the Belgian EADB site is funded in part by the Alzheimer Research Foundation (SAO-FRA), The Research Foundation Flanders (FWO), and the University of Antwerp Research Fund. FK is supported by a BOF DOCPRO fellowship of the University of Antwerp Research Fund. SNAC-K is financially supported by the Swedish Ministry of Health and Social Affairs, the participating County Councils and Municipalities, and the Swedish Research Council. BDR Bristol: We would like to thank the South West Dementia Brain Bank (SWDBB) for providing brain tissue for this study. The SWDBB is part of the Brains for Dementia Research programme, jointly funded by Alzheimer’s Research UK and Alzheimer’s Society and is supported by BRACE (Bristol Research into Alzheimer’s and Care of the Elderly) and the Medical Research Council. BDR Manchester: We would like to thank the Manchester Brain Bankfor providing brain tissue for this study. The Manchester Brain Bank is part of the Brains for Dementia Research programme, jointly funded by Alzheimer’s Research UK and Alzheimer’s Society. BDR KCL: Human post-mortem tissue was provided by the London Neurodegenerative Diseases Brain Bank which receives funding from the UK Medical Research Council and as part of the Brains for Dementia Research programme, jointly funded by Alzheimer’s Research UK and the Alzheimer’s Society. The CFAS Wales study was funded by the ESRC (RES-060-25-0060) and HEFCW as ‘Maintaining function and well-being in later life: a longitudinal cohort study’. We are grateful to the NISCHR Clinical Research Centre for their assistance in tracing participants and in interviewing and in collecting blood samples, and to general practices in the study areas for their cooperation. MRC: We thank all individuals who participated in this study. Cardiff University was supported by the Alzheimer's Society (AS; grant RF014/164) and the Medical Research Council (MRC; grants G0801418/1, MR/K013041/1, MR/L023784/1) (R.S. is an AS Research Fellow). Cardiff University was also supported by the European Joint Programme for Neurodegenerative Disease (JPND; grant MR/L501517/1), Alzheimer's Research UK (ARUK; grant ARUK-PG2014-1), the Welsh Assembly Government (grant SGR544:CADR), Brain’s for dementia Research and a donation from the Moondance Charitable Foundation. Cardiff University acknowledges the support of the UK Dementia Research Institute, of which J.W. is an associate director. Cambridge University acknowledges support from the MRC. Patient recruitment for the MRC Prion Unit/UCL Department of Neurodegenerative Disease collection was supported by the UCLH/UCL Biomedical Centre and NIHR Queen Square Dementia Biomedical Research Unit. The University of Southampton acknowledges support from the AS. King's College London was supported by the NIHR Biomedical Research Centre for Mental Health and the Biomedical Research Unit for Dementia at the South London and Maudsley NHS Foundation Trust and by King's College London and the MRC. ARUK and the Big Lottery Fund provided support to Nottingham University. A.Ram. : Part of the work was funded by the JPND EADB grant (German Federal Ministry of Education and Research (BMBF) grant: 01ED1619A). A. Ram. is also supported by the German Research Foundation (DFG) grants Nr: RA 1971/6-1, RA1971/7-1, and RA 1971/8-1. German Study on Ageing, Cognition and Dementia in Primary Care Patients (AgeCoDe): This study/publication is part of the German Research Network on Dementia (KND), the German Research Network on Degenerative Dementia (KNDD; German Study on Ageing, Cognition and Dementia in Primary Care Patients; AgeCoDe), and the Health Service Research Initiative (Study on Needs, health service use, costs and health-related quality of life in a large sample of oldestold primary care patients (85+; AgeQualiDe)) and was funded by the German Federal Ministry of Education and Research (grants KND: 01GI0102, 01GI0420, 01GI0422, 01GI0423, 01GI0429, 01GI0431, 01GI0433, 01GI0434; grants KNDD: 01GI0710, 01GI0711, 01GI0712, 01GI0713, 01GI0714, 01GI0715, 01GI0716; grants Health Service Research Initiative: 01GY1322A, 01GY1322B, 01GY1322C, 01GY1322D, 01GY1322E, 01GY1322F, 01GY1322G). VITA study: The support of the Ludwig Boltzmann Society and the AFI Germany have supported the VITA study. The former VITA study group should be acknowledged: W. Danielczyk, G. Gatterer, K Jellinger, S Jugwirth, KH Tragl, S Zehetmayer. Vogel Study: This work was financed by a research grant of the ‘‘Vogelstiftung Dr. Eckernkamp’’. HELIAD study: This study was supported by the grants: IIRG-09-133014 from the Alzheimer’s Association, 189 10276/8/9/2011 from the ESPA-EU program Excellence Grant (ARISTEIA) and the ΔΥ2β/οικ.51657/14.4.2009 of the Ministry for Health and Social Solidarity (Greece). Biobank Department of Psychiatry, UMG: Prof. Jens Wiltfang is supported by an Ilídio Pinho professorship and iBiMED (UID/BIM/04501/2013), and FCT project PTDC/DTP_PIC/5587/2014 at the University of Aveiro, Portugal. Lausanne study: This work was supported by grants from the Swiss National Research Foundation (SNF 320030_141179). PAGES study: Harald Hampel is an employee of Eisai Inc. During part of this work he was supported by the AXA Research Fund, the “Fondation partenariale Sorbonne Université” and the “Fondation pour la Recherche sur Alzheimer”, Paris, France. Mannheim, Germany Biobank: Department of geriatric Psychiatry, Central Institute for Mental Health, Mannheim, University of Heidelberg, Germany. Genotyping for the Swedish Twin Studies of Aging was supported by NIH/NIA grant R01 AG037985. Genotyping in TwinGene was supported by NIH/NIDDK U01 DK066134. WvdF is recipient of Joint Programming for Neurodegenerative Diseases (JPND) grants PERADES (ANR-13-JPRF-0001) and EADB (733051061). Gothenburg Birth Cohort (GBC) Studies: We would like to thank UCL Genomics for performing the genotyping analyses. The studies were supported by The Stena Foundation, The Swedish Research Council (2015-02830, 2013-8717), The Swedish Research Council for Health, Working Life and Wellfare (2013-1202, 2005-0762, 2008-1210, 2013-2300, 2013- 2496, 2013-0475), The Brain Foundation, Sahlgrenska University Hospital (ALF), The Alzheimer’s Association (IIRG-03-6168), The Alzheimer’s Association Zenith Award (ZEN-01-3151), Eivind och Elsa K:son Sylvans Stiftelse, The Swedish Alzheimer Foundation. Clinical AD, Sweden: We would like to thank UCL Genomics for performing the genotyping analyses. Barcelona Brain Biobank: Brain Donors of the Neurological Tissue Bank of the Biobanc-Hospital Clinic-IDIBAPS and their families for their generosity. Hospital Clínic de Barcelona Spanish Ministry of Economy and Competitiveness-Instituto de Salud Carlos III and Fondo Europeo de Desarrollo Regional (FEDER), Unión Europea, “Una manera de hacer Europa” grants (PI16/0235 to Dr. R. Sánchez-Valle and PI17/00670 to Dr. A.Antonelli). AA is funded by Departament de Salut de la Generalitat de Catalunya, PERIS 2016-2020 (SLT002/16/00329). Work at JP-T laboratory was possible thanks to funding from Ciberned and generous gifts from Consuelo Cervera Yuste and Juan Manuel Moreno Cervera. Sydney Memory and Ageing Study (Sydney MAS): We gratefully acknowledge and thank the following for their contributions to Sydney MAS: participants, their supporters and the Sydney MAS Research Team (current and former staff and students). Funding was awarded from the Australian National Health and Medical Research Council (NHMRC) Program Grants (350833, 568969, 109308). This work was supported by InnoMed (Innovative Medicines in Europe), an integrated project funded by the European Union of the Sixth Framework program priority (FP6-2004- LIFESCIHEALTH-5). Oviedo: This work was partly supported by Grant from Fondo de Investigaciones Sanitarias-Fondos FEDER EuropeanUnion to V.A. PI15/00878. Project MinE: The ProjectMinE study was supported by the ALS Foundation Netherlands and the MND association (UK) (Project MinE, www.projectmine.com). The SPIN cohort: We are indebted to patients and their families for their participation in the “Sant Pau Initiative on Neurodegeneration cohort”, at the Sant Pau Hospital (Barcelona). This is a multimodal research cohort for biomarker discovery and validation that is partially funded by Generalitat de Catalunya (2017 SGR 547 to JC), as well as from the Institute of Health Carlos III-Subdirección General de Evaluación and the Fondo Europeo de Desarrollo Regional (FEDER- “Una manera de Hacer Europa”) (grants PI11/02526, PI14/01126, and PI17/01019 to JF; PI17/01895 to AL), and the Centro de Investigación Biomédica en Red Enfermedades Neurodegenerativas programme (Program 1, Alzheimer Disease to AL). We would also like to thank the Fundació Bancària Obra Social La Caixa (DABNI project) to JF and AL; and Fundación BBVA (to AL), for their support in funding this follow-up study. Adolfo López de Munain is supported by Fundación Salud 2000 (PI2013156), CIBERNED and Diputación Foral de Gipuzkoa (Exp.114/17). P.S.J. is supported by CIBERNED and Carlos III Institute of Health, Spain (PI08/0139, PI12/02288, and PI16/01652, PI20/01011), jointly funded by Fondo Europeo de Desarrollo Regional (FEDER), Unión Europea, “Una manera de hacer Europa”. We thank Biobanco Valdecilla for their support. ​Amsterdam dementia Cohort (ADC): Research of the Alzheimer center Amsterdam is part of the neurodegeneration research program of Amsterdam Neuroscience. The AlzheimerCenter Amsterdam is supported by Stichting Alzheimer Nederland and Stichting VUmc fonds. The clinical database structure was developed with funding from Stichting Dioraphte. Genotyping of the Dutch case-control samples was performed in the context of EADB (European Alzheimer&Dementia biobank) funded by the JPco-fuND FP-829-029 (ZonMW project number #733051061). This research is performed by using data from the Parelsnoer Institute an initiative of the Dutch Federation of University Medical Centres (www.parelsnoer.org). 100-Plus study: We are grateful for the collaborative efforts of all participating centenarians and their family members and/or relations. We thank the Netherlands Brain Bank for supplying DNA for genotyping. This work was supported by Stichting AlzheimerNederland (WE09.2014-03), Stichting Diorapthe, Horstingstuit foundation, Memorabel (ZonMW project number #733050814, #733050512) and Stichting VUmcFonds. Additional support for EADB cohorts: WF, SL, HH are recipients of ABOARD, a public-private partnership receiving funding from ZonMW (#73305095007) and Health~Holland, Topsector Life Sciences & Health (PPP-allowance; #LSHM20106). The DELCODE study was funded by the German Center for Neurodegenerative Diseases (Deutsches Zentrum für Neurodegenerative Erkrankungen (DZNE)), reference number BN012.

# REFERENCES

1 Das, S. *et al.* Next-generation genotype imputation service and methods. *Nat Genet* **48**, 1284-1287 (2016). <https://doi.org:10.1038/ng.3656>

2 P, D. *et al.* Twelve years of SAMtools and BCFtools. *GigaScience* **10** (2021). <https://doi.org:10.1093/gigascience/giab008>

3 Chang, C. C. *et al.* Second-generation PLINK: rising to the challenge of larger and richer datasets. *Gigascience* **4**, 7 (2015). <https://doi.org:10.1186/s13742-015-0047-8>

4 The Atherosclerosis Risk in Communities (ARIC) Study: design and objectives. The ARIC investigators. *Am J Epidemiol* **129**, 687-702 (1989).

5 Ryan, J. *et al.* Randomized placebo-controlled trial of the effects of aspirin on dementia and cognitive decline. *Neurology* (2020). <https://doi.org:10.1212/WNL.0000000000009277>

6 McNeil, J. J. *et al.* Effect of Aspirin on Disability-free Survival in the Healthy Elderly. *N Engl J Med* **379**, 1499-1508 (2018). <https://doi.org:10.1056/NEJMoa1800722>

7 McKhann, G. M. *et al.* The diagnosis of dementia due to Alzheimer's disease: recommendations from the National Institute on Aging‐Alzheimer's Association workgroups on diagnostic guidelines for Alzheimer's disease. *Alzheimer's & dementia* **7**, 263-269 (2011).

8 Zheng, X. *et al.* A high-performance computing toolset for relatedness and principal component analysis of SNP data. *Bioinformatics* **28**, 3326-3328 (2012). <https://doi.org:10.1093/bioinformatics/bts606>

9 Genomes Project, C. *et al.* A global reference for human genetic variation. *Nature* **526**, 68-74 (2015). <https://doi.org:10.1038/nature15393>

10 Fitzpatrick, A. L. *et al.* Incidence and prevalence of dementia in the Cardiovascular Health Study. *J Am Geriatr Soc* **52**, 195-204 (2004). <https://doi.org:10.1111/j.1532-5415.2004.52058.x>

11 Lopez, O. L. *et al.* Evaluation of dementia in the cardiovascular health cognition study. *Neuroepidemiology* **22**, 1-12 (2003). <https://doi.org:10.1159/000067110>

12 Bellenguez, C. *et al.* New insights on the genetic etiology of Alzheimer’s and related dementia. *medRxiv*, 2020.2010.2001.20200659 (2020). <https://doi.org:10.1101/2020.10.01.20200659>

13 OA, S. *et al.* A Validation Study of Vascular Cognitive Impairment Genetics Meta-Analysis Findings in an Independent Collaborative Cohort. *Journal of Alzheimer's disease : JAD* **53** (2016). <https://doi.org:10.3233/JAD-150862>

14 N, B. *et al.* Texture Features of Magnetic Resonance Images: an Early Marker of Post-stroke Cognitive Impairment. *Translational stroke research* **11** (2020). <https://doi.org:10.1007/s12975-019-00746-3>

15 C, B. *et al.* Identification of a specific functional network altered in poststroke cognitive impairment. *Neurology* **90** (2018). <https://doi.org:10.1212/WNL.0000000000005553>

16 V, Z. *et al.* Early MoCA predicts long-term cognitive and functional outcome and mortality after stroke. *Neurology* **91** (2018). <https://doi.org:10.1212/WNL.0000000000006506>

17 C, D. *et al.* Hippocampal Deformations and Entorhinal Cortex Atrophy as an Anatomical Signature of Long-Term Cognitive Impairment: from the MCAO Rat Model to the Stroke Patient. *Translational stroke research* **9** (2018). <https://doi.org:10.1007/s12975-017-0576-9>

18 YL, C. *et al.* Apolipoprotein ɛ4 is Associated with Dementia and Cognitive Impairment Predominantly Due to Alzheimer's Disease and Not with Vascular Cognitive Impairment: A Singapore-Based Cohort. *Journal of Alzheimer's disease : JAD* **51** (2016). <https://doi.org:10.3233/JAD-150902>

19 T, P., T, E., R, V. & M, K. Comparison of stroke features and disability in daily life in patients with ischemic stroke aged 55 to 70 and 71 to 85 years. *Stroke* **28** (1997). <https://doi.org:10.1161/01.str.28.4.729>

20 K, S. *et al.* Czech Brain Aging Study (CBAS): prospective multicentre cohort study on risk and protective factors for dementia in the Czech Republic. *BMJ open* **9** (2019). <https://doi.org:10.1136/bmjopen-2019-030379>

21 C, C., G, G., C, M. & G, M. Validity of some neuropsychological tests in the assessment of mental deterioration. *Acta psychiatrica Scandinavica* **60** (1979). <https://doi.org:10.1111/j.1600-0447.1979.tb00264.x>

22 C, B. *et al.* New insights into the genetic etiology of Alzheimer's disease and related dementias. *Nature genetics* **54** (2022). <https://doi.org:10.1038/s41588-022-01024-z>

23 D, T. *et al.* Sequencing of 53,831 diverse genomes from the NHLBI TOPMed Program. *Nature* **590** (2021). <https://doi.org:10.1038/s41586-021-03205-y>

24 McCarthy, S. *et al.* A reference panel of 64,976 haplotypes for genotype imputation. *Nat Genet* **48**, 1279-1283 (2016). <https://doi.org:10.1038/ng.3643>

25 J, M., B, H., S, M., G, M. & P, D. A new multipoint method for genome-wide association studies by imputation of genotypes. *Nature genetics* **39** (2007). <https://doi.org:10.1038/ng2088>

26 W, Z. *et al.* Efficiently controlling for case-control imbalance and sample relatedness in large-scale genetic association studies. *Nature genetics* **50** (2018). <https://doi.org:10.1038/s41588-018-0184-y>

27 Willer, C. J., Li, Y. & Abecasis, G. R. in *Bioinformatics* Vol. 26 2190-2191 (2010).

28 Dawber, T. R. & Kannel, W. B. The Framingham study. An epidemiological approach to coronary heart disease. *Circulation* **34**, 553-555 (1966). <https://doi.org:10.1161/01.cir.34.4.553>

29 Feinleib, M., Kannel, W. B., Garrison, R. J., McNamara, P. M. & Castelli, W. P. The Framingham Offspring Study. Design and preliminary data. *Prev Med* **4**, 518-525 (1975). <https://doi.org:10.1016/0091-7435(75)90037-7>

30 Splansky, G. L. *et al.* The Third Generation Cohort of the National Heart, Lung, and Blood Institute's Framingham Heart Study: design, recruitment, and initial examination. *Am J Epidemiol* **165**, 1328-1335 (2007). <https://doi.org:10.1093/aje/kwm021>

31 Beiser, A., D'Agostino, R. B., Sr., Seshadri, S., Sullivan, L. M. & Wolf, P. A. Computing estimates of incidence, including lifetime risk: Alzheimer's disease in the Framingham Study. The Practical Incidence Estimators (PIE) macro. *Stat Med* **19**, 1495-1522 (2000). <https://doi.org:10.1002/(sici)1097-0258(20000615/30)19:11/12><1495::aid-sim441>3.0.co;2-e

32 Bachman, D. L. *et al.* Incidence of dementia and probable Alzheimer's disease in a general population: the Framingham Study. *Neurology* **43**, 515-519 (1993). <https://doi.org:10.1212/wnl.43.3_part_1.515>

33 Farmer, M. E. *et al.* Neuropsychological test performance in Framingham: a descriptive study. *Psychol Rep* **60**, 1023-1040 (1987). <https://doi.org:10.2466/pr0.1987.60.3c.1023>

34 DeCarli, C. *et al.* Measures of brain morphology and infarction in the framingham heart study: establishing what is normal. *Neurobiol Aging* **26**, 491-510 (2005). <https://doi.org:10.1016/j.neurobiolaging.2004.05.004>

35 Au, R. *et al.* New norms for a new generation: cognitive performance in the framingham offspring cohort. *Exp Aging Res* **30**, 333-358 (2004). <https://doi.org:10.1080/03610730490484380>

36 Seshadri, S. *et al.* Lifetime risk of dementia and Alzheimer's disease. The impact of mortality on risk estimates in the Framingham Study. *Neurology* **49**, 1498-1504 (1997). <https://doi.org:10.1212/wnl.49.6.1498>

37 M, C. *et al.* A bird's-eye view of Italian genomic variation through whole-genome sequencing. *European journal of human genetics : EJHG* **28** (2020). <https://doi.org:10.1038/s41431-019-0551-x>

38 BN, H., P, D. & J, M. A flexible and accurate genotype imputation method for the next generation of genome-wide association studies. *PLoS genetics* **5** (2009). <https://doi.org:10.1371/journal.pgen.1000529>

39 GR, A. *et al.* An integrated map of genetic variation from 1,092 human genomes. *Nature* **491** (2012). <https://doi.org:10.1038/nature11632>

40 X, Z. & M, S. Efficient multivariate linear mixed model algorithms for genome-wide association studies. *Nature methods* **11** (2014). <https://doi.org:10.1038/nmeth.2848>

41 M, B. *et al.* Design of a comprehensive Alzheimer's disease clinic and research center in Spain to meet critical patient and family needs. *Alzheimer's & dementia : the journal of the Alzheimer's Association* **10** (2014). <https://doi.org:10.1016/j.jalz.2013.03.006>

42 MF, F., SE, F. & PR, M. "Mini-mental state". A practical method for grading the cognitive state of patients for the clinician. *Journal of psychiatric research* **12** (1975). <https://doi.org:10.1016/0022-3956(75)90026-6>

43 R, B. *et al.* Clinical validity of the 'mini-mental state' for Spanish speaking communities. *Neuropsychologia* **39** (2001). <https://doi.org:10.1016/s0028-3932(01)00055-0>

44 T, d. S. Q. *et al.* [Spanish version of the 7 Minute screening neurocognitive battery. Normative data of an elderly population sample over 70]. *Neurologia (Barcelona, Spain)* **19** (2004).

45 M, B., L, T., G, M., OL, L. & JL, C. [Neuropsychiatric Inventory-Nursing Home version (NPI-NH): Spanish validation]. *Neurologia (Barcelona, Spain)* **20** (2005).

46 B, R., SH, F., MJ, d. L. & T, C. The Global Deterioration Scale for assessment of primary degenerative dementia. *The American journal of psychiatry* **139** (1982). <https://doi.org:10.1176/ajp.139.9.1136>

47 JC, M. The Clinical Dementia Rating (CDR): current version and scoring rules. *Neurology* **43** (1993). <https://doi.org:10.1212/wnl.43.11.2412-a>

48 G, B., BE, T. & M, R. The association between quantitative measures of dementia and of senile change in the cerebral grey matter of elderly subjects. *The British journal of psychiatry : the journal of mental science* **114** (1968). <https://doi.org:10.1192/bjp.114.512.797>

49 M, A. *et al.* Cut-off scores of a brief neuropsychological battery (NBACE) for Spanish individual adults older than 44 years old. *PloS one* **8** (2013). <https://doi.org:10.1371/journal.pone.0076436>

50 GM, M. *et al.* The diagnosis of dementia due to Alzheimer's disease: recommendations from the National Institute on Aging-Alzheimer's Association workgroups on diagnostic guidelines for Alzheimer's disease. *Alzheimer's & dementia : the journal of the Alzheimer's Association* **7** (2011). <https://doi.org:10.1016/j.jalz.2011.03.005>

51 GC, R. *et al.* Vascular dementia: diagnostic criteria for research studies. Report of the NINDS-AIREN International Workshop. *Neurology* **43** (1993). <https://doi.org:10.1212/wnl.43.2.250>

52 RC, P. Mild cognitive impairment as a diagnostic entity. *Journal of internal medicine* **256** (2004). <https://doi.org:10.1111/j.1365-2796.2004.01388.x>

53 OL, L. *et al.* Prevalence and classification of mild cognitive impairment in the Cardiovascular Health Study Cognition Study: part 1. *Archives of neurology* **60** (2003). <https://doi.org:10.1001/archneur.60.10.1385>

54 F, J. *et al.* A conceptual framework for research on subjective cognitive decline in preclinical Alzheimer's disease. *Alzheimer's & dementia : the journal of the Alzheimer's Association* **10** (2014). <https://doi.org:10.1016/j.jalz.2014.01.001>

55 S, M.-G. *et al.* Genome-wide association analysis of dementia and its clinical endophenotypes reveal novel loci associated with Alzheimer's disease and three causality networks: The GR@ACE project. *Alzheimer's & dementia : the journal of the Alzheimer's Association* **15** (2019). <https://doi.org:10.1016/j.jalz.2019.06.4950>

56 S, D. *et al.* Next-generation genotype imputation service and methods. *Nature genetics* **48** (2016). <https://doi.org:10.1038/ng.3656>

57 R, S. *et al.* Age-Related Central Auditory Processing Disorder, MCI, and Dementia in an Older Population of Southern Italy. *Otolaryngology--head and neck surgery : official journal of American Academy of Otolaryngology-Head and Neck Surgery* **163** (2020). <https://doi.org:10.1177/0194599820913635>

58 CL, C., KCB, T. & AWC, K. Cohort Profile: The Hong Kong Osteoporosis Study and the follow-up study. *International journal of epidemiology* **47** (2018). <https://doi.org:10.1093/ije/dyx172>

59 JD, C. *et al.* Investigating the association between cancer and the risk of dementia: Results from the Memento cohort. *Alzheimer's & dementia : the journal of the Alzheimer's Association* **17** (2021). <https://doi.org:10.1002/alz.12308>

60 C, D. *et al.* Cognitive and imaging markers in non-demented subjects attending a memory clinic: study design and baseline findings of the MEMENTO cohort. *Alzheimer's research & therapy* **9** (2017). <https://doi.org:10.1186/s13195-017-0288-0>

61 O, N. *et al.* Achieving universal health coverage in France: policy reforms and the challenge of inequalities. *Lancet (London, England)* **387** (2016). <https://doi.org:10.1016/S0140-6736(16)00580-8>

62 M, G. *et al.* Prevalence of mild cognitive impairment by multiple classifications: The Monongahela-Youghiogheny Healthy Aging Team (MYHAT) project. *The American journal of geriatric psychiatry : official journal of the American Association for Geriatric Psychiatry* **18** (2010). <https://doi.org:10.1097/JGP.0b013e3181cdee4f>

63 Bennett, D. A. *et al.* The Rush Memory and Aging Project: study design and baseline characteristics of the study cohort. *Neuroepidemiology* **25**, 163-175 (2005). <https://doi.org:10.1159/000087446>

64 Bennett, D. A. *et al.* Natural history of mild cognitive impairment in older persons. *Neurology* **59**, 198-205 (2002). <https://doi.org:10.1212/wnl.59.2.198>

65 Bennett, D. A., Schneider, J. A., Bienias, J. L., Evans, D. A. & Wilson, R. S. Mild cognitive impairment is related to Alzheimer disease pathology and cerebral infarctions. *Neurology* **64**, 834-841 (2005). <https://doi.org:10.1212/01.WNL.0000152982.47274.9E>

66 RF, d. B. *et al.* The potential for prevention of dementia across two decades: the prospective, population-based Rotterdam Study. *BMC medicine* **13** (2015). <https://doi.org:10.1186/s12916-015-0377-5>

67 HP, H., MP, S., SP, G., SM, H. & LI, G. Ethnic differences in health knowledge and behaviors related to the prevention and treatment of coronary heart disease. The San Antonio Heart Study. *American journal of epidemiology* **117** (1983). <https://doi.org:10.1093/aje/117.6.717>

68 JP, B. *et al.* Rapid rise in the incidence of type 2 diabetes from 1987 to 1996: results from the San Antonio Heart Study. *Archives of internal medicine* **159** (1999). <https://doi.org:10.1001/archinte.159.13.1450>

69 S, H. *et al.* Association Between Subclinical Cardiac Biomarkers and Clinically Manifest Cardiac Diseases With Cortical Cerebral Microinfarcts. *JAMA neurology* **74** (2017). <https://doi.org:10.1001/jamaneurol.2016.5335>

70 S, H. *et al.* Subcortical Atrophy in Cognitive Impairment and Dementia. *Journal of Alzheimer's disease : JAD* **48** (2015). <https://doi.org:10.3233/JAD-150473>

71 Jacqmin-Gadda, H., Fabrigoule, C., Commenges, D., Letenneur, L. & Dartigues, J. F. A cognitive screening battery for dementia in the elderly. *J Clin Epidemiol* **53**, 980-987 (2000).

72 3C_Study_Group. Vascular factors and risk of dementia: design of the Three-City Study and baseline characteristics of the study population. *Neuroepidemiology* **22**, 316-325 (2003).

73 Amieva, H. *et al.* The 9 year cognitive decline before dementia of the Alzheimer type: a prospective population-based study. *Brain* **128**, 1093-1101 (2005). <https://doi.org:10.1093/brain/awh451>

74 Amieva, H. *et al.* Prodromal Alzheimer's disease: successive emergence of the clinical symptoms. *Ann Neurol* **64**, 492-498 (2008). <https://doi.org:10.1002/ana.21509>

75 Katz, S., Ford, A. B., Moskowitz, R. W., Jackson, B. A. & Jaffe, M. W. STUDIES OF ILLNESS IN THE AGED. THE INDEX OF ADL: A STANDARDIZED MEASURE OF BIOLOGICAL AND PSYCHOSOCIAL FUNCTION. *JAMA* **185**, 914-919 (1963).

76 Lawton, M. P. Scales to measure competence in everyday activities. *Psychopharmacol Bull* **24**, 609-614 (1988).

77 Rosow, I. & Breslau, N. A Guttman health scale for the aged. *J Gerontol* **21**, 556-559 (1966).

78 *Diagnostic and statistical manual of mental disorders. 4th ed.*, (APA, 1994).

79 RE, M. *et al.* GWAS on family history of Alzheimer's disease. *Translational psychiatry* **8** (2018). <https://doi.org:10.1038/s41398-018-0150-6>

80 A, G. *et al.* Leveraging family history in population-based case-control association studies. *Genetic epidemiology* **38** (2014). <https://doi.org:10.1002/gepi.21785>
